# Plethora of New Marsupial Genomes Informs Our Knowledge of Marsupial MHC Class II

**DOI:** 10.1093/gbe/evae156

**Published:** 2024-07-20

**Authors:** Luke W Silver, Carolyn J Hogg, Katherine Belov

**Affiliations:** School of Life and Environmental Sciences, The University of Sydney, Sydney, New South Wales 2006, Australia; School of Life and Environmental Sciences, The University of Sydney, Sydney, New South Wales 2006, Australia; Australian Research Council Centre of Excellence for Innovations in Peptide and Protein Science, University of Sydney, Sydney, New South Wales 2006, Australia; School of Life and Environmental Sciences, The University of Sydney, Sydney, New South Wales 2006, Australia; Australian Research Council Centre of Excellence for Innovations in Peptide and Protein Science, University of Sydney, Sydney, New South Wales 2006, Australia

**Keywords:** evolution, major histocompatibility complex, genomes, marsupial, comparative genomics

## Abstract

The major histocompatibility complex (MHC) plays a vital role in the vertebrate immune system due to its role in infection, disease and autoimmunity, or recognition of “self”. The marsupial MHC class II genes show divergence from eutherian MHC class II genes and are a unique taxon of therian mammals that give birth to altricial and immunologically naive young providing an opportune study system for investigating evolution of the immune system. Additionally, the MHC in marsupials has been implicated in disease associations, including susceptibility to *Chlamydia pecorum* infection in koalas. Due to the complexity of the gene family, automated annotation is not possible so here we manually annotate 384 class II MHC genes in 29 marsupial species. We find losses of key components of the marsupial MHC repertoire in the Dasyuromorphia order and the Pseudochiridae family. We perform PGLS analysis to show the gene losses we find are true gene losses and not artifacts of unresolved genome assembly. We investigate the associations between the number of loci and life history traits, including lifespan and reproductive output in lineages of marsupials and hypothesize that gene loss may be linked to the energetic cost and tradeoffs associated with pregnancy and reproduction. We found support for litter size being a significant predictor of the number of *DBA* and *DBB* loci, indicating a tradeoff between the energetic requirements of immunity and reproduction. Additionally, we highlight the increased susceptibility of Dasyuridae species to neoplasia and a potential link to MHC gene loss. Finally, these annotations provide a valuable resource to the immunogenetics research community to move forward and further investigate diversity in MHC genes in marsupials.

**Significance**

Marsupials are a unique taxon that give birth to underdeveloped and immunologically naïve young and therefore offer exciting opportunities to investigate a key immune gene family, the major histocompatibility complex (MHC). With the increase in the number of reference assemblies sequenced with long read, or scaffolding, technologies it has become possible to bioinformatically characterize the MHC genes. We report deletions of gene clusters in Dasyuridae species and generate hypotheses of the biological consequences in relation to reproduction and immunity.

## Introduction

Marsupials are a clade of therian mammals that diverged from eutherian mammals around 147 million years ago (MYA) and are distinguished from eutherians by unique reproductive and immune physiology (Bininda-Emonds et al. 2007). In contrast to eutherians, where the primary period of development occurs within the uterus, in marsupials, the majority of development occurs within the more exposed environment of the pouch (Old and Deane 2000). During development the immunologically naïve young rely on protection via placental transfer of immunoglobulins, the presence of immune compounds in milk, antimicrobial pouch secretion, and maternal cleaning of the pouch (Edwards et al. 2012). Early studies into the underlying genetics of the marsupial immune system determined the complexity of the mammalian immune system evolved before the divergence of marsupials and eutherians, though there are some notable expansions and losses (Belov et al. 1998, 1999a, 1999b, 2007). The combination of unique physiology and key differences in the immune system make marsupial an ideal system to study the development and evolution of the immune system.

The major histocompatibility complex (MHC) is a major component of the vertebrate immune system. The MHC region is one of the most gene-dense and polymorphic regions in the genome and plays a vital role in disease and recognition of “self” (Klein 1986; Kumanovics et al. 2003; Trowsdale and Parham 2004). The MHC consists of three classes of genes, class I, class II, and class III and contains numerous genes responsible for the presentation of antigens to the host immune system. Class I molecules are expressed on the surface of all nucleated cells and contain an α chain subunit, encoded within the MHC region and a β2-microglobulin found outside the MHC region. Class II MHC molecules are primarily expressed on antigen presenting cells and are responsible for presenting peptides from extracellular pathogens (primarily bacteria and parasites) to *T*-cells (Klein 1986). Class II molecules are heterodimers consisting of an α and β chain, both encoded for by class II A and class II B genes, respectively. The classical class II molecules are responsible for the direct binding and presentation of pathogen peptides whereas nonclassical class II molecules primarily serve as accessory proteins in antigen presentation (Alfonso and Karlsson 2000; Neefjes et al. 2011). Typically, class I genes undergo a more rapid rate of birth-and-death compared to class II genes, meaning orthology of class I genes is rarely seen across mammalian lineages (Hughes and Nei 1989). In contrast the class II genes show orthologous relationships between gene regions within various clades (eutherians, birds, marsupials) (Hughes and Nei 1990; Takahashi et al. 2000). Class II genes have a slower evolutionary rate than class I genes resulting in class II genes showing homology across orders of eutherian mammals, whereas class I genes tend to show little orthology across taxa (Hughes and Nei 1990; Kumanovics et al. 2003). This feature lends class II genes to being more adept for comparative studies (Hughes and Nei 1990; Zhang et al. 2019).

The classical class II MHC genes in marsupials show no orthology to the clusters seen in eutherians indicating these two lineages have retained different MHC gene clusters in early speciation events (Belov et al. 2004, 2006). The marsupial MHC class II have been given the nomenclature of DA, DB, and DC (Belov et al. 2004, 2006). Genes within the MHC are some of the most polymorphic genes with over 9,700 alleles identified in human class II genes to date (IPD- IMGT/HLA Database; (Robinson et al. 2020)). The high diversity of MHC genes is a result of co-evolution with pathogens which constantly evolve new ways of avoiding host detection and elimination (Jiang and Fares 2010). A large portion of variation in MHC genes is located within the peptide binding region (PBR) which allows binding to a wide range of peptides (Chen et al. 2022; Cheng et al. 2022; Martin et al. 2022; Gowane et al. 2023). High levels of diversity within the MHC genes are considered an important indicator of population health. However, diversity is not just restricted to mutations within MHC genes but also the overall number of MHC genes or copy number variation (CNV). Gene copy number varies between marsupial species (Belov et al. 2006; Cheng et al. 2009, 2012b; Siddle et al. 2011; Johnson et al. 2018; Morris et al. 2018), within species (Siddle et al. 2010; Mason et al. 2011) and within populations (Cheng et al. 2012b). This can be attributed to a birth-and-death evolutionary process where new MHC genes arise through gene duplication or are removed or become nonfunctional through the accumulation of deleterious mutations (Nei et al. 1997). It is hypothesized that there are fitness benefits associated with higher levels of MHC diversity as increased diversity allows a population to bind a broader range of pathogens (Hughes and Nei 1989; Sommer 2005), and that this diversity is maintained through pathogen-mediated balancing selection (Jeffery and Bangham 2000; Spurgin and Richardson 2010).

For species with genomic resources available, the MHC is often one of the first regions targeted and investigated due to its vital role in the immune response to infection. Some of the first work on MHC in marsupials occurred in Tasmanian devils (*Sarcophilus harrisii*). Devils are currently threatened by devil facial tumor disease (DFTD) which has reduced populations of devils by over 80% since its discovery in 1996 (Hawkins et al. 2006; Pearse and Swift 2006; McCallum 2008; Siddle et al. 2013). Development of MHC typing methods in devils showed that devils have low MHC diversity and many of the variants are shared with DFTD cells (Siddle et al. 2007, 2010; Belov 2011; Cheng et al. 2012a). The high similarity between the devil and tumor MHC as well as downregulation of MHC genes in tumor cells has resulted in rapid transmission of DFTD between devils (Siddle et al. 2013). In koalas (*Phascolarctos cinerus*), alleles of both class I and class II MHC genes have been implicated with the presence of clinical disease signs (such as wet bottom and conjunctivitis) associated with *Chlamydia pecorum* infection (Lau et al. 2014; Quigley et al. 2018; Robbins et al. 2020; Silver et al. 2022). Lower MHC class II heterozygosity has been associated with increased nematode parasitism in a wild population of Tasmanian devils (Chong 2020). Additionally, a study comparing MHC diversity of two species of opossum (*Gracilinanus microtarsus* and *Marmosops incanus*) determined that *M. incanus* showed low levels of diversity at *DAB* loci and high parasite load whereas *G. microtarsus* had higher levels genetic diversity at *DAB* loci and lower parasite load, highlighting the importance of maintaining diversity in MHC genes to respond to infection of a wide range of pathogens (Meyer-Lucht et al. 2010).

Until recently, investigating the MHC repertoire of species has been primarily assessed using PCR primers to amplify exon 2 and/or exon 3 (which encode the PBR) of MHC class I and II genes (Cheng et al. 2012a; Lau et al. 2013; Hermsen et al. 2017; Old et al. 2020). Genomes for non-model species are being generated at an exceptional rate by both individual research groups (Brandies et al. 2020; Peel et al. 2021; Feigin et al. 2022) and large international consortia (Koepfli et al. 2015; Dudchenko et al. 2017, 2018; Voolstra et al. 2017; Lewin et al. 2018; Teeling et al. 2018; Rhie et al. 2021). Despite the increase in the number of genomes assembled with long read and scaffolding technologies, there are still difficulties in assembling the MHC region, with an ultra-long read human genome able to assemble the MHC class I into a single contig but the class II region remained fragmented (Jain et al. 2018). Additionally, we have shown previously that manual annotation of complex immune genes, such as the MHC is insufficient and manual curation is required to uncover the true repertoire of MHC genes in a reference genome (Peel et al. 2022b).

The aim of this study was to investigate the MHC class II repertoire across the marsupial taxa to infer patterns of copy number evolution. Previous studies using either PCR amplification or bioinformatic characterization of MHC genes have identified a range in the number of MHC class II genes, for example, attempted PCR amplification of red-tailed phascogale and Tasmanian devil DBB has been unsuccessful (Cheng et al. 2012a; Hermsen et al. 2017) Similarly, bioinformatic characterization of MHC class II failed to identify genes belonging to the DB or DC class in the antechinus (Peel et al. 2022b). At the time, these results were explained by a lack of primer specificity or fragmentation of the genome assembly because of assembly with short reads (Hermsen et al. 2017; Peel et al. 2022b). There are now numerous marsupial genomes representative of most families, sequenced with a range of technologies including short read, long reads, and scaffolding technologies enabling investigation into the gain and loss of MHC class II genes through a comparative framework. In this study we bioinformatically characterized MHC class II genes of 29 marsupial species representing 15 families and six orders utilizing publicly available reference genomes and provide and test hypotheses as to how species biology relates to the wide range in the copy number of MHC genes across marsupials. We investigated whether there has been a tradeoff between reproduction and immunity in marsupials by testing the relationship between average litter size and MHC gene loss. This study represents a timely contribution to the growing body of marsupial immunology and evolutionary research.

## Results and Discussion

We downloaded 30 publicly available reference genomes for marsupials representing 29 species, 15 families, and six orders. We were unable to locate a genome of any species within the order Paucituberculata, or the families Hypsiprymnodontidae, Tarsipedidae, and Acrobatidae. Genome sizes ranged from 2.84 Gbp for the fat-tailed dunnart (*Sminthopsis crassicaudata)* to 3.82 Gbp for the eastern-barred bandicoot (*Perameles gunnii*) and a number of scaffolds from 17 for the monito del monte (*Dromiciops gliroides*) to 4,188,623 for the northern quoll (*Dasyurus hallucatus*) (supplementary table S1, Supplementary Material online). The quality of reference genomes varied with N50 values ranging from 0.055 Mbp for the eastern-barred bandicoot (*Perameles gunnii*) to 670.776 Mb for the monito del monte (supplementary table S1, Supplementary Material online). The reference genomes used have been generated with a range of technologies including solely short read (*N* = 4), a combination of short read and scaffolding (*N* = 18), short read and long read (*N* = 1) and short read, long read and scaffolding (*N* = 6) (supplementary table S1, Supplementary Material online).

**Fig. 1.**
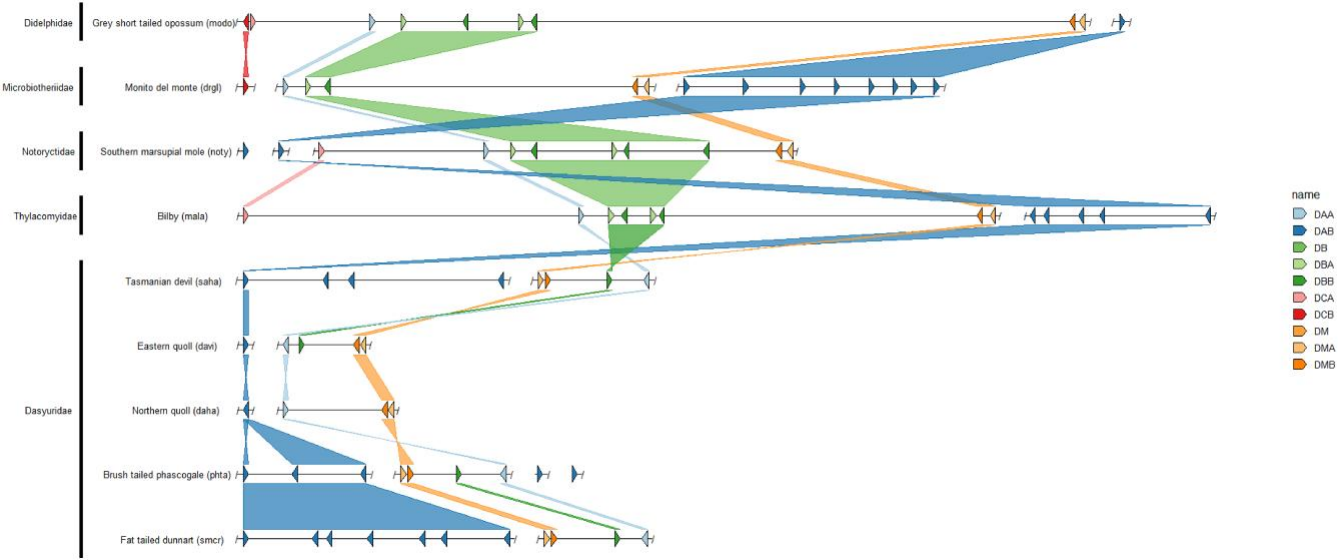
Genomic organization of class II MHC genes and synteny relationship between the gray short tailed opossum, Microbiotheriidae, Notoryctidae, Thylacomyidae and Dasyuridae species. The gray short tailed opossum is depicted as a model of a potential ancestral marsupial MHC organization. Dashed lines indication regions of the scaffold excluded for the purpose of clarity. Color of the synteny blocks is representative of the MHC locus (DA—blue; DB—green; DC—red; DM—orange).

**Fig. 2.**
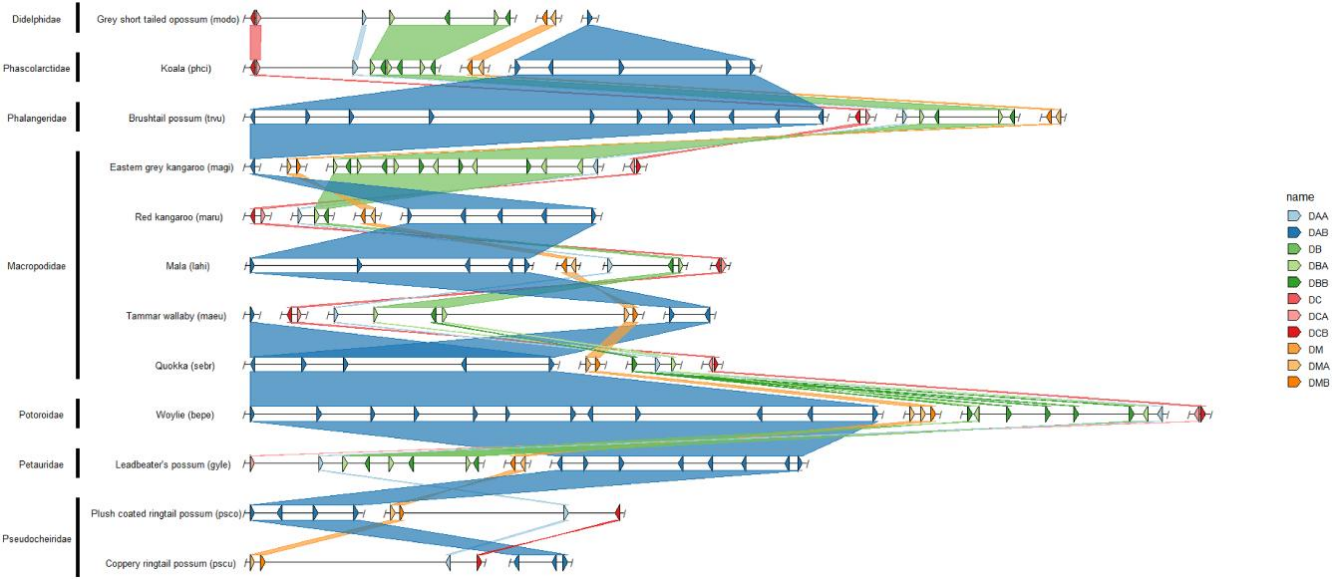
Genomic organization of class II MHC genes and synteny relationship between the gray short tailed opossum and diprotodontia species. The gray short-tailed opossum is depicted as a model of a potential ancestral marsupial MHC organization. Dashed lines indication regions of the scaffold excluded for the purpose of clarity. Color of the synteny blocks is representative of the MHC locus (DA—blue; DB—green; DC—red; DM—orange). Only names of scaffolds with multiple MHC genes are labeled.

In total we annotated 384 class II MHC genes across the 30 genomes, including 127 α chains and 257 β chains (Figs. 1 and 2, Table 1, supplementary table S2, Supplementary Material online, supplementary additional data S1, Supplementary Material online). A summary of MHC class II genes identified in marsupials is in Table 1 and more detailed results of the MHC gene repertoire can be found in the supplementary results, Supplementary Material online. As described by others, all species investigated contained varying numbers of loci for DA, DB, DC, and DM gene families (Belov et al. 2006; Cheng et al. 2012b; Lau et al. 2013; Hermsen et al. 2017), but by investigating representatives from the majority of marsupial families and mapping genes onto a phylogenetic tree allowed us to propose the order of gene loss and beyond that hypothesize about how species biology relates to the evolutionary trajectory of the MHC.

**Table 1:** Counts of each MHC class II gene in 30 genome assemblies of 29 species, numbers in brackets indicate partial genes (out of the total number of genes)

Species	Order	Family	*DAA*	*DAB*	*DBA*	*DBB*	*DCA*	*DCB*	*DMA*	*DMB*	Total
Gray short-tailed opossum (modo)	Didelphimorphia	Didelphidae	1	1	2	2	1	1	1	1	10
Monito del monte (drgl)	Microbiotheria	Microbiotheriidae	1	8	1	1	0	1 (1)	1	1	14 (1)
Southern marsupial mole (noty)	Notoryctemorphia	Notoryctidae	1	2	2	3	1 (1)	0	1	1	11 (1)
Bilby (mala)	Peramelemorphia	Thylacomyidae	1	5	2	2	1	0	1	1	13
Eastern-barred bandicoot (pegu)	Peramelemorphia	Peramelidae	1	3 (2)	1	1	1	0	1	1	9 (2)
Numbat (myfa)	Dasyuromorphia	Myrmecobiidae	1	4 (2)	0	1	0	0	0	0	6 (2)
Numbat DNAzoo (myfa_DNAzoo)	Dasyuromorphia	Myrmecobiidae	1	3 (3)	0	0	0	0	1	1 (1)	6 (4)
Tasmanian devil (saha)	Dasyuromorphia	Dasyuridae	1	4	0	1	0	0	1	1	8
Eastern quoll (davi)	Dasyuromorphia	Dasyuridae	1	1	0	1	0	0	1	1	5
Northern quoll (daha)	Dasyuromorphia	Dasyuridae	1	1	0	0	0	0	1	1	4
Brush-tailed phascogale (phta)	Dasyuromorphia	Dasyuridae	1	5 (2)	0	1	0	0	1	1	9 (2)
Antechinus (anst)	Dasyuromorphia	Dasyuridae	1	11 (4)	0	0	0	0	1	1	14 (4)
Fat-tailed dunnart (smcr)	Dasyuromorphia	Dasyuridae	1	7	0	1	0	0	1	1	11
Koala (phci)	Diprotodontia	Phascolarctidae	1	5	3	3	1	1	1	1	16
Common wombat (vour)	Diprotodontia	Vombatidae	1	1	1	0	1	1	1	1	7
Mountain pygmy possum (bupa)	Diprotodontia	Burramyidae	1	6 (4)	1	1	1	1	1	1	13 (4)
Ground cuscus (phgy)	Diprotodontia	Phalangeridae	1	7 (7)	2	1	1 (1)	1	1	1	15 (8)
Brushtail possum (trvu)	Diprotodontia	Phalangeridae	1	12	2	2	1	1	1	1	21
Eastern gray kangaroo (magi)	Diprotodontia	Macropodidae	1	1	7	5	1 (1)	1	1	1	18 (1)
Red kangaroo (maru)	Diprotodontia	Macropodidae	1	5	1	1	1 (1)	1	1	1	12 (1)
Western gray kangaroo (mafu)	Diprotodontia	Macropodidae	1	18 (16)	7	2	1 (1)	1	1	1	32 (17)
Mala (lahi)	Diprotodontia	Macropodidae	1	6 (1)	4 (3)	1	1 (1)	1	1	1	16 (5)
Tammar wallaby (maeu)	Diprotodontia	Macropodidae	1	3	2	2	1 (1)	1	1	1	12 (1)
Quokka (sebr)	Diprotodontia	Macropodidae	1	6 (1)	1	2	1 (1)	1	1	1	14 (2)
Woylie (bepe)	Diprotodontia	Potoroidae	1	12	2	5	1 (1)	1	2	1	25 (1)
Gilberts potoroo (pogi)	Diprotodontia	Potoroidae	1	6	4	3	1 (1)	1	1	1	18 (1)
Leadbeater's possum (gyle)	Diprotodontia	Petauridae	1	8	3	3	1	0	1	1	18
Plush coated ringtail possum (psco)	Diprotodontia	Pseudocheiridae	1	8 (4)	0	0	0	1 (1)	1	1	12 (5)
Western ringtail possum (psoc)	Diprotodontia	Pseudocheiridae	1	4 (2)	0	0	1 (1)	0	1	1	8 (3)
Coppery ringtail possum (pscu)	Diprotodontia	Pseudocheiridae	1	3	0	0	0	1 (1)	1	1	7 (1)

**Fig. 3.**
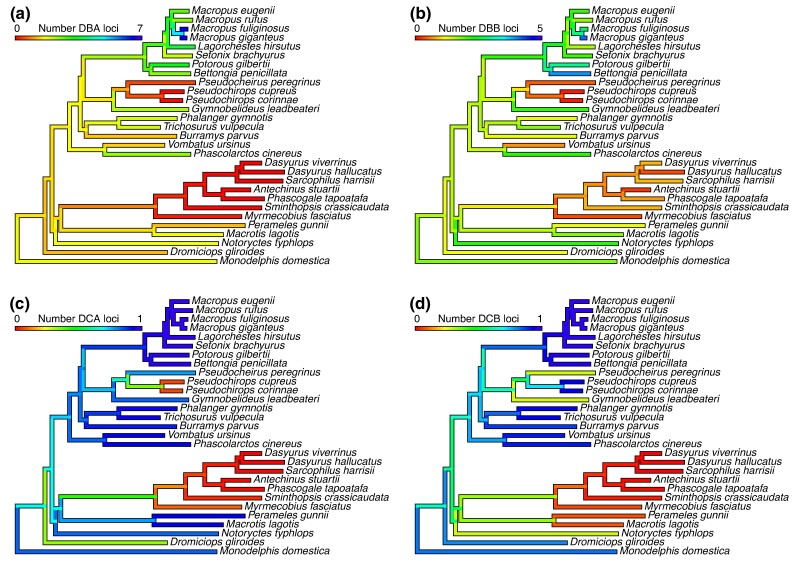
Projection of the copy number of actual present and reconstructed ancestral MHC class II genes onto the phylogeny of marsupial species for; a) *DBA*, b) *DBB*, c), *DCA*, d) *DCB*.

All marsupial species investigated had a single copy of *DAA*, *DMA,* and *DMB* except for two *DMA* genes found in the woylie (*Bettongia penicillata ogilbyi*) which given this has not been identified in any other species, may represent an error with the genome assembly falsely duplicating this gene. This may be explained by the Woylie reference genome being of comparatively lower quality to some of the other genomes investigated in this study as the genome was assembled without scaffolding data, and the specimen used was a roadkill individual meaning there is the potential for DNA degradation to have occurred prior to cold storage (Peel et al. 2021). All species had at least one copy of the *DAB* gene, but up to 12 were identified in both the woylie and brushtail possum (*Trichosurus vulpecula*). The *DAB* genes were more numerous than *DBB* genes in all species, except for the eastern gray kangaroo (*Macropus giganteus*). In the Peramelidae, Thylacomyidae, and Notoryctidae families no *DCB* gene was identified, the loss of the *DCB* gene results in a nonfunctional DC protein. Additionally, in the Microbiotheriidae only a partial *DCB* and no *DCA* gene could be identified even though the genome has a high N50 value and 94% complete BUSCOs (supplementary data S1, Supplementary Material online). It is therefore likely that the failure to locate *DCB* and *DCA* was not due to assembly fragmentation (Peel et al. 2022b). In the Notoryctidae only a partial *DCA* was located, indicating that the loss of one chain of an MHC molecule may relax selective pressure on genes that encode the other chain of the molecule. Species in the Dasyuridae and Myrmecobiidae families have no *DBA*, *DCA,* or *DCB* genes in their genomes, resulting in nonfunctional DB and DC proteins. Based on these findings, we hypothesize that the *DCB* gene had been lost in the ancestor of the polyprotodontia (Dasyuridae, Myrmecobiidae, Notoryctidae, Thylacomyidae, Peramelidae) and undergone pseudogenization in the ancestor of the Microbiotheriidae (Fig. 3). Additionally, we hypothesize that the *DBA* and *DCA* had been lost in the ancestor of the Dasyuidae and Myrmecobiidae. Furthermore, no *DBA* or *DBB* genes were identified in the Pseudocheiridae indicating the loss of the DB gene in this family (Fig. 3) and only partial sequences of either *DCA* or *DCB* could be identified.

To investigate whether our inability to identify MHC class II genes results from genome quality or are likely to represent real gene losses we ran a PGLS analysis for each gene cluster with variable numbers of loci (*DAB, DBA, DBB, DCA,* and *DCB*) with predictor variables of genome N50 and BUSCO completeness. From our analysis, only the number of *DAB* loci was found to be significantly impacted by genome N50 (*F* = 5.696, *P* = 0.025) and BUSCO completeness (*F* = 5.088, *P* = 0.033). The values of the model suggest that as BUSCO completeness increases so does the number of *DAB* loci recovered, and as genome N50 decreases the number of *DAB* loci recovered increases (supplementary table S3, Supplementary Material online). It has been shown previously, in marsupials, that as genome quality increases more immune genes are able to be recovered from the assembly (Peel et al. 2022b). For the gene families where we have hypothesized gene losses, we found no significant associations with genome quality metrics (supplementary table S3, Supplementary Material online). Additionally, by analyzing the MHC gene repertoire in a comparative framework with multiple species representing each family we can have higher certainty that the losses are in fact true gene losses and not an artifact of genome mis-assembly. Losses of MHC genes are not uncommon as MHC genes evolve through a rapid birth-and-death model through duplications, deletions, and rearrangements (Nei et al. 1997).

To provide further evidence that gene losses have a taxonomic correlation, we performed a phylogenetic correlation analysis and determined the number of loci at *DBA* (*I* = 0.389, *P* = 0.043), *DBB* (*I* = 0.443, *P* = 0.026), *DCA* (*I* = 0.558, *P* = 0.008) and *DCB* (*I* = 0.545, *P* = 0.01) have a correlation to the Family level and the number of *DCA* (*I* = 0.619, *P* = 1.11 ×10^−6^) and *DCB* (*I* = 0.654, *P* = 3.29 ×10^−7^) loci have an Order level correlation (supplementary table S4, Supplementary Material online). By mapping these gene loss events onto a species tree, we can hypothesize two gene loss events, one in the ancestor of the Notoryctemorphia, Peramelemorphia and Dasyuromorphia where the *DCB* gene was lost and a second in the ancestor of the Dasyuromorphia, where the *DCA* and *DBA* genes were lost (Fig. 3). A similar pattern is seen within the felids which are missing the DQ region and have a pseudogenized DP region (Yuhki et al. 2008). Cetaceans also appear to be missing *DPA* genes, while only fragments of the *DPB* gene have been identified (Zhang et al. 2019). From our phylogenetic gene trees the majority of gene clusters form monophyletic groups, with DC genes most closely clustering to DA genes (supplementary figs. S1 and S2, Supplementary Material online). The eutherian DP and DR genes are most closely related to the marsupial DC genes and the marsupial DA genes (supplementary figs. S1 and S2, Supplementary Material online). The eutherian DQ and DO genes also show orthology to the marsupial DB genes (supplementary figs. S1 and S2, Supplementary Material online) Whilst the bootstrap supports for each gene are greater than 50, some of the bootstrap supports within each gene are much lower, reflecting the rapid evolution of MHC genes within lineages (supplementary figs. S1 and S2, Supplementary Material online). Lower overall bootstrap values within the phylogenetic tree for MHC class II B genes indicate these genes are more similar to one another and further phylogenetic investigation is needed to resolve the relationship. We found similar patterns of evolution occurring across class II genes, with the Brownian model of evolution the best fit for *DCA* and *DCB* and Brownian motion scaled with *λ* the best model for *DAB*, *DBA,* and *DBB* (supplementary table S5, Supplementary Material online)

Immunity is an energetically demanding process and there are tradeoffs between increased energy expenditure on immunity and decreased reproductive output and vice versa (Schwenke et al. 2016; Hawash et al. 2021). For example the Dasyuridae family typically have a short life span (1 to 6 year) with a high reproductive input which in the case of the genus *Antechinus* results in a synchronized mating period followed by reduced immunoglobulin concentration and increased parasite load followed by total male mortality (a phenomenon known as semelparity) (Bradley et al. 1980; Naylor et al. 2008). Dasyurids appear to make a larger energetic investment into reproduction (Tyndale-Biscoe and Renfree 1987), with average litter sizes higher than those marsupial species with a full complement of MHC class II genes. For example, the northern quoll has an average litter size of 6.4 offspring and only four class II genes compared to the koala that has an average litter size of one offspring per female and has 16 class II genes (Table 1) (Tyndale-Biscoe and Renfree 1987). It is possible that variation in the presence/absence of MHC loci has evolved in parallel to the range of reproductive strategies and overall energetic investment into reproduction observed in marsupials. To better understand this hypothesis, we performed PGLS and BPMM analysis with the number of loci for each gene (*DAB, DBA, DBB, DCA,* and *DCB*) modeled against residual lifespan and the ratio of average offspring to number of teats. We chose the ratio of offspring to teats as a predictor variable, as marsupials have differing numbers of teats and therefore the number of teats is a limiting factor in the number of offspring that can be produced per litter. Our PGLS analysis indicated that the ratio of offspring to number of teats is a significant predictor of the number of *DBB* loci (*F* = 4.27, *P* = 0.049), with the model indicating as the ratio of offspring to number of teats increases (i.e. higher reproductive output) the number of *DBB* loci decreases (supplementary table S6, Supplementary Material online). We found that the ratio of offspring to teat number was a significant predictor for the number of *DBA* (HPD CI: [−6.06, −1.43]) and *DBB* (HPD CI: [−4.329, −0.074]) loci (supplementary table S7, Supplementary Material online). Future studies that investigate immune gene expression during gestation across a range of marsupials may elucidate the role of MHC in marsupial pregnancy. The bilby and bandicoots are characterized by their extremely rapid rate of reproduction with a gestation length of just 12.5 d in some species (Tyndale-Biscoe and Renfree 1987). Bilbies and bandicoots are also the only marsupials to have a chorioallantoic placenta that supports rapid development of the young (Tyndale-Biscoe and Renfree 1987; Renfree 2010). The increased energetic cost of this mode of reproduction may have contributed to our finding of a reduced repertoire of MHC loci in these species. During pregnancy trophoblast cells (the cells forming the outer layer of a blastocyst) downregulate expression of MHC class I genes and completely supress class II gene expression (Bacon et al. 2002; Holtz et al. 2003) in order to avoid detection by maternal immune responses. Further to this, early MHC class I investigations in the bilby show fewer class I genes compared to other marsupials (seven compared to 16 in the koala (Peel et al. 2022b)), with an increased number of partial class I genes highlighting the need for further investigations into the interplay between reproduction and immunity in marsupials (Hogg et al. 2024).

Each individual allele of an MHC gene broadens the spectrum of pathogens capable of being recognized by an individual and higher polymorphism within MHC genes has been associated with increased pathogen tolerance (Madsen and Ujvari 2006; Meyer-Lucht et al. 2010; Minias et al. 2019). Diversity in the number of MHC genes provides an additional level of diversity to an individual. For example, Minias et al. (2019) identified that CNV in MHC class II B genes was associated with migratory distance in avian species, with the assumption that long-distance migrators are exposed to broader taxonomic array of pathogens and it is therefore selectively beneficial to harbor an increased number of MHC genes (Altizer et al. 2011). Minias et al. (2019) also identified that the number of class I genes in birds increased with average lifespan, under the assumption that longer lived species will encounter higher numbers of pathogens over their lifespan. In our study, we identify similar patters with shorter lived species, such as Dasyurids, having fewer copies of MHC class II genes compared to longer lived species, such as Macropods. To test this hypothesis, we also included residual lifespan in our models. In our BPMM analysis we found residual lifespan was a significant predictor of the number of *DAB* (HPD CI: [0.456, 16.961]) loci (supplementary table S7, Supplementary Material online). We hypothesize that the higher number of MHC genes in Macropodidae and Potoroidae species provides increased fitness by increasing the range of pathogens able to be recognized and defended against.

Our finding that the Dasyuromorphia order rely solely on DA molecules as a part of their classical class II system is significant as Dasyurids are far more prone to developing cancerous lesions when compared to other species (Canfield et al. 1990; Peck et al. 2019; Flies et al. 2020) which may in part be explained by these species only having a functional DA and DM gene in their MHC class II repertoire. MHC class II has been implicated in susceptibility to cancer in Californian sea lions (*Zalophus californianus*), which generate diversity in class II genes primarily through CNV rather than SNPs and the presence of the *DRB*. A locus was associated with increased risk of cancer (Bowen et al. 2004, 2005). Cervical carcinoma in humans has been positively associated with the presence of the DQB1*03 allele and negatively associated with the DRB1*13 allele (Wank and Thomssen 1991; Nawa et al. 1995; Lin et al. 2001). Tasmanian devils are susceptible to two of only nine known transmissible cancers, and only two of the three in vertebrates (Hawkins et al. 2006; Murgia et al. 2006; Metzger et al. 2016; Pye et al. 2016; Yonemitsu et al. 2019). In addition to low diversity at MHC class I genes (Siddle et al. 2007; Cheng et al. 2012b), a reduced number of MHC genes may have lowered the histocompatibility barrier resulting in transmission of tumor cells between individual devils (Siddle et al. 2010). It has been noted that there are distinct similarities between the way devil tumor cells and trophoblast cells in the foetus avoid detection by the immune system (Moffett and Loke 2006; Siddle et al. 2013) and it has been shown that there are similarities between the cellular behavior of cancerous and trophoblast cells (Khorami Sarvestani et al. 2023). Furthermore, Dujon et al. (2023) have determined that cancer is more likely to occur in species with larger litter sizes, longer lactation periods and a semi-invasive placenta emphasizing the interplay between reproduction, cancer and immunity.

Pseudochiridae contain the ringtail possums, three of which we investigate here, the western ringtail possum (*Pseudocheirus peregrinus occidentalis*), coppery ringtail possum (*Pseudochirops cupreus*) and plush coated ringtail possum (*Pseudochirops corinnae*) (Dudchenko et al. 2017, 2018). All species contained a single copy of the *DAA* and both DM genes and copies of *DAB* varied from three (coppery ringtail possum) to eight (plush coated ringtail possum). For the DC molecule only partial *DCA* sequence could be located in the western ringtail possum genome and partial *DCB* sequences in the coppery and plush coated ringtail possums and neither DB gene could be identified in any of the three assemblies (Figs. 2 and 3).

Finally, this research unequivocally supports the idea that the complexity of the MHC system evolved prior to the divergence of the eutherian mammals and marsupial lineages (Belov et al. 2004, 2007). However, within the marsupial lineage, there have been at least three deletion events, one resulting in the loss of the *DCB* gene in the ancestor of the polyprotodonts, one resulting in the loss of the *DCA* and *DBA* in the ancestor of the Dasyuromorphia clade, and one resulting in the loss of the *DBA* and *DBB* genes in the ancestor of the Pseudocheiridae family. This study represents the largest marsupial MHC investigation to date, with a total of 384 MHC class II genes annotated across 29 species.

## Methods

We downloaded available marsupial reference genomes from NCBI, DNAZoo (https://www.dnazoo.org/) and the Australasian Genomes github (https://awgg-lab.github.io/australasiangenomes/). We calculated basic genome quality statistics such as number of scaffolds and N50 using BBmap v37.98 (http://sourceforge.net/projects/bbmap) and BUSCO completeness using BUSCO v5.3.2 on the public server; galaxy.org.au (Simao et al. 2015) (supplementary table S1, Supplementary Material online). Where multiple assemblies were available for the same species, we chose the most recent version (with the exception of the numbat as we were unable to annotate the entire class II repertoire in either of the two assemblies and so used both assemblies to assess the MHC class II repertoire) (Dudchenko et al. 2017, 2018; Peel et al. 2021, 2022a). To investigate MHC class II genes we used BLASTn searches (v.2.2.30), using default parameters, (Altschul et al. 1990) with known marsupial MHC sequences as queries (Belov et al. 2006; Cheng et al. 2018; Silver et al. 2022; Peel et al. 2022b). BLASTn results were inspected manually and potential genes were visualized in IGV (v.2.11.2) (Robinson et al. 2011) to identify accurate exon boundaries following the AG/GT convention. A gene was considered complete if the expected number of exons were present in the BLAST output or partial if not all exons could be located within the genome assembly. Complete exon sequences were extracted from the reference genome using Bedtools (v.2.29.2) Getfasta (Quinlan and Hall 2010). Genomic organization and synteny of MHC genes was visualized using the gggenomes package (Hackl and Ankenbrand 2022) in R v4.2.1 (R Core Team 2022). Genomic organization and synteny were only investigated for those species where most class II genes were present on a single scaffold as it is known that class II MHC genes in marsupials occur in a cluster on single chromosome (Belov et al. 2006; Deakin et al. 2007) and so assemblies with MHC class II genes present on multiple scaffolds can be said to be as a result of fragmentation of the genome assembly. Pairwise synteny between scaffolds containing the MHC class II genes of gray short-tailed opossum, greater bilby, Tasmanian devil and koala were investigated using mummer4 v4.0.0 (Marcais et al. 2018) and dot plots were visualized using ggplot2 (Wickham 2016) in R v4.2.1 (R Core Team 2022). These four species were chosen as the annotated genes were all located on a single scaffold, these species were chosen to be representative of the gene losses which have occurred within each of these taxonomic families. Nucleotide sequences were aligned for each gene cluster (DA, DB, DC, and DM) with human class II sequences (DQ, DP, DR, and DM) using muscle in MEGAX (Kumar et al. 2018). Maximum likelihood trees were constructed using a general time reversible model with a gamma distribution (Nei and Kumar 2000; Kumar et al. 2018) with 1,000 bootstrapping replicates and the tree was rooted using already annotated reptile class II genes (FJ623746.1, FJ623749.1, and DQ124231.1). Reptile class II MHC genes were used to root our trees, as they are evolutionarily distinct enough to form a distinct group from therian MHC class II genes. The maximum likelihood “Find Best DNA/RNA Model” function in MEGAX was used to determine the best model for generating the phylogenetic tree. The number of loci for each gene was mapped onto a representative marsupial phylogenetic tree (Mitchell et al. 2014) using the contMap function as part of the phytools package (Revell 2012) in R v.4.2.1. Abbreviations used for naming the MHC class II genes followed convention of the first two letters of the genus and species as follows; *anst* brown antechinus, *bepe* woylie, *bupa* mountain pygmy possum (*Burramys parvus*), *daha* northern quoll, *davi* eastern quoll, *drgl* monito del monte, *gyle* Leadbeater's possum (*Gymnobelideus leadbeateri*), *hosa* human, *lahi* Mala (*Lagorchestes hirsutus*), *maeu* tammar wallaby, *mafu* western gray kangaroo (*Macropus fuliginosus*), *magi* eastern gray kangaroo, *mala* bilby, *maru* red kangaroo (*Macropus rufus*), *modo* gray short-tailed opossum, *myfa* numbat, *myfa*_DNAzoo numbat DNAzoo assembly, *noty* southern marsupial mole, *pegu* eastern-barred bandicoot, *phci* koala, *phgy* ground cuscus (*Phalanger gymnotis*), *phta* brush-tailed phascogale, *pogi* Gilbert's potoroo (*Potorous gilbertii*), *psco* plush coated ringtail possum, *pscu* coppery ringtail possum, *psoc* western ringtail possum, *saha* Tasmanian devil, *sebr* quokka (*Setonix brachyurus*), *smcr* fat-tailed dunnart, *trvu* brushtail possum, *vour* common wombat (*Vombatus ursinus*).

To investigate whether the variation in the number of class II genes recovered could be because of genome quality assembly, we ran phylogenetic generalized least squared (PGLS) models (Martins and Hansen 1997) for each gene with variation in the number of loci recovered (*DAB*, *DBA*, *DBB*, *DCA,* and *DCB*). We used the marsupial phylogeny determined by Mitchell et al. (2014), retaining the 29 species used in this study for all models. Firstly, we identified the most appropriate evolutionary model for each gene by fitting three models. The first model fitted was Brownian motion which is based on neutral evolution where a trait evolves purely under genetic drift (Edwards 1970; Felsenstein 1985). Secondly, Brownian motion with a scaling parameter *λ*, where *λ* can vary between 0 and 1, and the branches of the phylogenetic tree a multiplied by the value of *λ* meaning values close to 1 indicating the trait is evolving under typical the Brownian motion model and values closer to 0 indicating all species are equally related (Pagel 1999). The third model tested was Ornstein-Uhlenbeck (OU) which describes the evolution of a trait to a constant optimum (Felsenstein 1988; Hansen 1997). All models were fitted with the fitContinuous function in the geiger package (Harmon et al. 2008) in R v4.2.1. The best model was chosen based on Akaike weights (*ω*_i_). Using the best evolutionary model for each gene we ran PGLS models of the number of loci for each gene as a function of genome N50 and BUSCO completeness (calculated above). PGLS models were fitted using the gls function in the ape package (Paradis et al. 2004) in R v4.2.1.

To test for phylogenetic signals within our data we ran phylogenetic correlograms of Moran's *I* against taxonomic levels (Order/Family/Genus) for each gene using the correlogram.formula function in the ape package (Paradis et al. 2004) in R v4.2.1.

We obtained data on life history traits, including maximum lifespan (obtained from AnAge database (de Magalhães et al. 2009) and other sources (Smith 2004; Wayne et al. 2005; Stead-Richardson et al. 2010)), weight (obtained from AnAge database (de Magalhães et al. 2009)), number of teats and average offspring per litter (Tyndale-Biscoe and Renfree 1987). For our models we coded for reproductive output as a percentage of the average offspring per litter out of the maximum possible offspring (based off teat number) and residual lifespan which was calculated by accounting for allometry (Witting 2008) by modeling the log_10_ of maximum lifespan against log_10_ weight and calculating the residual between the actual and predicted lifespan. For our two life history traits we assessed phylogenetic correlation with taxonomy (Order/Family/Genus) using the correlogram.function as above. As these traits have a phylogenetic basis we ran both a PGLS and a Bayesian phylogenetic mixed model (BPMM) to investigate whether MHC class II gene gain and loss may be influenced by life history. Estimates with a 95% HPD CI excluding zero were taken as statistically significant at *α* = 0.05. For PGLS analysis, we used the appropriate evolutionary model as determined by above using the gls function in the ape package (Paradis et al. 2004) in R v4.2.1. For BPMM we used the MCMCglmm package (Hadfield 2010) in R v4.2.1, we set uninformative priors and for each analyses ran 2,500,000 iterations with a burnin of 150,000 and thinning intervals of 1,500. Model diagnostics were assessed to ensure autocorrelation <0.1 and a Heidelberg stationary test and Gelmen-Rubin statistic were used to confirm chain convergence across three runs of each model (Heidelberger and Welch 1981, 1983; Gelman and Rubin 1992; Brooks and Gelman 1998). We chose the best model out of the three by the lowest Deviance Information Criterion (DIC). For both modeling methods we ran a different model for each gene with variation in the number of loci (DAB, DBA, DBB, DCA, and DCB).

## Supplementary Material

evae156_Supplementary_Data

## Data Availability

All genomes used in this study, apart from the mountain pygmy possum and eastern-barred bandicoot, are freely accessible online. All MHC sequences identified in this study are provided as Supplementary material with annotations in a gff format in supplementary table S2, Supplementary Material online and sequences in fasta format in supplementary results to the article.
