# Supplementary material for: Plethora of New Marsupial Genomes Informs Our Knowledge of Marsupial MHC Class II
Source: Genome Biol Evol. 2024 Jul 20;16(8):evae156. doi: 10.1093/gbe/evae156 (PMC11305139; doi:10.1093/gbe/evae156)
Supplement: evae156_Supplementary_Data [file evae156_supplementary_data.zip › Supplementary Results.docx]

## Supplementary Results

# Plethora of new marsupial genomes informs our knowledge of marsupial MHC

Luke W. Silver^1^, Carolyn J. Hogg^1,2^, Katherine Belov^1,2^

^1^ School of Life and Environmental Sciences, The University of Sydney, New South Wales, 2006, Australia.

^2^ Australian Research Council Centre of Excellence for Innovations in Peptide and Protein Science, University of Sydney, Sydney NSW 2006, Australia

Corresponding Author: Carolyn J. Hogg,^1^ School of Life and Environmental Sciences, The University of Sydney, New South Wales, 2006, Australia.

Email: [Carolyn.hogg@sydney.edu.au](mailto:Carolyn.hogg@sydney.edu.au)

## Didelphidae

We investigated a single species in the Didelphidae family, the grey short-tailed opossum (Olga Dudchenko et al., 2018; Edwards & Westerman, 1995; Mikkelsen et al., 2007). We identified ten MHC class II genes all located on a single scaffold with each gene present in a single copy except the DB genes where two copies of both the α and β were identified. In the grey short-tailed opossum we found genes encoding each chain of the DB, DC and DM molecules adjacent to one another and the alpha and beta genes of the DA molecule separated by the genes of the two DB and the DM molecule (Figure 1, (Belov et al., 2006)). All genes identified were full length sequences in a genome that had 5,136 scaffolds and 78.7% BUSCO completeness.

## Microbiotheriidae

Microbiotheriidae is a single species family, the monito del monte, which is a South American species of marsupial. We identified 14 genes in the monito del monte, eight of these are DAβ genes and a single copy of the DAα and both the DB and DM genes. All genes identified in the monito del monte showed synteny with the opossum with the exception of the DCβ gene which has been inverted (Figure 1). We failed to identify any DCα genes and could only identify a partial DCβ gene. All sequences were located on a single scaffold, the genome had 17 scaffolds and was 93.9% complete.

## Notoryctidae

The Notoryctidae family contains two species of marsupial moles, we investigated the southern marsupial mole (O. Dudchenko et al., 2017; Olga Dudchenko et al., 2018) and identified 11 genes. A single copy of the DAα and both DM genes, two copies of DAβ and DBα and three copies of DBβ were identified. We failed to locate a DCβ and could only identify a partial DCα gene. Sequences were located across two scaffolds, one containing only a single DAβ gene. This genome was 87.0% complete with 10,900 scaffolds.

## Thylacomyidae

Thylacomyidae contains a single extant species, the greater bilby with the lesser bilby going extinct in the 1960s. We located 13 genes, a single copy of DAα, DCα and both DM genes, two copies of both DB genes and five copies of DAβ. We were unable to locate a DCβ gene and all sequences were located on a single scaffold. This draft genome was 88.5% complete with 663 scaffolds (C. Hogg, unpublished data).

## Peramelidae

The Peramelidae family contains a number of bandicoot species occurring in New Guinea and Australia, we investigated one species from this family, the eastern barred bandicoot. We identified nine MHC class II genes, a single complete copy of DCα and both DA, DB and DM genes and two partial DAβ sequences. We did not locate a DCβ gene and the nine genes identified were located across seven scaffolds. This draft genome was 65.2% complete with 170,614 scaffolds (A. Weeks, unpublished data).

## Myrmecobiiidae

The Myrmecobiidae family contains a single species, the numbat which feed exclusively on termites. We investigated two genome assemblies for this species, one assembled using solely short linked-read data (Peel et al., 2022) and the other assembled using a combination of both short read and HiC data (O. Dudchenko et al., 2017; Olga Dudchenko et al., 2018). We used both assemblies as neither assembly contained the complete MHC class II repertoire. The short read assembly contained six genes, a single DAα and DBα, two complete and two partial DAβ genes. The HiC assembly contained six genes, a single DAα gene (this gene was split across two scaffolds in the assembly) and DMα gene as well as three partial DAβ and a partial DMβ gene. From the combination of the two assemblies we can conclude the numbat has at least a single DAα, both DB and DM genes and three DAβ genes. The short linked-read genome had 112,299 scaffolds and was 73.2% complete, whilst the DNAZoo genome had 110,7131 scaffolds and was 73.2% complete.

## Dasyuridae

The Dasyuridae family contains a number of insectivores and carnivorous species which are primarily nocturnal. We investigated six species of dasyurids here: Tasmanian devil (106 scaffolds, 91.7% complete), eastern (386,594; 84.6%) and northern quolls (4,188,623 scaffolds, 92.5%), brush tailed phascogale (295,136 scaffolds, 89.0%), antechinus (30,876 scaffolds and 92.5%) and fat tailed dunnart (1,644 scaffolds and 85.0%) (Brandies, Tang, Johnson, Hogg, & Belov, 2020; O. Dudchenko et al., 2017; Olga Dudchenko et al., 2018; Stammnitz et al., 2023). We identified between four (northern quoll) and 14 (antechinus) MHC class II genes. All species have a single copy of DAα, DBβ (except the northern quoll, where no DBβ was located) and both DM genes. The DAβ genes ranged in number from one in the northern and eastern quolls to 11 in the antechinus. The DBα or DC gene were unable to be identified in any of the dasyuridae species.

## Phascolarctidae

The Phascolarctidae family contains a single species, the koala (O. Dudchenko et al., 2017; Olga Dudchenko et al., 2018; Johnson et al., 2018). We identified 16 MHC class II genes in the koala genome, a single copy of DAα, both DC and DM genes, three copies of both DB genes and five copies of the DAβ gene. These genes were located on a single scaffold with an identical organisation to the grey short-tailed opossum MHC with the DC genes upstream from the DAα, which is separated from the DAβ genes by the DB and DM genes (Figure 4) showing conserved genomic organisation of the class II MHC across the Didelphidae and Diporotodontia lineages. The conserved genomic organisation is shown further by the synteny plot between scaffold 2 and scaffold 3 of the opossum and koala, respectively (Supp. Figure 3).

## Vombatidae

The Vombatidae family contain three species of wombat, one of which we investigated here, the common wombat (O. Dudchenko et al., 2017; Olga Dudchenko et al., 2018). We located seven genes, a single copy of each class II gene with the exception of DBβ where no gene was located, and all seven genes were located on a single scaffold; the genome has 633,737 scaffolds and is 89.3% complete.

## Burramyidae

The Burramyidae family consists of five species of pygmy possum, we investigated the mountain pygmy possum here. We located 14 MHC class II genes across 11 scaffolds in the genome assembly, a single copy of DAα and of both DB, DC and DM genes and two copies of DAβ. The DCβ gene was located across two scaffolds in the assembly. In addition. we identified five partial segments of the DAβ gene. This draft genome was 69.8% complete with 33,138 scaffolds (A. Weeks, unpublished data).

## Phalangeridae

Phalangeridae contains a number of arboreal possum-like species, the best known being the brush-tailed possum. We investigated the genomes of both the brushtail possum and the ground cuscus (O. Dudchenko et al., 2017; Olga Dudchenko et al., 2018) and identified 21 and 15 genes, respectively. In both species we located a single copy of DAα and both genes of the DC (the DCα gene in the ground cuscus is a partial sequence and the DCβ gene is split between two scaffolds) and DM molecule, we located two copies of both DB genes in the brushtail possum and two copies of DBα and a single DBβ in the ground cuscus. We identified 12 DAβ genes in the brushtail possum and seven partial DAβ genes in the ground cuscus. The possum genome is 211 scaffolds and 94.0% complete, whilst the ground cuscus is 1,143,155 scaffolds and 83.1% complete.

## Macropodidae

The Macropodidae family is one of the largest and arguable most well know family of marsupials containing a number of kangaroo and wallaby species. We investigated six Macropodidae species: eastern grey kangaroo (1,012,396 scaffolds, 88.7% complete), western grey kangaroo (1,417,922 scaffolds; 84.2%), red kangaroo (1,417,180 scaffold and 86.3%), tammar wallaby (750,707 scaffold and 84.2%), Mala (569,651 scaffold and 89.8%) and quokka (1,480,866 scaffolds and 87.7%) (O. Dudchenko et al., 2017; Olga Dudchenko et al., 2018). Across all the species investigated we located a similar number and distribution of MHC class II genes, in all species a single copy of the DAα and both DC and DM genes were located. Numbers of DAβ ranged from one (eastern grey kangaroo) to six (Mala and quokka), DBα genes ranged from one (quokka and red kangaroo) to seven (western grey kangaroo) and DBβ genes ranged from 1 (Mala and red kangaroo) to five (eastern grey kangaroo). Whilst we annotated 18 DAβ genes in the western grey kangaroo, 16 of these were partial sequences and they were located across 18 different scaffolds.

## Potoroidae

The Potoroidae show similar features to the Macropodidae however are much smaller and often nocturnal, we investigate two species the woylie (1,116 scaffolds; 94.2% complete) and Gilbert’s potoroo (1,148,657 scaffolds; 85.3% complete) in this study (O. Dudchenko et al., 2017; Olga Dudchenko et al., 2018; Peel, Silver, Brandies, Hogg, & Belov, 2021). We annotated 18 and 25 genes for Gilbert’s potoroo and the woylie, with a single copy of DAα and both DC and DM genes (the DMβ gene in Gilbert’s potoroo is split between two scaffolds) identified, however interestingly we located two copies of the DMα gene in the woylie. We annotated the highest number of DAβ genes out of any species in the woylie with 12, we also annotated two and five copies of the DBα and DBβ gene respectively. For Gilbert’s potoroo we annotated six, four and three DAβ, DBα and DBβ genes, respectively.

## Petauridae

The Petauridae family contain striped possums, the gliders and Leadbeater’s possum, which is the only species investigated here (O. Dudchenko et al., 2017; Olga Dudchenko et al., 2018). We annotated a total of 18 MHC class II genes, a single copy of DAα, DCα and both DM genes, three copies of both DB genes, eight copies of the DAβ gene and no gene was identified for the DCβ gene. This genome has 1,702 scaffolds and is 94.3% complete.

## Pseudochiridae

Pseudochiridae contain the ringtail possums, three of which we investigate here, the western ringtail possum (918,255 scaffolds; 77.3% complete), coppery ringtail possum (1,056,859 scaffold and 82.6%) and plush coated ringtail possum (552,633 scaffold and 77.9%) (O. Dudchenko et al., 2017; Olga Dudchenko et al., 2018). All species contained a single copy of the DAα and both DM genes and copies of DAβ varied from three (coppery ringtail possum) to eight (plush coated ringtail possum). For the DC molecule only partial DCα sequence could be located in the western ringtail possum genome and partial DCβ sequences in the coppery and plush coated ringtail possums and neither DB gene could be identified in any of the three assemblies.

## References

Belov, K., Deakin, J. E., Papenfuss, A. T., Baker, M. L., Melman, S. D., Siddle, H. V., . . . Miller, R. D. (2006). Reconstructing an ancestral mammalian immune supercomplex from a marsupial major histocompatibility complex. *PLoS Biol, 4*(3), e46. doi:10.1371/journal.pbio.0040046

Brandies, P. A., Tang, S., Johnson, R. S. P., Hogg, C. J., & Belov, K. (2020). The first antechinus reference genome provides a resource for investigating the genetic basis of semelparity and age-related neuropathologies. *Gigabyte, 2020*, gigabyte7. doi:10.46471/gigabyte.7

Cheng, Y., Polkinghorne, A., Gillett, A., Jones, E. A., O'Meally, D., Timms, P., & Belov, K. (2018). Characterisation of MHC class I genes in the koala. *Immunogenetics, 70*(2), 125-133. doi:10.1007/s00251-017-1018-2

Dudchenko, O., Batra, S. S., Omer, A. D., Nyquist, S. K., Hoeger, M., Durand, N. C., . . . Aiden, E. L. (2017). De novo assembly of the *Aedes aegypti* genome using Hi-C yields chromosome-length scaffolds. *Science, 356*(6333), 92-95. doi:10.1126/science.aal3327

Dudchenko, O., Shamim, M. S., Batra, S. S., Durand, N. C., Musial, N. T., Mostofa, R., . . . Aiden, E. L. (2018). The Juicebox Assembly Tools module facilitates de novo assembly of mammalian genomes with chromosome-length scaffolds for under $1000. *bioRxiv*, 254797. doi:10.1101/254797

Edwards, D., & Westerman, M. (1995). The molecular relationships of possum and glider families as revealed by DNA-DNA hybridizations. *Australian Journal of Zoology, 43*(3), 231-240. doi:10.1071/Zo9950231

Johnson, R. N., O'Meally, D., Chen, Z., Etherington, G. J., Ho, S. Y. W., Nash, W. J., . . . Belov, K. (2018). Adaptation and conservation insights from the koala genome. *Nat Genet, 50*(8), 1102-1111. doi:10.1038/s41588-018-0153-5

Kumar, S., Stecher, G., Li, M., Knyaz, C., & Tamura, K. (2018). MEGA X: Molecular Evolutionary Genetics Analysis across computing platforms. *Mol Biol Evol, 35*(6), 1547-1549. doi:10.1093/molbev/msy096

Mikkelsen, T. S., Wakefield, M. J., Aken, B., Amemiya, C. T., Chang, J. L., Duke, S., . . . Lindblad-Toh, K. (2007). Genome of the marsupial *Monodelphis domestica* reveals innovation in non-coding sequences. *Nature, 447*(7141), 167-177. doi:10.1038/nature05805

Nei, M., & Kumar, S. (2000). *Molecular evolution and phylogenetics*. New York: Oxford University.

Peel, E., Silver, L., Brandies, P., Hayakawa, T., Belov, K., & Hogg, C. J. (2022). Genome assembly of the numbat (*Myrmecobius fasciatus*), the only termitivorous marsupial. *Gigabyte, 2022*, gigabyte47. doi:10.46471/gigabyte.47

Peel, E., Silver, L., Brandies, P., Hogg, C. J., & Belov, K. (2021). A reference genome for the critically endangered woylie, Bettongia penicillata ogilbyi. *Gigabyte, 2021*, gigabyte35. doi:10.46471/gigabyte.35

Stammnitz, M. R., Gori, K., Kwon, Y. M., Harry, E., Martin, F. J., Billis, K., . . . Murchison, E. P. (2023). The evolution of two transmissible cancers in Tasmanian devils. *Science, 380*(6642), 283-293. doi:10.1126/science.abq6453

Supplementary Table 1: Order and family classification of the 29 marsupial species investigated in this study, along with name of the genome assembly file and basic genome statistics such as N50 and genome size.

| Species | Order | Family | Genus | Group Responsible | Genome Name | Sequencing Technology Used | Size (Mb) | No. Scaffolds | N50 (Mb) | N90 (Mb) | Gaps (%) | Busco v.5.3.2 |
| --- | --- | --- | --- | --- | --- | --- | --- | --- | --- | --- | --- | --- |
| Grey short tailed opossum (modo) | Didelphimorphia | Didelphidae | Monodelphis | (Dudchenko et al., 2017; Dudchenko et al., 2018; Mikkelsen et al., 2007) | MonDom5_HiC.fasta | short+scaffold | 3573.328 | 5136 | 524.29 | 437.175 | 2.063 | C:78.7%[S:73.2%,D:5.5%],F:6.3%,M:15.0%,n:9226 |
| Monito del monte (drgl) | Microbiotheria | Microbiotheriidae | Dromiciops | VGP (BioProject ID: PRJNA728141) | GCF_019393635.1_mDroGli1.pri_genomic.fasta | short+long+scaffold | 3303.2 | 17 | 670.776 | 496.997 | 0.089 | C:93.9%[S:90.2%,D:3.7%],F:1.0%,M:5.1%,n:9226 |
| Southern marsupial mole (noty) | Notoryctemorphia | Notoryctidae | Notoryctes | (Dudchenko et al., 2017; Dudchenko et al., 2018) | Notoryctes_typhlops_wtdbg2.shortReadsPolished_HiC.fasta | short+long+scaffold | 3475.141 | 10900 | 317.686 | 302.649 | 0.504 | C:87.0%[S:78.9%,D:8.1%],F:3.1%,M:9.9%,n:9226 |
| Bilby (mala) | Peramelemorphia | Thylacomyidae | Macrotis | (Hogg et al., 2023) | bilby.v1.4.4b.hiriseplus.fasta | short+long+scaffold | 3663.585 | 663 | 343.869 | 305.047 | 0.876 | C:93.3%[S:88.5%,D:4.8%],F:1.2%,M:5.5%,n:9226 |
| Eastern barred bandicoot (pegu) | Peramelemorphia | Peramelidae | Perameles | (A. Weeks, *unpublished data*) | EBB_10x-rails.scaffolds.fasta | short | 3829.861 | 170614 | 0.05527 | 0.05066 | 1.517 | C:65.2%[S:60.9%,D:4.3%],F:11.4%,M:23.4%,n:9226 |
| Numbat (myfa) | Dasyuromorphia | Myrmecobiidae | Myrmecobius | (Peel et al., 2022) | mMyrfas1.pri.20211206.fasta | short | 3424.377 | 112299 | 0.22276 | 0.13 | 3.524 | C:78.7%[S:73.2%,D:5.5%],F:6.3%,M:15.0%,n:9226 |
| Numbat DNAzoo (myfa_DNAzoo) | Dasyuromorphia | Myrmecobiidae | Myrmecobius | (Dudchenko et al., 2017; Dudchenko et al., 2018) | Myrmecobius_fasciatus_HiC.fasta | short+scaffold | 3298.104 | 1107131 | 321.463 | 0.01393 | 0.984 | C:74.7%[S:73.2%,D:1.5%],F:8.2%,M:17.1%,n:9226 |
| Tasmanian devil (saha) | Dasyuromorphia | Dasyuridae | Sarcophilus | (Stammnitz et al., 2023) | GCF_902635505.1_mSarHar1.11_genomic.fasta | short+long+scaffold | 3086.674 | 106 | 611.347 | 464.895 | 0.002 | C:91.7%[S:90.4%,D:1.3%],F:1.5%,M:6.8%,n:9226 |
| Eastern quoll (davi) | Dasyuromorphia | Dasyuridae | Dasyurus | (Dudchenko et al., 2017; Dudchenko et al., 2018) | Dasyurus_viverrinus_HiC.fasta | short+scaffold | 3085.922 | 386594 | 531.476 | 250.653 | 0.841 | C:84.6%[S:83.5%,D:1.1%],F:4.6%,M:10.8%,n:9226 |
| Northern quoll (daha) | Dasyuromorphia | Dasyuridae | Dasyurus | (Dudchenko et al., 2017; Dudchenko et al., 2018) | Dasyurus_hallucatus_10X_01_style3pseudohapfull_HiC.fasta | short+scaffold | 3148.304 | 4188623 | 605.075 | 462.366 | 1.958 | C:92.5%[S:91.0%,D:1.5%],F:1.5%,M:6.0%,n:9226 |
| Brush tailed phascogale (phta) | Dasyuromorphia | Dasyuridae | Phascogale | (Dudchenko et al., 2017; Dudchenko et al., 2018) | Phascogale_tapoatafa_HiC.fasta | short+scaffold | 3206.286 | 295136 | 577.65 | 441.184 | 0.736 | C:89.0%[S:87.2%,D:1.8%],F:3.2%,M:7.8%,n:9226 |
| Antechinus (anst) | Dasyuromorphia | Dasyuridae | Antechinus | (Brandies et al., 2020) | antechinusM_pseudohap2.1.fasta | short | 3307.513 | 30876 | 72.733 | 77.883 | 2.75 | C:92.5%[S:90.5%,D:2.0%],F:1.6%,M:5.9%,n:9226 |
| Fat tailed dunnart (smcr) | Dasyuromorphia | Dasyuridae | Sminthopsis | (Dudchenko et al., 2017; Dudchenko et al., 2018) | Sminthopsis_crassicaudata_HiC.fasta | short+scaffold | 2836.948 | 1644 | 578.922 | 423.564 | 1.245 | C:85.0%[S:83.6%,D:1.4%],F:1.0%,M:14.0%,n:9226 |
| Koala (phci) | Diprotodontia | Phascolarctidae | Phascolarctos | (Dudchenko et al., 2017; Dudchenko et al., 2018; Johnson et al., 2018) | phaCin_unsw_v4.1_HiC.fasta | short+long+scaffold | 3192.89 | 1318 | 480.108 | 413.95 | 0.01 | C:94.0%[S:92.5%,D:1.5%],F:1.2%,M:4.8%,n:9226 |
| Common wombat (vour) | Diprotodontia | Vombatidae | Vombatus | (Dudchenko et al., 2017; Dudchenko et al., 2018) | vu-2k.fasta | short+scaffold | 3341.35 | 633737 | 576.111 | 438.249 | 0.541 | C:89.3%[S:87.4%,D:1.9%],F:2.8%,M:7.9%,n:9226 |
| Mountain pygmy possum (bupa) | Diprotodontia | Burramyidae | Burramys | (A. Weeks, *unpublished data*) | MPP_2019_10x_LR_assembly.fasta | short | 2999.037 | 33138 | 0.20187 | 0.09907 | 0 | C:69.8%[S:65.8%,D:4.0%],F:4.4%,M:25.8%,n:9226 |
| Ground cuscus (phgy) | Diprotodontia | Phalangeridae | Phalanger | (Dudchenko et al., 2017; Dudchenko et al., 2018) | pg-2k.fasta | short+scaffold | 3624.08 | 1143155 | 353.052 | 333.943 | 0.926 | C:83.1%[S:79.7%,D:3.4%],F:5.1%,M:11.8%,n:9226 |
| Brushtail possum (trvu) | Diprotodontia | Phalangeridae | Trichosurus | VGP (BioProject ID: PRJNA562014) | mTriVul1.pri.cur.20200115.fasta | short+long+scaffold | 3359.331 | 211 | 442.56 | 310.106 | 0.557 | C:94.0%[S:89.8%,D:4.2%],F:1.1%,M:4.9%,n:9226 |
| Eastern grey kangaroo (magi) | Diprotodontia | Macropodidae | Macropus | (Dudchenko et al., 2017; Dudchenko et al., 2018) | mg-2k.fasta | short+scaffold | 3535.641 | 1012396 | 392.871 | 382.18 | 0.771 | C:88.7%[S:86.6%,D:2.1%],F:3.1%,M:8.2%,n:9226 |
| Red kangaroo (maru) | Diprotodontia | Macropodidae | Macropus | (Dudchenko et al., 2017; Dudchenko et al., 2018) | mr-2k.fasta | short+scaffold | 3600.371 | 1417180 | 238.281 | 138.077 | 0.774 | C:86.3%[S:84.2%,D:2.1%],F:3.9%,M:9.8%,n:9226 |
| Western grey kangaroo (mafu) | Diprotodontia | Macropodidae | Macropus | (Dudchenko et al., 2017; Dudchenko et al., 2018) | mf-2k.fasta | short+scaffold | 3638.57 | 1417922 | 341.276 | 341.276 | 0.789 | C:84.2%[S:82.2%,D:2.0%],F:4.9%,M:10.9%,n:9226 |
| Mala (lahi) | Diprotodontia | Macropodidae | Lagorchestes | (Dudchenko et al., 2017; Dudchenko et al., 2018) | Lagorchestes_hirsutus_HiC.fasta | short+scaffold | 3381.153 | 569651 | 401.334 | 271.949 | 0.744 | C:89.8%[S:87.1%,D:2.7%],F:2.8%,M:7.4%,n:9226 |
| Tammar wallaby (maeu) | Diprotodontia | Macropodidae | Macropus | (Dudchenko et al., 2017; Dudchenko et al., 2018) | me-1k.fasta | short+scaffold | 3448.232 | 750707 | 387.072 | 375.259 | 0.767 | C:84.2%[S:81.8%,D:2.4%],F:5.0%,M:10.8%,n:9226 |
| Quokka (sebr) | Diprotodontia | Macropodidae | Setonix | (Dudchenko et al., 2017; Dudchenko et al., 2018) | Setonix_brachyurus_HiC.fasta | short+scaffold | 3692.382 | 1480866 | 260.848 | 183.105 | 0.558 | C:87.7%[S:84.9%,D:2.8%],F:3.7%,M:8.6%,n:9226 |
| Woylie (bepe) | Diprotodontia | Potoroidae | Bettongia | (Peel et al., 2021) | mBetpen1.pri.20210916.fasta | short+long | 3393.487 | 1116 | 6.94 | 4.76 | 0.403 | C:94.2%[S:91.6%,D:2.6%],F:1.0%,M:4.8%,n:9226 |
| Gilbert’s potoroo (pogi) | Diprotodontia | Potoroidae | Potorous | (Dudchenko et al., 2017; Dudchenko et al., 2018) | Potorous_gilbertii_HiC.fasta | short+scaffold | 3826.526 | 1148657 | 557.805 | 266.134 | 0.668 | C:85.3%[S:74.9%,D:10.4%],F:4.1%,M:10.6%,n:9226 |
| Leadbeater’s possum (gyle) | Diprotodontia | Petauridae | Gymnobelideus | (Dudchenko et al., 2017; Dudchenko et al., 2018) | LBP_v1_HiC.fasta | short+scaffold | 3475.047 | 1702 | 317.957 | 280.492 | 0.166 | C:94.3%[S:86.0%,D:8.3%],F:1.2%,M:4.5%,n:9226 |
| Plush coated ringtail possum (psco) | Diprotodontia | Pseudocheiridae | Pseudochirops | (Dudchenko et al., 2017; Dudchenko et al., 2018) | Pseudochirops_corinnae_HiC.fasta | short+scaffold | 3503.581 | 552633 | 209.248 | 203.747 | 0.964 | C:77.9%[S:73.3%,D:4.6%],F:6.6%,M:15.5%,n:9226 |
| Western ringtail possum (psoc) | Diprotodontia | Pseudocheiridae | Pseudocheirus | (Dudchenko et al., 2017; Dudchenko et al., 2018) | Pseudocheirus_occidentalis_HiC.fasta | short+scaffold | 3603.847 | 918255 | 213.462 | 208.556 | 0.953 | C:77.3%[S:72.3%,D:5.0%],F:6.9%,M:15.8%,n:9226 |
| Coppery ringtail possum (pscu) | Diprotodontia | Pseudocheiridae | Pseudochirops | (Dudchenko et al., 2017; Dudchenko et al., 2018) | Pseudochirops_cupreus_HiC.fasta | short+scaffold | 3579.984 | 1056859 | 425.238 | 425.238 | 0.932 | C:82.6%[S:79.5%,D:3.1%],F:5.4%,M:12.0%,n:9226 |

Supplementary Table 3: Coefficients and standard errors for genome quality metrics (N50 and BUSCO completeness) as predictors for number of loci for each class II gene (*DAB, DBA, DBB, DCA*, and *DCB*) assessed through a phylogenetic least squares (PGLS) model. Significant predictors are highlighted in bold.

|  | Predictors | Estimate | Std Err | F | df | P |
| --- | --- | --- | --- | --- | --- | --- |
| DAB | Intercept | -11.635 | 8.663 | 1.804 | 1 | 0.191 |
|  | **Busco completeness** | **-0.009** | **0.004** | **5.696** | **1** | **0.025** |
|  | **N50** | **24.09** | **10.68** | **5.088** | **1** | **0.033** |
| DBA | Intercept | -1.654 | 4.04 | 0.171 | 1 | 0.683 |
|  | Busco completeness | 0.001 | 0.002 | 0.357 | 1 | 0.555 |
|  | N50 | 3.183 | 4.91 | 0.431 | 1 | 0.522 |
| DBB | Intercept | -4.727 | 2.901 | 2.655 | 1 | 0.115 |
|  | Busco completeness | -0.001 | 0.001 | 0.631 | 1 | 0.434 |
|  | N50 | 7.745 | 3.567 | 4.714 | 1 | 0.4 |
| DCA | Intercept | 0.712 | 0.582 | 1.495 | 1 | 0.233 |
|  | Busco completeness | -0.0001 | 0.0002 | 0.33 | 1 | 0.57 |
|  | N50 | 0.066 | 0.671 | 0.01 | 1 | 0.923 |
| DCB | Intercept | 0.627 | 0.621 | 1.02 | 1 | 0.322 |
|  | Busco completeness | 0.0002 | 0.0002 | 0.488 | 1 | 0.491 |
|  | N50 | -0.158 | 0.715 | 0.05 | 1 | 0.826 |

Supplementary Table 4: Results of phylogenetic correlation analysis, displayed are Moran’s I values and P values for correlation at genus, family and order levels for each gene

|  | Taxonomic Level | Moran I | P value |
| --- | --- | --- | --- |
| DAB | Genus | -0.074 | 0.918 |
|  | Family | 0.012 | 0.819 |
|  | Order | 0.058 | 0.462 |
| DBA | Genus | 0.331 | 0.327 |
|  | **Family** | **0.389** | **0.043** |
|  | Order | 0.151 | 0.139 |
| DBB | Genus | 0.216 | 0.512 |
|  | **Family** | **0.443** | **0.026** |
|  | Order | 0.111 | 0.256 |
| DCA | Genus | 0.586 | 0.120 |
|  | **Family** | **0.558** | **0.008** |
|  | **Order** | **0.619** | **1.11 X10-6** |
| DCB | Genus | 0.502 | 0.180 |
|  | **Family** | **0.545** | **0.010** |
|  | **Order** | **0.654** | **3.29 X10 -7** |

Supplementary Table 5: Relative goodness of fit for three evolutionary models for class II gene (*DAB, DBA, DBB, DCA*, and *DCB*)

|  | Evolution Model | AIC_C_ | ∆AIC_C_ | Relative Importance (ꞷ_i_) |
| --- | --- | --- | --- | --- |
| DAB | **Brownian Motion adjusted for λ** | **169.557** | **0** | **1** |
|  | Brownian Motion | 226.905 | 57.348 | <0.001 |
|  | Ornstein-Uhlenbeck | 225.845 | 56.288 | <0.001 |
| DBA | **Brownian Motion adjusted for λ** | **127.018** | **0** | **0.998** |
|  | Brownian Motion | 136.764 | 13.746 | 0.001 |
|  | Ornstein-Uhlenbeck | 136.355 | 13.337 | 0.001 |
| DBB | **Brownian Motion adjusted for λ** | **107.217** | **0** | **0.999** |
|  | Brownian Motion | 136.153 | 28.936 | <0.001 |
|  | Ornstein-Uhlenbeck | 135.537 | 28.320 | <0.001 |
| DCA | Brownian Motion adjusted for λ | 10.755 | 2.498 | 0.182 |
|  | **Brownian Motion** | **8.256** | **0** | **0.635** |
|  | Ornstein-Uhlenbeck | 10.750 | 2.493 | 0.183 |
| DCB | Brownian Motion adjusted for λ | 14.038 | 2.498 | 0.182 |
|  | **Brownian Motion** | **11.539** | **0** | **0.634** |
|  | Ornstein-Uhlenbeck | 14.012 | 2.473 | 0.184 |

Supplementary Table 6: Coefficients and standard errors for life history metrics (Ratio of average offspring to number of teats and residual lifespan as predictors for number of loci for each class II gene (*DAB, DBA, DBB, DCA*, and *DCB*) assessed through a phylogenetic least squares (PGLS) model. Significant predictors are highlighted in bold.

|  | Predictors | Estimate | Std Err | F | df | P |
| --- | --- | --- | --- | --- | --- | --- |
| DAB | Intercept | 7.715 | 4.818 | 2.564 | 1 | 0.121 |
|  | Residual Lifespan | 1.930 | 4.353 | 0.196 | 1 | 0.661 |
|  | Ratio of Offspring/Teat Number | -1.604 | 4.045 | 0.157 | 1 | 0.695 |
| DBA | Intercept | 2.454 | 1.970 | 1.564 | 1 | 0.222 |
|  | Residual Lifespan | -0.754 | 1.772 | 0.181 | 1 | 0.634 |
|  | Ratio of Offspring/Teat Number | -2.806 | 1.726 | 2.642 | 1 | 0.116 |
| DBB | Intercept | 3.562 | 1.511 | 5.555 |  | 0.026 |
|  | Residual Lifespan | 0.972 | 1.362 | 0.509 | 1 | 0.482 |
|  | **Ratio of Offspring/Teat Number** | **-2.67** | **1.304** | **4.27** | 1 | **0.049** |
| DCA | Intercept | 1.060 | 0.337 | 9.881 | **1** | 0.004 |
|  | Residual Lifespan | 0.132 | 0.270 | 0.237 | 1 | 0.63 |
|  | Ratio of Offspring/Teat Number | -0.498 | 0.309 | 2.586 | 1 | 0.12 |
| DCB | Intercept | 0.258 | 0.372 | 0.482 | 1 | 0.494 |
|  | Residual Lifespan | -0.288 | 0.298 | 0.935 | 1 | 0.343 |
|  | Ratio of Offspring/Teat Number | 0.244 | 0.341 | 0.512 | 1 | 0.481 |

Supplementary Table 7: Coefficient estimates and 95% HPD CI intervals for life history metrics (Ratio of average offspring to number of teats and residual lifespan as predictors for number of loci for each class II gene (*DAB, DBA, DBB, DCA*, and *DCB*) assessed through Bayesian Phylogenetic Mixed Models (BPMM)

|  |  | Estimate | Lower 95% HPD CI | Upper 95% HPD CI |
| --- | --- | --- | --- | --- |
| DAB | Intercept | 5.655 | 2.316 | 9.241 |
|  | Ratio of Offspring/Teat Number | -0.182 | -6.101 | 5.386 |
|  | Residual Lifespan | **8.597** | **0.456** | **16.961** |
| DBA | Intercept | 3.490 | 1.865 | 5.175 |
|  | Ratio of Offspring/Teat Number | **-3.645** | **-6.606** | **-1.143** |
|  | Residual Lifespan | 1.153 | -2.489 | 4.590 |
| DBB | Intercept | 2.624 | 1.271 | 3.857 |
|  | Ratio of Offspring/Teat Number | **-2.789** | **-4.329** | **-0.074** |
|  | Residual Lifespan | 0.542 | -2.320 | 3.342 |
| DCA | Intercept | 1.040 | 0.562 | 1.565 |
|  | Ratio of Offspring/Teat Number | -0.530 | -1.144 | 0.024 |
|  | Residual Lifespan | 0.474 | -0.228 | 1.179 |
| DCB | Intercept | 0.635 | 0.051 | 1.201 |
|  | Ratio of Offspring/Teat Number | 0.010 | -0.559 | 0.727 |
|  | Residual Lifespan | 0.096 | -0.734 | 0.941 |


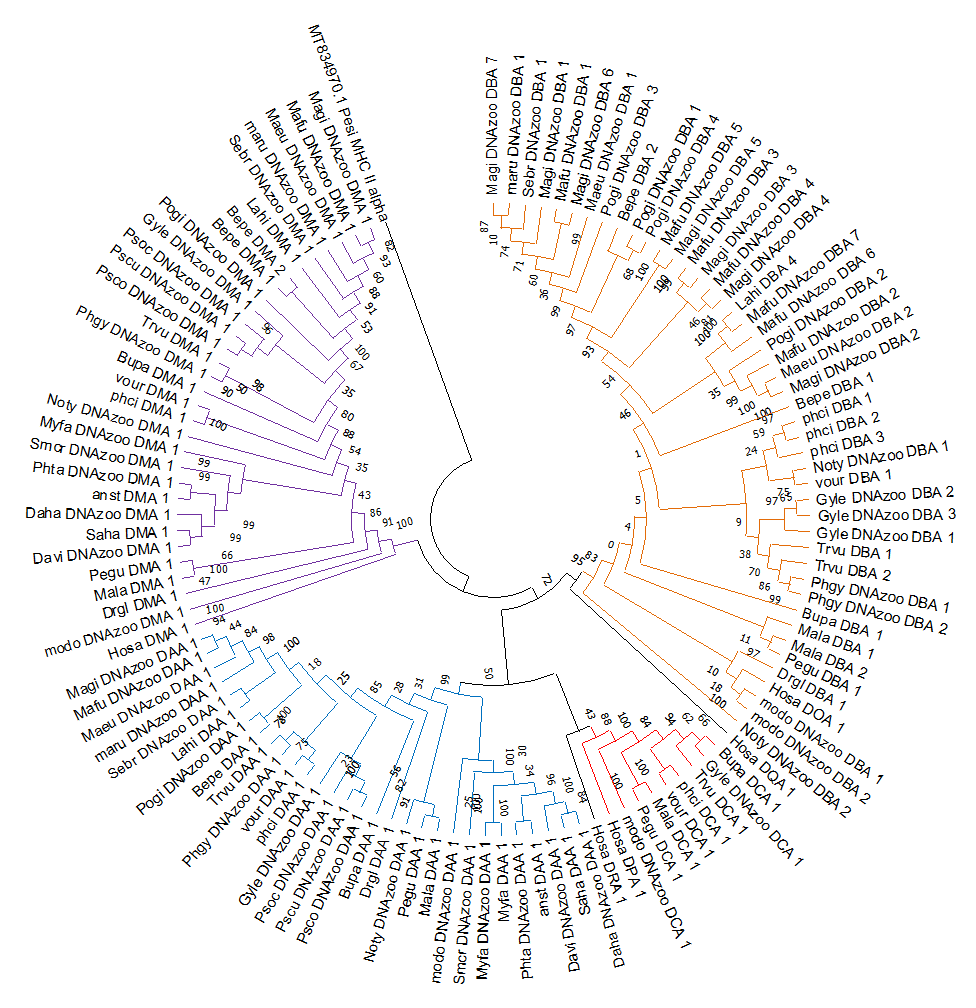


Supplementary Figure 1: Phylogenetic arrangement of marsupial MHC class II alpha genes. Phylogenetic tree was constructed in MEGAX using a maximum likelihood method using General Time Reversible model with a Gamma distribution and rooted using koala class I genes (Cheng et al., 2018; Kumar, Stecher, Li, Knyaz, & Tamura, 2018; Nei & Kumar, 2000). Prefixes of gene names are first two letters of the genus and first two letters of the species (see methods for all species abbreviations). *DAA* genes are coloured blue, *DMA* genes are coloured purple, *DCA* genes are coloured red and *DBA* genes are coloured orange. Node labels are bootstrap percentages


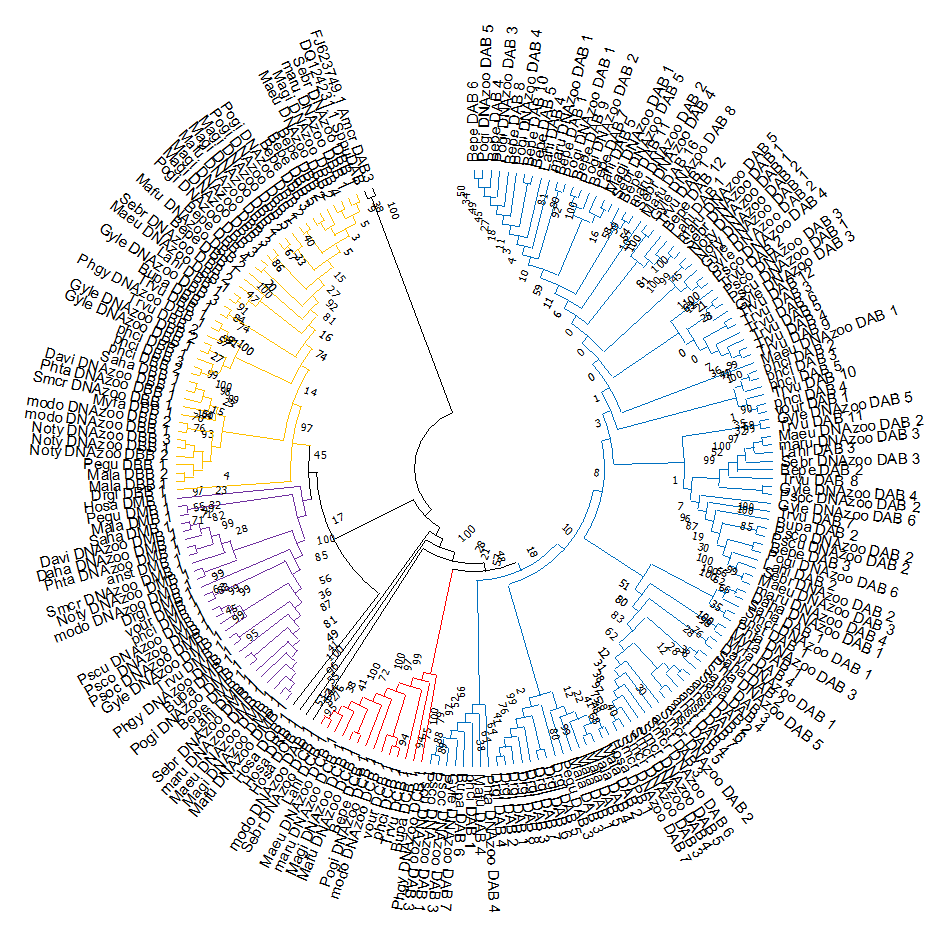


Supplementary Figure 2: Phylogenetic arrangement of marsupial MHC class II beta genes. Phylogenetic tree was constructed in MEGAX using a maximum likelihood method with General Time Reversible model with a Gamma distribution and rooted using koala class I genes (Cheng et al., 2018; Kumar et al., 2018; Nei & Kumar, 2000). Prefixes of gene names are first two letters of the genus and first two letters of the species (see methods for all species abbreviations). *DAB* genes are coloured blue, *DM****B*** genes are coloured purple, *DCB* genes are coloured red and *DBB* genes are coloured orange. Node labels are bootstrap percentages


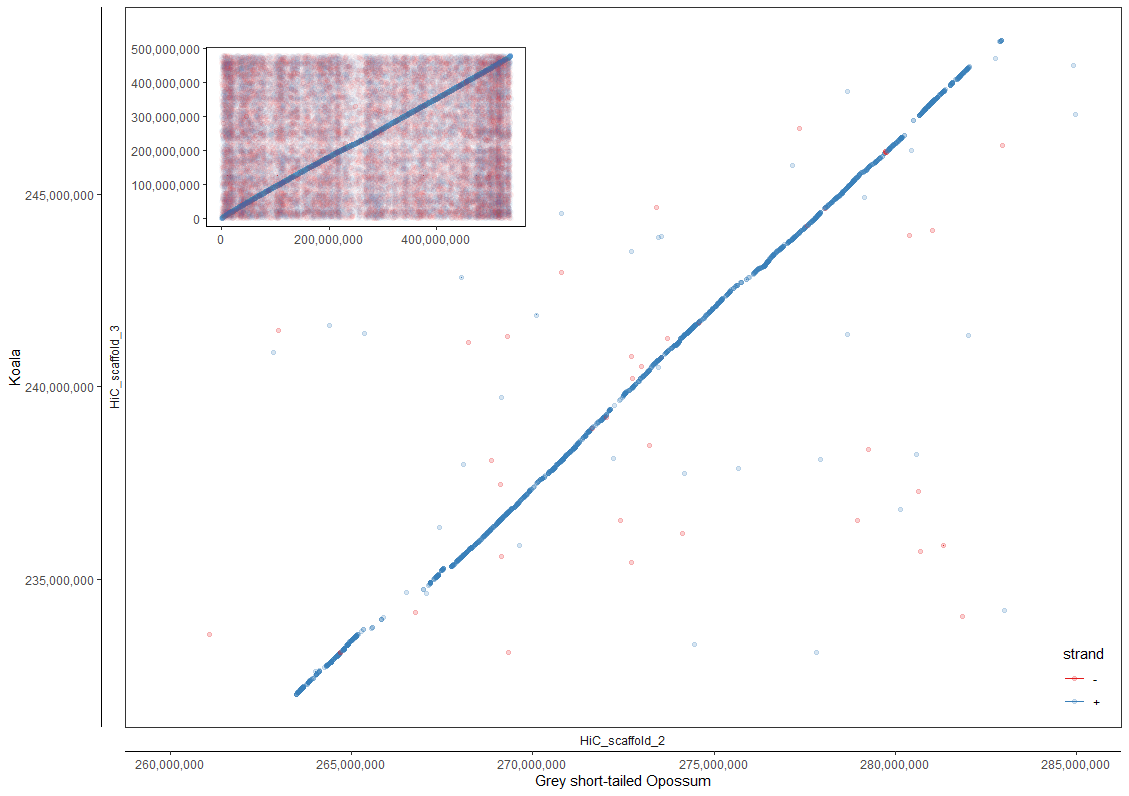


Supplementary Figure 3: Synteny dot plot of the MHC region on scaffold 2 of the grey short-tailed opossum and on scaffold 3 of the koala, showing the same orientation of the genes within the class II MHC region. Inset plot is a synteny dot plot of the entire of scaffold 2 and scaffold 3 in the grey short-tailed opossum and koala, respectively


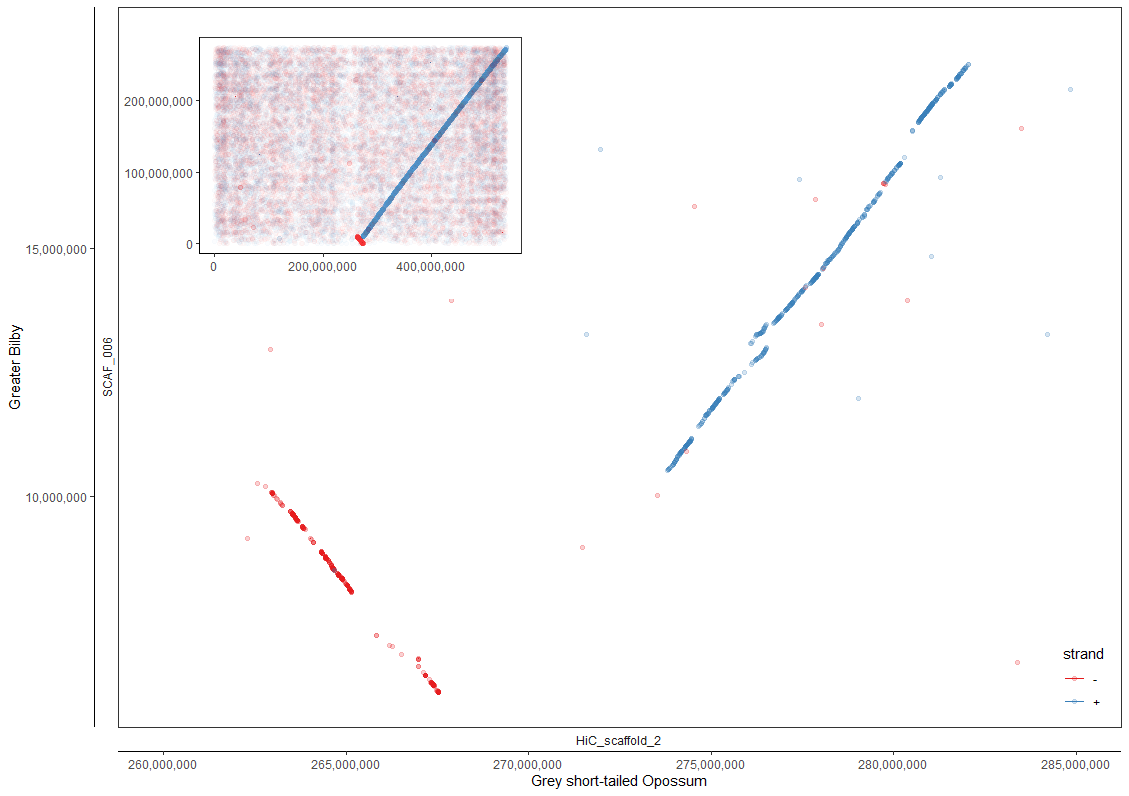


Supplementary Figure 4: Synteny dot plot of the MHC region on scaffold 2 of the grey short-tailed opossum and on scaffold 6 of the bilby, showing a portion of class II MHC region has been inverted in the bilby genome. Inset plot is a synteny dot plot of the entire of scaffold 2 and scaffold 6 in the grey short-tailed opossum and bilby, respectively


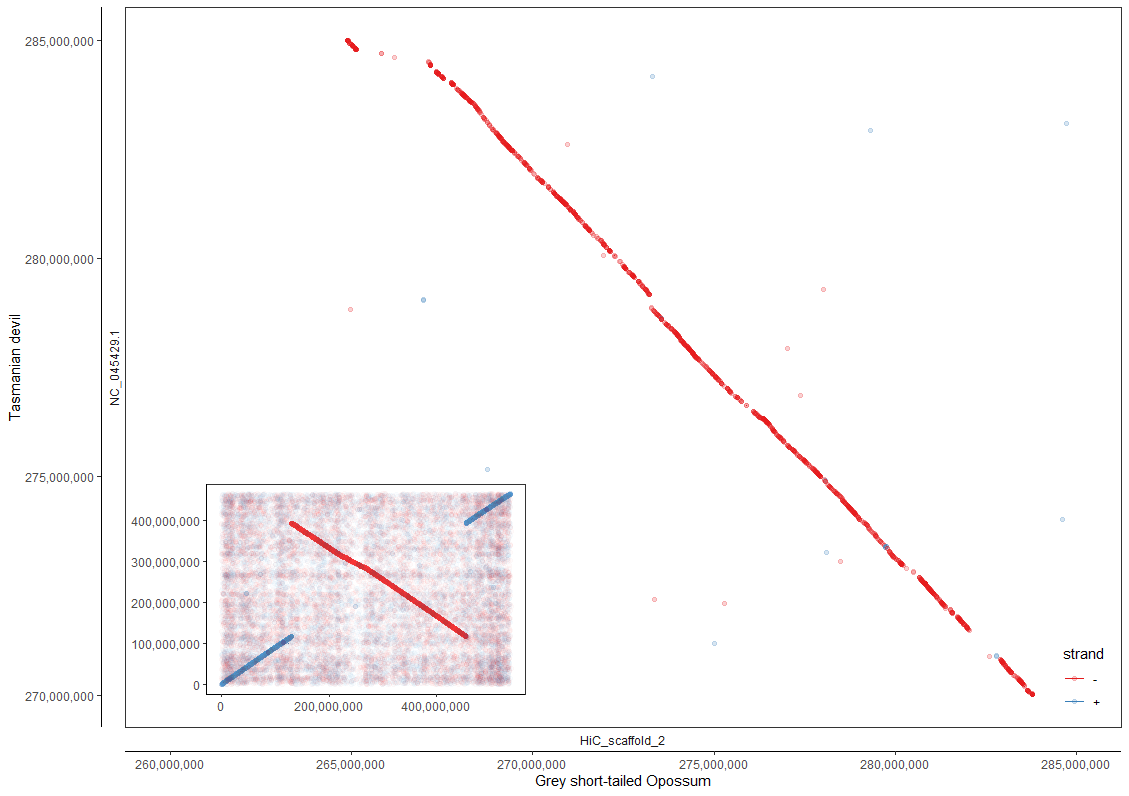


Supplementary Figure 5: Synteny dot plot of the MHC region on scaffold 2 of the grey short-tailed opossum and on scaffold NC_045429 of the Tasmanian devil, showing the entire MHC class II region has been inverted in the Tasmanian devil. The inset plot is a synteny dot plot of the entire of scaffold 2 and scaffold NC_045429 of the grey short-tailed opossum and Tasmanian devil, respectively

Additional Data 1

>anst_DAA_1

ATGATCCCCAACAAAGCTTTGATCCTAGGGGCTTTCACCCTGGCTGTACTGCTGAATCCCTGGGGAGCCAGAGCCATTAAAGAGAATCATGTGATTATCCAAGCTGAGTTCTCCCAGACCCACAGCCCTTTAGGAGAGTTCATGTTTGATTTTGATGGCGATGAAATTTTCCATGTGGATTTGGACAAGAAAGAGACAGTCTGGCGTCTTCCTGACTTCAGCAAATTTGCCAGCTTTGAGGCTCAGGGTGCTCTGGCCAATCTTGCTGTGGACAAAGCCAATCTGGACATCATGATGAAAAGGTCCAACTACACTCCTGATACCAATGTGCCCCCTGAAGTCACAGTGTTTCCTCAGAGCCCAGCGGAGCTGGACGAGCCCAATGTCCTTATCTGCTTCATTGACAAGTTCTCTCCCCCGGTACTTAATGTGACATGGCTTCGTAATGGGCAGCCTGTCACTGAGGGTGTGTTTGAGACTGTCTTCCTCCCCCGCCCTGATCATGCCTTCAGAAAATTCCACTACCTCACCTTCATCCCCTCTGCCAATGATTATTATGACTGCAAGGTCGATCACTGGGGACTGGACCAACCTGTTTTGAAACATTGGGAACCAGAAATACGAACCCCACTGCCAGAGACAACAGAGACTGTGGTCTGTGCCCTGGGCCTAGCCATAGGCCTCGTGGGCATCGTTGCAGGCACCATCCTGATTATCAAGGGCATGAAAGCAAACAACACTTCCCGTGGTGGCCCTCGTGGACCCCTGTAA

>anst_DMA_1

ATGGGACTTGAGCAAAACCTGGGAGTTACACTGTCACTACTGAAGCTACAGTCATCATTGCTTTTGTCCCTGTTCTGGGAAGCCACTCCAGTGTTGGCAGTGTTTGGGAATAATTTACAGAATTACACATTCTCTCACACACTATTCTGCCAGAATGGAGAATCCTCCTTGGGTCTATCAGAAAACTTCAATGGGGACCATCTCTTCTCCTTTGACTTCTCCAAGAACTCCCGGGTGCCCCGGCTGCCTGAATTTGCTGCTTGGGCCACTGATAAAGGAGACATCGAGAGCATAGATGCTGACAAGAATCTCTGCCAGGAGCTGCAACATAAATTGAGTAAACTTTGTAAAGGAAAGATCCCTGAGGCTAGAGGAATCCCCGTGGCTGAAGTTTTCACTCTGGAGCCTCTGGAGTTTGGGAAGCCCAACACTCTCGTTTGTTTTGTTAGCAATTTCTTCCCACCTCGTATAACCGTGACCTGGCAACATGAAGGAGTCTCTGTGGAAAGCAGCAGCCCCACCTTTCTCTCAGCTACAGATGGACTTAACTTCCAGGCCTTTTCTTATCTGAACATCACACCCAAATCCACTGATGTTTTCTCTTGCATTGTGGCACAGGAAGGTGACCTCTTCAGCACTATAGCTTTTTGGGTGCCTCAGAATCCAATACCCTCTGCATTGTTGGAAAACATACTGTGTGGTATTGCCTTTGGCTTGGGCATTGTTGGTATCATCGTAGGTGCTTCCCTCATCATCTACTTCCAAAAGCCATGTGTAAATGGTGCAGGTAA

>Mala_DAA_1

ATGGCCCCCAACAAAGCCTTGATCCTAAGTGTCTTCACTCTGACAATGCTACTGAATCCCTGGAGAGTCAGGGCGATTAAAGAGAAACATGTGGTCATCCAAGCAGAGTTCTACCAGACTCATGAACCCACGGGAGAATTCATGTTTGACTTTGATGGGGATGAGATTTTCCATGTGGATTTGCAAAAGAAAGAGACAGTTTGGAGACTTTCAGACTTCAGCAAATTTGCCAGTTTTGAGGCTCAGGGTGCTCTGGCCAATCTTGCTGTGGACAAAGCCAACCTGGAAATCATGATGAAACGGTCCAACAACACCCCTGACACCAATGTGCCCCCTGAAGTGACAGTGTTTCCCAAAGGCCCAGTGGAGCTGGGTCAGCCCAATGTTCTCATCTGCTTCATTGACAAGTTCTCTCCTCCTGTACTTAATGTGACATGGCTTCATAACAAACGTCCTATCACTGATGGTGTATTTGAGACTGTCTTCCTCCCTCGCTCTGACCATTCCTTCAGAAAATTCCACTATCTCACCTTTATCCCCTCTGCTGACGATTTCTATGACTGTAAAGTTGATCACTGGGGACTGGAAGAACCTCACCAGAAATACGGACTCCACTGCCAGAGACAACAGAAACTGTGGTCTGTGCCCTGGGCTTGGCCATAGGCCTGGTGGGCATCATCATAGGCACCATCCTGATTTTCAGGGGTATGCGAACAAGCAACAATTCCCGAGGAGGCCCCCGAGGACCCCTGTAA

>Mala_DBA_1

AACCCCAGGTTCTATTCCCACTACCAGAGACAACAGAAACTGTGGTCTGTGCCCTTGGTCTGACTGTAGGCCTGGTGGGCATCATTATGGGCACCATCCTCATTATCAAAGGCATATGCTCCAGCAGTAGGATACAACACCAAGGACCTCTGTGATGCCCCCTGAGGTGACTGTGTTCTCAGAGAGCCCCGTGGAGGTGGGCCAGCCAAATATCCTTATCTGCTTGGTGGACAACATCTTCCCTCCAGTGGTCAACATCACATGGCTTCGTAATGGGCAGTTGGTCACCACAGGTGTGTCTGAGACAGACTTCTACCCTCGGCCTAACCACAGATTCTGCAAGTTCTATTATCTCACTTTTCTTCCCAGTGCAGAAGATTTTTATGACTGCAAAGTGGAGCACTGGGGCCTGGAGCAGCCATTCCTCAAGCATTGGGATGACCATATGGGGACCTATGGCACAACTCTGTACCAGACCTATGAAGCCATAGGCCAGTTTACAGAAGAATTTGATGAGGATGAGCTCTTTTATGTGGACCTGCAGAAGAAGGACACTGTCTGGCGACTGCCAGAATTTAATCATTTTAGTACCTTTGACCCTCAGGGAGGGCTGAGAAGCATAGCCATAATGAAAGAAAACCTGGACAGTCTCATCAAACGCTTCAACATAACAGGGGCAATCAGTGATGGCCATCATCAGAGTCTGGATGCTAGGGATCCTCTCACTGACCCTGCTTCTGAGTTCCCGAGGAGCTTCTGAGTCCACTGAAG

>Mala_DBA_2

ATGGCCATCAACAGAGTCCTGATCCTAGGGACCCTCTCACTGGCCGTGCTGCTGAGTCCCCCAGGAGCTTGCGAGTCCATTGAAGCTGACCATATAGGAATCTATGGCACAACTCTGTACCAGGATTATGGGCCCTCAGGCCAGTATACACAAGAGTTTGATGGTGATGAGCTCTTTTATGTGGACCTACAGAAGAAAGAGACTGTATGGCGACTTGAAGAGTTTAGCCATTTTAGTAGCTTTGATCCTCAGGGAGGGTTGACAAACATAGCCACAGCAAAGCACAACCTGGACATCTTGATGAAACGCTCCAACAGAACCAGGGCCCTCATGCCCCCCGAGGTAACTGTGTTCTCAGAGAGCCCTGTAGAGGTGGACCAGCCAAATGTCCTCATCTGCTTGGTGGACAACATCTTTCCCCCAGTGGTCAACATCACATGGCTTCGTAATGGGCAGTTGGTCACCACAGGTGTGTCTGAGACAGACTTCTACACTCGGCCTGACAACAGATTCCGCAAGTTCTACTACCTTACTTTTCTCCCCAACACAGAAGATATCTATGACTGCAAAGTGGAGCATTGGGGCCTGGAGCAGCCAATCCTCAAGCACTGGGAACCCCAGGTTCCATCCCCACTGCCGGAGACAACAGAAACAGTGGTCTGTGCCCTTGGTCTGGCTGTAGGCCTGGTGGGCATCATTGTGGGCACCATCCTCATTATAAGAAACATGCGTTCTAGCAGTAGGATCCAACATCAAGGGCCTCTGTGA

>Mala_DCA_1

ATGAATCTTAGTCTAGCCTCAGGCTTAGGGATCCTCAGCCTGGCTGCCCGGCTGATCAGACTAAGAGCCTGGGCTGCTAACAACCCTGTAATCAAATCAACATTTGTTCAGACCCACAGACTTGCATGAATTTGATGAAGTTGAGTAATTCCACATGGACCTTGAGAGGAAGAAAACAGTAAGGAAGCTTCCTGAGTTTGGCCACATCTTTAGTTTTGATGCCCAGATTGGGCTAGGCTACTACTGTGGACTTGAACGACCTTATCAGGCAAAACATCCACACCTGGGCCACCCTTATGCCTTTGGAGGTGACAGTATTTCCTAAGGAGCCCATACAACTAGAGGAGCCAAACACCCTCTGCCACACTGATATGTTTCCCCCCACAGTGATTAATGTCACATGGTTGTGCAATGGACAGTCAGTCACTATAGGGATATCTGAGACCCCTTTCCTCCTTCACAATGACTATTCTTTTCCACAGGTTTCAGTTCCTCACTTTCCTTCCCTCAACTGATGATGTCTATGACTGCCTGGTTGAACACTAGGACCTGGAGAAGTCACTTTTCAGGCACTGAGCCTGAGATGCTAACACACCATCTGAGACAATGGAGACCCTCATCTTTATTCTAGGACTGGCTGTGGGCCTGGGGGGCATCACTGTGGCTACCATCCTCATCATCAAAATCTTGTTCTCCGGCAAATGA

>Mala_DMA_1

ATGGGACCTGAGCAAAGCCTGGGAGCTACACTGTTACTACTGCAACTTCACTCCTCATTGCTTTTGTCCCTGTCCTTGGGAGACACTCCAGAATTTACATCATTACTAGAGAACAGCCTGCAGAACTACACATTCTCTCACACGATATTCTGCCAGAATGCGGAACCCTTTGTGGGTCTGTCTGAGAGCTTCAATGGGGACCAGCTCTTCTCCTTTGACTTCCCCAGTAACTCCCGGGTGCCCCGACTCCCCGAGTTTGCTGCTTGGGCCAGTGATGAGGGAGATGTCCAGGCCATAGATGCTGAAAAAAATCTCTGCCAGAATCTGCAAAATGCATTCAGCCAAGCTTGTCAAGGCCATATCCCTGAGTCTAGAGGAAACCCTGTGGCTGAAGTTTTCACTCTGGAGCCCCTGGAGTTTGGAAAGCCCAACACTCTCATCTGCTTTGTTAGTAACCTCTTCCCACCTCAAGTATATGTGAAATGGCAGCACAATGGAGTCCCTATAGAAAGCAACAGCCCCATTTTTCTCTCAGCTATAGATGGACTTGGCTTCCAGGCCTTCTCTTACCTGAACTTCACACCTACATCTTCGGATATCTTTACTTGCGTTGTGGAACGGGATGGTGACCTCTTCAGTACTATAGCATTCTGGGTGCCTCAGAATCCAATACCTTCTGCATTGTTGGAAAACATACTGTGTGGTATTGCCTTTGGCCTGGGCATTGTTGGCATCATAGTGGGTGCTGCCCTCATCATCTGCTTCCAAAAACCATGTGCAAGTGGTGCAGGTAA

>Phta_DNAzoo_DAA_1

ATGATCCCCAACAAAGCATTGATCCTAGGGGCTTTCACCCTGGCCGTGCTGCTGAATCCCTGGGGAGCCAGAGCTATTAAAGAGAATCATGTGATCATCCAAGCTGAGTTCTACCAGACCCACAGCCCTTTAGGAGAGTTCATGTTTGATTTTGATGGCGATGAAATTTTCCATGTGGATTTGGACAAGAGAGAGACAGTCTGGCGTCTTCCTGACTTCAGCAAATTTGCCAGCTTTGAGGCTCAGGGTGCTCTGGCCAATCTTGCTGTGGACAAAGCCAATCTGGACATCATGATGAAAAGGTCCAACTACACTCCTGATACCAATGTGCCCCCTGAAGTGACAGTGTTTCCTCAGAGCCCAGCGGAGCTGGATGAGCCCAATGTCCTTATCTGCTTCATTGACAAGTTCTCTCCTCCGGTACTTAACGTGACATGGCTTCGTAATGGTCATCCCATCACTGAGGGTGTGTTTGAGACTGTCTTCCTCCCCCGGCCTGATCATGCCTTCAGAAAATTCCACTACCTCACCTTCATCCCCTCTGCCAATGATTACTATGACTGTAAGGTCGATCACTGGGGACTGGACCAACCTGTATTGAAACATTGGGAACCAGAAATACGAACCCCACTGCCAGAGACAACAGAGACTGTGGTCTGTGCCCTGGGCCTAGCCATAGGCCTCGTGGGCATCGTTGCAGGCACCATCCTGATTATCAAGGGCATGAAAGCAAACAATACTTCCCGTGGTGGCCCTCGTGGACCCCTGTAA

>Phta_DNAzoo_DMA_1

ATGGGACTTGAGCAAAACCTGGGAGTTACACTGTCACTACTGAAGCTACAGTCATTATTGCTGTTGTCCCTGTTCTGGGAAGCCACTCCAGTGTTGGCAGTGTTTGGGAATAATTTACAGAATTACACGTTCTCCCGCACACTCTTCTGCCAGAATGGAGAATCCTCCTTGGGTCTATCAGAGAACTTCAATGGGGACTATCTCTTCTCCTTTGACTTCTCCAAGAACTCCCGGATGCCCCGGCTGCCTGAATTTGCTGCTTGGGCCACTGATAAAGAAGACATCGAGAGCATAGATGCTGACAAGAATCTCTGCCAGGAGCTGCAACACAAATTGAGTAAACTTTGTAAAGGAAAGATCCCTGAGGCTAGAGGAATCCCTGTGGCTGAAGTTTTCACTCTGGAGCCCCTGGAGTTTGGGAAGCCCAACACTCTCGTTTGTTTTGTTAGCAATTTCTTCCCACCTCGTATAACCGTGACCTGGCAACATGAAGGAGTCTCTGTGGAAAGCAGCAGCCCCACCTTTCTCTCAGCTACAGATGGCCTTAACTTCCAGGCCTTTTCTTATCTGAACATCACACCCAAATCCACTGATGTTTTCTCTTGTACTGTGGCACAGGAAGGTGACCTCTTCAGCACTATAGCTTTCTGGGTGCCTCAGAATCCAATACCCTCTCCATTGTTGGAAAACATACTGTGTGGTATTGCCTTTAGCCTGGGCATTGTTGGTATCATCGTAGGTGCTTCCCTCATCATCTACTTCCAAAAACCATGTGTAAATGGTGCAGGTGA

>Trvu_DAA_1

ATGGACCCCAACAAAGCCTTCATCTTAGGAGTCTTCAACCTAGCAGTGCTGCTGAGTCCCTGGGGAGCCAGGGCCATTAAAGAGAATCATGTGATCATCCAAGCAGAGTTCTACCAGACCCACAACCCCTCGGGCGAGTTCATGTTTGACTTTGATGGAGATGAGATTTTCCATGTGGATTTGGAGAACAAGCAGACAGTCTGGCGGCTTCCTGACTTCAGCAAATTTGCCAGCTTTGAGGCTCAGGGTGCTCTGGCCAATCTTGCTGTGGATAAAGCCAACCTGGAAATCATGATGAAACGGTCCAACAATACCCCTGACACCAATGTGGCCCCTGAAGTGACAGTGTTTCCAAAGGGCCCAGTGGAGCTGGGCCAGCCCAATGTCCTTGTCTGCTTCATTGACAAGTTCTCTCCTCCGGTACTTAACGTGACGTGGCTTCATAATGGGAATCCCATCACTGATGGTGTGTTTGAGACTGTCTTCCTCCCCCGTTCTGACCATGCCTTCAGAAAGTTCCACTATCTCACCTTCATCCCCTCTGCCACCGATTACTATGACTGCAAGGTCGAACACTGGGGACTGGAACAACCTGCTGTCAAACACTGGGAACCAGAAGTACGGACCCCACTGCCAGAGACAACAGAGACTGTGGTCTGTGCCCTAGGCCTGGCCATAGGCCTGGTGGGCATCATCGTAGGCACCACCCTTATTGTCAAGGGCATGCGATCAAGCAATACTTCCCGTGGCGGCTCCCGTGGACTGTAA

>Trvu_DBA_1

ATGACTGCCAACAGAGTCCTGATCCTAGGGACCCTCTCACTGGCTGTGCTGCTGAGTCCCCAAGGAGCTTCTGAGTCCATTGAAGCTGACCATGTGGGAACCTATGGCACAACTATGTACCAGTCCTATGGACCCTCAGGCCAGTACACACAAGAATTTGATGAAGATGAGATCTTTTATGTGGACCTGCAGAAGAAGGAGACTGTGTGGCGGCTCCCAGAGTTCAGCCATTTTACTAGCTTTGACTCCCAGGGAGGGCTGACAAACATAGCCACAGCCAAACACAACCTGGATGTCATGATCAAAGACTCCAACGGAAGCAGGGCCATCAGTGTGCCCCCTGAGGTGACCGTGTTCTCAGAGAGTCCTGTGGAGATGGGCGAGCCGAACGTACTCATCTGCTTGGTGGACAACATCTTCCCCCCAGTCGTCAACATCAAGTGGCTTCGTAATGGGCAGTTGGTCACTGAAGGTGTGTCTGAGACAGACTTCTACCCCCGACCTGACCACAGATTCCGCAAGTTCTACTACCTCACTTTTCTCCCCAACACAGAAGACTTTTATGACTGCAAAGTGGAGCACTGGGGCCTGGAGCAGCCAGTCCTCAAGCACTGGGAACCGCAGGTTCCATCCCCACTGCCAGAGACAGCAGAAACTGTGGTCTGTGCCCTTGGTCTGGCTGTGGGCCTGGTGGGCATCATTGTGGGTACCGTCCTCATAATCAGAGGCATGCGTTCCAGCAGTAGGATCCAACATCAAGGGCCTCTGTGA

>Trvu_DBA_2

ATGGCTGCCGACAGAGTCCTGATCCTAGGGACCCTCTCACTGGCTGTGCTGCTGAGTCCCCAAGGAGCTTCTGAGTTCATTGAAGCTGAACATGTTGGGGTCTACGGCACAACTTTTTGCCAGTCCTATGGGCCCTCAGGCCAGTTTACACAAGAATTTGATGAAGATGAACTCTTTTATGTGGACCTGGAGAAGAAGGAGACTGTGTGGCGGCTCCCAGAGTTCAGCCATTTTGCTAGCTTTGACTCTCAGGGAGGGCTGACAAACATAGCCATGTGCAAGCGCCACCTGGAAATCTCAATCAAACGCTACAACAGAAGCAGGATCATCGCCGTGCCCCCTGAGGTGACTGTGTTCTCGGAGAGTCCCGTGGAGATGGGCCAGCCAAACATACTCATCTGCTTGGTGGACAACATCTTCCCCCCAGTGGTCAACATCAAGTGGCTTCGTAATGGGCAGTTGGTCACTGAAGGTGTGTCTGAGACAGACTTTTACCCTCGGCCTGACCACAAATTCCGCAAGTTCTACTACCTCACTTTTCTCCCCAACACAGAAGACTTTTATGACTGCAAAGTGGAGCACTGGGGCCTGGAGCAGCCAGTCCTCAAGCACTGGGAACCCCAGGTTCCATCCCCACTGCCAGAGACAACAGAAACTGTGGTCTGTGCCCTTGGACTGGCTGTGGGTCTGGTGGGCATCATTGTGGGCACCATCCTCATAATCAGAGCCACGCGTTCCAACAGTAGGGTCCAACATCAAGGGCCTCTGTAA

>Trvu_DCA_1

ATGACCTCCGGCCTACACTCAATATTGGGGATTCTCAGCCTGGCTACCTTGCTGATCAAACAAAGAACCTGGGTCACTAATGACCCTGTAATCAGCTCCATGACATTTGTTCAGACCCACAAACCTTCTGGCAGTACCTGCATGAATTTGATGAGGATGAGCAGTTCCATGTGGACTTTGACAGGAAGGAAACAGTCTGGTGGCTTCCTGAGTTTGGCCACATCTTCAGTTTCCATGCACAGATTGGGCTAGGCAACATTGCTGTGGACATGGCTAACTTGAACCAACTTATCAGGCAAAACAACCACACCCAGGCCACCATTGTGACTCCAGAGGTGACAGTGTTTCCCAAGGAGCCCGTGGAGCTAGAAGAACCCAGAATCCTCATCTGCCACACTGATAAGTTCTCACCCCCAGTGGTCAATGTCACGTGGCTGTGCAATGGCAAGCCAGTCACCACAGGGGTGTCTGAGACCACCTTCCTGCATCAGGATGACTATTCTTTCCACAAGTTCAATTAGCTCGCTTTCCTGCCCTCAGCTGATGATGTCTATGACTGTGTAGTTGAACACTGGGGCCTGGAAAAGCCACTTTTCAAGCACTGGGATATGAAGCCTGAGATGCTAACACCACCTTCTGAGACAGTGGAGACACTCATCTGTATTCTAGGACTGACTGTGGGCGTGGTGGGCAACCCTGTTGCTGCCACCCTCATTATCAAAGGCTTGTGATTAAGCAAATGATGCCTGACCCCATATATGGATCAAGTATCAACTTCTGCAAGAAATCTTTTCCAATGCTCCTTAATCTTAGTGCTTTCCCCTGA

>Trvu_DMA_1

ATGGGACCTGAGCAAAGCCTGGGAGCTACACTGTTACTCCTGCAGCTGCAGTCATCCCTGTCCTGGGGAGCCACTCCACAGTGTTGGCATCATTATTGGGGAATAACCTAGAGAACTACACATTCTCTCACACACTGTTCTGCCAGGATATGGAACCCCTCCTGGGTCTGTCCGAGACCTTCAATGGGGACCAGCTCTTCTCCTTCGACTTCTCCAGGAACTCTCGGGTGCCCCGGCTGCCTGAGTTTGCTGCTTGGGCCGGTGATGAGGGAGACATCAAGGCCATAGAAGATGACAAGAAGCTCTGCCAGGAATTGCAAAACGTTTTGAGTAGAATTTTGGAAGGCCAAATCCCTGAAGCTAGAGGAAACCCTGTGCCTGAAGTTTTCACTCTGGAGCCCCTGGAGTTTGGGAAGCCCAATACACTCGTCTGCTTTGTTAGTAACATCTTCCCACCTCAAATAACTGTGAGCTGGCAGCACAAAGGAGTCTCTGTGGAAGGCAGTAGCCCCACTTTTCTCTCAGCTATAGATGGACTTGGCTTCCAGGCCTTCTCCTATCTGAACTTCACACCCACATCCTCTGATGTCTTCTCTTGCAATGTGGCACGGGAAGGTGACCTCTTCAGTGCTATAGGCTTCTGGGTGCCTGAGAATCCACTACCCTCTGAATTGTTGGAAAATGTACTGTGTGGTATTGCCTTTGGCCTGGGAATTGTCGGCATCATAGTGGGTATTGCCCTCATCATCTACTCCCGAAAACCATGTCTAAGTGGTACAGGTGA

>Pscu_DNAzoo_DAA_1

ATGGCCCCCAACAAAGCCTTGATCCTAGAAGCCTTCATCCTGGCAGTGCTGCTGAGTCCCTGGGGAGCCAGGGCCATTAAAGAGAACCATGTGATCATCCAAGCAGAGTTCTACCAGACCGCCAAACCCTCCGGAGAGTTCATGTTTGACTTTGATGGGGATGAGATTTTCCATGTGGATTTGGAAAAGAAGGAGACAGTCTGGCGGCTTTCTGACTTCAGCAAATTTGCCAGCTTTGAGGCTCAGGGTGCTTTGGCCAATCTTGCTGTGGACAAAGCCAATCTGGAAATCATGATGAAACGGTCTAACAACACTCCTGACACCAATGTGCCCCCTGAAGTGACAGTGTTTCCCAAGGGCCCAGTGGAGCTGGAACAGCCCAACGTCCTTGTCTGCTTTGTTGACAAGTTCTCTCCTCCTGTACTTACTGTGACATGGCTTCATAATGGGAATCCCATCACTGATGGTGTGTTTGACACTGTCTTCCTTCCTCGCCCTGACCATACCTTCAGAAAATTCCACTACCTCACCTTCATCCCCTCTGCCACTGATTACTATGACTGCAAGGTCGAGCACTGGGGACTGGAACAACCTGTTGTCAAACACTGGGAACCAGAAATACGGACCCCACTGCCAGAGACAACAGAGACTGTGGTCTGTGCCCTAGGCCTGGCCATAGGCCTGGTGGGCATCATCGTAGGCACCATCCTTATTATCAAGGGCATGAAATCAAACAACACTTCCCGTGGTGGCTCCCGTGGACCCCTGTAA

>Pscu_DNAzoo_DMA_1

TGGGACCTGTGCAAATCCTGGGAGCTACACTGTTATTCCTGCAGCTGCAGTCATCGCTGTTTTTGTTTCTGTCCTGGGGAGCCACTCCACAGTGTTGGCATCATTATTGGGGAATAGCCTACAAAACTACACATTCTCTCACACACTGTTCTGCCAGGATGAGGAACCCTTCCTGGGTCTATCGGAGACCTTCAATGGGGACCAGCTCTTCTCCTTCGACTTCTCCAGGAACTCTCTGGTGCCCCGGCTGCCTGAGTTTGCTGCTTGGACTGGTGATGAGGAAGACATTGAGACCATAAAAAGTGATGGGAAGCTCTGCCAGGAATTGCAAAAAGTTTTGAGTAGAATTTTGGAAGACCAAATCCCTGAGGCTAGAGGAAACCCTGTGGCTGAAATTTTCACTCTGGAGCCCCTGGAGTTTGGGAAGCCCAACACTCTCACCTGCTTTGTTAGTAACATCTTCCCACCTCAAATAACTGTGAGCTGGCAGCACAAAGGAGTCCCTGTGGAAAGCAGCAGCCCCACTTTCCTCTCAGCTGTGGATGGACTTGGCTTCCAGGCCTTCTCTTATCTGAACTTCACACCCACATCCTCTGATGTCTTCTCTTGCACTGTGGAACGGGAAGGTGACCTCTTCAGCACTATCACCTACTGGGTGCCTCAGGATCCGATACCCTCTGAATTGTTGGAAAATATACTGTGTGGTATTGCCTTTGGCCTGGGAATCGTTGGCATCATAGTGGGTGCTGCCCTCATCATCTACTTCCGAAAACCATGTGCAAGTGGTGCAGGTAA

>Smcr_DNAzoo_DAA_1

ATGATCCCCAACAAAGCTTTGATCCTAGGGGCTTTCACCCTGGCAGTGTTGCTGAATCCCTGGGGAGCTAGAGCCATTAAAGAGAATCATGTCATCATCCAAGCTGAGTTCTACCAGACCCACAACCCCTTAGGAGAGTTCATGTTTGATTTTGATGGGGATGAAATTTTCCACGTGGGTTTGGATAAGAGACAGACAGTCTGGCGTCTTCCTGAATTCAGCAATTTTGCCAGCTTTGAGGCTCAGGGTGCTCTGGCCAATCTTGCTGTGGACAAAGCCAATCTGGAAATCATGATGAAACGGTCCAACAACACTCCTGATGTCAATGTGCCCCCTGAAGTGACAGTGTTTCCTAAGGGCCCAGTGGAGCTGGGCCAGCCCAATGTCCTTATCTGCTTCATTGACAAGTTCTCTCCTCCAGTACTTAATGTGACATGGCTTCATAATGAGCGTCCCATCACTGAGGGTGTGTTTGAGACTGTTTTCCTCCCCCGCCCTGATCATACCTTCAGAAAATTCTACTACCTCACCTTCATCCCCTCTGCCAACGATTTCTATGACTGTAAGGTCGATCACTGGGGACTGGAACAACCTGTTATCAAACATTGGGAACCAGAAATACGAACCCCACTGCCAGAGACAACAGAGACTGTGGTTTGTGCCCTGGGCCTAGCTGTTGGCCTAGTGGGCATCATTGCAGGCACCATCCTGATTATCAAGGGCATGCAATCAAGCAACAATTCCCGTGGTGGCCCTCGTGGACCCCTGTAA

>Smcr_DNAzoo_DMA_1

ATGGGACTTGAGCAAAACCTGGGAGTTACACTGTTACTACTGAAGCTACAGTCATCATTGCTTTTATCCCTGTTTTGGGAAGCCACTCCAGTGTTGGCAGTGTTTGGGAATAATTTACAGAATTACACATTCTCTCACACACTATTCTGCCAGAATGGAGAATCTCCCTTGGGTCTGTCAGAAGACTTCAATGGGGACCATCTCTTCTCCTTTGACTTCTCCAAGAACTCCCGGGTGCCTCGGCTGCCTGAATTTGCTGCTTGGGCCACTGATAAAGGAGACATTAAGAGCATAGATGCTGACAAGAATCTCTGCCAGCAGCTGCAACATGAATTGAGTAAACTTTGTAAAGGACAGATCCCTGAGGCCAGAGGAAACCCTGTGGCTGAAGTTTTCACTCTGGAGCCCCTGGAGTTTGGGAAGCCCAACACTCTTGTTTGCTTTGTTAGCAATCTCTTCCCACCTCATGTAACCGTGACCTGGCAACACGAAGGAGTCTCTGTGGAAAGCAGCAGCCCCACCTTTCTGTCAGCTATAGATGGACTTGGCTTCCAGGCCTTTTCTTATCTGAATATCACACCCACATCCACTGATGTTTTCTCGTGCACTGTGGCACAGGAAGGTGACCTCTTCAGCACTATAGCTTTTTGGGTGCCTCAGAATCCAATACCCTCTGCATTGTTGGAAAACATACTGTGTGGTATTGCCTTTGGCCTGGGCATTGTTGGTATCATCGTGGGTGCTTCCCTCATCATCTACTTCCAAAAGCCATGTGTAAATGGTGCAGGTGA

>Pegu_DAA_1

ATGGCCCCCAACAAAGCCTTGATCCTAAGTATCTTCACTCTGACAATGCTACTGAATCCCTGGAGAGCCAGGGCAATTAAAGAGAAACATGTGATCATCCAAGCAGAATTCTACCAGACTCATGAACCCTCAGGAGAGTTCATGTTTGACTTTGATGGAGATGAGATTTTCCATGTGGATTTGCAAAAGAAGGAGACAGTTTGGCGACTTTCGGACTTCAGCAAATTTGCCAGTTTTGAGGCTCAGGGTGCTCTAGCCAATCTTGCTGTAGACAAAGCCAACCTGGAAATCATGATGAAACGATCCAACAACACCCCTGACACCAATGCCCCCTGAAGTGACAGTGTTTCCTAAAGGCCCAGTGGAGCTGGGTCAGCCCAACGTCCTCATCTGCTTCATTGACAAGTTCTCTCCTCCTGTACTTAATGTGACATGGCTTCATAACAAGCGTCCTATCACTGATGGTGTATTTGAAACTGTCTTCCTCCCTCGCTCTGACCATTCTTTCAGAAAATTCCACTATCTCACCTTTATCCCTTCTGCTGATGATTTCTATGACTGTAAAGTTGATCACTGGGGACTGGAAGAGCCTGTACTTAGACATTGGGGTAAAAATACGGACTCCACTGCCAGAGACAACAGAAACTGTGGTCTGTGCCCTGGGCTTGGCCATAGGCCTGGTGGGCATCATTGTAGGCACCATCCTGATTTTTAAGGGAATGCGAGCAAGCAACAATTCCCGAGGTGGCCCCCGAGCACCCCTGTAA

>Pegu_DBA_1

ATGGCCATCAACAGAATCCTGATCCTAAGAACCCTCTCACTGGCTGTGTTGCTGAGTCCCCCAGGGGCTTCCGAGTCCATTGAAGCTGACCATGTGGGGGTCTATGGCACAACTGTGTACCAGTACTATGGGCCCTCAGGCCAGTATACACAGGAGTTTGATGAGGATGAGCTCTTTTATGTGGACCTGCAAAAGAAGGAGACTGTATGGCGGCTGCCAGAGTTTAGTCATTTTGCTGGCTTTGATCCTCAAGGGGGGCTGACAAACATAGCCACAGCAAAGTACAACCTGGACATCCTGATGAAACGCTCCAACAGAAGCAGGGTCATCATACCCCCAGAGGTGACTGTGTTCTCAGAGAGCCCTGTGGAGGTGGGCCAGCCAAATGTCCTCATCTGCTTCGTGGACAACATCTTCCCCCCAGTGGTCAACATCACATGGCTACGTAATGGACAGCTGGTCACCACAGGTGTGTCTGAGACAGACTTCTACACTCGACCTGACGACAGATTCCGCAAGTTCTACTACCTCACTTTTCTCCCCAACACAGAAGATTTTTATGACTGCAAAGTGGAACACTGGGGCCTGGAGCAGCCGGTCCTCAAGCACTGGGAACCCCAAGTTCCATCCCCACTGCCGGAGACAACAGAAACTGTGGTCTGTGCCCTTGGTCTGGCTGTGGGCCTGGTGGGCATCGTTGTGGGCACTATCCTCATTATCAGAGGCATGCGTTCTAGCAGTAGGATCCAACATCAAGGGCCTCTGTGA

>Pegu_DCA_1

ATGACCTTTAGTCTAGTCTCAGGTTTAGGGATCCTCAGCCTGGCTGCCCTGCTAATCAAACTAAGAGCCTGGGCTGTTAATAGACCATGTAATCAACTCTGTGACATTTGTTCAGACTCACAGACATGTATGAATATGATGAGTTTAAGTAATTCCATGTCAAGACAGGAAGAAAATAGTATGGAAGCTTCCTGAGTTTGGCCACATCTTTAATTTTGATGCCCAGATTGGACTAGGCAACACTACTGTGGATCTGGCTAACTTGAATGCCTTATCAGGCAAAACGCCCATCGTGTCTGTGGAGGGGACAGTGTTTCCCATGGAGCTTGTGTAACCAGAGGAGCCAAACACCCTCATCTGCCATATTGATATGTTCTCCCCTCCAGTGATTAATGCCACATGGTTGTGCAATGGACAACCGGTTACTATAGGGTATCTCAGACCTCCTTCCTGCCTTGTGATGACTTTTCTTTCCACAAGTTCCATTTCCTCACTTTCCTCCCCTCAACTGATGATGTCTGTGACTGCATGGTTGAACACTAGGGCCAGGAGGAGCCACTTTTCAAGCACTGGAAGTTTGAGATGCTAACACCACCATCTGAAACACTAGAGACCCTATTTTTATTCTAGGACTGGCTGTGGGCCTGGTGAACATCACTGTGGCTACTGCCTTCCTCATCAAATCTTGTGCTCAGGCAAATGA

>Pegu_DMA_1

ATGGGACCTGAGCACAGCCTGGTAGCTACACTGTTACTACTGCAACTTCACTCCTCACTTCTTTTGTCCCTGTCCTTGGAAGACACTCCAGAATTAACATCATTACCGGAGAACAGCCTACAGAACTACACATTCTTTCACACGATATTCTGCCAAAATGAGGAACCCTTTGTGGGTCTGTCGGAGACCTTTAATGGGGACCAGCTCTTCTCCTTTGACTTCCCCAGAAACTCCCGGGTGCCCCGGCTGCCTGAGTTTGCTGCTTGGGCCAGTGATGAGGGAGATGTCCAGGCCATAGATGCTGAAAAAGTTCTCTGCCAGAATCTGCTAAACACATTCAGTCAAGTTTGTGAAGGCAAGATCCCTGAGTCTAGAGGAAACCCTGTGGCTGAAGTTTTCACTCTGGAGCCCCTGGAGTTTGGAAAGCCCAACACACTCATCTGCTTTGTTAGTAATCTCTTCCCACCTCAAGTATCCGTGAAGTGGCAACATGATGGAGTCCCTATAGAAAGCAACAGCCCCACTTTTCTCTCAGCTGTAGAAGGACTTGGTTTCCAGGCTTTCTCTTACCTGAACTTCACACCCACGTCTTCAGATATCTTTACTTGCATTGTGGAACGGGATGGTGACCTCTTCAGTACTATAGCATTCTGGGTGCCTCAGAATCCAATACCTTCTACATTGTTGGAAAACATACTGTGTGGTATTGCCTTTGGCCTGGGCATTGTTGGCATCATAGTGGGTGCTGCCCTTATCATCTACTTCCAAAAACCATGTGCAAGTGGTGCAGGTAA

>Magi_DNAzoo_DAA_1

ATGACTTCCAACAAATCCTTGATCCTAGGAGCCTTCATTCTGTCAGTGCTGCTGGGTCCCTGGGGAGCCAGGGCCATTAAAGAGAACCATGTGATCATCCAAGCAGAGTTCTACCAGACCCACGAACCCTCTGGAGAGTTTATGTTCGACTTTGATGGGGATGAGATTTTCCATGTGGATTTGAACAAGAAAGAGACAGTCTGGCGGCTTCCTGACTTCAGCAAATTTGCCAGCTTTGAGGCTCAGGGTGCCTTGGCCAATCTTGCTGTGGACAAAGCCAACCTGGAAATCATGATGAAACGATCCAATAACACCCCTGACACCAATGTGGGCCCTGAAGTGACAGTGTTTCCCAAAGGCCCAGTGGAGCTGGGCCAGCCCAACATCCTTGTCTGCTTCATTGACAAGTTCTCTCCTCCGGTACTTACTGTGACCTGGCTTCATAATGGGGTTCCCATCACTGATGGTGTGTTTGAAACTGTCTTCCTCCCTCGCTCTGACCATGCCTTCAGAAAATTCCACTACCTCACCTTCATCCCCTCTGCCACCGATTACTATGACTGTCAGGTTGAGCACTGGGGACTGGAACAACCTGTTGTCAAACACTGGGAACCAGAAGTACGGACCCCACTGCCAGAGACAACAGAGACTGTGGTCTGCGCCCTAGGCCTGGCCATAGGCCTTGTGGGCATCATCGTAGGCACCATTCTTATTATCAAGGGCATGAGATCAAACAACACTTCCCGTGGTGGCTCCCGTGGACCCCTGTAA

>Magi_DNAzoo_DBA_1

ATGGCTGCCAGCAGAGTGCTGATCCTAAGGACCCTGTCACTGGTTGTGCTGCTGAGTCCCCAAGGAACTTCTCAGTCCATTGAAGGCAACTGACCACGTGGGGATCTATGGCACAGGTGTATACCAGTCCTATGAGTCCTCAGGCCAGTACACACAGGAATTTGATGAGGACGAGCTGTTTTACGTAGACCTGCAGAAGAAGGAGACTGTGTGGCGGCTACCAGAGTTTAGCCATTTTAGCAGCTTTGACCCTCAGGGAGGGCTGCGTGAAAAAGCCACATGCAAGTACAACCTGGACATCCTGATCAAGCGCTCCAACAGAAGCAGGGCCATTATGCCCCCTGAAGTGACTGTGTTCTCGGAGAGTCCTGTGGAGTTGGGGCAGCCAAATGTACTCATATGCTTGGTGGACAACATCTTCCCTCCTGTGGTCAACATCAAGTGGCTTCGTAATGGCCAGGTGATCACCACTGGTGTGTCTGAGACAGACTTCTACTCTCGGCCTGACCACAAATTCCGCAAGTTCTACTACCTCACTTTTCTCCCCAACACAGAGGACTTTTATGACTGCAAAGTGGAGCACTGGGGCTTGGAGCAGCCACTCCTCAAGCACTGGGAACCCCAGATTCCATCCCCAGTGCCAGAGACAACAGAAACTGTGGTCTGTGCCCTTGGTCTGGCAGTGGGCCTGGTGGGCATCATTGTGGGCACCATCCTTATAATCAGAGGCATGCGTTCCAGCAATAGGTTCCAACATCAAGGGCCTCTGTGA

>Magi_DNAzoo_DBA_2

ATGGCTGTCAACAGAGTTCTGATCCGAGGGACCCTCTCATTAGTTTTGCTCCTGAGTCCCCAAGGAGCTTGAGTCTGTTGAAGCCAAATTGCTGACCATGTTAGGGTCTATGGCACAGATATATGCCAGAACTATGGGCCCTCAAGCCAGTACACACAAGAATTTGATGATAATGAGCTGTTTTATGTGGATCTGCAGAAGGAGATTGTATGGTGGCTGCCAGAGTTTAGCCATTTTGCTGGCTTTGACCCTCAGGGAGGGTTAAGAAACATACCCATACCCAAGTTCACCATGGACACCCTCATCAAACACTCCAACAGAAGCAGGGCCATCAGGGTGCCCCCTGAGGTAACAGTATTCTCAGAGAATACTGTGGAGATAGGCCAGCCAAATGTACTCATCTGCTTGGTGGACAACATCTTCCCCTCAGCTGTCAGCATCATGTGGCTTCATAATGACCAGTTGGTCACCACTGGTGTGTCTGAGACAGACTTTTACTCTCGGCCTGACCATGAATTCCACAAGTTCTACTACCTCACTTTTCTCCCCAACACAGAGGATTTTTATGACTGCAAAGTGGAGCACTGGGGCTTGGAACAGCCAGTCCTCAAGCACTGGGAACCCCAGATTCCATCCCCAGTGCCAGAGACAACAGAAACTGTGTTCTGTGCCCTTGGTCTGTCAGTGGGCCTGGTGGGCATCATTGGGGGCACCATCCTCATAATCAGAGGCATGCGTTCCAGCACTCGGATCCAACATCAAGGGCCTCTGTGA

>Magi_DNAzoo_DBA_3

ATGGCTGCCAGCAGAGTTCTGATCCTAGGGACCCTCTCACTGACTGTGCTGCTGAGTCCCCAAGGAGCTTCTGAGCCCATCGAAGGCAACTGACCATGTGGGGCTCTATGGCACAGATATATACCAGACCTATGGGCCCTCAGGCCAGTACACACAGGAATTTGATGAGGACGAGCTGTTTTACATAGACCAGCAGAAGAGGGAGACTGTGTGGCGGCTGCCAGAGTTTAGCCATTTTGCTAGCTTTGACCCTCAGGGAGGACTGCGTGAAATAGCCATATGCAAGCACACCCTGGACATCCTGATCAAACGCTCCAACAGAAGCAGGGCCGTCATGCCGCCTGAAGTGACTGTGTTCTCAGAGAGTCCCGTGGAGGTGGGCCAGCCAAATACACTCATCTGCTTGGTGGACAACATCTTCCCTCCAGTAGTCAACATCACGTGGCTTCGTAATGGCCAATTGGTCACCATAGGTGTGTCTGAGACAGACTTCTACACTCGGCCTGACCACAAATTCCGCAAGTTCCACTACCTCGCTTTTCTCCCCAACACAGAGGATTTTTATGATTGCAAAGTGGAGCACTGGGGCTTGGAGCAGCCAGTCCTCAAGCACTGGGAACCCCAGGTTCCATCCCCACTGCCAGAGACAACAGAAACTGTGGTCTGTGCCCTTGGTCTGGCTGTGGGCCTGGTGGGCATCATTGTGGGCACCATCCTCATAATCAGAGGCATGCCTTCCAGCAGCAGGATCCAACATCAAGGACCTCTGTGA

>Magi_DNAzoo_DBA_4

ATGGCTGCCAGCAGAGTTCTGATCTTAGGGACCCTCTCACTGGCTGTGCTACTGAGTCCCCAAGGAGCTTCTGAGTCCATTGAAGGCAACTGACCATGTTGGGCTCTATGGTACAGATATATACCAATCCTATGGGCCCTCAGGCCAGTACGCACAGGAATTTGATGAAGATAAAGAATTTTATGTAGCCTTGCAGAAGAAGGAGACTGTGTGGTGGCTGCCAGAGTTTAGCCATTTTGCTCGCTTTGACCCTCAGGGAGGACTGCATGAAATAGCCACAAGTAAGTACAACCTGGACATTGTAATCAAATGCTCCAACAGAAGCAGGGCCATCAGCGTGCCGCCTGAGGTGACTATGTTCTCAGAAAGCCTGTGGAGGTGATGGGCCAGCCAAATATACTCATCTGATTGGTGAACAACATCTTCCCTCCAGTGGTCAACATCACATGGCTTCTTAGTTGCAGTTGGTCACCATAGGTGTGTCTGAGACAGACTTCTACTCTCAGCCTGACCACAAATTCCGCAAGTTCTACTACCTCACTTTTCTCCCCAACACAGAGGATTTTTATGACTGCAAAGTGGAGCACTGGGGCTTAAAGCAGCCGGTCCTCAAGCACTGGGAACCCCAGTTTCCATCCCCATTGCCAGAGACAACAGAAACTGTGATCTGTGCCCTCGGTTTGGCAGTGGGCCTGGTGGGCATCATTGTGTGCACCATCCTTATAATCAGAGGCATGCATTCCAGCAGTAGGATCAAGGGCCTCTGTGA

>Magi_DNAzoo_DBA_5

ATGGGTGCCAGCAGAGTCCTGATCCTAGGGACCCTCTCATTGGTTGTGCTGCTGAGTCCCCAAGGAACTTCTCAGTCCATTGAAGGCAACTGACCATGTGGGGATCTATGGCACAGGTGTATGCCAGTCCTATGGGCCCTCAGGCCAGTACACACAGGAATTTGATGGGGATGAAAAATTTTATGTAGACCTGCAGAAGAAGGAGACTGTGTGGCGGCTGCCAGAGTTTAGCCATTTTACCAGCTTTGACCCTCAGGGAGGACTGCGTGAAATAGCCACATGCAAGTACAACCTGGACATCCTGATCAAACGCTCCAACAGAAGCAGGGCCATCATGCCCCCTGAAGTGACTGTGTTCTCAGAGAGTCCTGTGGAGGTGGGCCAGCCAAACATACTCATCTGCTTGGTGGACAACATCTTCCCTCCAGTGGTCAACATAAAGTGGCTTCGTAATGGCCAGGTCATCACCACTGGTGTGTCTGAGACAGACTTCTACTCTCGGCCTGACCACAAATTCCGCAAGTTCTACTACCTCACTTTTCTCCCCAACACAGAGGAATTTTATGACTGCAAAGTGGAACACTGGGGCTTGGAGCAGCCAGTCCTCAAGCACTGGGAACCCCAGTTTCCATCCCCAGTGCCAGAGACAACAGAAACTGTGGTCTGTGCCCTTGGTCTGGCAGTGGGCCTGGTGGGCATCATTGTGGGCACCATCCTTATAATCAGAGGCATGCGTTCCAGCAGTAGGATCCAACATCAAGGGCCTCTGTGA

>Magi_DNAzoo_DBA_6

ATGGCTGCCAGCAGAGGGCTAATCCTAAGGACGCTGTCACTGGTTGTGCTGCTGAGTCCCCAAGGAACTTCTCAGTCCATTGAAGGCAACTGACCACGTGGGGATCTACGGCACAGGTGTATACCAGTCCTATGAGTCCTCAGGCCAGTACACACAGGAATTTGATGAGGACGAGCTGTTTTACGTAGACCTGCAGAAGAAGGAGACTGTGTGGCGGCTGCCAGAGTTTAGCCATTTTAGCAGCTTTGACCCTCAGGGAGGGCTGCGTGAAAAAGCCACATGCAAGTACAACCTGGACATCCTGATCAAACTCTCCAACAGAAGCAGGACCATCATGGCCCCTGAAGTGACTGTGTTCTCAGAGAGTCCTGTGGAGTTGGGCCAGCCACATGTACTCATCTGCTTGGTGGACAACATCTTCCCTCCAGTGGTCAACATCAAGTGGCTTTGTAATGGCCAGGTGATCGCCACTGGTGTGTCTGAGACAGACTTCTACTCTTGGCCTGACCACAAATTCCGCAAGTTCTACTACCTCACTTTTCTCCCCAACACAGAGGCCTTTTATGACTGCAAAGTGGAGCACTGGGGCTTGGAGTAGCCACTCCTCAAGCACTGGGAACCCCAGATTCCATCCCCAGTGCCAGAGACAACAGAAACTGTGGTCTGTGCCCTGGGTCTGGCAGTGGGCCTGGTGGGCATCATTGTGGGCACCATCCTTATAATCAAAGGCATGCGTTCCAGCAGTAGGATCCAACATCAAGGGCCTCTGTGA

>Magi_DNAzoo_DBA_7

ATGGCTGCCAGTAGAGTGCTGATCCTAAGGACCCTGTCACTGGTTGTGCTGCTGAGTCCCCAAGGAACTTCTCAGTCCATTGAAGGCAACTGACCATGTGGGGATCTATGGCACAGGTGTATACCAGTCCTATGAGTCCTCAGGCCAGTACACACAGGAATTTGATGAGGACGAGCTGTTTTATGTAGATCTGCAGAAGAAGGAGACTGTGTGGCGGCTGCCAGAGTTTAGCCATTTTAGCAGCTTTGACCCTCAGGGAGGGCTGCGTGAAAAAGCCACATGCAAGTACAACCTGGACATCCTGATCAAGCGCTCCAACAGAAGCAGGGCCATCATGCCCCCTGAAGTGACTGTGTTCTCGGAGAGTCCTGTGGAGTTGGGCCAGCCGAACGTACTCATCTGCTTGGTGGACAACATCTTCCCTCCAGTGGTCAACATCAAGTGGCTTCGTAATGGCCAGGTGATCACCACTGGTGTGTCTGAGACAGACTTCTACTCTCGGCCTGACCACAAATTCCGCAAGTTCTACTACCTCACTTTTCTCCCCAACACAGAGGACTTTTATGACTGCAAAGTGGAGCACTGGGGCTTGGAGCAGCCACTCCTCAAGCACTGGGAACCCCAGATTCCATCCCCAGTGCCAGAGACAACAGAAACTGTGGTCTGTGCCCTGGGTCTGGCAGTGGGCCTGGTGGGCATCATTGTAGGCACCATCCTTATAATCAGAGGCATGCGTTCCAGCAATAGGTTCCAACATCAAGGGCCTCTGTGA

>Magi_DNAzoo_DCA_1_partial_exon_2-4

ACCATGGAATCAGCTCTGTGACAATTGTTCAGACCTACAAGCCATCTGAGCAGGACCTGCATGAATTTGATGAAGATGAACCATTTCACATGGACTATGAAAAGAAGGAAACAGTCTGGCAGCTTCCTGAGTTTGGCCGTATCTTCAGTTCCAGTGCACAGATTGGGCTAGGTGACATTGCTGCGGACATGGCTAACTTGAACCAACTTATCAGGCAAACCAAGCACACCCAAGCCACCATTGTGACTCCAGAGGTGGCAATGTTTCCCAAGGAGGCCGTGGAACTAGAAGAACCCAGCGTCCTCCTCTACCACATCCTAGTGATCAATGTCACATGGCTGTGCAATGGTGAGTCAGTCACCACAGCAGTATCTGAAACTGTCTTCCTGCCTCAGGATGACTGTTCTTCCCACAAGTTTCATTACTTCACTTTCTTCCTCTCAACTGATGATATTTATGACTGCGTAGTTGAACCCTGGGGCCTCAAAAACCACTTTCCAAGTGTTGGGATATGAATCAGTACATAGCCTGAGATGCTAACACCACCGTCTGAGACAATGGAGATGCTCTTCTCTATTCTAGGAATGGCTATGGGCCTGGTGGGCATCATGGTAGCTGCCAGCTTCATTATCAGAGGCTTGTGCTCAGGCAAATGGTTCCTGA

>Magi_DNAzoo_DMA_1

ATGGGACCTGTGCAAAGCCTGGGAGCTACACTGTTACTCCTGCAGCTGCAGTCATCACTGTTCTTGTCTCTGTCCTGGGGAGCCACTCCAGTGTTGGCATCATTACTGGGGAATAGCCTACAGAACTATACATTCTCTCACACAATGTTCTGCCAGGATGAGCAACCCTTAGTGGGTCTGTCTGAGGTCTTCGATGGGGACCTGCTCTTCTCCTTCGACTTCTCCAGGAACACTCGGGTACCCCGGCTGCCTGAGTTTGGTGCTTGGGCCAGTGATGAGGGAGATGTCAAGATCATAGAAGATGACAAGACACTCTGCCAGGGATTGCAGAAATTTTTGAGTGAACATTTTGAAGGCCGAATCCCAGAGGCTAGAGGAAACCCTGTGGCTGAAGTTTTCACTCTGGAACCCCTGGAGTTTGGGAAGCCCAACACTCTCATTTGCTTTGTTAGTAACATCTTCCCACCTCAGATAACAGTGACTTGGCAGTACAAAGAAGTTCCTGTGGAAAGCAGCAGCCCCACTTTTCTCTCTGCTGTAGATGGACTTGGCTTCCAGGCCTTCTCCTATCTGAATTTCACACCCACTGCCTCTGATATCTTCTCTTGCACTGTGGCACGGGAAGGCGAACTCTTCAGCACTATAGCCTTCTGGGTGCCTCAGAATCCGATACCCTCTGAATTGCTGGAAAATATACTGTGCGGTATTGCCTTTGGCCTGGGAATTCTAGGCATCATAGTGGGTGCTGCCCTCATCATCTACTTCCAAAAACCATGTGGAAGTGGTGCAGGTAA

>Davi_DNAzoo_DAA_1

ATGATGCCCAACAAAGCTTTGATCCTAGGGGCTTTCACCCTGGCCGTGCTGTTGAATCCCTGGGGAGCCAGAGCCATTAAAGAGAATCATGTGATCATCCAAGCTGAGTTCTACCAGACCCACAACCCTTTAGGAGAGTTCATGTTTGATTTTGATGGGGATGAAATTTTCCATGTGGATTCGAGCAAGAGAGAGACAGTCTGGCGTCTTCCTGACTTCAGCAAATTTGCCAGCTTTGAGGCTCAGGGTGCTCTGGCCAATCTTGCTGTGGACAAAGCCAATCTGGAAATCATGATAAAACGGTCCAACAACACTCCTGATACTAATGTGCCCCCTGAAGTGACAGTGTTTCCTAAGAGCCCAGCGGAGATGGACCAGCCCAATGTCCTTATCTGCTTCATTGACAAGTTCTCTCCTCCCGTACTTAATGTGACATGGCTTCGTAATGGGCAGCCCATCACTGATGGTGTGTTTGAGACTGTCTTCCTCCCCCGCCCTGATCATGCCTTCAGAAAATTCCACTACCTCACCTTCATCCCCTCTGCCAATGATTACTATGACTGTAAGGTCGATCACTGGGGACTGGAACAACCTCTTATGAAACATTGGGAACCAGAAATACGAACCCCACTGCCAGAGACAACAGAGACTGTGGTCTGTGCCCTGGGCCTAGCCATTGGTCTGGTGGGCATCGTTGTGGGCACCATCCTGATTATCAAGGGCATGAAATCAAGCAACGCTTCCCGTGGTGGCCCTCGTGGACCCCTGTAA

>Davi_DNAzoo_DMA_1

ATGGGACTTGAGCAAAACCTGGGAGTTACACTGTTACTACTGAAGCTACAGTCATCATTGCTTTTGTCCCTGTTCTGGGAAGCCACTCCAGTGTTGTCCGTGTTTGGGAATAATTTACAGAATTATACATTCTCTCACATACTATTCTGCCAGAATGGAGAATCCTCTTTGGGTCTGTCAGAAAACTTCAATGGGGACTACCTCTTCTCCTTTGACTTCTCCAAGAACTCCCGGGTGCCCCGGCTGCCTGAATTTGCTGCTTGGGCCACTGATAAAGGAGACATCAAGACCATAGATGCTGACAAGAATCTCTGCCAGGAGCTACAACATCAATTGAGTAGACTTTGTAAAGGACGGATCCCTGAGGCTAGAGGAAATCCTGTGGCTGAAGTTTTCACTCTGGAGCCCCTGGAGTTTGGGAAGCCCAACACTCTCGTTTGTTTTGTTAGCAATATCTTCCCACCTCGTATAACCGTGACCTGGCAACATGAAGGAGTCTCTGTGGAAAGCAGCAGCCCCACCTTTCTCTCAGCTACAGATGGACTTGACTTCCAGGCCTTTTCTTATCTGAACATCACACCCACATCCACTGATGTTTTCTCTTGCACTGTGACACAGGAAGGTGACCTCTTCAGCACTATAGCATTTTGGGTGCCTCAGAATCCAATACCCTCTGCATTGTTGGAAAACATACTGTGTGGTATTGCCTTCGGACTGGGCATTGTTGGTATCATTGTAGGTGCTTCCCTCATCATCTACTTCCAAAAGCCATGTGTAAATGGTGCAGGTGA

>Pogi_DNAzoo_DAA_1

ATGACCCCCAACAAATCCTTGATCCTAGGAGCCTTCATTCTGTCAGTGCTGCTGAGTCCCTGGGGAGCCAGGGCCATTAAAGAAAACCATGTGATCATCCAAGCAGAGTTCTACCAGACCCATGAACCTTCTGGAGAGTTTATGTTCGACTTTGATGGGGATGAGATTTTCCATGTGGATTTGCAGAAGAAAGAGACAGTCTGGCGGCTTCCTGACTTCAGTAAATTTGCCAGCTTTGAGGCTCAGGGTGCCTTGGCCAATCTTGCTGTGGACAAAGCCAACCTGGAAATCATGATGAAACGGTCCAATAACACCCCTGACACCAATGTGGGCCCTGAAGTGACAGTGTTTCCTAAAGGCCCAGTGGAGCTGGGCCACCCCAACATCCTTGTCTGCTTCGTTGACAAGTTCTCTCCTCCGGTACTTACTGTGACCTGGCTTCATAATGGGGTTCCCATCACTGATGGTGTGTTTGAAACTGTCTTCCTCCCTCGCTCTGACCATGCCTTCAGAAAATTCCACTACCTCACCTTCATCCCCTCTGCCACCGATTACTATGACTGCAAGGTCGAACACTGGGGACTGGAACAACCTACTGTCAAACACTGGGAACCAGAAGTACGGACCCCACTGCCAGAGACAACAGAGACTGTGGTCTGTGCCCTAGGCCTGGCCATAGGCCTTGTGGGCATCATCGTAGGCACCATTCTTATTATCAAGGGCATGCGATCAAACAACACTTCCCGTGGTGGCTCCCGTGGACCCCTGTAA

>Pogi_DNAzoo_DBA_1

ATGGCTGCCAGAACCGTGCTGATCCTAAGGACCCTCTCACTGGTTGTGCTGCTGAGTCCCCAAGGAACTTCTCAGTCCATTGAAGGCAACTGACCACGTGGGGATCTACGGCACAGGTGTGTACCAGTCCTATGAGTCTTCAGGCCAGTACACACAGGAATTTGATGGGGATGAGCTGTTTTACGTAGACCTGCAGAAGAAGGAGACTGTATGGAGGCTGCCAGAGTTTGGCCATTTTAGCAGCTTTGATCCTCAGGGAGGGCTGCGTGAAAAAGCCACATGCAAGTACAACCTGGACATCCTGATCAAACGCTCCAACAGAAGCAGGGCCATCATGCCCCCTGAAGTGACTGTGTTCTCAGAGAGTCCTGTGGAGTTGGGCCAGCCAAACGTACTCATCTGCTTGGTGGACAACATCTTCCCTCCAGTGGTCAACATCAAGTGGCTTCGAAATGGCCAGGTGATCACCACTGGTGTGTCTGAGACAGACTTCTACTCTCGACCTGACCACAAATTCCGCAAGTTCTACTACCTCACTTTTCTCCCCAACACAGAGGACTTTTATGACTGCAAAGTGGAGCACTGGGGCTTGGAGCAGCCACTCCTCAAGCACTGGGAACCCCAGATTCCATCCCCACTGCCAGAGACAACAGAAACTGTGGTCTGTGCCCTTGGTCTGGCTGTGGGCCTGGTGGGCATCATTGTGGGCACCATCCTTATAATCAGAGGCATGCGTTCCAGCAGTAGGATCCAACATCAAGGGCCTCTGTGA

>Pogi_DNAzoo_DBA_2

ATGGCTTCCAACAGAGTTCTGATCCTAGGGACCCTCTCATTGACTTTGTTGCTGAGTCCTCAAGGAGCTTCTGAGTCTATTGAAGCCAAATTTCTGACCATGTTGGGGTCTATGGTACAGACGTATGCCAAACCTGTGGGCCCTCAGGCCAATACACACAAGAATTTGATGAGAATAAGCTGTTTTATGTGGACCTGTGGAAGGAGACTCTATGGTGGCTGTCAGAGTTTAGCCATTTTGCTGGATTTCACCCTCAGGGAGGGTTGAGAAACATATGCATAACCAGGCTCACCCTGAACACCCCCATCAAACACTCCAACAGAAGCAGGGTCATCATGCCCCCTGAGGTGACTGCGTTCTCAGAGAGTCCCGTGGAGATGGGTCAGCCAAATGTACTCATCTGCTTGGTGGACAACATCTTCCCTCCAGTGATCAACATCACGTGGCTTCGTAATGGTGTCTGAGACAGACTTCTATACTCCGCCTGACCACAAATTCCGCAAGTTCTACTACCTCACTTTTCTCCCCAACACAGAGAACTTTTATGACTTCCAAGTGGAGCGTTGTTGTTCGGGGCAGCCAGTCCTCAAACATCGGCGTTCTATCCCCACTGACAGAGACAACAGAAACTGTGATCTGTGCCCTTGGTCTGGCTGTGGGCCTGTTGGGCATCATTGTGGGCACTATCCTCATAATCAGAGACATGCAATCCATAAGTAAGATCCAACATCAAGGGCCTCTGTGA

>Pogi_DNAzoo_DBA_3

ATGGCTGCCAGCAGAGTGCTGATCCTAAGGACCCTCTCACTGGTTGCGCTGCTGAGTCCCCAAGGAACTTCTCAGTCCATTGAAGGCAACTGACCACGTGGGGATCTATGGCACAGGTGTATACCAGTCCTATGAGTCCTCAGGCCAGTACACACAGGAATTTTATGAGGATGAGCTGTTTTACATAGACCTGCAGAAGGAGATTGTATGGCGGCTACCAGAGTTTGGTCATTTTAGCAGCTTTGACCCTCAGGGAGGGCTGCATGAAAAAGCCACATGTAAGTACAACCTGGACATCCTGATCAAACGCTCCAACAGAAGCAGGACCATCATACCCCCCTGAAGTGACTGTGTTCTCAGAGAGTCCTGTGGAGTTGGGCCAGCCAAACGTACTCATCTGCTTGGTGGACAACATCTTCCCTCCAGTGGTCAACATCAAGTGGCTTCGTAATGGCCAGGTGATCACCACTGGTGTGTCTGAGACAGACTCTACTCTCGGCCTGACCACAAATTCCGCAAGTTCTACTACCTCACTTTTCTCCCCAACACAGAGAACTTTTATGACTGCAAAGTGGAGCACTGGGGCTTGGAGCAGCCACTCCTCAAGCACTGGGAACCCCAGATTCCATCCCCACTGCCAGAGACAACAGAAACTGTGGTCTGTGCCCTTGGTCTGGCAGTGGGCCTGGTGGGCATCATTGTGGACATCATCCTTATAATCAGAGGCATGCGTTCCAGCAGTAGGATCCAACATCAAGTACCTCTGTGA

>Pogi_DNAzoo_DBA_4

ATGGCTGCCAGAACCGTGCTGATCCTAAGGACCCTCTCACTGGTTGTGCTGCTGAGTCCCCAAGGAACTTCTCAGTCCATTGAAGGCAACTGACCACGTGGGGATCTACGGCACAGGTGTGTACCAGTCCTATGAGTCTTCAGGCCAGTACACACAGGAATTTGATGGGGATGAGCTGTTTTACGTAGACCTGCAGAAGAAGGAGACTGTATGGAGGCTGCCAGAGTTTGGCCATTTTAGCAGCTTTGATCCTCAGGGAGGGCTGCGTGAAAAAGCCACATGCAAGTACAACCTGGACATCCTGATCAAACGCTCCAACAGAAGCAGGGCCATCATGCCCCCTGAAGTGACTGTGTTCTCAGAGAGTCCTGTGGAGTTGGGCCAGCCAAACGTACTCATCTGCTTGGTGGACAACATCTTCCCTCCAGTGGTCAACATCAAGTGGCTTCGAAATGGCCAGGTGATCACCACTGGTGTGTCTGAGACAGACTTCTACTCTCGACCTGACCACAAATTCCGCAAGTTCTACTACCTCACTTTTCTCCCCAACACAGAGGACTTTTATGACTGCAAAGTGGAGCACTGGGGCTTGGAGCAGCCACTCCTCAAGCACTGGGAACCCCAGATTCCATCCCCACTGCCAGAGACAACAGAAACTGTGGTCTGTGCCCTTGGTCTGGCTGTGGGCCTGGTGGGCATCATTGTGGGCACCATCCTTATAATCAGAGGCATGCGTTCCAGCAGTAGGATCCAACATCAAGGGCCTCTGTGA

>Pogi_DNAzoo_DCA_1_partial_exon_2-4

ACCATGTAATCAGCTCTGTGACAATTGTTCAGACCTACAAACTATCTGAGCAGTACCTGCATGAATTTGATGAAGACAAACAATTCCATATGGACTATGAAAAGAAGGAAACAGTCTGGCAGCTTCCTGTGTTTGGCCATATCTTCAGTTCCAATGCACAGCTTAGGCTAGGTGACATTGCTGTGGACATGTCTAACTTGAACCAACTTATCAGGCAAACCAACCACACCCAAGCCACCATTGTGACTCCAGAGGTGGCAGTGTTTCCCAAGGAGGCGTGGAACTAGAAGCACCCAGCGTCCCCATCTGCCACATTGATAAGTTCGCCATCCCAGTGATTAATGTCACATGGCTGTGCAATGGTGAGTCAGTTACCACAGGGGTACCTGAGACCACCTTCCTGCCTCAGGATGACTGTTCTTTCCACAAGTTTCATTACCTCACTTTCTTCCTTTCAACTGATGATATTTATGACTGTGCAGTTGAACACTGAGGCCTCAAAAACCACTTTTCAAGTAGCCAGAGATGCTAATATTACCGTCTGAGACGATGGAGATGCTCATCCATATTCTAGGAATGGCTATGGGCCTGGTGGGCATCATGGTGGCTGCCAGCTTCATTATCAGAGGCTTGTGCTCAGGAAATGATTCCTGACCCCATAGATCAAGTATAATATTCTGCAAGAAATCTTTCCAATCCTCCTTAATCTTAGTGCCTTCCCCTGA

>Pogi_DNAzoo_DMA_1

ATGGGACCTGTGCAAAGCCTGGGAGCTCCACTGTTACTCCTGCAGCTGCAGTCATCACTGTTCTTGTCCCTGTCCTGGGGAGCTCCTCCAGTGTTGGCATCATTACTGGGGAATAGCCTACAGAACTATACATTCTCTCACACACTGTTCTGCCAGGATGAGCAACCCGTCCAGGGTCTGTCCGAGGTCTTCGATGGGGACCTGCTCTTCTCCTTCGACTTCTCCAGGAACACTCGTGTACCTCGGCTGCCTGAGTTTGGTGCTTGGGCCAGTGATGAGGGAGATGTCCAGATCATAGAAGATGACAAGAAGCTCTGCCAGCAATTGCAAAAAGTTTTGAGTAGAATTTTGGAAGGCCAAATCCCAGAGGCTAGAGGAAACCCTGTGGCTGAAGTTTTCACTCTGGAGCCCCTGGAGTTTGGGAAGCCCAACACTCTCATCTGCTTTGTTAGTAACATCTTTCCACCTCAGATAACAGTGACTTGGCAGTACAAAGAAGTTCCTGTGGAAAGCAGCAGCCCCACTTTTCTCTCTGCTGTAGACGAACTTGGCTTCCAGGCCTTCTCTTATCTGAATTTCACACCCACTGCCTCTGATATCTTCTCTTGCACTGTGGCACGGGAAGGTGACCTCTTCAGCACTATAGCCTTCTGGGTGCCTCAGAATCCGATACCCTCTGAATTGCTGGAAAATATACTGTGTGGTATTGCCTTTGGCCTGGGAATTCTAGGCATCATAGTAGGTGCTGCCCTCATCATCTACTACCAAAAACCATGTGGAAGTGGTGCAGGTAA

>modo_DNAzoo_DAA_1

ATGGCCCCCAACAAAGCCTGGATCCTAGGGGCCCTCACCCTGGCAGCCCTGCTGAGTCCCTGGGGAGCCAAAGCCATCCAAGAGAATCATGTGATCATCCAAGCAGAATTCGCCCAGACCTCCAACCCCGAAGGAGAGTTCATGTTCGACTTTGATGGGGATGAGATTTTCCATGTGGATATGGATAAAAGGGAAACTGTCTGGCGGCTCACTGACTTCAGCAATTTTGCCAGCTTTGAGGCCCAGGGTGCTCTGGCCAATCTTGCTGTGGACAAAGCCAACCTGGAAATCATGATGAAACGGTCCAACAACACCCCCGACACCAATGTGCCCCCTGAAGTGACAGTCTTTCCAAAGGGTCCAGTGGAGCTGGGTCATCCCAATGTCCTGGTCTGCTTCATTGACAAGTTCTCCCCTCCGGTGCTCAATGTGACATGGCTTCAAAATGGAAAGCCCATCACTGATGGTGTGTTTGAGACTGTCTTCCTCCCCCGCGCTGACCATTCCTTCAGAAAATTCCACTACCTCACCTTCATTCCCTCTGCCACTGATTTCTATGACTGTGAGGTTGACCACTGGGGTTTGGAGCAACCTACTATCAAACACTGGGAACCAGAAGTGCGAACCCCATTGCCAGAGACGACTGAAACTGTGGTCTGTGCCCTGGGCCTGGCTGTAGGCTTGGTGGGCATCATTGCAGGCACCATCTTGTTTATCAAGGCCAAGAGGTCAAGCAATACCTCCCGTGGTGGCCCTCGTGGACCCCTGTAA

>modo_DNAzoo_DBA_1

ATGGCCTCCAGCAGAGTCCTGATTCTAGGGACCCTCTCACTGGTCATGCTGCTCAGTCCCTGGGCAGCTTCTGAGTTCATCAAAGCTGACCATGTGGGGACCTACGGCACAACCATGGCCCAGTCCTACGGGCCTTCAGGCCAGTATACCATGGAGTGTGATGAGGATGAAATTTTCTATGTGGACCAGCAGAAGAAGGAGACTGTGTGGCGGCTGCCAGAATTTAGCCATTTTGCTGGCTATGACCCTCAGGGAGGGCTGAGAAACATAGATGTAATGGAGCACAACCTGGACATCCTGATTAAACGCTCCAATAGAACAAGGGCCATCCCTGTGGCTCCTGAGGTGACTGTGTTCTCTGAGAGTCCTGTGGAGCTGGACCAGCCAAATGTACTCATCTGTTTGGTGGACAACATCTTCCCCCCAGTGGTCAACATCACGTGGCTCCGTAATGGGCAGATAATCACCACGGGTGTGTCTGGGACAGAATTCTACCCTAGATCTGACCACAGATTCCAAAAGTTCTACTACCTCACCTTTCTCCCCAACACAGAAGATGTTTATGACTGTCAAGTGGAGCACTGGGGCCTGGAGCAGCCAGTCCTCAGGCACTGGGaacctcagtttccatctCCACTGCCAGAGACAACAGAAACTGTGGTCTGTGCCCTTGGTCTGGCTGTGGGCCTGGTGGGCATCATCTTGGGCACCATCCTAATTATCAAGAGCATGCGTTCTAGCAGTAGGATTCAACTCCAAGGGCCTCTGTGA

>modo_DNAzoo_DBA_2

ATGGCCGCCGGCAGAATCCTGATCCTAGAGACCCTCTCACTGGTCATGCTGCTCAGTCCCTGGGCAGCTTCTGAGTCCATTGAAGCTGACCATGTGGGCAGATACGGCACAACCATGGCCCAGTCCTACGGGCCTTCAGGCCAGTATACCATGGAGTTTGATGAGGATGAACTTTTCTATGTGGACCAGCAGAAGAAGGAGACTGTGTGGCGGCTGCCAGAATTTAGCCATTTTGCTGGCTTTGATCCTCAGACAGGGCTGAGAGAAATAGCCACAAATGAGCACAACCTGGACTGCCTGATTAAACGCTCCAATAGAACAAAGGCCATCCCTGTGGCTCCTGAGGTGACTGTGTTCCCTGAGAGTCCTGTGGAGCTGGACCAGCCAAATGTACTCATCTGTTTGGTGGACAACATCTTCCCTCCAGTGGTCAACATCACGTGGCTCTGTAATGGGCAGGTGATCACCACGGGTGTGTCTGGGACAGAATTCTACCCTCGATCTGACTACAAATTTCGCAAGTTCTACTACCTCACCTTTCTCCCCAACACAGAAGATGTTTATGACTGCCAAGTGGAGCACTGGGGCCTGGAGCAGCCAATCTTCAGGTACTGGGaacctcagtttccatctCCACTGCCAGAAACAACAGAAACTATGGTCTGTGCCCTTGGTCTGGCTGTGGGTCTGGTGGGCATCATCTTGGGCACCATCCTAATTATCAAGAGCATGTGTTCTAGCAGTAGGATTCAACTCCAAGGGCCTCTGTGA

>modo_DNAzoo_DCA_1

ATGACCTCTAGCCTCGTCTCAATCTTGGGGATCCTCAGCCTGGCTGCCCTGCTGATCAAATGGAGAGTCTGGGCTACGAATGATCACGTAATCAACTCTGTGACATTTGTTCAGACCCACAAACCATCTGGACAGTTCCTGCATGAATTTGATGGGGATGAGCAATTGCACGTGGACCTTGACAGGAAGGAAATAGTCTGGCGGCTTCCTGAGTTTGGCCACATCTTCAGCTTCAGTGCACAGATTGGGTTAGGCAACATTGCTGTGGATATGGCTAACCTGAATCACCTTATCAGGCAAACAAACCACACCCCGGCCACATGGCTCCAGAGGTGACAATATTTCCCAAGGAACCCGTGGAAGCAGAAGAGCCCAATTTGCTCATCTGCCACATTGATAAGTTCTCGCCCCCAGTGATCAATGTCACATGGCTGCGCAATGGACAATCAGTCACCACAGGGATAGATGAGACTGTCTTCCTGCCTCGAGATGACTACTCTTTCCACAAGTTCCATTACCTTACTTTCTTCCCCTCGAATGATGATGTCTATGACTGCGTGGTTGAACATTGGGGCCTGGAAAAGCCACTTTTCAAGCACTGGGAGCCTGAGATGCTAACACCACCATCTGAGATAATGGAGACAATTGTCTGTGTTCTAGGACTGGTCATGGGCCTGGTGGGCATCATTGGGGGTATCCCCCTCATTATCAGAGGCTTGCGCTCAGGCGATCCTGA

>modo_DNAzoo_DMA_1

ATGGGACCTGAGCAAAGCCTGGGGGCTCCACTGTTCCTTCTGCTGCTGCAAGCATCAGTGCTTTTGTCCCTGCCCTGGGAGGACACTCCAGCAGGGCTGACATCGTTCCTGGGGAATAGCCTACACAACTACACATTCTCCCACACATTCTTCTGCCAGAATGAGGCACCCATGGTGAGTCTGTCAGAGGCCTTTGGTGGGGACCAGCTTTTCTCCTTTGACTTCTCCAAGAACTCCCGGGTGCCCCGGCTGCCAGAGTTTGCTCCCTGGGCCGGTGATCAGAGTGACCTCCAGGCCATAGATCGTGACAAGCAACTATGCCAGGAGCTGCAGAAAGCACTGAGTAACTTTTTAGAAGGCCAGATCCCTGAGGCTAGAGGAAACCCTGTGGCTGAAGTTTTCACTCAGGAGCCCCTGGAGTTTGGGAAGCCCAACACCCTCATCTGCTTCATCAGTAATCTCTTCCCACCCAATATCATGGTGACCTGGCAGCATGATGGAGAACCTGTCACGGGCAGCAGCCCTGTTTTTCTCTCAGCTGTAGATGGACTTGGCTTCCAGGCCTTCTCTTACCTGAACTTCACACCTTCATCCACTGACATCTTCACTTGCAGGGTGTCTCGAGAGGGCAACCTCTTTAGCACGATAGCCTTCTGGGtgCCTCAGAATCCAATACCCTCTACATTGTTAGAAAATATATTGTGTGGTATTGCCTTTGGCCTGGGCATCCTGGGCATCATAGTGGGTGTTGCCCTCATCATCTACTTCCGAAAACCATGTGCATGTGGTGCAGGTGA

>Phgy_DNAzoo_DAA_1

ATGGACCCCAACAAAGCCTTCATCTTAGGAGTCTTCAACCTAGCAGTGTTGCTGAGTCCCTGGGGAGCCAGGGCCATTAAAGAGAACCACGTGATCATCCAAGCGGAGTTCTACCAGACCCACAACCCCTTGGGCGAGTTCATGTTCGACTTTGATGGAGATGAGATTTTCCACGTGGATTTGGAGAACAAGCAGACGGTCTGGCGGCTTCCTGACTTTAGCAAATTTGCCAGCTTTGAGGCTCAGGGTGCTCTGGCCAACCTTGCTGTGGATAAAGCCAACCTGGAAATCATGATGAAACGGTCCAACAACACCCCTGACACCAATGTGGCCCCTGAAGTGACGGTGTTTCCAAAGGGCCCAATAGAGCTGGGCCAGCCCAATGTCCTTGTCTGCTTCGTTGACAAGTTCTCTCCTCCGGTACTTAACGTGACGTGGCTTCATAATGGGAATCCCATCACTGATGGTGTGTTTGAGACTGTCTTCCTACCCCGTTCTGACCATGCCTTCAGAAAGTTCCACTACCTCACCTTCATCCCCTCTGCCACCGATTACTATGACTGCAAGGTCGAACACTGGGGACTGGAACAACCTGCTGTCAAACACTGGGAACCAGAAGTACGGACCCCACTGCCAGAGACAACAGAGACTGTGGTCTGTGCCCTAGGCCTGGCCATAGGCCTGGTGGGCATCGTCGTAGGCACCATCCTTATTGTCAAGGGCATGCGATCAAGCAATACTTCCCGTGGTGGCTCCCGTGGACTGTAA

>Phgy_DNAzoo_DBA_1

ATGGCTGTCAATAGAGTCCTGATCCTAGGGACCCTCTTACTGGCTGCGCTGGTGAGTCCCCAAGGAGCTTCTGAGTCCATCGAAGCTGACCATGTTGGGGTCTACGGCACAACTATGTACCAGTTCTATGGCCCCTCAGGCCAGTTTACAGAAGAATTTGATGGGGATGAACTCTTTTATGTGGACCTGGAGAAGAAGGAGACTGTGTGGCGGCTCCCAGAGTTCGGCCATTTTTCTAGCTTTGACCCTCAGGGAGGGCTGAGAAACATAGCCACAGCCAAACACAACCTGGACATCATGATCAAACGCTCCAACAGAAGCAGGATTATCATGCCCCCTGAGGTGACTGTGCTCTCGGAGAGTCCTGTGGAGATGGGCCAGCCAAACATACTCATCTGCTTGGTGGACAACATCTTCCCCCCTGTGGTCAGCATCACATGGCTTCGTAATGGGCAGACGGTCACTGAAGGTGTGTCTGAGACAGACTTTTACCCTCGTTCTGACCAGAAATTCCGCAAGTTCTACTACCTCACTTTTCTCCCGAACACAGAAGACTTTTATGACTGCAAAGTGGAGCACTGGGGCCTGGAGCGGCCACTCCTCAAGCACTGGGAACCCCAGGTTCCATCCCCACTGCCAGAGACAACAGAAACTGTGGTCTGTGCCCTTGGACTGGCTGTGGGTCTGGTGGGCATCATTGTGGGTACCGTCCTCATAATCAGAGGCATGCGTTCCAGCGGCAGGATCCAAGATCAAGGGCCTCTGTGA

>Phgy_DNAzoo_DBA_2

ATGGCTGTCAATAGAATCCTGATCCTAGGGACCCTCTTACTGGCTGCACTGGTGAGTCCCCAAGGAGCTTCTGAGTCCATTGAAGCTGACCATGTTGGGGTCTACGGCACAACTATGTACCAGTTCTATGGCTCCTCAGGCCAGTATACACAAGAATTTGATGGGGATGAGAAGTATTATGTGGACCTAGAGAAGAAGGAGACTGTGTGGAGGGTGCCAGAGTTTGGCCGTTTTGCTAGGTTTGACCCTCAGGGAGGGCTGAGAAACATAGCCACAGTCAAGCACAACCTGGACATCGTGATCAAACGCTCCAACAGAAGCAGGATTATCAGCGTGCCCCCTGAGGTGACTGTGCTCTCGGAGAGTCCTGTGGAGATGGGCCAGCCAAACATACTCATCTGCTTGGTGGACAACATCTTCCCCCCAGTGGTAAACATCACATGGCTTCGTAATGGGCAGACGGTCACTGAAGGTGTGTCTGAGACAGACTTTTACCCTCGGCCTGACCACAAATTCCGCAAGTTCTACTACCTCACTTTTCTCCCGAACACAGAAGACTTTTATGACTGCAAAGTGGAGCACTGGGGCCTGGAGCGGCCACTCCTCAAGCACTGGGAACCCCAGGTTCCATCCCCACTGCCAGAGACAACAGAAACTGTGGTCTGTGCCCTTGGACTGGCTGTGGGTCTGGTGGGCATCATTGTGGGTACCCTCCTCATAATCAGAGGCATGCGTTCCAGTGGCAGCATCCAAGATCAAGGGCCTCTGTGA

>Phgy_DNAzoo_DCA_1_partial_exon_2

TGATAGTGGCCTGGGTGTGGTTGGTTTGCCTGATAAGTTGGTTCAGGTTAGCCATGTCCACAGCAATGTCACCTAGCCCAATCTGGGCATTGAAACTGAAAATGTGGCCAAACTCAGGAAACCATCAGACTGTTTCCTTCCCGTCAAAGTCCATGTGGATCTGCTCATCCTCATCAAATTCATGAAGGTACTGCCAGAAGGTTTGTGGGTTTGAACAAATGTCATGGAGCTCATTACAGGGT

>Phgy_DNAzoo_DMA_1

ATGGGACCTGAGCAAAGCCTGGGAGCTACACTGTTACTCCTGCAGCTGCAGTCATCCCTGTCCTGGGGAGCCACTCCAGCAGCGTTGGCATCATTATTGGGGAATAACGTAGAGAACTACACGTTCTCTCACACGCTGTTCTGCCAGGATACGGAACCCCTCCTGGGTCTGTCCGAGACCTTCAATGGGGACCAGCTCTTCTCCTTCGACTTCTCCAGGAACTCTCGGGTGCCCCGGCTGCCTGAGTTTGCTGCTTGGGCCGGTGATGAGGGAGACATCAAGGCCATAGAAGATGACAAGAAGCTCTGCCAGGAATTGCAAAAAGTTTTGAGTACAATTTTGGATGGCAAAATCCCTGAGGCTAGAGGAAGCCCTGTGCCTGAAGTTTTCACTCTGGAGCCCCTGGAATTTGGGAAGCCCAACACACTCGTCTGCTTTGTTAGTAACATCTTCCCACCTCAAATAACTGTGAGCTGGCAGCACAAAGGAGTCCCTGTGGAAGGCAGCAGCCCCACTTTTCTCTCAGCTATAGATGGACTTGGCTTCCAGGCCTTCTCCTATCTGAACTTCACACCCACATCCTCTGATGTCTTCTCTTGCAATGTGGCACGGGAAGGTGACCTCTTCAGTGCTATAGGCTTCTGGGTGCCTGAGAATCCACTACCCTCTGAATTGTTGGAAAACGTACTGTGTGGTATTGCCTTTGGCCTGGGAGTTGTCGGCATCATAGTGGGTATTGCCCTCATCATCTACTCCCGAAAACCATGTCTAAGTGGTACAGGTGA

>phci_DAA_1

ATGACCCCCAACAAAGCCTTGATCCTAGGAGTCTTCATCCTGGCAGTGCTGTTGAGTCCCTGGGGTGCCAGAGCTGTTAAAGAGAAGCATGTGATCATCCAGGCAGAGTTCTACCAGACCCACGACCCCTTGGGAGAGTTCATGTTTGACTTTGATGGAGATGAGATTTTCCATGTGGATTTGAAGAGCAAAGAGACAGTCtggaggcttcctgacttcagcaaATTTGCCAGTTTTGAGGCTCAGGGTGCTCTGGCCAACCTTGCTGTGGACAAAGCCAACCTGGAAACCATGATGAAACGGTCCAACAACACCCCTGACACCAACGTACCCCCTGAAGTGACAGTGTTTCCCAAGGGCCCAGTGGAGCTGGGCCAGCCCAACGTCCTTGTCTGCTTTGTTGACAAGTTCTCTCCTCCCGTGCTTACTGTGACATGGCTTCATAATGGGCGTCCCATCACTGATGGTGTGTTTGAGACTGTCTTCCTCCCCCGTCCTGACCACTCCTTCAGAAAATTCCACTACCTCACTTTCATCCCCTCTGCCACCGATTACTATGACTGCAAGGTCGAGCACTGGGGACTGGAACAACCTGCTGTCAAACATTGGGAACCAGAAATACGGACCCCACTGCCAGAGACAACAGAGACTGTGGTCTGTGCCCTGGGCCTGGCCATAGGCCTGGTGGGCATCGTCGTAGGCACCATCCTTATTATTAAGGGCATGCGAGCAAGCAACGCTTCCCGTGGTGGCCCTCGTGGAACCCTGTAA

>phci_DBA_1

ATGGCTGCCAACAGAGTCCTGATCCTAGGGACCCTCACACTGACCTCATTGTTGAGTCCCCAAGGAGCTTCCGAATCCATTGAAGCTGACCACGTGGGGGTCCATGGCACACATATGTACCAGTCCTATGGGCCCTCAGGCCAGTATACACACGAATTTGATGGAGATGAGTTGTTTTATGTGGACCTGCAGAAGAAGGAGACTGTATGGCGGCTGCCAGAGTTTAGCCACTTTGCCAGCTTTGACCCTCAGGGAGGGCTGAGAAACATAGACATAGCCAAGCACAACCTGGACATCCTGATCAAACGCTCCAACAGAACCAGGGCCATCATGCCTCCTGAGGTGACTGTGTTCTCAGAGAGTCCCGTGGAGATGGGTCAGCCGAACATACTCATCTGCTTGGTGGACAACATCTTTCCCCCAGTGGTCAACATCACGTGGCTTCGTAATGGACAGTTGGTCACTGTAGGTGTGTCTGAGACAGACTTCTACCCTCGGTCTGACTACAAGTTCCGCAAGTTCCACTACCTCACTTTTCTCCCTAACACAGAAGACTTTTATGACTGCAAAGTGGAGCACTGGGGCCTGGAGCAGCCAGTTCTCAAGCACTGGGagCCCCAGGTTCCATCCCCACTGCCAGAGACAACAGAAACTGTGGTCTGTGCCCTTGGTCTGGCTGTGGGCCTGGTGGGCATCGTTGTGGGCACCATCCTCATAATCAGGGGCATGCGTTCCAGCAGCAGGATCCAACATCAAGGGCCTCTGTGA

>phci_DBA_2

ATGGCTGCCAACAGAGTCCTGATCCTAGGGACCCTCACACTGACCTCATTGTTGAGTCCCCAAGGAGCTTCCGAATCCATTGAAGCTGACCATGTGGGGGTCTACTGCACAGGTGTGTACCAGTCCTATGGGCCCTCAGGCCAGTATACACAAGAATTTGGTGGAGATGAGTTGTTTTATGTGGACCTGCAGAAGAAGGAGACCATGTGGTGGCTGCCAGAATTTAACCACTTTGCTGGCTTTGACCCTCAGGGAGGGCTGAGGGAAAGAGCCACAGTCAAGCACAACCTGGAAATCCTGATCAAACACTCCAATAGAACCAGGGCCATCAAGCCCCCTGAGGTGACTGTATTATCAGAGAGTCCCTTGGAGATGGATGAGCTGAACATACTCATCTGCTTGGTGGACAACAACTTCCCCCCAGTGGTCAACATCACGTGGTTTCGTAATGGGCCGTTTGTCACTGTAGGTGCATCTGAGACAGACTTCTACCCTCGGTCTGACTACAAATTCCACAAGCTCCACTACTTCACTTTTCTCCCAAGCACAGAAGACTTTTATGACTGCAAAGTGGAGCACTGGGGCCTGGAGCAGCCAGTTCTCAAGCACTGGGaTCCCCAGGTTTCATTCCCACTGCCAAAGACAACAGAAACTGTGGTCTGTGCCCTTGGTCTGGCTGTGGGCCTGGTGGGCATCGTTGTGGGCACCATCCTCATAATCAGGGGCATGCATTCCAGCAGTAGGATCCAACATCAAGGACCTCTGTGA

>phci_DBA_3

ATGGCTGCAGATGGAGTCCTGATCTTAGAGATCCTCTCACTGGCCCTACTGCTGAGTCCCCAAGGAGCTTCTGAATCTATTAAAGCTGACCATGTGGGGGTCTACAGCACAACTATGTACCAATCCTATGGGCCCTCAGGCCAGTTTACACAAGAATTTGATGAGGATGAGAAGTTTTAGGTGGACTTGCAGAAGAAGGAGACTGTGTGGCGGCTGCCAGAGTTTAGCTATTTTGCTGGCTTTGACCCACAGGAAGGGCTGAGATGCATAGCCATAGTCAAGCACAACCTGGACATCCTGATCCAATGCTCCAACAGAACCAGGGCCATCATGCCCCTGAGGTGACTGTATTCTCAGAGAGTCCAGTGGAGATAGGCCAGCCCAATGTACTCATCCGCTTGGTGGACAACATCTTCCCTCCAGTGGTCAACATCACGTGGCTTCGTAATGGGCAGTTGGTCACTGTAGGTGTGTCTGAGACAGACTTCTACCCTCAGCCTGACCACAAATTTCACAAGTTCTCCTACCTTGCTTTTCTCCCCAACACAGAAGATTTTTATGACTGCAAAGTGGAGCACTGGGGCCTGGAGCAGCCAGTTCTCAAGCACTGGGagCCCCAGGTTCCATCCCCCCTGCCAGAGACAACAGAAACTGTGGTCTGTGCCCTTGGTCTGGCTATTGGCCTAGTGGGCATCGTTGTGGGCACCATCCTCATAATCAGGGGCATGTGTTCCAGCAGCAGGAGCCAACATTAAGGGCCAGTGTGA

>phci_DCA_1

ATGACTTCCAGTCTAGACTCAGTCTTGGGGATCCTCATCCTGGCTGCCCTGCTGATCAAACAAAGAACCTGGGCCACTAATGACCATGTAATCAGCTCTGTGACATTTGTTCAGACCCACAAACCATCTGGGCAGTACCTGCATGAATTTGATGAGCATGAGCTATTCCATGTGGACTTTGACCAGAAGGAAACAGTCTGGAGGCTTCCTGAGTTTGGGCACATCTTCAGTTTCGATGCACAGATTGGGCTAGGTGACATTGCTGTGGACATGGCTAACTTGAACCAACTTATCAAGCAAACCAACCACACCCAGGCCACCATTGTGACTCCAGAGGTGACAGTGTTTCCCAAGGAGCCCGTGGAACTGGAAGAACCCAACATCCTCATCTGCCACGTTGATAAGTTTTCACCCCCAGTGGTTAATGTCACATGGTTGTGCAATGGAGAACCAGTCACCACAGGGGTATCTGAGACTGCCTTAATGCCTCGGGATGACTATTCTTTCCATAAGTTCCATTATCTCACTTTCCTCCCCTCAGCCAATGATGTCTATGACTGTGTGGTTGAACACTGGAGCCTGGAAAAGCCACTTTTCAAGCATTGGGAGCCTGAGATATTAACACCACCATCTGAGACAATGGAGACACTCCTCTGTATTCTAGGCCTGGCCGTGGGCCTGGTGGGCATCACTGTGGCTGCCTCCCTTATTATCAAAAGCTTGCACTTAGGCGAATGA

>phci_DMA_1

ATGGGACCTGAGCAAAGCCTGGGAGCTATGCTGCTACTGCTGCAGCTACAGTCATCCCTGCTTTTATCCCTGTCCTGGGGAGCCACTCCAGTGTTGGCATCATTATTGGAGAATAGCCTACAGAACTACACGTTCTCTCACACACTGTTCTGCCAGGATGAGGATCCTGTCCTGAGTCTGTCAGAGGCCTTCAATGAAGACCAGCTCTTCTCCTTTGACTTCTCCAGGAACTCCCGGGTGCCCCGGCTGCCTGAGTTTGCTCCTTGGGCCAGTGACAAGGGAGATATCGAGGCCATAAAAGCTGACCAGCGGCTCTGCCAGGAACTGCAAAAAGAATTGAGTAAACTATTGGAAGGCCACATCCCTGAGGCTAGAGGAAACCCTGTGGCTGAAGTTTTCACTCTGGAGCCCCTGGAGTATGGGAAGCCCAACACTCTCATCTGCTTTGTTAGTAATATCTTCCCACCTCAAGTAACTGTGAGCTGGCAGTACCAACAAGTCCCTGTGCAAAGCAGCAGCCCTACTTTTCTCTCAGCTATAGATGGACTTGCCTTCCAGGCCTTCTCTTATCTGAACTTCACACCCACATCCTCTGATGTCTTCTCTTGCACTGTGACCTGGGAAGGTGACCTCTTCAGCACTATAGCCTTCTGGGTGCCTCAGAATCCTATACCCTCTGAATTGTTGGAAAATATATTGTGTGGCATTGCCCTTGGCTTGGGAATTGTTGGCATCATAGTGGGTACTGCGCTCATCATCTACTTCCAAAAACCATGTGCAAGTGGTGCAGGTAA

>Gyle_DNAzoo_DAA_1

ATGACCCCCAACAAAGCCTTGATCCTAGGAACCTTTCTCCTGGCAGTGCTACTGAGTCCCTGGGGAGTCAGGGCCATTAAAGAGAACCATGTGATCATCCAAGCAGAGTTCTACCAGACCAGCAATCCCTTGGGAGAGTTCATGTTTGACTTTGATGGGGATGAGATTTTCCATGTGGATTTGGATAAGAGGGAGACAGTCTggaggcttcctgacttcagcaaATTTGCCAGCTTTGAGGCTCAGGGTGCTTTGGCCAATCTTGCTGTGGACAAAGCCAACCTGGAAATCATGATGAAACGTTCCAAGAACACCACTGACACCAATGTGCCCCCTGAAGTGACAGTGTTTCCCAGGGGCCCAGTGGAGCTGGGACAGCCCAACACTCTGGTCTGCTTCGTTGACAAGTTCTCTCCTCCAGTACTTAATGTGACATGGCTTCATAATGGGAATCCCATCACTGATGGTGTGTTTGAGACTGTCTTCCTTCCCCGGTCTGATCATGCCTTCAGAAAATTCCATTACCTCACCTTCATCCCCTCTGCCACTGATTACTATGACTGCAAGGTTGAGCACTGGGGCCTGGAACAGCATGTTGTCAAACACTGGGAACCAGAAGTACGGACCCCACTGCCAGAGACAACAGAGACTGTGGTCTGTGCCCTAGGCCTGGCTATAGGCCTGGTGGGCATCATCATAGGCACCATCCTTATTATCAAGGGCATGCGATCAAACAACACTTCCCATGGTGGCTCCCGTGGACCCCTGTAA

>Gyle_DNAzoo_DBA_1

ATGGCTACCAACAGAGTCCTGATCCTAGTGACCCTCTCACTGGCTTTGCTGCTGAGTCCCCAAGGAGCTTCTGAATCCATCGAAGCTGACCATGTTGGGGTCTACGGTACAACTATATACCAGTCCTATGGGCCTTCAGGCCAGTATACACAAGAATTTGATGGTGATGAGTTGTTTTATGTGGACCTGCAGAAGAAGGAGACTGTGTGGCGGCTGCCAGAGTTTAGCCATTTTGCTGGCTTTGACCCTCAGGGAGGGCTGAGAAACATAGCCACAGTGAAGCACAACCTGGACATCATGATCAAACGCTCCAACAGAAGCAGGGCCATCATGCCGCCTGAGGTGACTGTGTTCTCAGAGAGTCCTGTGGAGATGGGCCAGCCGAATGTACTCATCTGCTTGGTGGACAACATCTTCCCCGCAGTGGTCAGTGTCACCTGGCTCCGTAATGGTCAGTTGGTCACTGGAGGTGTGTCTGAGACAGACTTTTACCCTAGGCCTGACCACAAATTCCGCAAGTTCTACTACCTCACTTTTCTCCCCAACACAGAAGACTTTTATGACTGCAAAGTGGAGCACTGGGGCCTGGAGCAGCCAGTCCtcaagcactgggAACCCCAGGTTTCATCCCCACTGCCAGAGACAACAGAAACTGTGGTCTGTGCCCTTGGTCTGGCTGTGGGCCTGGTGGGCATCATGGTGGGCACCATCCTCATAATCAGAGGCATGCATTCCAGCACTAGGATCCAACACCAAGGGCCCCTGTGA

>Gyle_DNAzoo_DBA_2

ATGGCTGCCAACAGAGTCCTGGTCCTAGGGACCCTCTCACTGCTGCTGAGTCCCCAAGGAGCTTCTGAATCCACTGAAACTGACCATGTTGGGGTCTATGGTACAACTATATACCAGTCCTATGGGCCCTCAGgccagtatacatttgaatttgatGAGGAGGAGATATTTTCTGTGGACCTGAAGAAGAAGGAGACTGTGTGCCGGCTGCTAGAGTTTAGCCATTTTGCTGGCTTTGACCCTCAGGGAAGACTGACAGCCACATCGAAGCACAACCTGGTTGTCATGATCAAAGACTCCAACAGAAGCAGGGCCATCATGCCTCCTGAGGTGACTGTGTTCTCAGAGAGTCCTGTGGAGATGGGCCAGCCAAATGTACTCATCTGCTTGGTGGACAACATCTTCCCCACAGTGGTCAACATCACCTGGCTTCATAATGGCCAGTTGGTCACTGGAGGTGTGTCTGAGACAGACTTCTACTCTCAGCCTGACCACAAATTCTGCAAGTTCTACTACCTCACTTTTCTCCCCAACACAGAAGACTTTTATGACTGCAAAGTGGAACACTGGGGCCTGGAGCAGCCAGTCCtcaagcactgggaacCCCAGGTTCCATCCCCACTACCAGAGACAACAGAAACTGTGGTCTGTGCCCTTGGTCTGGCTGTGGGCCTGGTGGGCATCGTGGTGGGCACCATCCTCATAATCAGAGGCATGCATTCCAGCACTAGGATCCAACACCAAGGGCTCCTGTGA

>Gyle_DNAzoo_DBA_3

ATGGCTACCAACAGAGTCCTGATCCTAGGGACCCTCTCACTGGCTTTGCTGCTGAGTCCCCAAGGAGCTTCTGAATCCATTGAAGCTGACCATGTTGGGATCTATGGTGAAGGTATGGGCCAGTCCTATGGGCCCTCAGGCCAGTATACACAAGAATTTGATGGGGACGAGGAGTTTTATGTGGACCTGCAGAAGAAGGAGACTGTGTGGCGGCTTCCAGAGTTTAGCCATTTTGCTGGCTTTGACCCTCAGGGAGGGCTGACAGAGATAGCCACAGTGAAGCACAACCTGGACGTCATGATCAAACGCTCCAACAGAAGCAGGGCCATCATGCCGCCTGAGGTGACTGTGTTCTCAGAGAGTCCTGTGGAGATGGGCCAGCCGAACGTACTCATCTGCTTGGTGGACAATATCTTCCCCCCAGTGGTCAACATCACCTGGCTTCATAATGGCCAGTTGGTCACTGGAGGTGTGTCTGAGACAGACTTCTACTCTCGGCCTGACCACAAATTCCGCAAGTTCTACTACCTCACTTTTCTCCCCAACACGGAAGACTTTTATGACTGCAAAGTGGAACACTGGGGCCTAGAGCAGCCAGTCCtcaagcactgggAACCCCAGATTCCATCCCCACTGCCAGAGACAACGGAAACTGTGGTCTGTGCCCTTGGTCTTGCTGTGGGTCTGGTGGGCATCGTGGTGGGCACCATCCTCATAATCAGAGGCATGCGTTCCAGCACTAGGATCCAACACCAAGGGCCCCTATGA

>Gyle_DNAzoo_DCA_1

ATGACCTCAAGTCTACACTCAATCTTGGGGAATCCTCAGCCTGACTGCCCTgctgatcaaacaaagaacctggGCCACCAATGACCATGTAATCAGCTAGGTGACATTTGTTCAGACCCATGAACCATTTGGGCAGTACCTGCATGAATTTGATGAGAATGAACTATTCCATGTGGACTATGACAAGGTGGAAACAGTCTTGTGGCTTCCTGAATTTGGCCACATCTTCAATTTTGATGCACAGATTGGGCTAGGCAACATTGCTGTGGACATGGCTAACTTGAACCAACTTATCAGGCAAACCAGTCACACCCAGGTCACCACTGTGACTCCAGATGTGACAGTGTTTCCCAGGAGCCTGTGGGGCTAGAAGAACCCAATATCCTCACTTGCCACATTGATAAGTTCTCACCCCCAGTGATCAATGTCACATGGCTGTGCAATGGAGAACCAGTCACCACAGGGGTATCTGAGACCATCTTCCTGCTTCAGGATGACTATTCTTTCCACAAGTTCTATTACCTCACTTTTCTCCCCTCAACTGATGATGTCTATGACCATGTAGTTGAACACTGGggcctggaaaagtcacttttcaAGCATCGGGAGCCTGAGAATCTAACACCACCATCTGAGACAATGAAGATGCTCATCTGTATTCTAGGACTGGCTGTGGGCCTGGTGGGTGTCACTGTAGTTGCCACCCTTATTATCAAAGGCTTGTGCTCAAGCAAATGA

>Gyle_DNAzoo_DMA_1

ATGGGACCTGTGCAAATTCTAGGAGCTACACTGTTCCTACTGCAGCTGCAGGCATCACTGTTTTTGTCTGTGTCCTGGGGAGCCACTCCAGCAGTGTTGGCATTATTATTGGGGAATAACCTACAGAACTACACATTATCTCACACACTGTTCTGCCAGGATGAGGAACCCCTCCTGGGTCTATCGGAGATGTTTAATGGGGACCAGCTCTTCTCCTTCGACTTCTCCAAGAATGCTCTGGTGCCCCGCCTGCCTGAGTTTGCTGCTTGGACCGGTGATAAAGAAGACATTCAGACCACAAAAAACGATGGGGACTTCTGCAAGGAATTGCAGATAGCTTTGAGTAGGATTTTGGAAAACAAAATTCCTGAGGCTAGAGGAAACCCTGTGGCTGAAATTTTCACCCTGGAGCCCCTGGAGTTTGGGAAGCCCAACACTCTCATCTGCTATGTCAGTAACATCTTCCCACCTCAGATAACTGTGACCTGGCAGTACAAAAATGTCCCCGTGGAAAGCAGCAGCCCCACTTTCCTCTCAGCTGTGGATGGACTTGGCTTCCAGGCCTTCTCTTATCTGAACTTCACACCCACATCCTCTGATGTGTTCTCCTGCACTGTGGAAAGGGAAGGTGACATCTTTAGCACTATCACCTACTGGGTGCCTCAGGATCCGATACCCTCTGAACTGTTGGAAAATGTACTGTGTGGTATTGCCTTTGGCCTGGGAATTGCTGGCATCATAGTGGGTGCTGCCCTCATCATCTACTTCCGAAAACCATGTGCAAGTGGTGCAGGTGA

>Pegr_tran_DAA_1

ATGGCCCCAAACAAAGCCTTGATCCTAGGAGCCTTCCTCCTGGCAGTGCTGCTGAGTCCCTGGGGAGCCAGGGCCATTAAAGAGAACCATGTGATCATCCAAGCAGAGTTCTATCAGACCAGCAAACCCTTGGGAGAGTTCATGTTCGACTTTGATGGGGATGAGATTTTCCATGTGGATTTGGAAAAGAGCGAGACAGTCTGGAGGCTTCCTGACTTCAGCAAATTTGCCAGCTTTGAGGCTCAGGGTGCTTTGGCCAATCTTGCTGTGGACAAAGCCAACCTGGAAATCATGATGAAACGGTCCAAGAACACCACTGATACCAATGTGCCCCCTGAAGTGACAGTGTTTCCCAAGGTCCCAGTGGAGCTGGGACAGCCCAACATCCTGGTCTGCTTCGTTGACAAGTTCTCTCCTCCAGTACTTAATGTGACATGGCTTCATAATGGGAATCCTATCACTGAGGGTGTGTTTGAGACTGTCTTCCTTCCCCGCTCTGATCATGCCTTCAGAAAATTCCATTACCTCACCTTCATCCCCTCTGCCACCGATTACTATGACTGCAAGGTTGAGCACTGGGGACTGGAACAACCTGTTGCCAAACACTGGGAACCAGAAGTACGGACCCCACTGCCAGAGATAACAGAGACTGTGGTCTGTGCCCTAGGCCTGGCCATAGGCCTGGTGGGCATCATTGTCGGCACCATCCTTATTATCAAGGGCATGCGATCAAACAACACTTCCCGTGGTGGCTCCCGTGGACCCCTGTGA

>Pegr_tran_DBA_1

TGGCTGCCAACCGAGTCCTGATCCTAGGGACCCTCTCACTGGTTTTGCTGCTGAGTCCCCAAGGAGCCTCTGAATCCATCAAAGCTGACCATGTAGGGGTCTACGGTACAACTATATCCCAGTCCTATGGACCCTCAGGCCAGTATACACAAGAATTTGATGAGGATGAACTGTTTTATGTGGACCTGCAGAAGAAGGAGACTGTGTGGCGGCTACCAGAATTTAGCCATTTTTCTGGCTTTGACCCTCAGGGAGGGCTGAGAAACATAGCCACAGCAAAGTACAACCTGGACATCCTGATCAAACGCTCCAACGGAAGCAGGGCCATCAGTGTGGCCCCTGAGGTGACTGTGTTCTCAGAGAATCCTGTGGAGATGGGCCAGCCGAACGTACTCATCTGCTTGGTGGACAGCATCTTCCCCCCAGTGGTCAACATCACCTGGCTTCGTAATGGGCAGTTGGTCACTGGAGGTGTGTCTAAGACAGACTTCTACCCTCGGCCTGACCACAAATTCCGCAAGTTCTACTACCTCACTTTTCTCCCCAACACAGAAGACTTTTATGACTGCAAAGTGGAACACTGGGGCCTGGAGCAGCCAGTCCTCAAGCACTGGGAACCCCAGGTTCCATCCCCACTGCCAGAGACAACAGAAACTGTGGTCTGTGCCCTTGGTCTGGCTGTGGGCCTGGTGGGCATCGTTGTGGGCACCATCCTCATAATCAGAGGCATGCGTTCCAGCACTAGGATCCAACACCAAGGGCCCCTGTG

>Pegr_tran_DMA_1

ATGGGGCCTGTGCAAATCCTAGGAGCTACACTGTTACTCCTGCAGCTGCAGTCATCACTGTTTTTGTCTCTGTCCTGGGAAGCCACTCCAGTACTGGCATCATTATTGGGGAATAGCCCACAGAACTACACATTCTCTCACACACTGTTCTGCCAGGATGAGGAACCCCTCCTGGGTCTATCGGAGAACTTCAATGGGGACCAGCTCTTCTCCTTCGACTTCTCCAAGAAAGCTCTGGTGCCCCGCCTACCGGAGTTTGCTGCTTGGACCGGTGATAAAGAAGACATCAAGACCACAGAAAGTGATGGGAAGCTCTGCAAGGAATTGCAAAATGCTTTGAGTAGAATTTTGGAAGACCAAATCCCTGAGGCTAGAGGAAACCCTGTGGCTGAAATTTTCACCCTGGAGCCCCTGGAGTTTGGGAAGCCCAACACTCTCATCTGCTTTGTTAGTAACATCTTCCCACCTCAAATAACTGTGACCTGGCAGTACAAAAAAGTGTCTGTGGAAAGCAGCAGCCCCACTTTCCTCTCAGCTGTGGATGGACTTGGCTTCCAGGCCTTCTCTTATCTGAACTTCACACCCACATCATCTGATGTCTTCTCTTGCACTGTGGAACGGGAAGGTGACATCTTCAGTACTATCACCTACTGGGTGCCTGAGGATCCGATACCCTCTGAATTGTTGGAAAATGTACTGTGTGGTATTGCCTTTGGCCTGGGAATTGCTGGCATCATAGTGGGTGCTGCCCTAATCATCTACTTCCGAAAACCATGTGCAAGTGGTGCAGACTGA

>Lahi_DAA_1

ATGACCTCCAACAAATCCTTGATCCTAGGAGCCTTCATTCTCTCAGTGATGCTGAGTCCCTGGGGAGCCAGGGCCATTAAAGTTATGTTTGGTGCCATACAGAGAACCATGTGATCATCCAAGCAGAGTTCTACCAGACCAACGAACCCTCTGGAGAGTTTATGTTCGACTTTGATGGGGATGAGATTTTCCATGTGGATTTGAAGAAGAGAGAGACAGTCTGGCGGCTTCCTGACTTCAGCAAATTTGCCAGCTTTGAGGCTCAGGGTGCCTTGGCCAATCTTGCTGTGGACAAAGCCAACCTGGAAATCATGATGAAACGATCCAATAACACCCCTGACACCAATGTGGGCCCTGAAGTGACTGTGTTTCCCAAAGGCCCAGTGGAACTGGGCCAGCCCAACATCCTTGTCTGCTTTGTTGACAAGTTCTCTCCTCCAGTACTTACTGTGACCTGGCTTCATAATGGGGTTCCCATCACTGATGGTGTGTTTGAAACTGTCTTCCTCCCTCGCTTTGACCATGCCTTCAGAAAATTCCACTACCTCACCTTCATCCCCTCTGCCACCGATTACTATGACTGTAAGGTTGAGCACTGGGGACTGGAACAACCTGTTGTCAAACACTGGGAACCAGAAGTACGGACCCCACTGCCAGAGACAACAGAGACTGTGGTCTGTGCCCTAGGCCTGGCCATAGGCCTTGTGGGCATCATCGTAGGCACCATTCTTATTATCAAGGGCATGAGATCAAACAACACTTCCCGTGGTGGCTCCCGTGGACCCTTGTAA

>Lahi_DBA_1_partial_exon_2-4

CTGACCATGTTGGGGTCTACGGCACAGATATATGCCAGAATTATGGGCCCTCAGGCCAGTACACACAAGAATTTGATGATGATGAGATGTTTTATGTGGACCTGCAGAAGGAGATTGTATGGTGGCTGCCAGAGTTCAGCCATTTTGCTGGCTTTGACCCTCAGGGAGGGTTAAGAAACATACGCATATCCAAGCTCACCATGGACACCCTCATCAAACACTCCAACAGAAGCAGGGCCATCAGGGTGCCCCCTGAGGTGACTGTATTCTCAGAGGATACTGTGGAGATAGGCCAGCCAAATGTACTCATCTGCTTGGTAGACAACATCTTCCCCTCAGCTGTCAACATCATGCGGCTTCATAATGACCAGTTGGTCACCACTGCTGTGTCTGAGACAGACTTCTACTCTCGGCCTGACCATGAATTCCACAAGTTCTACTACCTCACTTTTCTCCCCAACACAGAGGATTTTTATGACTGCAAAGTGGAGCACTGGGGCTTGGAACAGCCAGTCCTCAAGCACTGGGAACCCCAGATTCCATCCCCAGAGCCAGAGACAACAGAAACTGTGGTCTGTGCCCTTGGTCTGGCAGTGGGCCTGGTGGGCATCATTGTGGGTACCATCCTTATAATCAGAGGCATGAGTTCCAGCAATAGGTTCCAACATCAAGGGCCTCTGTGA

>Lahi_DBA_2_partial_exon_2-4

CTGACCACGTGGGGATCTACGGCACAGGTTTATACCAGTCCTATGAGTCCTCAGGACAGTACACACAGGAATTTGATGAGGATGAGCTGTTTTATGTAGATCTGCAGAAGAAGGAGACTGTGTGGCGGCTGCCAGAGTTTAGCCATTTTAGCAGCTTTGACCCTCAGGGAGGGCTGCGTGAAAAAGCCACATGCAAGTACAACCTGGACATCCTGATCAAACGCTCCAACAGAAGCAGGGCCATCAGTGTGCCCCCTGAAGTGACTGTGTTCTCAGAGAGTCCTGTGGAGTTGGGCCAGCCAAATGTACTCATCTGCTTGGTGGACAACATCTTCCCTCCAGTGGTCAACATCAAGTGGCTTCGTAATGGCCAGGTGATCACCACTGGTGTGTCTGAGACAGACTTCTACTCTCGGCCTGACCACAAATTCCGCAAGTTCTACTACCTCACTTTTCTCCCCAACACAGAGGACTTTTATGACTGCAAAGTGGAGCACTGGGGCTTGGAGCAGCCACTCCTCAAGCACTGGGAACCCCAGATTCCATCCCCAGTGCCAGAGACAACAGAAACTGTGGTCTGTGCCCTTGGTCTGGCAGTGGGCCTGGTGGGCATCATTGTGGGCACCATCCTTATAATCAGAGGCATGCGTTCCAGCAATAGGTTCCAACATCAAGGACCTCTGTGA

>Lahi_DBA_3_partial_exon_2-4

CTGACCACGTGGGGATCTACGGCACAGGTGTATACCAGTCCTATGAGTCCTCAGGCCAGTACACACAGGAATTTGATGAGGACGAGCTGTTTTATGTAGATCTGCAGAAGAAGGAGACTGTCTGGCGGCTGCCAGAGTTTAGCCATTTTAGCAGCTTTGACCCTCAGGGAGGGCTGCGTGAAAAAGCCACATGCAAGTACAACCTGGACATCCTGATCAAACGCTCCAACAGAAGCAGGGCCATCAGTGTGCCCCCTGAAGTGACTGTGTTCTCAGAGAGTCCTGTGGAGTTGGGCCAGCCAAATGTACTCATCTGCTTGGTGGACAACATCTTCCCTCCAGTGGTCAACATCAAGTGGCTTCGTAATGGCCAGGTGATCACCACTGGTGTGTCTGAGACAGACTTCTACTCTCGGCCTGACCACAAATTCCGCAAGTTCTACTACCTCACTTTTCTCCCCAACACAGAGGACTTTTATGACTGCAAAGTGGAGCACTGGGGCTTGGAGCAGCCACTCCTCAAGCACTGGGAACCCCAGATTCCATCCCCAGTGCCAGAGACAACAGAAACTGTGGTCTGTGCCCTTGGTCTGGCAGTGGGCCTGGTGGGCATCATTGTGGGCACCATCCTTATAATCAGAGGCATGCGTTCCAGCAATAGGTTCCAACATCAAGGTCCTCTGTGA

>Lahi_DBA_4

ATGGTTGTCCACAGAGTCCTGATCCTAGGGACCCTCTCACTGACTATGCTTCCCAAGGAACTTCTGAGTCCATTGAAGATGACCATGTTGGGGTCTACAGCACAGGTATGTACCAGTCCTATGGGCCTTCAGGCCAGTATACACAGGAATTTGATGAGGACGAGCTATTTTATGTGGACCTGCAGAAGAAGGAGACTGTGGTGGCAGCCAGAGTTTAGCCATTTTGCTGACTTTCACCCACAGGGAGGACTGAGAAACATAGCTGTCATGCAGCACAGCCTGGACATCATGATGAAATGCTCCAACAGAAGCAGGGCCATCGGTGTGCCCCCTGAGGTGACTGCGTTCTCAGAGAATCCCGTGGAGATGGGCCAGCTGAACATATTCATCTACTTGGTGGACAATATCTTCCCTCCAGTGCTTAACATCACATGGCTTCATAATGGCCAGTTGATCACCTCTGGTGTGTCTAAGAAAGACTTCTATTCTTGGCCTGACCACAAATTTCTCAAGTTCTACTACCTCACCTTTCTCCCCAAAACACAGGATTTTTACGACTGCAGAGTGGAACACTGGGGCCTGGAGCAGCCAGTCCTCAAGCGCTGGATGCCAGAGACAGCAGAAACTCTGGTCTGTGCCCTTGGACTGGCTGTGGGCTTGGTGGGCATCATTGTGGGCACCATCCTCATAATCAGAGGCATGTGTTCCAGCAGGAGGATCCAACATCAAGGGCTTCTGTGA

>Lahi_DCA_1_partial_exon_2-4

ACCATGTAATCAGCTCTGTGACAATTGTTCAGACCTACAAACCATCTGAGCAGTACCTGCATGAATTTGATGAAGATGAACCATTTCACATGGACTATGAAAAGAAGGAAACAGTCTGGCAGCTTCCTGAGTTTGGCCATATCTTCAGTTCCAGTGCACAGATTGGGCTAGGTGACATTGCTGCGGACATGGCTAACTTGAACCAACTTATCAGGCAAACCAACCACACCCAAGCCACCATTGTGACTCCAGAGGTGGCAATGTTTCCCAAGGAGGCTGTGGAACTAGAAGAACCCAGCGTCCTCCTTTACCACATTGATAAGTTCTCCATCCCAGTGATCAATGTCATATGGCTGTGCAATGGTGAGTCAGTCACCACAGCAGTATCTGAGACTGCCTTCCTGCCTCAGGATGACTGTTCTTCCCACAAGTTTCATTACTTCACTTTCTTCCTCTCAACTGATGATATTTATGACTGCGTAGTTGAACCCTGGGGGCTCAAAAACCACTTTTCAAAGCCTGAGATGCTAAGACCACCATCTGAGACAATGGAGATGCTCATCCCTATTCTAGGAATGGCTATGGGCCTCTTGGGCATCATGGTGACTGCCAGCTTCATTATCAGAGGCTTGTGCTCAGGCAAATGGTTCCTGA

>Lahi_DMA_1

ATGGGACCTGTGCAAAGCCTGGGAGCTACACTGTTACTCCTGCAGCTGAAGTCATCACTGTTCTTGTCTCTGTCCTGGGGAGCCACTCCAGCAGTGTTGGCATCATTACTGGGGAATAGCCTACAGAACTATACATTCTCTCACACACTGTTCTGCCAGGACGAGCAACCCTTAGTGGGTCTGTCTGAGGTCTTCGATGGAGACTTGCTCTTCTCCTTCGACTTCTCCAGGAACACTCGGGTACCCCGGCTGCCTGAGTTTGGTGGTTGGGCCAGTGATGAGGGAGATGTCAAGATCATAGAAGATGACAAGATACTCTGCCAGGATTTGCAGAAAGCTTTGAGTAGAATTTTGGAAGGCCAAATCCCAGAGGCTAGAGGAAACCCTGTGGCTGAAGTTTTCACTCTGGAACCCCTGGAGTTTGGGAAGCCCAACACTCTCATCTGCTTTGTTAGTAACATCTTCCCTCCTCAGATAACAGTGACTTGGCAGTACAAAGAAGTTCCTGTGGAAAGCAGCAGCCCCACTTTTCTCTCTGCTATAGACGGACTTGACTTCCAGGCCTTCTCCTATCTGAATTTCACACCCACTCCCTCTGATATCTTCTCTTGCACTGTGGCACGGGAAGGCGACCTCTTCAGCACTATAGCCTTCTGGGTGCCTCAGAATCCGATACCCTCTGAATTACTGGAAAATATACTGTGCGGTATTGCCTTTGGCCTGGGAATTCTAGGCATCATAGTGGGTGCTGCCCTTATCATCTACTTCCAAAAACCATGTGGAAGTGGTGCAGGTAA

>Noty_DNAzoo_DAA_1

ATGTCCCCCAACAAAGCCTTGATCCTAGAGGTTTTCATGCTGACAGTGCTGCTGAGTCCCAGGGGAACCAAGGCCATTAAAGAGAATCATGTGATCATCCAAGCAGAGTTCTACCAGAACCATGACCCCTCAGGAGAGTTCATGTTTGACTTTGATGGGGATGAGATTTTCCATGTGGATTTGAAGAAGAAGGAGACAGTCTGGCGGCTTCCTGACTTTAGCAAATTTGCCAGCTTTGAGGCTCAGGGTGCTCTGGCCAATCTTGCTGTGGACAAAGCCAACCTGGAAATAATGAAGAAATGGTCTAACAACACCCCTGACACCAATGTGTCCCCTGAGGTGACAGTGTTTCCCAAGGGCCCAGTGGAGCTGGGCCAGCCCAACATCCTTGTCTGTTTCATTGACAAGTTCTCTCCTCCTGTGCTTAACGTGACATGGCTTCATAATCAAGAACCCATCACTGATGGTACATTTGAGACTGTCTTCCTCCCCCGCCCTGACCATTCCTTCAGAAAATTCTACTACCTCGCCTTTATCCCCTCTGCCAACGATTTCTATGACTGTAAGGTCGATCACTGGGGACTGGAACAACCTGCTATCAAACACTGGGAACCAGAAGTACGGACCCCACTTCCAGAGACAACAGAGACTGTGGTCTGTGCCCTGGGCCTGGCCATAGGCCTGGTGGGCATCATCATAGGCACCATCCTGATAATCAAGGGCATGCGATCAAGTAGCACTTCACGTGGTGGCCCCCGTGGACCCCTGTAA

>Noty_DNAzoo_DBA_1

ATGGCCACCAAGAGAGTCCTGATCCTAGGAACCCACTCAGTGGCCATGATGCTGAATCTCCAAGGAGCTTCCCAGTCCATTGAAGCTGACCACACAGGGACCTATGTTATAACTATGTACCAGACCTATGGGTCCTCAGGCCAGTATGCACACGAATTTGATGGGGATGAAGAGTTTTATGTGGACCTGAAGAAGAAAAAGAGTATATGCCGGCTGCCAGAGTTTGCCCATTTAATTTAACCCTGGGGCTACCCTAAAGAAGAACAACCTGGAACTCATGATAAAATGTTCCAACAGAACCAGAGCTATCTATGCCCCCTGAGGTGATTCTCAGAGAGTCCCGTGGAGGTAGGCCAGCCCAATATACTCATCTGTTTAGTGGACAGCATCTTCCCCACAGTGATCAACATCACTTGGCTTCGTAATGGGAAGTTGGTCACTGTGGGTGTGTCTGAGACAGAATTCTATTCTCGGCATGACCACAAATTCCGCAAGTTCTACTACCTCGCTTTTCTCCCCAACACAGGTGATTTTTATGACTGCAAAGTGGCGCACTGGGGCCTGGAGCTTTGGTACTGAACCTCAGGCTGCATCCCCACTGACAGAGACAACAGAAACTGTGGTTTGTGCCCTTGGTCTGGCTGTGGCCTTAGTGGGCATTATTATGGGCACCATCCTCCTAATTAGAGGCATGCATTCCAACATTAGGCTCCAACACCAAGAGGCTCTATGA

>Noty_DNAzoo_DBA_2

ATGACCATCAACAGATTCTTGATCCTAAAGACCCTCTCACTGGCTGTGCTGCTGAGTCCTGAAGGAGCTTCTGAGTCCATTGAAGCTGACCACGTGGGTGTCTATGGCACAACTGTATACCAGTCCTATGGGTCCTCAGGCCAGTACACAATGGAATTTGATGAGGATGAGGAACTTTATGTGGACCTGAAGAAGAAGGAGACAGTATGGCAGCTGCCAGAGTTTAGCCATTTTGTCAGCTTTGACCCTCAAGGAGGGCTGAGAAACATAGCCACAATGAAGTACAACCTGGACATCCTGATCAAACGCTCCAACAGAACCAGGGCCATCAATGTGCCTCCCGAGGTGACTGTGTTCTCCAAGAGTCCTGTGGAGATAGGCCAGCCAAATGTACTCATCTGCTTGGTGGACAATATCTTCCCCCCAGTGGTCAATATCACGTGGCTTCGTAATGGGCAGTTGGTCACCATAGGTGTGTCTGAGACAGATTTCTACCCTCAGCTTGACTGCAAATTCCACAAGTTCTACTATCTCACTTTTCTCCCCAACACGGAAGATTATTATGACTGCAAAGTGGAGCACTGGGGCTTGGAGCAGCCAGTCCTCAAGCACTGGGAACCCCAGATTCCATCCCCACTACCAGAGACAACAGAAACTGTGGTCTGTGCCCTTGGTCTGGCTGTGGGCCTGGTGGGCATCATTGTGGGCACCATCCTCATAATCAGAGGCATGCATTCCAGCAGTAGGATCCAACGCCAAGGACCTCTGTGA

>Noty_DNAzoo_DCA_1_partial_exon_2-4

CTCTGTGACATTTGTTCAAACTCACAAACCATCTGGGCAGTACCTACCTGCTTGAACTTGATGGGGATGAGCAATTCCACATGAACCTTGACAGGAAGGAAATAGCCTGGCAGCTTCTCAAATTTGGCCAATTCTTCAGTTTTCATGCAAAGCTAGCTAACTTAAACCACCTTATCAGGCAAACCATCCAGCACTAGGCCACCATCATGCCTCTGGAGGTGAGAATGTTTCCCAAGGAGCCTGTGGGACTAAAGGAGTCGAACATTTTCATCTGTTACATTGATAATTTCTCACCCCTAGCGATTAGTATCATATGACTGTGCAATGGACAGCCTGTCACCAGGGGGCTATCTGAGGCTGCCTTCCTGCCTCAGGGTGACTATTCTTTCCACAAGTTCCATTACCTCACTTTCCATCCCTCAACTGATGATGTCTATGACTGTGTTTGAACGCTGGGGCCTAGAAAAGCCACTTTTCAAGCACTAGGATAGATGCTCATCTGTATCCTAGGACTAGCTGTGGGCCTGGTGGGAATCACTGTGGCTGCCACCTTCATTATAGAGGCTGGTGCCCAGGCACATGATGCCCGACCCCAAGATGCTGCAAGTCTTACCTCCTGCAAGAAATCTTTACCAATCTTTAATCTTAGTGA

>Noty_DNAzoo_DMA_1

ATGGGACCTGAGCAGAGCCCAGGAGCTACACTGTTACTACTGCAGCTTCAGTCTTCACTGCTTTTGTTCCTCTCCTGGGAAGCCACTCCAGTGTTGGCATCATTATTGGAGAATAGCCTGCAAAACTACACATTTTCTCACACACTCTTCTGCCAGAGTAAGAAACCCTATGTGGGTCTGTCTGAGTCCTTCAACGGAGACCAGCTCTTCTCCTATGACTTCACCAACCACACCCGGGTGCCTCGGCTGCCTGAGTTTGCTGCTTGGGCCAGGGATAAGGACGACATCAAGGCCATAAAAGCTGACAAGGAGCTCTGCCAGACATTGCAACAGGCCTTGAGTGCATTTTTAAAAGACAAGATCCCCCAGGCTAGAGGAAACCCGGTGGCTGAAGTTTTCACTCTGGAGCCCCTGGAGTTTGGGAAGGCCAACACTCTCATCTGCTTTGTTAGTAACATCTTCCCACCTCAAATAACCGTGACCTGGCAGAAAGGCAAAGTCTCTGTGGAAAGCAGCAGCCCTACTTTTCTCTCAGCTATAGATGGACTTGGCTTCCAGGCCTTCTCTTATCTGAACTTCACACCCTCATTCTCTGATGTCTTCTCTTGCACTGTGGCAAGGGAAGATGAATTCAGCACTATAGCCTTCTGGGTGCCTCATGATCCAATACCTTCTGCATTGTTGGAAAACATACTGTGTGGTATTGCCTTTGGCCTGGGCATTGTTGGTATCATAGTGGGTGCTGCCCTCATCATCTATTTTCGAAAACCATGTGCAAGTGGTGCAGGTGA

>Drgl_DAA_1

ATGGCCCCCAACAAAGACTTGATCCTATGGACCTTCACCCTGGCAGTCCTGCTGAGTCCCTGGGGAGCCAGGGCTATTAAAGAGAACCACGTGATCATCCAAGCAGAGTTCTACCAGACCCATGAGCCTTCAGGAGAGTTCATGTTCGATTTTGATGGGGATGAGATTTTCCATGTGAATTTGGAAAAGAAGGAGACATTCTggaggcttcctgacttcagcaaATTTGCCAGTTTTGAAGCTCAGGGTGCTCTGGCCAATCTTGCTGTGGACAAAGCCAACCTGGAAATCATGATGAAACGGTCCAACAATACCCCTGACACCAATGTGCCCCCTGAAGTGACTGTGTTTCCCAAGGGCCCAGTGGAGCTGGGCCAGCCCAACGTCCTTGTCTGCTTCATTGACAAATTCTCTCCTCCTGTACTTAATGTGACATGGCTTCATAATGGGCGTCCCATCACTGATGGTGTGTTCGAGACTGTCTTCCTCCCTCGCTCTGACCATGCCTTCAGAAAATTCCACTACCTCACCTTTATCCCCTCTGCCACTGATTACTATGACTGTGAGGTTGATCACTGGGGACTGGAGCAACCTGTTATCAAACACTGGGAACCAGAAGTACGGACTCCACTGCCAGAGACAACAGAGACCGTGGTCTGTGCCCTGGGCCTGGCCATTGGCCTGGTGGGCATCATCGCAGGCACCATCCTGATTATCAAGGGCATGCGATCAAGCAACACTTCCCGTGGTGGCCCCCGTGGACCCCTGTAA

>Drgl_DBA_1

ATGGCCACCAGCAGAGTCCTGATCCTAGGGACCCTCTCACTGGCCGTGCTGCTGATTCCCCCAGGAGCTTCTGAGACCATTGAAGCTGACCATGTGGGGACCTACGGCACAACTGTGTACCAGTCCTATGGGCCCTCAGGCCAGTATACACAAGAATTTGATGAGGATGAGAAGTTTTATGTGGACCTGCAGACGAAGGAGACTGTATGGCGGCTGTCAGAGTTTAGCCATTTTACTAGCTTTGACGCTCAGAGAGGGCTGAGAAACATAGCGACAATGAAGTACAACCTGGACATCCTGATCAAACGCTCCAATGAAACCTGGGCCGTCATGCCTCCTGAGGTGACTGTGTTCTCGGAGAGTCCTGTGGAGCTGGGCCAGCCCAATGTACTCATCTGCTTGGTGGACAACATCTTCCCCCCGGTGGTCAACATCACATGGCTTCGTAATGGGCAATTGGTCACCCAGGGTGTGTCTGAGACAGACTTCTATCCTCGGCCTGACCACAGATTCCGCAAGTTCCACTACCTCACTTTTCTCCCCAACACAGAAGATTTTTATGACTGCAAAGTGGAGCACTGGGGCCTGGAGCAGCCAGTCCTCAAGCACTGGGagCCCCAGGTTCCGTCCCCACTGCCAGAGACAACAGAAACTGTGGTCTGTGCCCTTGGTCTGGCTGTGGGCCTGGTGGGCATCATTGTAGGCACCATCCTCATAGTCAAAGGCATGCGTTCCAGCAGTAGGATCCAACACCAAGGTCCTCTGTGA

>Drgl_DMA_1

ATGGGACCTGAGCAAAGCCTGGGAGCTACACTGTTACTGCTGCAGCTGCAGTTGTCACTGCTTTTGTCCCTGTCCTGGGGAGCCACTCCAGCATTGACATCGTTACTCGGAGAGAGCCTACAGAACCACACATTCTCTCACGCGGTGTTCTGCCAGAATCAGGAGCCCTTCCTTGGCCTGACAGAGACTTTTGATGGGGATCTGCTCTTCTCCTTTGATTTCTCCAGGAACTCCTGGGTGCCCCGGCTGCCTGAGTTTGCTGCTTGGGCCGGTGATGAGGGTGACCTCCAGGCCATAGATTTTGACAAGGCGTTCTGTCAAAACCTGCAAAAAGCACTGAGTCGAATTTGTGAAGGCCAGATCCCTGAGGCTAGAGGAAACCCTGTGGCAGAAGTTTTCACTCTGGAGCCCCTGGAGTTTGGGAAGCCCAACACTCTCATCTGCTTCGTTAGTAACCTCTACCCCCCTCACATAACCGTGACCTGGCAGCACGAAGGAGTCCCGGTGGAAAGCAGCAGCCCCACTTTTCTCTCAGCCGTCGATGGACTTGGCTTCCAGGCCTTCTCTTATCTGAACTTCACACCCTCATCCACTGATGTCTTCTCTTGTACTGTGGCACGGGAAGGTGACCTCTTCAGCACCATAGCCTTCTGGGTACCTCAGAATCCGATACCTTCTGCATTGTTGGAAAACATACTGTGTGGCATTGCCTTTGGCCTGGGCATTGTCGGCATCATAGTGGGTGCTGTCCTCATCATCTGCTCCCAAAAACCATGTGGACGAGGTGCAGGTGA

>Bupa_DAA_1

ATGGCCCCCAACAAAGTCTTGATCCTAGGAGCCTTCGTCCTGGCAATGCTGCTGAGTCCCTGGGGAGCCAGGGCCATTAAAGAGAACCATGTGATCATCCAAGCAGAGTTCTACCAGACCCACAATCCCTTAGGAGAATTCATGTTCGACTTTGATGGGGATGAGATTTTCCACGTGGATTTGGGAAGGAGGGAGACAGTCTGGCGGCTTCCGGACTTCAGCAAATTTGCCAGCTTTGAGGCTCAGGGCGCTCTGGCCAATCTTGCTGTGGACAAAGCCAACCTGGAAATCATGATGAAGCGGTCCAACAACACCCCCGACAGCAACGTGCCCCCTGAAGTGACAGTGTTTCCCAAGAGCCCAGTGGAGCTGGGCCAGCCCAACATCCTCGTCTGCTTCATTGACAAGTTTTCTCCTCCTGTACTTAATGTGACATGGCTTCATAATGGGAATCCCATCACTGATGGTGTGTTTGAGACTGTCTTCCTCCCTCGTTCTGACCATGCCTTCAGAAAATTCCACTACCTCACCTTCATCCCCTCTGCCACTGATTACTATGACTGCAAGGTTGATCACTGGGGACTGGAACAGCCCACTGTCAAACACTGGAACCAGAAGTACGGACCCCACTGCCAGAGACAACAGAGACTGTGGTCTGTGCCCTAGGCCTGGCCATAGGCCTGGTGGGCATCACCGTAGGCACCATCCTTATTATCAAGGGCATGAGATCAAACAACACTTCCCGTGGTGGCTCCCGTGGACCCCTGTAA

>Bupa_DBA_1

ATGGCTGCCAACAACGTCCTGATCCTAGGGGCTCTCTTACTGGCTGTGCTGCTGAATCCCCAAGGAGCTTCCGAGTCCATTGAAGCTGACCACGTGGGTGTCTATGGCACAACTATGTTCCAGTCCTATGGGCCCTCAGGCCAATATACACAAGACTTTGATGAGGACGAGTTGTTTTATGTGGACCTGCAGAAGAAGGAGACTGTATGGCGGCTGCTGGAGTTTAGCCATTTTGCTGGCTTTGATCCTCAGGGAGGGCTAAGAAACCTAGCCACAGTCAAGCACAACCTGGACATCCTGATCCAGCGCTCCAACAGAAGCAAGGCCATCATTGTGCCCCCTGAGGTGACTGTATTCTCAGAGAGTCCCGTGGAGATGGGCCAGCCAAATGTACTCATCTGCTTGGTGGACAACATCTTCCCCCCAGTGGTCAACATTGCATGGCTTCGTAATGGGCAGTTGGTCAGCACAGGTGTGTCTGAGACAGACTTCTACCCTCGGCCTGACCACAAATTCCGCAAGTTCTACTACCTCACTTTTCTTCCCAACACAGAAGACTTTTATGACTGCAAAGTGGAGCACTGGAGCCTGAAGCAACCAGTCCTCAAGCACTGGGAACCCCAGGGTGCATCCCCACTGCCAGAGACAACAGAAACCGTGGTCTGTGCCCTTGGTCTGGCTGTGGGCCTGGTGGGCATTGTTGTGGGCACCATCCTCATAATTAGAGGCATGCGTTCCAGCAGTAGGATCCAACATCAAGGGCCCCTGTGA

>Bupa_DCA_1

ATGACCTCCACTCTAGACTAAATCTTGGGGATCCTCAGCCTGGCTGCCCTGCTAATCAGACATAGAACCTGGGCCACTAATGACCATGTAACCAGCTCGGTGATATTTGTTCAGACCCACAAACCATCTGGGCAGTACTTGCAAGATGAGGATAAGCAATTCCATGGGGACTATGACAGGAAGGAAACAGTCTGGCGGCTTCCTGAGTTTGGCCATATCTTCAGTTTTGATGCACACACTGGGCTAGGTAACATTGCTGTGGACATGGCTAATTTGAACCAACTTACCAGGCAAACCAACCACACCCAGGCCACCATTGTGACTCCAGAGGTGACAGTGTTTCCCAAGGAGCCTGTGGAACTAGAACCCAACATCCTCATCTGCCACATCGATAAGCTCTCACCCCCAGTGATCAATGTCACATGGCTGTGCCACGGAGCCAGTCACCACAGGGGTATCTGAGACTACCTTCCTGCCTTGGGATGACTATTCTTTCCACGAGTTCCATTACCTCACTTTTCTCCCCTCAACTGATGCTATCTATGTGTAGTTGAACAGTGGGGCCTGAAAAAGCCACTTTTCAAGTATTGGGCCTGACATGCTAACATCACCATCTGAAACAATGGAGATGCTCATCTGTACTCTAGGACTGGCTGTGGGCCTGGTGGGCATCCCTGTGGCTGCCACCCTGATTATCAGAGGCTCCAGCAAATGTCTGACCCCATTGATCAAGTATCACCTTCTGTGAGAAATCTTTTCCAATCCTCCTTAATCTTAGTGCCTTCCCCTAA

>Bupa_DMA_1

ATGGGACCTGAGCAAAGCCTGGGAGCTATACTGTTACTTCTGCAGCTGCAGTCATCACTGTTTTTGTCCCTGTCCTGGGGAACCACTCCAGCAGTGTTGGCATCATTATTGGGCAATAGTCTACAGAACGACACATTCTCTCACACACTGTTCTGCCAGGATGCAGAACCCTTTCTGAGTCTGTCTGAGACCTTCAATGGGGACCAGCTCTTCTCCTTCGACTTCTCCAAGAACTCTCGGGTGCCCCGGCTGCCTGAGTTTGCTGCTTGGGCCAGTGATGAGGGAGACATCACGGCCATAGAAACTGACAAGAACCTCTGCCAGGAATTGCAAAGAGAATTGAGCAACATTTTGGAAGGCCAAATCCCTGTGGCTAGAGGAAACCCTGTGCCTAAAATTTTCACTCTGGAGCCCCTGGAGTTTGGGAAGCCCAACACTCTCATCTGCTTTGTTAGTAACATCTTCCCACCTCAAATAACTGTGACCTGGCAGTACAAAGGAGTCTCTGTGGAAAGCAGCAGCCCCGTTTTTCTCTCAGCTGTAGATGGACTTGGCTTCCAGGCTTTCTCTTATCTGAACTTCACACCCACATCTTCTGATATCTTCTCTTGCATTGTAGCACGGGAAGGTGACCTCTTCAGTGCTATAGACTTCTGGGTGCCTCAGAATCCAATACCCTCTGAATTGTTGGAAAATATACTGTGTGGTATTGCCTTTGGCCTGGGTGTTGTTGGCATCATAGTGGGTGCTGCCCTCATCATCTACTTCCGAAAACCATGTGCAAGTGGTGCAGGTGA

>Daha_DNAzoo_DAA_1

ATGATGCCCAACAAAGCTTTGATCGTAGGGGCTTTCACCCTGGCCGTGCTGTTGAATCCCTGGGGAGCCAGAGCCATTAAAGAGAATCATGTGATCATCCAAGCTGAGTTCTACCAGACCCACAAACCTTTAGGAGAGTTCATGTTTGATTTTGATGGGGATGAAATTTTCCATGTGGATTTGGACAAGAGAGAGACAGTCTGGCGTCTTCCTGACTTCAGCAAATTTGCCAGCTTTGAGGCTCAGGGTGCTCTGGCCAATCTTGCTGTGGACAAAGCCAATCTGGAAATCATAATGAAACGGTCCAACAACACTCCTGATACTAATGTGCCCCCTGAAGTGACAGTGTTTCCTAAAAGCCCAGTGGAGATGGACCAGCCCAATGTTCTTATCTGTTTCATTGACAAGTTCTCTCCTCCCGTACTTAATGTGACATGGCTTCGTAATGGGCAGCCCATCACTGATGGTGTGTTTGAGACTGTCTATCTCCCCCGCCCTGATCATGCCTTCAGAAAATTCCACTACCTCACCTTCATCCCCTCTGCCAATGATTACTATGACTGTGAGGTCGATCACTGGGGACTGGAACAACCTCTTATGAAACATTGGGGTAAAAATACGAACCCCACTGCCAGAGACAACAGAGACTGTGGTCTGTGCCCTGGGCCTAGCCATTGGCCTAGTGGGCATCGTTGTGGGCACCATCCTGATTATCAAGGGCATGAAATCAAGCAACGCTTCCCGTGGTGGACCTCGTGGACCCCTGTAA

>Daha_DNAzoo_DMA_1

ATGGGACTTGAGGAAAACCTGGGAGTTACACTGTTACTACTGAAGCTACAGTCATCATTGCTTTTGTCCCTGTTCTGGGAAGCCACTCCAGTGTTGGCCGTGTTTGGGAATAATTTACAGAATTACACATTCTCTCACATACTATTCTGCCAGAATGGAGAATCCTCTTTGGGTCTGTCAGAAAACTTCAATGGGGACTATCTCTTCTCCTTTGACTTCTCCAAGAACTCCCGGGTGCCCCGGCTGCCTGAATTTGCTGCTTGGGCCACTGATAAAGGAGACATCAAGACCATAGATGCTGACAAGAATCTTTGCCAGGAGCTGCAACATCAATTGAGTAGTCTTTGTAAAGGACAGATCCCTGAGGCTAGAGGAAACCCTGTGGCTGAAGTTTTCACTCTGGAGCCCCTGGAGTTTGGGAAGCCCAACACTCTTGTTTGTTTTGTTAGCAATGTCTTCCCTCCTCGTATAACCGTGACCTGGCAACATGAAGGAGTCTCTGTGGAAAGCAGCAGCCCCACCTTTCTCTCAGCTACAGATGGACTTGACTTCCAGGCCTTTTCTTATCTGAACATCACACCCACATCCACTGATGTTTTCTCTTGCACTGTGACACAGGAAGGTGACCTCTTCAGCACTATAGCGTTTTGGGTGCCTCAGAATCCAATACCCTCTGCATTGTTGGAAAACATACTGTGTGGTATTGCCTTCGGACTGGGCATTGTTGGTATCATCGTAGGTGCTTCCCTCATCATCTACTTCCAAAAGCCATGTGTAAATGGTGCAGGTGA

>Myfa_DNAzoo_DAA_1

ATGATCCCCAACAAAGCTTTGATCCTAGGGGCTTTCACCCTGGTAGTGCTGCTGAATCCCTGGGGAGCCAGTGCCATTAAAGAGAATCATGTAATCATCCAAGCTGAGTTCTACCAGACCCACAACCCTTCAGGAGAGTTCATGTTTGATTTTGATGGGGATGAAATTTTCCATGTGGATTTGGAGAAGAAAGAGACAGTCTGGCGTCTTCCTGACTTCAGCAAATTTGCCAGCTTTGAGGCTCAGGGTGCTCTGGCCAATCTTGCTGTAGACAAAGCTAACCTGGAAATCATGATGAAACGGTCCAACAATACTCCTGATACCAATGTGCCCCCTGAAGTGACAGTGTTTCCTAAGGGTCCAGTGGAGCTGGGCACACCCAATGTCCTTGTCTGCTTTATTGACAAGTTCTCTCCTCCGGTACTTAATGTGACATGGCTTCATAACGGGCATCCCATCACTGATGGTGTGTTTGAGACTGTCTTCCTCCCTCGACCTGATCATTCCTTCAGAAAATTCCATTACCTCACCTTCATCCCCTCTGCCAATGATTATTATGACTGTAAGGTCGATCACTGGGGACTGGAACAATCTGTTTTGAAACATTGGGAACCAGAAATAGGAACCCCACTGCCAGAGACAACAGAGACTGTGGTCTGTGCCCTGGGCCTAGCCATAGGCCTAGTGGGTATCATTGCAGGCACCATCCTGATTATCAAGGGCATGCGATCGAGCAACACTTCTCCTGGTAGCCCTCGTGGACCTCTGTAA

>Myfa_DNAzoo_DMA_1

ATGGGACTTGAGCAAAGCTTGGGAGTTAAACTGTTACTACTGCAGCTACAGTCATCATTGCTTTTGTCCCTGTTCTGGGAAGCTACTCCAGTGTTGGCAGTGTTGGGGAATAACTTACAGAACTACACATTTTCTCACACACTGTTCTGCCAGAATGGGGAATCCTCCTTGGGTCTGTCGGAAGACTTCAATGGGGACCATCTCTTCTCCTTTGACTTCTCCAAGAACTCCCGGGTGCCCCGGCTGCCTGAGTTTGCTGCTTGGGCCAGTGATAAAGGAGACATCAAGGCCATAGATGCTGACAAGAATCTCTGCCAGATGCTGCAACATGCATTGAGTAGATGTTGTAAAGACTGGATCCCTGAGGCTAGAGGAAACCCTGTGGCTGAAGTTTTCACTCTGGAGCCCCTGGAGTATGGGAAGCCCAATACTCTCGTTTGTTTTGTTAGCAACTTCTTCCCACCTCCTGTAACCGTGACCTGGCAACATGAGGGAGTCTCTGTGGAAAGCAGCAGCCCCACCTTTCTTTCAGCTACAGATGAACTTGGCTTCCAGGCGTTTTCTTATTTGAACTTCACACCCACATCCACTGATGTTTTTTCTTGCACTGTGGCAAAGGAAGGTGAAGTCTTCAACACTATAGCTTTTTGGGTGCCTCAGAATCCAATACCCTCTGCATTGTTGGAAAACATACTGTGTGGCATTGCCTTTGGCCTGGGCATTGTTGGTATCATTGTGGGTGCTTCCCTCATCATCTACTTCAAAAAGCCATGTGCAAATGGTGCAGGTGA

>Myfa_DAA_1

ATGATCCCCAACAAAGCTTTGATCCTAGGGGCTTTCACCCTGGTAGTGCTGCTGAATCCCTGGGGAGCCAGTGCCATTAAAGAGAATCATGTAATCATCCAAGCTGAGTTCTACCAGACCCACAACCCTTCAGGAGAGTTCATGTTTGATTTTGATGGGGATGAAATTTTCCATGTGGATTTGGAGAAGAAAGAGACAGTCTGGCGTCTTCCTGACTTCAGCAAATTTGCCAGCTTTGAGGCTCAGGGTGCTCTGGCCAATCTTGCTGTAGACAAAGCTAACCTGGAAATCATGATGAAACGGTCCAACAATACTCCTGATACCAATGTGCCCCCTGAAGTGACAGTGTTTCCTAAGGGTCCAGTGGATCTGGGCACACCCAATGTCCTTGTCTGCTTTATTGACAAGTTCTCTCCTCCGGTACTTAATGTGACATGGCTTCATAACGGGCATCCCATCACTGATGGTGTGTTTGAGACTGTCTTCCTCCCTCGACCTGATCATTCCTTCAGAAAATTCCATTACCTCACCTTCATCCCCTCTGCCAATGATTATTATGACTGTAAGGTCGATCACTGGGGACTGGAACAATCTGTTTTGAAACATTGGGAACCAGAAATAGGAACCCCACTGCCAGAGACAACAGAGACTGTGGTCTGTGCCCTGGGCCTAGCCATAGGCCTAGTGGGTATCATTGCAGGCACCATCCTGATTATCAAGGGCATGCGATCGAGCAACACTTCTCCTGGTAGCCCTCGTGGACCTCTGTAA

>Psco_DNAzoo_DAA_1

ATGGCCCCCAACAAAGCCTTGATCCTAGAAGCCTTCATCCTGGCAGTGCTGCTGAGTCCCTGGGGAGCCAGGGCCATTAAAGAGAACCATGTGATCATCCAAGCAGAGTTCTACCAGACCGCCAAACCCTCCGGAGAGTTCATGTTTGACTTTGATGGGGATGAGATTTTCCATGTGGATTTGGAAAAGAAGGAGACAGTCTGGCGGCTTCCTGACTTCAGCAAATTTGCCAGCTTTGAGGCTCAGGGTGCTTTGGCCAATCTTGCTGTGGACAAAGCCAATCTGGAAATCATGATGAAACGGTCCAACAACACTCCTGACACCAATGTGCCCCCTGAAGTGACAGTGTTTCCCAGGGGCCCAGTGGAGCTGGGACAGCCCAACGTCCTTGTCTGTTTCGTTGACAAGTTCTCTCCTCCTGTACTTACTGTGACATGGCTTCATAATGGGAATCCCGTCACTGATGGTGTGTTTGAGACTGTCTTCCTTCCCCGCCCTGACCATGCCTTCAGAAAATTCTACTACCTCACCTTCATCCCCTCTGCCACTGATTACTATGACTGCAAGGTCGAGCACTGGGGACTGGAACAACCTGTTGTCAAACACTGGGAACCAGAAATACGGACCCCACTGCCAGAGACAACAGAGACTGTGGTCTGTGCCCTAGGCCTGGCCATAGGCCTGGTGGGCATCATCGTAGGCACCATCCTTATTATCAAGGGCATGAAATCAAACAACACTTCCCGTGGTGGCTCCCGTGGACCCCTGTAA

>Psco_DNAzoo_DMA_1

ATGGGACCTGTGCAAATCCTGGGAGCTACACTGTTATTCCTGCAGCTGCAGTCATCACTGTTTTTGTTTCTGTCCTGGGGAGCCACTCCAGTGTTGGCATCATTACTGGGGAATAGCCTACAAAACTACACATTCTCTCACACACTGTTCTGCCAGGATAAGGAACCCTTCCTGGGTCTATCGGAGACCTTCAATGGGGACCAGCTCTTCTCCTTCGACTTCTCTAGGAACTCTCTGGTGCCCCGGCTGCCTGAGTTTGCTGCTTGGACTGGTGATGAGGGAGACATTGAGACCATAAAAAGTGATGGGAAGCTCTGCCAGGAATTGCAAAAAGTTTTGAGTAGAATTTTGGAAGACAAAATCCCTGAGGCTAGAGGAAACCCTGTGGCTGAAATTTTCACTCTGGAGCCCCTGGAGTTTGGGAAGCCCAACACTCTCACTTGCTTTGTTAGTAATATCTTCCCACCTCAAATAACTGTGAGCTGGCAGCACAAAGGAGTCCCTATGGAAAGCAGCAGCCCCACTTTCCTCTCAGCTGTGGATGGACTTGGCTTCCAGGCCTTCTCTTATCTGAACTTCACACCCACATCCTCTGATGTCTTCTCTTGCACTGTGGAACAGGAAGGTGACCTCTTCAGCACTATCACCTACTGGGTGCCTCAGGATCCGATACCCTCTGAACTGTTGGAAAATATACTGTGCGGTATTGCCTTTGGCCTGGGAATCATTGGCATCATAGTGGGTGCTGCCCTCATCATCTACTTCCGAAAACCATGTGCAAGTGGTGCAGGTGA

>Sebr_DNAzoo_DAA_1

ATGACTTCCAACAAGTCCTTGATCCTAGGAGCCTTCATTCTGTCAGTGCTGCTGAGTCCCTGGGGAGCCAGGGCCGTTAAAGAGAACCATGTGATCATCCAAGCAGAGTTCTACCAGACCAACGAACCCTCTGGAGAGTTTATGTTCGACTTTGATGGGGATGAGATTTTCCATGTGGATTTGAAGAAGAGAGAGACAGTCTGGCGGCTTCCTGACTTCAGCAAATTTGCCAGCTTTGAGGCTCAGGGTGCCTTGGCCAATCTTGCTGTGGACAAAGCCAACCTGGAAATCATGATGAAACGATCCAATAACACCCCTGACACCATGGGCCCTGAAGTGACAGTGTTTCCCAAAGGCCCAGTGGAGCTGGGCCAGCCCAACATCCTGGTCTGCTTCGTTGACAAGTTCTCTCCTCCAGTACTTACTGTGACCTGGCTTCATAATGGGGTTCCCATCACTGATGGTGTGTTTGAAACTGTCTTCCTCCCTCGCTCTGACCATGCCTTCAGAAAATTCCACTACCTCACCTTCATCCCCTCTGCCACCGATTACTATGACTGTAAGGTTGAGCACTGGGGACTGGAACAACCTGTTGTCAAACACTGGGAACCAGAAGTACGGACCCCACTGCCAGAGACAACAGAGACTGTGGTCTGTGCCCTAGGCCTGGCCATAGGCCTTGTGGGCATCATCGTAGGCACCATTCTTATTATCAAGGGCATGAGATCAAACAACACTTCCCGTGGTGGCTCCCGTGGACCCCTGTAA

>Sebr_DNAzoo_DBA_1

ATGACTGCCAGCAGAGTGCTGATCCTAAGGACCTTGTCCCTGGTTGTGCTGCTGAGTCCCCAAGGAACTTCTCAGTCCATTGAAGGCAACTGACCACGTGGGGATCTACGGCACAGGTGTATACCAGTCCTATGAGTCCTCAGGCCAGTACACACAGGAATTTGATGAGGATGAGCTGTTTTACGTAGACCTGCAGAAGAAGGAGACTGTGTGGCGGCTGCCAGAGTTTAGCCATTTTAGCAGCTTTGACCCTCAGGGAGGGCTGCGTGAAAAAGCCACATGCAAGTACAACCTGGACATCCTGATCAAGCGCTCCAACAGAAGCAGGGCCATCATGCCCCCTGAAGTGACTGTGTTCTCGGAGAGTCCTGTGGAGTTGGGCCAGCCAAACGTACTCATCTGCTTGGTGGACAACATCTTCCCTCCAGTGGTCAACATCAAGTGGCTTCGTAATGGCCAGGTGATCACCACTGGTGTGTCTGAGACAGACTTCTACTCTCGGCCTGACCACAAATTCCGCAAGTTCTACTATCTCACTTTTCTCCCCAACACAGAAGACTTTTATGACTGCAAAGTGGAGCACTGGGGCTTGGAACAGCCACTCCTCAAGCACTGGGAACCCCAGATTCCATCCCCAGTGCCAGAGACAACAGAAACTGTGGTCTGTGCCCTTGGTCTGGCAGTGGGCCTGGTGGGCATCATTGTGGGCACCATCCTTATAATCAGAGGCATGCGTTCCAACAATAGGTTCCAACATCAAGGGCCTCTGTGA

>Sebr_DNAzoo_DCA_1_partial_exon_2-4

ACCATGTAATCAGCTCTGTGACAATTGTTCAGACCTACAAACCATCTGAGCAGTACCTGCATGAATTTGATGAAGATGAACCATTTCACATGGACTATAAAAAGAAGGAAACAGTCTGGCAGCTTCCTGAGTTTGGCCGTATCTTCAGTTCCAGTGCACAGAATGGGCTAGGTGACATTGCTGCGGACATGGCTAACTTGAACCAACTTATCAGGCAAACCAAGCATATCCAAGCCACCACTGTGACTCCAGAGGTGGCAGTGTTTCCCAAAGAGGCTGTGGAACTAGAAGAACCCAGCGTCCTCATCTACCACATTGATAAGTTCTCCATCCCAGTGATCAATGTCATATGGCTGTGCAATGGTGAGTCAGTCACCACAGCGGTATCTGAGACCACCTTCCTGCCTCAGGATGACTGTTATTCCCACAAGTTTCATTACTTCACTTTCTTCCTCTCAACTGATGATATTTATGACTGTGTAGTTGAACCCTGGGGCCTCAAAAACCACTTTTCAAGTAGCCTGAGACGTTAACACCACCGTCTGAGACAATGGAGATGCTCATCTCTATTCTAGGAATGGCTATGGGCCTGGTGGGCATCATGGTGGCTGCCAGCTTCATTATCAGAGGCTTGTGCTCAGGCAAATGGTTCCTGA

>Sebr_DNAzoo_DMA_1

ATGGGACCTGTGCAAAGCCTGGGAGCTACACTGTTACTCCTGCAGCTGCAGTCATCACTGTTCTTGTCTCTGTCCTGGGGAGCCACTCCAGCAGTGTTGGCATCATTACTGGGGAATAGTCTACAGAACTATACATTCTCTCACATAATGTTCTGCCAGGATGAGCAACCCTTAGTGGGTCTGTCTGAGGTCTTCGATGGGGACCTGCTCTTCTCCTTCGACTTCTCCAGGAACACTCGGGTACCCCGGCTGCCTGAGTTTGGTGCTTGGGCCAGTGATGAGGGAGATATCAAGATCATAGAAGATGACAAGACACTCTGCCAGGAATTGCAGAAACTTTTGAGTGGAAGTTTGGAAGGCCAAATCCCAGAGGCTAAAGGAAACCCTGTGGCTGAAGTTTTCACTCTGGAACCCCTGGAGTTTGGGAAGCCCAACACTCTCATTTGCTTTGTTAGTAACATCTTCCCACCTCAGATAACAGTGACTTGGCAGTACAAAGAAGTTCCTGTGGAAAGCAGCAGCCCCACTTTTCTCTCTGCTATAGATGGACTTGGCTTCCAGGCCTTCTCCTATCTGAATTTCACACCCACTGCCTCTGATATCTTCTCTTGCACTGTGACACGGGAAGGCGAACTCTTCAGCACTATAGCCTTCTGGGTGCCTCAGAATCCGATACCCTCTGAATTGCTGGAAAATATACTGTGCGGTATTGCCTTTGGCCTGGGAATTCTAGGCATCATAGTAGGTGCTGCCCTCATCATCTACTTCCAAAAACCATGTGGAAGTGGTGCAGGTAA

>maru_DNAzoo_DAA_1

ATGACTTCCAACAAATCCTTGATCCTAGGAGCCTTCATTCTGTCAGTGCTGCTGAGTCCCTGGGGAGCCAGGGCCATTAAAGAGAACCATGTGATCATCCAAGCAGAGTTCTACCAGACCCACGAACCCTCTGGAGAGTTTATGTTCGACTTTGATGGGGATGAGATTTTCCATGTGGATTTGAACAAGAGAGAGACAGTCTGGCGGCTTCCTGACTTCAGCAAATTTGCCAGCTTTGAGGCTCAGGGTGCCTTGGCCAATCTTGCTGTGGACAAAGCCAACCTGGAAATCATGATGAAACGATCCAATAACACCCCTGACACCAATGTGGGCCCTGAAGTGACAGTGTTTCCCAAAGGCCCAGTGGAGCTGGGCCAGCCCAACATCCTTGTCTGCTTCATTGACAAGTTCTCTCCTCCGGTACTTACTGTGACCTGGCTTCATAATGGGGTTCCCATCACTGATGGTGTGTTTGAAACTGTCTTCCTCCCTCGCTCTGACCATGCCTTCAGAAAATTCCACTACCTCACCTTCATCCCCTCTGCCACCGATTACTATGACTGTAAGGTTGAGCACTGGGGACTGGAACAACCTGTTGTCAAACACTGGGAACCAGAAGTACGGACCCCACTGCCAGAGACAACAGAGACTGTGGTCTGCGCCCTAGGCCTGGCCATAGGCCTTGTGGGCATCATCGTAGGCACCATTCTTATTATCAAGGGCATGAGATCAAACAACACTTCCCGTGGTGGCTCCCGTGGACCCCTGTAA

>maru_DNAzoo_DBA_1

ATGGCTGCCAGTAGAGTGCTGATCCTAAGGACCCTGTCACTGGTTGTGCTGCTGAGTCCCCAAGGAACTTCTCAGTCCATTGAAGGCAACTGACCACGTGGGGATCTACGGCACAGGTGTATACCAGTCCTATGAGTCCTCAGGCCAGTACACACAGGAATTTGATGAGGACGAGCTGTTTTATGTAGATCTGCAGAAGAAGGAGACTGTGTGGCGGCTGCCAGAGTTCAGCCATTTTAGCAGCTTTGACCCTCAGGGAGGGCTGCGTGAAAAAGCCACATGCAAGTACAACCTGGACATCCTGATCAAGCGCTCCAACAGAAGCAGGGCCATCATGCCCCCTGAAGTGACTGTGTTCTCAGAGAGTCCTGTGGAGTTGGGCCAGCCAAACGTACTCATCTGCTTGGTGGACAACATCTTCCCTCCAGTGGTCAACATCAAGTGGCTTCGTAATGGCCAGGTGATCACCACTGGTGTGTCTGAGACAGACTTCTACTCTCGGCCTGACCACAAATTCCGCAAGTTCTACTACCTCACTTTTCTCCCCAACACAGAGGACTTTTATGACTGCAAAGTGGAGCACTGGGGCTTGGAGCAGCCACTCCTCAAGCACTGGGAACCCCAGATTCCATCCCCAGTGCCAGAGACAACAGAAACTGTGGTCTGTGCCCTTGGTCTGGCAGTGGGCCTGGTGGGCATCATTGTAGGCACCATCCTTATAATCAGAGGCATGCGTTCCAGCAATAGGTTTCAACATCAAGGGCCTCTGTGA

>maru_DNAzoo_DCA_1_partial_exon_2-4

ACCATGTAATCAGCTCTGTGACAATTGTTCAGACCTACAAACCATCTGAGCAGGACCTGCATGAATTTGATGAAGATGAACCATTTCACATGGACTATGAAAAGAAGGAAACAGTCTGGCAGCTTCCTGAGTTTGGCCGTATCTTCAGTTCCAGTGCACAGATTGGGCTAGGTGACATTGCTGCGGACATGGCTAACTTGAACCAACTTATCAGGCAAACCAAGCACACCCAAGCCACCATTGTGACTCCAGAGGTGGCAATGTTTCCCAAGGAGGCCGTGGAACTAGAAGAACCCAGCGTCCTCCTTTACCACATTGATAAGTTCTCCATCCCAGTGATCAGTGCCATATGGCTGTGCAGTGGTGAGTTAGTCACCACAGCAGTATCTGAGACCGCCTTCCTGCCTCAGGATGACTGTTCTTCCCACAAGTTTCATTACTTCACTTTCTTCCTCTCAACTGCTGATATTTATGACTGCGTAGTTGAACCCTGGGGCCTCAAAAACCACTTTTCAAGTAGCCTGAGACGCTAACACCACCGTCTGAGACAATGGAGATGCTCATCTCTATTCTAGGAATGGCTATGGGCCTGGTGGGCATCATGGGGGCTGCCAGCTTCATTGTCAGAGGTTTGTGCTCAGGCAAATGGTTCCAGACCCCACAGATCAAGTAATTTTCTGCAATAAATCTTTCCAATCCTCCTTAATCTTAGTGCCTTCCCCTGA

>maru_DNAzoo_DMA_1

ATGGGACCTGTGCAAAGCCTGGGAGCTACACTGTTACTCCTGCAGCTGCAGTCATCACTGTTCTTGTCTCTGTCCTGGGGAGCCACTCCAGTGTTGGCATCATTACTGGGGAATAGCCTACAGAACTATACATTCTCTCACATAATGTTCTGCCAGGATGAGCAACCCTTAGTGGGTCTGTCTGAGGTCTTCGATGGGGACCTGCTCTTCTCCTTCGACTTCTCCAGGAACACTCGGGTACCCCGGCTGCCTGAGTTTGGTGCTTGGGCCAGTGATGAGGGAGATGTCAAGATCATAGAATATGACAAGACACTCTGCCAGAATTTGCAGAAAGGTTTGAGTGAAAGTTTTGAAGGCCAAATCCCAGAGGCTAGAGGAAACCCTGTGGCTGAAGTTTTCACTCTGGAACCCCTGGAGTTTGGGAAGCCCAACACTCTCATTTGCTTTGTTAGTAACATCTTCCCACCTCAGATAACAGTGACTTGGCAGTACAAAGAAGTTCCTGTGGAAAGCAGCAGCCCCACTTTTCTCTCTGCTATAGATGAACTTGGCTTCCAGGCCTTCTCCTATCTGAATTTCACACCCACTGCCTCTGATATCTTCTCTTGCACTGTGGCACAGGAAGGCGAACTCTTCAGCACTATAGCCTTCTGGGTGCCTCAGAATCCGATACCCTCTGAATTGCTGGAAAATATACTGTGCGGTATTGCCTTTGGCCTGGGAATTCTAGGCATCATAGTGGGTGTTTCCCTCATCATGTACTTCCAAAAACCATGTGGAAGTGGTGCAGGTAA

>Maeu_DNAzoo_DAA_1

ATGACTTCCAACAAATCCTTGATCCTAGGAGCCTTCATTCTGTCAGTGCTGCTGAGTCCCTGGGGAGCCAGGGCCATTAAAGAGAACCATGTGATCATCCAAGCAGAGTTCTACCAGACCCACGAACCCTCTGGAGAGTTTATGTTCGACTTTGATGGGGATGAGATTTTCCATGTGGATTTGAACAAGAAAGAGACAGTCTGGAGGCTTCCTGACTTCAGCAAATTTGCCAGCTTTGAGGCTCAGGGTGCCTTGGCCAATCTTGCTGTGGACAAAGCCAACCTGGAAATCATGATGAAACGATCCAATAACACCCCTGACACCAATGTGGGCCCTGAAGTGACAGTGTTTCCCAAAGGTCCAGTGGAGCTGGGCCAGCCCAACATCCTTGTCTGCTTCATTGACAAGTTCTCTCCTCCGGTACTTACTGTGACCTGGCTTCATAATGGGGTTCCCATCACTGATGGTGTGTTTGAAACTGTCTTCCTCCCTCGCTCTGACCATGCCTTCAGAAAATTCCACTATCTCACCTTCATCCCCTCTGCCACCGATTACTATGACTGTAAGGTTGAGCACTGGGGACTGGAACAACCTGTTGTCAAACACTGGGAACCAGAAGTACGGACCCCACTGCCAGAGACAACAGAGACTGTGGTCTGCGCCCTAGGCCTGGCCATAGGCCTTGTGGGCATCATCGTAGGCACCATTCTTATTATCAAGGGCATGAGATCAAACAACACTTCCCGTGGTGGCTCCCGTGGACCCCTGTAA

>Maeu_DNAzoo_DBA_1

ATGGCTCCCAGCAGAGTGCTGATCCTAAGGACCCTCTCACTGGTTGTGCTGCTGAGTCCCCAAGGAAATTCTCAGTCCATTGAAGCTGACCATGTGGGGATCTACGGCACAGGTGTATACCAGTCCTATGAGTCCTCAGGCCAGTACACACAGGAATTTGATGAGGACGAGCTGTTTTATGTAGACCTGCAGAAGAAGGAGACTGTGTGGCGGCTTCCAGAGTTTAGCCATTTTAGCAGCTTTGACCCTCAGGGAGGGCTGCGTGAAAAAGCCACATGCAAGTACAACCTGGACATCCTGATCAAACGCTCCAACAGAAGCAGGGCCATCATGCCCCCTGAAGTGACTGTGTTCTCAGAGAGTCCTGTGGAGTTGGGCCAGCCAAACGTACTCATCTGCTTGGTGGACAACATCTTCCCTCCAGTGGTCAACATCAAGTGGCTTCGTAATGGCCAAGTGATCACCACTGGTGTGTCTGAGACAGACTTCTACTCTCGGCCTGACCACAAATTCCGCAAGTTCTACTACCTCACTTTTCTCCCCAACACAGAGGACTTTTATGACTGCAAAGTGGAGCACTGGGGCTTGGAGCAGCCACTCCTCAAGCACTGGGAACCCCAGATTCCATCCCCAGTGCCAGAGACAACAGAAACTGTGGTCTGTGCCCTTGGTCTGGCGGTGGGCCTGGTGGGCATCATTGCGGGCACCATCCTTATAATCAGAGGCATGCGTTCCAGCAGTAGGATCCAACATCAAGGACCTCTGTGA

>Maeu_DNAzoo_DBA_2

ATGGCTGCCAACAGAGTTCTGATCCGAGGGACCCTCTCACTAGTTTTGCTCCTGAGTCCCCAAGGAGCTTGAGTCTGTTGAAGCCTGACCATGTTAGGGTGTATGGCACAGATATATGCCAGAATTATGGGCCCTCAAGCCAGTACACACAAGAATTTGATGATGATGAGCTGTTTTATGTGGACCTGCAGAAGGAGATTGTATGGTGGCTGCCAGAGTTTAGCCATTTTGCTGGCTTTGACCCTCAGGGAGGGTTAAGAAACATACGTATACCCAAGCTCACCATGGACACCCTCATCAAACACTCCAACAGAAGCAGGGCCATCAGGGTGCCCCCTGAGGTGACTGTATTCTCAGAGAATACTGTGGAGATAGGCCAGCCAAATGTACTCATCTGCTTGGTGGACAACATCTTCCCCTCAGCTATCAGCATCATGTGGCTTCATAATGACCAGCTGGTCACCACTGGTGTGTCTGAGACATACTTTTACTCTCGGCCTGACCATGAATTCCACAAGTTCTATTACCTCACTTTTCTCCCCAACACAGAGGATTTTTATGACTGCAAAGTGGAGCACTGGGGCTTAGAACAGCCAGTCCTCAAGCACTGGGAACCCCAGATTCCATCCCCAGAGCCAGAGACAACAGAAACTGTGGTCTGTGCCTTTGGTCTGACAGTGGGCCTGGTGGGCATCATTGTGGGCACCATCCTTATAATCAGAGGCATGTGTTCCAGCACTGGGATCCAACATCAAGGGCCTCTGTGA

>Maeu_DNAzoo_DCA_1_partial_exon_2-4

ACCATGTAATCAGCTCTGTGACAATTGTTCAGACCTACAAACCATATGAGCAGGACCTGCATGAATTTGATGAAGATGAACCATTTCACATGGACTATGAAAAGAAGGAAACAGTCTGGCAGCTTCCTGAGTTTGGCCGTATCTTCAGTTCCAATGCACAGATTGGGCTAGGTGACATTGTTGTGGACATGACTAACTTGAACCAACTTATCAGGCAAACCAGCCACACTCAAGTCACCACTGTGACTTCAGAGGTGGCAATGTTTCCCAAGGAGGCCGTGGAATTAGAAGAACCCAGCGTCCTCCTTTACCACATTGATAAGTTCTCCATCCCAGTGATCAATATCACATGGCTGTGCAATGGTGAGTCAGTCACCACAGCGGTATCTGAGACCGCCTTCCTGCCTCAGGATGACTGTTATTCCCACAAGTTTCATTACTTCACTTTCTTCCTCTCAACTGATGATATTTATGACTGTGTAGTTGAACCCTGGGGCCTCAAAAACCACTTTTCAAGCCCTGAGACGCTAACACCACCGTCTGAGACAATGGAGATGCTCATCTCTATTCTAGGAATGGCTATGGGCCTGGTGGGCATCATGGTGGCTGCCAGCTTCATTATCAGAGGCTTGTGCTCAGGCAAATGGTTCCTGACGCCACAGATCAAGTATAATTTTCTGCAAGAAATCTTTCCAATCCTCCTTAATCTTAGTGCCTTCCCCTGA

>Maeu_DNAzoo_DMA_1

ATGGGACCTGTGCAAAGCCTGGGAGCTACACTGTTACTCCTGCAGCTGCAGTCATCACTGTTCTTGTCTCTGTCCTGGGGAGCCACTCCAGTGTTGGCATCATTACTGGGGAATAGCCTACAGAACTATACATTCTCTCACACAATGTTCTGCCAGGATGAGCAACCCTTAGTGGGTCTGTCTGAGGTCTTCGATGGGGACCTGCTCTTCTCCTTCGACTTCTCCAGGAACACTCGGGTACCCCGGCTGCCTGAGTTTGGTGCTTGGGCCAGTGATGAGGGAGATGTCAAGATCATAGAAGATGACAAGGCACTCTGCCAGGATTTGCAGAAAGGTTTGAGTGAAACTTTTGAAGGCCGAATCCCAGAGGCTAGAGGAAACCCTGTGGCTGAAGTTTTCACTCTGGAACCCCTGGAGTTTGGGAAGCCCAACACTCTCATTTGCTTTGTTAGTAACATCTTCCCACCTCAGATAACAGTGACTTGGCAGTACAAAGAAGTTCCTGTGGAAAGCAGCAGCCCCACTTTTCTCTCTGCTATAGATGGACTTGGCTTCCAGGCCTTCTCCTATCTGAATTTCACACCCACTGCCTCTGATATCTTCTCTTGCACTGTGGCACGGGAAGGCGAACTCTTCAGCACTATAGCCTTCTGGGTGCCTCAGAATCCGATACCCTCTGAATTGCTGGAAAATATACTGTGCGGTATTGCCTTTGGCCTGGGAATTCTAGGCATCATAGTGGGTGCTGCCCTCATCATCTACTTCCAAAAACCATGTGGAAGTGGTGCAGGTAA

>Saha_DAA_1

ATGATGCCCAACAAAGCTTTGATCCTAGGGACTTTCACCCTGGCCATGCTGCTGAATCCCTGGGGAGCCAGAGCCATTAAAGAGAATCATGTGATCATCCAAGCTGAGTTCTACCAGACCCACAACCCTTTAGGAGAGTTCATGTTTGATTTTGATGGGGATGAAATTTTCCATGTGGATTTGGACAAGAGAGAGACAGTCTGgcgtcttcctgacttcagcaaATTTGCCAGCTTTGAGGCTCAGGGTGCTCTGGCCAATCTTGCTGTGGACAAAGCCAATCTGGAAATCATGATAAAACGGTCCAACAACACTCCTGATACTAATGtGCCCCCTGAAGTGACAGTGTTTCCTAAGAGCCCAGCGGAGATGGACCAGCCCAATGTCCTTATCTGCTTCATTGACAAGTTCTCTCCTCCCGTACTTAATGTGACATGGCTTCGTAATGGGCAGCCCATCACTGATGGTGTGTTTGAGACTGTCTTCCTCCCCCGCCCTGATCATGCCTTCAGAAAATTCCACTACCTCACCTTCATCCCCTCTGCCAATGATTACTATGACTGTAAGGTCGATCACTGGGGACTGGAACAACCTCTTATGAAACATTGGGGTAAAAATACGAACCCCACTGCCAGAGACAACAGAGACTGTGGTCTGTGCCCTGGGCCTAGCCATTGGCCTAGTGGGCATCGTTGTGGGCACCATCTTGATTATCAAGGGCATGAAATCAAGCAACGCTTCCCGTGGTGGCCCTCGTGGACCCCTGTAA

>Saha_DMA_1

GGGACTTGAGCAAAACCTGGGAGTTACACTGTTACTACTGAAGCTACAGTCGTCATTGCTTTTGTCCCTGTTCTGGGAAGCCACTCCAGTGTTGGCCGTGTTTGGGAATAATTTACAGAATTACACATTCTCTCACATACTATTCTGCCAGAATGGAGAATCCTCTTTGGGTCTGTCAGAAAACTTCAATGGGGACTATCTCTTCTCCTTTGACTTCTCCAAGAACTCCCGGGTGCCCCGGCTGCCTGAATTTGCTGCTTGGGCCACTGATAAAAGAGACATCAAGACCATAGATGCTGACAAGAATCTTTGCCAAGAGCTGCAACATCAATTGAGTAGACTTTGTAAAGGACGGATCCCTGAGGCTAGAgGAAACCCTGTGGCTGAAGTTTTCACTCTGGAGCCCCTGGAGTTTGGGAAGCCCAACACTCTCGTTTGTTTTGTTAGCAATGTCTTCCCACCTCGTATAACCGTGACCTGGCAACATGAAGGAGTCTCTGTGGAAAGCAGCAGCCCCACCTTTCTCTCAGCTACAGATGGACTTGACTTCCAGGCCTTTTCTTATCTGAACATCACACCCACATCCACTGATATTTTCTCTTGCACTGTGACACAGGAAGGTGACCTCTTCAGCACTATAGCGTTTTGGGTGCCTCAGAATCCAATACCCTCTGCATTGTTGGAAAATATACTGTGTGGTATTGCCTTCGGACTGGGCATTGTTGGTATCATCGTAGGTGCTTCCCTCATCATCTACTTCCAAAAGCCATGTGTAAATGGTGCAGGTGA

>Mafu_DNAzoo_DAA_1

ATGACTTCCAACAAATCCTTGATCCTAGGAGCCTTCATTCTGTCAGTGCTGCTGGGTCCCTGGGGAGCCAGGGCCATTAAAGAGAACCATGTGATCATCCAAGCAGAGTTCTACCAGACCCACGAACCCTCTGGAGAGTTTATGTTCGACTTTGATGGGGATGAGATTTTCCATGTGGATTTGAACAAGAAAGAGACAGTCTGGCGGCTTCCTGACTTCAGCAAATTTGCCAGCTTTGAGGCTCAGGGTGCCTTGGCCAATCTTGCTGTGGACAAAGCCAACCTGGAAATCATGATGAAACGATCCAATAACACCCCTGACACCAATGTGGGCCCTGAAGTGACAGTGTTTCCCAAAGGCCCAGTGGAGCTGGGCCAGCCCAACATCCTTGTCTGCTTCATTGACAAGTTCTCTCCTCCGGTACTTACTGTGACCTGGCTTCATAATGGGGTTCCCATCACTGATGGTGTGTTTGAAACTGTCTTCCTCCCTCGCTCTGACCATGCCTTCAGAAAATTCCACTACCTCACCTTCATCCCCTCTGCCACCGATTACTATGACTGTCAGGTTGAGCACTGGGGACTGGAACAACCTGTTGTCAAACACTGGGAACCAGAAGTACGGACCCCACTGCCAGAGACAACAGAGACTGTGGTCTGCGCCCTAGGCCTGGCCATAGGCCTTGTGGGCATCATCGTAGGCACCATTCTTATTATCAAGGGCATGAGATCAAACAACACTTCCCGTGGTGGCTCCCGTGGACCCCTGTAA

>Mafu_DNAzoo_DBA_1

ATGGCTGCCAGCAGAGGGCTAATCCTAAGGACGCTGTCACTGGTTGTGCTGCTGAGTCCCCAAGGAACTTCTCAGTCCATTGAAGGCAACTGACCACGTGGGGATCTACGGCACAGGTGTATACCAGTCCTATGAGTCCTCAGGCCAGTACACACAGAAATTTGATGAGGACGAGCTGTTTTACGTAGACCTGCAGAAGAAGGAGACTGTGCGGCGGCTGCCAGAGTTTAGCCATTTCAGCAGCTTTGACCCTCAGGGAGGGCTGCGTGAAAAAGCCACATGCAAGTACAACCTGGACATCCTGATCAAGCGCTCCAACAGAAGCAGGGCCATCATGCCCCCTGAAGTGACTGTGTTCTCAGAGAGTCCTGTGGAGTTGGGCCAGCCAAATGTACTCATCTGCTTGGTGGACAACATCTTCCCTCCAGTGGTCAACATCAAGTGGCTTTGTAATGGCCAGGTGATCACCACTGGTGTGTCTGAGACAGACTTCTACTCTTGGCCTGACCACAAATTCCGCAAGTTCTACTACCTCACTTTTCTCCCCAACACAGAGGCCTTTTATGACTGCAAAGTGGAGCACTGGGGCTTGGAGTAGCCACTCCTCAAGCACTGGGAACCCCAGATTCCTTCCCCAGTGCCAGAGACAACAGAAACTGTGGTCTGTGCCCTTGGTCTGGCAGCGGGCCTGGTGGGCATCATTGTGGGCACCATCCTTATAATCAAAGGCATGCGTTCCAGCAGTAGGATCCAACATCAAGGTCCTCTGTGA

>Mafu_DNAzoo_DBA_2

ATGGCTGCCAACAGAGTTCTCATCCGAGGGACCCTCTCATTGGCTTTGCTCCTGAGCCCCCAAGGAGCTTAAGTCTATTGAAGCCGAACTTCTGACCATGTTGGGGTCTATGGCACAGATATATGCCAGAACTGTGGGCTCTCAGGCCAGTACGCACAAGAATTTGATGACGATGAGCTGTTTCATGTGGACCTGCAGAAGGAGACTATATGGTGGCTGCCAGAGTTTAGCCATTTTGCTGGCTTTGACCCTCAGGGAGGGTTAAGAAATGTATGCATAGCCAAACTCACCTTGGACACCCTCATCAAACACTCCAACAGAAGCAGGGCCATCAGGGTGCCCCCTGAGGTGACTGTATTCTCAGAGAACACTATGGAGATAGGCCAGACAGATGTACTCATCTGCTTGGTGGACAACATCTTCCCCTCAGCTGTCAATATCACGTGGCTTCGTGATGACCAGTTGGTCACCACTGGTGTATCTGAGACAGACTTTTACTCTCGGTCTGACCACAAATTCCACAAGTTCTACTACCTCACTTTCCTCCCCAACACAGAGGATTTTTATGACTGCAAAGTGGAGCACTGGGGCTTGGAGCAGCCAGTCCTCAAGCACTGGAACCCCAGATTCCATCCCCAGTACCAGAGACAACAGAAACTGTGGTCTGTGCCCTTGGTCTGGCAGTGGGCCTGGTGGGCATCATTGTGGGCACCATCCTTATAATCAGAGGCATGCGTTCCAGCTCTGGGATCCAACATCAAGGGCCTCTGTGA

>Mafu_DNAzoo_DBA_3

ATGGCTGCCAGCAGAGTTCTGATCCTAGGGACCCTCTCACTGACTGTGCTGCTGAGTCCCCAAGGAGCTTCTGAGCCCATCGAAGGCAACTGACCATGTGGGGCTCTATGGCACAGATATATACCAGACCTATGGGCCCTCAGGCCAGTACACACAGGAATTTGATGAGGACGAGCTGTTTTACATAGACCAGCAGAAGAGGGAGACTGTGTGGCGGCTGCCAGAGTTTAGCCATTTTGCTAGCTTTGACCCTCAGGGAGGACTGCGTGAAATAGCCATATGCAAGCACACCCTGGACATCCTGATCAAACGCTCCAACAGAAGCAGGGCCGTCATGCCGCCTGAAGTGACTGTGTTCTCAGAGAGTCCCGTGGAGGTGGGCCAGCCAAATACACTCATCTGCTTGGTGGACAACATCTTCCCTCCAGTAGTCAACATCACGTGGCTTCGTAATGGCCAATTGGTCACCATAGGTGTGTCTGAGACAGACTTCTACACTCGTCCTGACCACAAATTCCGCAAGTTCCACTACCTCGCTTTTCTCCCCAACACAGAGGATTTTTATGATTGCAAAGTGGAGCACTGGGGCTTGGAGCAGCCAGTCCTCAAGCACTGGGAACCCCAGGTTCCATCCCCACTGCCAGAGACAACAGAAACTGTGGTCTGTGCCCTTGGTCTGGCTGTGGGCCTGGTGGGCATCATTGTGGGCACCATCCTCATAATCAGAGGCATGCCTTCCAGCAGCAGGATCCAACATCAAGGACCTCTGTGA

>Mafu_DNAzoo_DBA_4

ATGGCTGCCAGCAGAGTTCTGATCTTAGGGACCCTCTCACTGGCTGTGCTACTGAGTCCCCAAGGAGCTTCTGAGTCCATTGAAGGCAACTGACCATGTTGGGCTCTATGGTACAGATATATACCAATCCTATGGGCCCTCAGGCCAGTACACACAGGAATTTGATGAAGATAAAGAATTTTATGTAGCCTTGCAGAAGAAGGAGACTGTGTGGTGGCTGCCAGAGTTTAGCCATTTTGCTCGCTTTGACCCTCAGGGAGGACTGCATGAAATATCCACAAGCAAGTACAACCTGGACATTGTGATCAAATGCTCCAACAGAAGCAGGGCCATCAGCGTGCCGCCTGAGGTGACTATGTTCTCAGAAAGCCTGTGGAGGTGATGGGCCAACCAAATATACTCATCCGATTGGTGAAAAACATCTTCCCTCCAGTGGTCAACATCACATGGCTTCTTAGTTGCAGTTGGTCACCATAGGTGTGTCTGAGACAGACTTCTACTCTCAGCCTGACCACAAATTCCGCAAGTTCTATTACCTCACTTTTCTCCCCAACACAGAGGATTTTTATAACTGCAAAGTGGAGCACTGGGGCTTAAAGCAGCCAGTCCTCAAGCACTGGGAACCCCAGTTTCCATCCCCATTGCCAGAGACAACAGAAACTGTGATCTGTGCCCTTGGTCTGGCAGTGGGCCTGGTGGGCATCGTTGTGTGCAGCATCCTCATAATCAGAGGCATGCATTCCAGCAGTAGGATCAAGGGCCTCTGTGA

>Mafu_DNAzoo_DBA_5

ATGGGTGCCAGCAGAGTCCTGATCCTAGGGACCCTCTCATTGGTTGTGCTGCTGAGTCCCCAAGGAACTTCTCAGTCCATTGAAGGCAACTGACCATGTGGGGATCTATGGCACAGCTGTATGCCAGTCCTATGGGCCCTCAGGCCAGTACACACAGGAATTTGATGGGGATGAAAAATTTTATGTAGACCTGCAGAAGAAGGAGACTGTGTGGAGGCTGCCAGAGTTTAGCCATTTTACCAGCTTTGACCCTCAGGGAGGACTGCGTGAAATAGCCACATGCAAGCACAACCTGGACATCCTGATCAAACGCTCCAACAGAAGCAGGGCCATCAGCGTGCCCCCTGAAGTGACTGTGTTCTCAGAGAGTCCTGTGGAGGTGGGCCAGCCAAACGTACTCATCTGCTTGGTGGACAACATCTTCCCTCCAGTGGTCAACATCAAGTGGCTTCGTAATGGCCAGGTCATCACCACTGGTGTCTCTGAGACAGACTTCTACTCTCGGCCTGACCACAAATTCCGCAAGTTCTACTACCTCATTTTTCTCCCCAACACAGAGGAATTTTATGACTGCAAAGTGGAGCACTGGGGCTTGGAGCAGCCAGTCCTCAAGCACTGGGAACCCCAGTTTCCATCCCCATTGCCAGAGACAACAGAAACTGTGGTCTGTGCCCTTGGTCTGGCAGTGGGCCTGGTGGGCATCATTGTGGGCACCATCCTTATAATCAGAGGCATGCGTTCCAGCAGTAGGATCCAACATCAAGGGCCTCTGTGA

>Mafu_DNAzoo_DBA_6

ATGGTTGTCCACAGAGTCCTGATCCTAGGGACCCTCTCACTGACTGTGCTTCTCAAGGAACTTCTGAGTCCATTGAAGTGACCATGTTGGTGTCTACAGCACAGGTAGGTACCAGACCTATGGGCCTTCAGGCCAGTACATACAGGAATTTGATGAGGATGAGCTATTTTATGTGGACCTGCAGAAAAAGGAGACTGTGTGGTGGCAGCCAGAGTTTAGCCATTTTGCTGAATCTGACCCTCAGGGAGGACTGAGAAACATAGCTGTCATGCAGCACAACCTGGACATCATGATCACATGCTCCAACAGAAGCAGGGCCATCAGCATGCCCCCTGAAGTGACTGTGTTCTCAGAGAATCCCGTGGAGATGGGCCAGCCAAACATATTCATCTACTTGGTGGACAATATCTTCCCTCCAGTGCTTAACATCACATGGCTTCATAATGGCCAGTTGATCACCTCTGGTATGTCTGAGACAGACTTCTTTTCTCAGCCTGACCACAAATTTCACAAGTTCTACTACCTCACTTTTCTCCCCAAAACACAGGATTTTTACGACTGCAAAGTGGAACACTGGGGGCGAGAGCAGCCAGTCCTCAAGCACTAGAAACCCCAGATTCCATCCCCAGTGCCAGAGACAACAGAAACTCTGGTCTGTGCCCTTGGATTGGCTGTGGGCCTGGTGGGCATCATTGTGGGCACCATCCTCATAATCAGAGGCATGTGTTCCAGGAGGAGGATCCAACATCAAGGGCCTCTGTGA

>Mafu_DNAzoo_DBA_7

ATGGTTGTCCACAGAGTCCTGATCCTAGGGACCCTCTCACTGACTATGCTTCCCAAGGAACTTCTGAGTCCATTGAAGCTGACCATGTTGGGGTCTACAGCACAGATATGTACCAGTCCTATGGGACTTCAGGCCAGTACACACAGGAATTTGATGAGGATGAGCTATTTAATGTGGACCTGCAGAAGAAGGAGACTGTGTGGTGGCAGCCAGAGTTTAGCCATTTTGCTGAATCTGACCCTCAGGGAGGACTGAGAAACATAGCTGTAATGCAGCACCGCCTGGACATCATGATCAAATGCTCCAACAGAAGCAGGGCCATCAGCAGTACCCCCTGAGGCGACTGTGTTCTCAGAGAATCCCGTGGAGATGGGCCAGCCGAACATATTCATCTACTTGGTGGACAATATCTTCCCACCAGTGCTTAACATCACATGGCTTCATAATGGCCAATTGATCACCTCTGGTGTGTCTGAGAAAGACTTCTATTCTCGGCCTGACCACAAATTTCGCAAGTTCTACTACCTCACTTGTCTCCCCAAAACACAGGATTTTTACGACTGCAGCGTGGAACACTGGGGCCTGGAGCAGCCAGTCCTCAAGCACTGGAAACCCCAGATTCCATCTCCAGTGTCAGAGACAGGAGAAACTCTGGTCTGTGCCCTTGGACTGGCTGTGGGCCTGGTGGGCATCATTGTGGGCACCATCTTCATAATCAGAGGCATGTATTCCAGCAGGACGATCCAACATCAAGGGTCTTTGTGA

>Mafu_DNAzoo_DCA_1_partial_exon_2-4

ACCATGGAATCCGCTCTGTGACAATTGTTCAGACCTACAAACCATCTGAGCAGGACCTGCATGAATTTGATGAAGATGAACCATTTCACATGGACTATGAAAAGAAGGAAACAGTCTGGCAGCTTCCTGAGTTTGGCCGTATCTTCAGTTCCAGTGCACAGATTGGGCTAGGTGACATTGCTGCGGACATGGCTAACCTGAACCAACTTATCAGGCAAACCAAGCACACCCAAGCCACCATTGTGACTCCAGAGGTGGCAATGTTTCCCAAGGAGGCCGTGGAACTGGAAGAACCCAGCGTCCTCATCTACCACATTGATAAGTTCTCCATCCCAGTGATCAATGTCACATGGCTGTGCAATGGTGAGTCAGTCACCACAGCGGTATCTGAAACTGTGTTCCTGCCTCAGGATGACTGTTCTTCCCACAAGTTTCATTACTTCACTTTCTTCCTCTCAACTGATGATATTTATGACTGTATAGTTGAACCCTGGGGCCTCAAAAACCACTTTCCAAGTGTAGCCTGAGACGCTAACACCACCGTCTGAGACAATGGAGATGCTCTTCTCTGTTCTAGGAATGGCTATGGGCCTGGTGGGCATCATGGGGGCTGCCAGCTTCATTATCAGAGGCTTGTGCTCAGGCAAATGGTTCCTGACCCCACAGATCAAGTATAATTTTCTGCAAGAAATCTTTCCAATCCTCCTTAATCTTAGTGCCTTCCCCTGA

>Mafu_DNAzoo_DMA_1

ATGGGACCTGTGCAAAGCCTGGGAGCTACACTGTTACTCCTGCAGCTGCAGTCATCACTGTTCTTGTCTCTGTCCTGGGGAGCCACTCCAGTGTTGGCATCATTACTGGGGAATAGCCTACAGAACTATACGTTCTCTCACACAATGTTCTGCCAGGATGAGCAACCCTCAGTGGGTCTGTCTGAGGTCTTCGATGGGGACCTGCTCTTCTCCTTCGACTTCTCCAGGAACACTCGGGTACCCCGGCTGCCTGAGTTTGGTGCTTGGGCCAGTGATGAGGGAGATGTCAAGATCATAGAAGATGACAAGACACTCTGCCAGGGATTGCAGAAATTTTTGAGTGAACATTTTGAAGGCCAAATCCCAGAGGCTAGAGGAAACCCTGTGGCTGAAGTTTTCACTCTGGAACCCCTGGAGTTTGGGAAGCCCAACACTCTCATTTGCTTTGTTAGTAACATCTTCCCACCTCAGATAACAGTGACTTGGCAGTACAAAGAAGTTCCTGTGGAAAGCAGCAGCCCCACTTTTCTCTCTGCTATAGATGGACTTGGCTTCCAGGCCTTCTCCTATCTGAATTTCACACCCACTGCCTCTGATATCTTCTCTTGCACTGTGGCACGGGAAGGCGAACTCTTCAGCACTATAGCCTTCTGGGTGCCTCAGAATCCGATACCCTCTGAATTGCTGGAAAATATACTGTGCGGTATTGCCTTTGGCCTGGGAATTCTAGGCATCATAGTGGGTGCTGCCCTCATCATCTACTTCCAAAAACCATGTGGAAGTGGTGCAGGTAA

>Psoc_DNAzoo_DAA_1

ATGGTCCCTGACAAAGCCTTGATCCTAGAAGCCTTCATCCTGGCAGTGCTGCTGAGTCCCTGGGGAGCCAAGGCCATTAAAGAGAACCATGTGATCATCCAGGCAGAGTTCTACCAGACTTCCAAACCCTTGGGAGAGTTCATGTTTGACTTTGATGGAGATGAGATTTTCCATGTGGATTTGGACAACAAGGAGACAGTCTGGAGGCTTCCCGACTTCAGCAAGTTTGCCAGTTTTGAGGCTCAGGGTGCTTTGGCCAATCTTGCTGTGGATAAAGCCAACCTGGAAATCATGATGAAACGGTCCAACCACACTCCTGACACCAATGTGCCCCCTGAAGTGACAGTATTTCCCAGGGGCCCAGTGGAGCTGGGACAGCCCAACGTCCTTGTCTGCTTTGTTGACAAGTTCTCTCCTCCTGTACTTACTGTGACATGGCTTCATAATGGGAATCCCATCACTGATGGTGTGTTTGAGACTGTCTTCCTTCCCCGCTCTGACCACACCTTCAGAAAATTCCACTACCTCACCTTCATCCCCTCTGCCACTGATTACTATGACTGCAAGGTCAATCACTGGGGACTGGAACAACCTGCTGTCAAACACTGGGAACCAGAAGTACGGACCCCACTGCCAGAGACAACAGAGACTGTGGTCTGTGCCCTGGGCCTGGCTATAGGCCTGATAGGCATCATCGTAGGAACCATCCTTATTATCAAGGGCATGAGATCAAACAACGCTTCCCGTGGTGGCTCCCGTGGACCCCTGTAA

>Psoc_DNAzoo_DCA_1_partial_exon_1_3-5

ATGGTGTCTGTGGAATTTCTTGGAGGCCTATTGATATGACAGTCCTATTGATAGTGCTGAGCACACCCATAGTGTGGGGCAGGGACATTCCAGGTAAGTCCGGCCCCAAGTGAAGGTTTTCCCCTTGGTACAACCGCTTGGGCACCACAAACCTGCTTCTCTGTTCTGCGACCTGTTTCTATCCTGGTGAGATCAAGATCAGCTGGTTCAGGAATGGAAAAGAGAAGACTAGGGTCATGTCCACAGGCCAAATCCAGAATGGTGACTGGAGCTTCCAGACCCTTGGGATGCTGGAAATGACCCCCCAAAGAGGAGATGTCTTTACTTGTCATGTGGACCATGTCAGCTTGCAGAGAGCCCTGTCACCATAGACTGGAGAGCACAGTCTGAATCTGCCTAGAAAGATGCTGACTGGAATTGGGGGCCTAGTGCTTGGACTATTCTTACTTGGAATAGAACTTGTCATCCACCTCAGAAATTTGAAAGATTCCTGTTCTGGGACCAAAGAAGATACAAATGTGGAAAGGATTGTGAACTTTGCAACAAGATTTTCCCAGAGCTGTTGCCCAGCCCTTGA

>Psoc_DNAzoo_DMA_1

ATGGGACCTGTGCAAATCCTGGGAGCTACACTGTTACTCCTGCAGCTACAGTCATCGCTGTTTTTGTTTCTGTCCTGGGGAGCCACTCCAGTGTTGGCATCATTATTAGGGAATAGCCTACAGAACTACACATTCTCTCACACACTGTTCTGCCAGGATGAGGAACCCTTCCTGGGTCTATCGGAGACCTTCAATGGGGACCAGCTCTTCTCCTTCGACTTCTCCAGGAGCTCTCTGGTGCCCCGGCTGCCTGAATTTTCTGCTTGGACTGGTGATGAAGGAGACATCGAGACCATAAAAAGTGATGGGAACCTCTGCCAGGAATTGCAAAAAGAATTGAGTAGAATTTTGGAAGGCCAAATCCCTGAGGCTAGAGGAAACCCTGTGGCTGAAATTTTCACTCTGGAGCCCCTGCAGTTTGGGAAGCCCAACACTCTCATCTGCTTTGTTAGTAACATCTTCCCACCTCAAATAACTGTGACCTGGCAGCTCGAAGGAGTTGCTGTGGAAAGCAGCAGCCCCACTTTCCTCTCAGCTGTGGATGGACTAGGCTTCCAGGCCTTCTCTTATCTGAACTTCACACCCACACCCTCGGATGTCTTCTCTTGCACTGTGGAACGGGAAGGTGACCTCTTCAGCACTATCACCTACTGGGTGCCTCAGGATCCAATACCCTCTGAATTGTTGGAAAATGTACTCTGTGGTATTGCCTTTGGCCTGGGAATCGTTGGCATCATAGTGGGTGCTGCCCTCATCATCTACTTCCGAAAACCATGTGCAAGTGGTGCAGGTG

>vour_DAA_1

ATGGCCCCCAACAAAGCCTTGCTCCTAGGAGTCTTCATCTTGGCAGTGCTACTGAGTCCCTGGGGTGCCAGAGCTGTTAAAGAGAAGCATGTGATCATCCAAGCAGAGTTCTACCAGACCCACGACCCCTCGGGAGAGTTCATGTTTGACTTTGATGGGGATGAGATTTTCCATGTGGATTTGAAGAGCAAAGAGACAGTCTGGAGGCTTCCTGACTTCAGCAAATTTGCCAGTTTTGAGGCTCAGGGTGCTCTGGCCAATCTTGCTGTGGACAAAGCCAACCTGGAAATCATGATGAAACGATCCAACAACACCCCTGACACCAATGTACCCCCTGAAGTGACAGTGTTTCCCAAGGGCCCAGTGGAGCTGGGTCAGCCCAACGTCCTTGTCTGCTTCGTTGACAAGTTCTCTCCTCCCGTACTTACTGTGACATGGCTTCATAATGGGCGTCCCATCACTGATGGTGTGTTTGAGACTGTCTTCCTCCCCCGCCCCGACCATTCCTTCAGAAAATTCCACTACCTCACTTTCATCCCCTCTGCCACCGATTACTATGACTGCAAGGTCGAGCACTGGGGACTGGAACAGCCCACTGTCAAACATTGGGAACCAGAAGTACGGACCCCACTGCCAGAGACAACAGAGACTGTGGTCTGTGCCCTGGGCCTGGCCATAGGCCTGGTGGGCATCATCATAGGCACCATCCTTATTATTAAGGGCATGCGAGCAAGCAACGCTTCCCGTGGTGGTCCCCGTGGAACCCTGTAA

>vour_DBA_1

ATGGCTGCCAACAGAGTCCTGATCCTGGGGACCCTCTCACTGGCCATGTTGCTGAGTCACCAAGGAGCTTCTGAGTCCATTGAAGAGCTGACCATCTGGGGGTGTATGGCACAACCATGTACCAGTCCTATGGGTCCTCAGGCCAGTATACACATGAATTTGATGAGGATGAAGAGTTTTATGTGGACCTGCAGAAGAAGGAGACTATATGGAGGCTGCCAGAGTTTGGCCATTTTACTAGCTTTGACCATCACACTGCGCTGAGAAACATAGCCGTAGCCAAGTATAACTTGCACCTCCTGATCAAATGCTGCAACAGAACCAGGGCCATTAATGTGCCCCCTGAGGTGACTGTGTTCTCAGACAGTCCCGTGGAGATGGGCCAGCCGAACATACTCATCTGCTTGGTGGACAACATCTTCCCCCCAGTGGTCAACATCACGTGGCTTCGTAATGGGCAGTTAGTCACTGCAGGTGTGTCTGAGACAGACTTCTACCCTCGACCTGACCACAAATTCCGCAAGTTCCACTACCTCGCTTTTCTCCCCAACACAGAAGATTTTTATGACTGCAAAGTGGAGCACTGGGGCCTGGAGCAGCCAGTCCTCAAGCACTGGGAACCCCAGGTTCCATCCCTGCTGCCAGAGACAACAGAAGCTGTGGTCTGTGCCCTTGGTCTGGCTGTGGGTCTGGTGGGCATCGTTGTGGGCACTGTCCTCATAATCAGGGGCATACATTCCAGCAGTAGGACCCAACATCAAGGGCCTCTGTG

>vour_DCA_1

ATGACTTCAAGTGTAGACTCAGTCTTGGGGATCCTCATCCTGGCTGCCCGGTTGATCAAACAAAGAACCAGGGCCACTAATGACCATGTAATCAGCTCTGTGACATTTGTTCAGACCCACAAACCATCTGGGCAGTACCTGCACAAGTTTGATGAGCATGAGCAATTCCATGTGGACTTTGACCAGAAGGAAACAGTCTGGTGGCTTCTTAAGTTTGGCCATATCTTCAGTTTTGATGCACAGATTGGGCTAGGTGACATCGCTGTGGACGTGGCTAACTTGAACCAACTTATCAAGTAAACCAACCACACCCAGGCCACCATTGCCACTCCAGAGGTGACAGTGTTTCCCAAGGAGCCCATGGAACTGGAAGAACACAACATCCTCATCTGCCACATTGATAAGTTCTCACCCCCAGTGATTAATGTCACATGGCTGTGCCATGGAGAGCCAGTCACCACAGGGGTATCTGAGACCACCTTCATGCCTCGGGATGACTATTCTTTCCTCAAGTTCCATTATCTCACTTTCCTTCCCCCAACTGATGATGTCTATGACTGTGTGGTTGAACACTGGGGCCTGGAAAAGCCACTTTTCAAGCATTGGGAGCCTGAGATGCTGACACCACCATCTGAGCCAATGGAGACACTCCTCTATATTATAGGCCTGGCTGGGGGCCTGGTGGGCATCACTGTGGCTGCCACCCTTATTATGAGAAGCTTGCACTTAGGCAAATCACACCTGACCCCACAGATCAAATCTTTTCCAGTCCTACTTAATCTTACTGCCTTCCCCTGA

>vour_DMA_1

ATGGGACCTGAGCAAAGCCTGGGAGCTGCACTGTTACTGCTGCAGCTGCAGCCATCACTGCTTTTATCCCTGTCCTGGGGAGACACTCCAGTGTTGGCATCATTACTGGGGAAGAGCCTACAGAACTACACATTCTCTCACACACTGTTCTGCCAGGATGAGGAACCTGTCCTGGGTCTGTCTGAGGCCTTCAATGAGGACCAGCTCTTCTCCTTTGACTTCTCCAGGAACTCCCGGGTACCCCGGCTCCCTGAGTTTGCTGCTTGGGCCAGTGACAAGAGAGATATTAAGGCCATACAAGCCGACCAGCAGCTCTGCCAGGAACTGCAAAAAGAATTGAGTTCAGCTTTGGAAGGCAAAATCCCTGAGGCTAGAGGAAACCCTGTGGCTGAAGTTTTCACTCTGGAGCCCCTGGAGTTTGGGAAGCCCAACACTCTCATCTGCTTTGTTAGTAACATCTTCCCACCTCAAGTAACTGTGAGCTGGCAGCACCACCAAGTCCCTGTGCAAAGCAGCAGCCCCACTTTTCTCTCAGCTATCGATGGACTTGGCTTCCAGGCCTTCTCTTATCTGAACTTCACACCCACATCCTCTGATGTCTTCTCTTGCATTGTGACACGGGAAGGTGACCTCTTCAGCACTATAGCCTTCTGTGTGCCTCAGAATCCAATACCCTCTGAATTGTTGGAAAATATACTCTGTGGCATTGCCTTTGGCCTGGGAATTGTTGGCATCATAGTGGGTATTGCACTCATCATCTACTTCCAAAAACCATGTGCAAGTGGTGCAGGTAA

>Bepe_DAA_1

ATGACGCCCAACAAATCCTTGATCCTAGGAGCCTTCATTCTGTCAGTGCTGCTCAGTCCCTGGGGAGCCAGGGCCATTAAAGAGGACCATGTGATCATCCAAGCAGAGTTCTACCAGACCCACGAACCCTCTGGAGAGTTTATGTTCGACTTTGATGGGGATGAGATTTTCCATGTGGATTTGCAGAAGAAAGAGACAGTCTGGCGGCTTCCTGACTTCAGCAAATTTGCCAGCTTTGAGGCTCAGGGTGCTTTGGCCAATCTTGCTGTGGATAAAGCCAACCTGGAAATCATGATGAAACGGTCCAATAACACCCCTGACACCAATGTGGGCCCTGAAGTGACAGTGTTTCCCAAAGGCCCAGTGGAACTGGGCCAGCCCAACATCCTTGTCTGCTTCATTGACAAGTTCTCTCCTCCGGTACTTACTGTGACCTGGCTTCATAATGGGGTTCCCGTCACTGATGGTGTGTTTGAAACTGTCTTCCTCCCTCGCTCTGACCATGCCTTCAGAAAATTCCACTACCTTACCTTCATCCCCTCTGCCACCGATTACTATGACTGCAAGGTCGAACACTGGGGACTGAAACAACCTACTGTCAAACACTGGGAACCAGAAGTACGGACCCCACTGCCAGAGACAACAGAGACTGTGGTCTGTGCCCTAGGCTTGGCCATAGGCCTTGTGGGCATCATCGTAGGCACCATTCTTATTATCAAGGGCATGCGATCAAACAACACTTCCCGTGGTGGCTCCCGTGGACCCCTGTAA

>Bepe_DBA_1

ATGGCTGCCAACAGAGTTGTGATACTAGGGACCATCTCATTGGCTGTGCTGCTGAGTCCCCAGGGAGCTTCTGAGTTATGACAAAACTGATCATGTGGGGATCTTTGGCACAGCTATATACCAGTCCTATGGGCCTTTGGGCCAGTACACACACGAATTTGATGAGGACGAGCTGTTTTATGTGGACCTGAAAAAGAAGGAGACTGTGTGGCGGCAGCCAGAATTTAGCCATTATACTAGCTTTGACCCTCAGGAAGGGCTGAGAATCATAGCCATAGCCAAGAAGAGTCTGGATTTTCTGATCAAACATTCCAACAGAAGCAGCACCATCTCTGTGCCCCCTGAGGTGACTGTGTTCTTGGAGAGTCCCGTGGAGATGGGCCAGCCAAATGTACTCATCTGCTTGGTGGACAATATCTTCCCCCCAGTGGTCAACATCACATGGCTTCGTAATGGGCAGTTAATCACCATTGGTGTGTCTGAGACAGACTTTTACCCTCGGCCTGACCACAAATTCCGCAAGTTCTACTACCTCACTTTTCTCCCCAACACAGAAGATTTTTATGACTGCAAAGTGGAGCACTGGGGCTTGGAGCAGCCAGTCCTCAAGCACTGGGAACCCCAGGTTCCATCCCCACTGCCAGAGACAACAGAAACTCTGGTCTGTGCCCTTGGTCTGGCAGTGGGTCTGGTGGGTATCATTGTGGGCACCATCCTCATAATCAAAGGCATGCGTTCCGGTAGTAGGATCCAACATCAAGGGTCTCTGTGA

>Bepe_DBA_2

ATGGCTGCCAGCACCGTGCTGATCCTAAGGACCCTCTCACTGGTTGTGCTGCTGAGTCCCCAAGGAACTTCTCAGTCCATTGAAGGCAACTGACCACGTGGGGATCTATGGCACAGGTGTATACCAGTCCTATGAGTCCTCAGGCCAGTACACACAGGAATTTGATGAGGATGAGCTGTTTTACGTAGACCTGCAGAAGAAGGAGACTGTGTGGCGACTGCCAGAGTTTAGCCATTTTAGCAGCTTTGACCCTCAGGGAGGGCTGCGTGAAAAAGCCACATGCAAGTACAACCTGGACATCTTGATCAAACGCTCCAACAGAAGCAGGGCCATCAGTGTGCCCCCTGAAGTGACTGTGTTCTCAGAGAGTCCTGTGGAGTTGGGGCAGCCAAACGTACTCATCTGCTTGGTGGACAACATCTTCCCTCCAGTGGTCAACATCAAGTGGCTTCGTAATGGCCAGGTGATCACTACTGGTGTGTCTGAGACAGACTTCTACTCTCGTCCTGACCACAGATTCCGCAAGTTCTACTACCTCACTTTTCTCCCCAACACAGAGGACTTTTATGACTGCAAAGTGGAGCATTGGGGCTTGGAGCAGCCACTCCTCAAGCACTGGGAACCCCAGATTCCATCCCCACTGCCAGAGACAACAGAAACTGTGGTCTGTGCCCTTGGTCTGGCAGTGGGCCTGGTGGGCATCATTGTGGGCACCATCCTTATAATCAGAGGCATGCGTTCCAGCAGTAGGATCCAACATCAAGGGCCTCTGTGA

>Bepe_DCA_1_partial_exon_2-4

ACCATGTAATCAGCTCTGTGACAATTGTTCAGACCTACAAACCATCTGAACAGTACCTGCATGAATCTGATGAAGATGAACCATTCCACATGGACTGTGAAAAGAAGGAAACTGTCTGGCAGCTTCCTGAGTTTGGTCATATCTTCAGTTCCAATTCAGTTCAGATTGGGCTAAGTGACATTGCTGTGGACATGGCTTACTTGAACCAACTTATCAGGCAAACCAACCACACCCAAGCCACCATTGTGACTCCAGAGGTGGCAGTGTTTCCCAAGGAGGACGTGGAACTAGAAGAACCCAGTGTCCTCATCTGCCACATTGATAAGTTCTCCATCCCACTGATCAATGTCACATGGCTGTGCAATGGTGAGTCAGTCACCACAGGAGTATCTGAGACTGCCTTCCTGCCTCAGGATGACTGTTCTTTCCACAAGTTTCATTACCTCACTTTCTTCCTTTCAACTGATGATATTTATGACTGTGCACTTGAACACTGGGGCCTCAAAAACCACTTTTCAAGTAGCCTGAGATGCTAACACCACCATCTGAGACAATGGAGATGCTTATCCCTCTTCTAAGAATGGCTGTGGGCCTGGTGGGCATCATGGTGGCTGCCAGCTTCATTATCAGAGGCTTGTGCTCAGGCAAATGATTCCTGATCCCATAGATCAAGTATAATTTTCTGCAAGAAATTTCTGCAAGAAATCTTTCCAGTCCTCCTTAATCTTGGTGCCTTCCCCTGA

>Bepe_DMA_1

ATGGGACCCGTGCAAACCCTGGGAGCTACACTGTTACTCCTGCAGCTGCAGTCATCACTGTTCTTGTCCCTGTCCTGGGGAGCCACTCCAGTGTTGGCATCATTACTGGGGAATAGCCTACAGAACTATACATTCTCTCACACACTGTTCTGCCAGGATGAGCAACCCCTTCTGGGTCTGTCCGAGGTCTTCGATGGGGACCTGCTCTTCTCCTTCGACTTCTCCAGGAGCACTCGAGTACCCCCGGCTGCCTGAGTTTGGTGCTTGGGCCAGTGATGAGGGAGATGTCAAGATCATAGAAGATGACAAGATGCTCTGCCAGCAATTGCAAAAAGTTTTGAGTAGGATTTTGGAAGGCCAAATCCCAGAGGCTAGAGGAAACCCTGTGGCTGAAGTTTTCACTCTGGAGCCCCTGGAGTTTGGGAAGCCCAACACTCTCATCTGCTTTGTTAGTAACATCTTCCCACCTCAGATAACAGTGACTTGGCAGTACAAAGAAGTTCCTGTGGAAAGCAGCAGCCCCACTTTTCTCTCTGCTATAGATGGACTTGGCTTCCAGGCCTTCTCCTATCTGAATTTCACACCCACTGCCTCTGATATCTTCTCTTGCACTGTGGCACGGGAAGGCGACCTCTTCAGCACTATAGCCTTCTGGGTGCCTCAGAATCCGATACCCTCTGAATTGCTGGAAAATATACTGTGTGGTATTGCCTTTGGCCTGGGAATTCTAGGCATCATAGTGGGTGCTGCCCTCATCATCTACTTCCAAAAACCATGTGGAAGTGGTGCAGGTAA

>Bepe_DMA_2

ATGGGACCCGTGCAAACCCTGGGAGCTACACTGTTACTCCTGCAGCTGCAGTCATCACTGTTCTTGTCCCTGTCCTGGGGAGCCACTCCAGTGTTGGCATCATTACTGGGGAATAGCCTACAGAACTATACATTCTCTCACACACTGTTCTGCCAGGATGAGCAACCCCTTCTGGGTCTGTCCGAGGTCTTCGATGGGGACCTGCTCTTCTCCTTCGACTTCTCCAGGAGCACTCGAGTACCCCGGCTGCCTGAGTTTGGTGCTTGGGCCAGTGATGAGGGAGATGTCAAGATCATAGAAGATGACAAGATGCTCTGCCAGCAATTGCAAAAAGTTTTGAGTAGGATTTTGGAAGGCCAAATCCCAGAGGCTAGAGGAAACCCTGTGGCTGAAGTTTTCACTCTGGAGCCCCTGGAGTTTGGGAAGCCCAACACTCTCATCTGCTTTGTTAGTAACATCTTCCCACCTCAGATAACAGTGACTTGGCAGTACAAAGAAGTTCCTGTGGAAAGCAGCAGCCCCACTTTTCTCTCTGCTATAGATGGACTTGGCTTCCAGGCCTTCTCCTATCTGAATTTCACACCCACTGCCTCTGATATCTTCTCTTGCACTGTGGCACGGGAAGGCGACCTCTTCAGCACTATAGCCTTCTGGGTGCCTCAGAATCCGATACCCTCTGAATTGCTGGAAAATATACTGTGTGGTATTGCCTTTGGCCTGGGAATTCTAGGCATCATAGTGGGTGCTGCCCTCATCATCTACTTCCAAAAACCATGTGGAAGTGGTGCAGGTAA

>anst_DAB_1

ATGGTGTGTGTCTTGCTCCCCAAGGGCATCTGGAAAGAAGTTCTGACTGTGGCCCTGTTGGTGCTGAATCCCCAGGTGGCTGCAGGCAGACACAGCCCAAAGCACTTCACAAAACAGTCAAAGTGCGAGTGTTACTTTGTGAATGGGACAGAGCATGTGCAGTATGTGGAGAGACACATGTACAACCAGAAGGAATATGTGCGCTTTGACAGCAATGTGGGGAAATATGCTGCAGTGATGGAGCTGGGCCGACCAGAGGCTGAATACTGGAACAACCATAAGGAGATTCTAGATGACTTACGGGCCCGGGTGGACACCTTGTGCAGACACAACTACCAGATTTTTGAGCCCTTCTTGTTGTCCAGGAGTGGTGACCTGAAGTGATTGTGTATCCATCAAAGACAGCTCCCCTGGGACACCACAACCTGCTTGTCTGCTCTGTCAGCGGTTTCTACCCCGGGGACATCGAGGTCCGGTGGTTCCTGAATGGGCGGGAGGAGACAGCCGGGGTGGTGTCCACAGGCTTAATCAGCAATGGGGACTGGACCTACCAGTTACAGGTGATGCTGGAAATGATCCCCAAGAGGGGAGATATCTACACCTGCCAAGTGGAGCACAGCAGCCTTCAGAGACCCGTCCTCTTGGACTGGAAAGCCCAGTCTGAATCTGCCCAGAGAAAGATGCTGAGTGGAGTTGGGGGCATCGTTCTGGGTTTGATCTTCTTTGGAGTTGGTCTCATTGTCCACAAGAGAAGTCGGAAAGGTGA

>anst_DAB_10_partial_exon_2-4

AGAACTTCATGGAGCAGACGAAGGCGGAGTGTCACTTTGTCAATGGGATTGAGCACGTGCAGTTTGTGGGGAGACTCATATACAACCACGAGGAGATCCTCCGCTTTGACAGCGAAGTGGGGAAGTTCGTGGCTTTGACCGAGCTGGGACGGCCCATTGAGGAGCTAATGAACAGTCAACTGGAGGCTCTGGAGCAAGCGCGGGCCCAGGTGGCCATGTGCAGAGACAACTATATCCTGTGGGAGTCCTTGTGGAATCGGAGGAGAGTTGAGCCTGAAGTAACTGTGTATTCATCAAAGATAACTCCCCTAGGATACCCAAACCAGCTTATCTGTTTTATTACTGGTTTTTATCCTGGGGACATTGAGGTCAGATGGTTCCTGAATGGTCAAGAGGAGACAGCTGGGGTTTTGTCCACAGGCCTGATTAACAATGGAGACTGGACCTTTCAGACTCAGGTTATGCTGGAAATGATCCCCAATCCTGGAGATGTCTACACTTGCCAAGTGGAGCACTCCAGCCTTCAGAACCCTGTCATCGGAGTTTGGGAAGCACAGTATGGATCTGCCCAGAAAAAGATGCTGAGTGGAGTTGGGGGCCTTGTACTAGGGCTGATTTTCCTTGGGGTTGGCCTTGCTGTTTATCTTAAAAGTCAAAAAGGTGA

>anst_DAB_11_partial_exon_2-4

AGCACTTCACGGAGCAGGCAAAGTGCGAGTGTCACTTCGAGAACGGGACGGAGCACGTGCGGTATGTGGAGAGATACATATACAACCGGGAGGAGTACGTGCGCTTCGACAGCGACGTGGGGGAGTACGTGGCGGTGACGGAGCTGGGGCGGGGCATTGCGGAGTACTATAACAGCCAGAAGGAGATCCTGGAGGATGAACGGACCCGGGTGGACACTTTCTGCAGATACAACTACGGAATATCTGAGCACTTCTTAGTGCCCAGGAGCGTCCAGCCCCAGGTGACCGTGTATCCATCCAAGGTGGCTCCCCCGGGACACCACAACCTGCTTGTCTGCTCCGTCCGCGGTTTCTATCCCGGGGACATCGAGGTCCGGTGGTTCCTGAACGGGCGGGAGGAGACGGCTGGGGTGGTGTCCACGGGCCTGATGGGCAACGGAGACTGGACCTACCAGACCCTGGTGATGCTGGAGATGACCCCCCGGCGCGGAGATGTCTACACCTGCCACGTGGAGCACTCCAGCCTTCAGGGACCCGTCCTCTTGGCCTGGAGAGCCCAGTCTGAGTCTGCCCAGAGTAAGATGCTGAGCGGGGTCGGGGGCCTCGTGCTGGGGCTGATCTTCTTGGGGGTCGGCCTCATTGTCCACAAGAGGAGCCAGAAAGGTGA

>anst_DAB_2

ATGGGGTGTGTCTTGCTCCCCAGGGAAGTCTGGATAGAAGTTCTGGCTGTGACCCTGCTAATGCTGAATCCCCAGGTGGCTGCAGGCAGACACAGCCCAGAGCACTTCAGGTGGCTGGGAAAGGCCGAGTGTTACTTCGAGAACGGGACGGAGCACGTGCGGTATGTGGAGAGATACATCTACAACCGGGAGGAGTACGTGCGCTTCGACAGCGACGTGGGGGAGTTCGTGGCGGTGACGGAGCTGGGGCGGCGGGATGCGGAGTACTGGAACAGCCAGAAGGAGATCCTGGAGAGGAAACGGGCCTCGGTGGACTGGTTATGCAGGGTCTGCTATGAGATTTCTGAGCCCTTCTTAGTGCGCAGGAGCGTCCAGCCCCAGGTGACTGTGTATCCATCCAAGGTGGCTCCCCCGGGACACCACAACCTGCTTGTCTGCTCCGTCAGCGGTTTCTATCCCGGGGACATCGAGGTCCGGTGGTTCCTGAACGGGCGGGAGGAGACGGCCGGGGTGGTGTCCACGGGCCTGATGGGCAACGGAGACTGGACCTACCAGACCCTGGTGATGCTGGAGGTGACTCCCCGGCGCGGAGATGTCTACACCTGCCACGTGGAGCACTCCAGCCTTCAGGGACCCGTCCTCTTGGCCTGGAGAGCCCAGTCTGAGTCTGCCCAGAGTAAGATGCTGAGCGGGGTCGGGGGCCTCGTGCTGGGGCTGATCTTCTTGGGGGTCGGCCTCATTGTCCACAAGAGGAGCCAGAAAGGTGA

>anst_DAB_3

ATGGGGTGTGTCTTGCTCCCCAAGGAAGTCTGGAGAGAAGTTCTGGCTGTGACCCTGCTGGTACTGAATTCCCAGATGGCTGCAGGCAGACACAGCCCAGAGCACTTCACGGGGCAGTTTAAGGGCGAGTGTTACTTCGAGAACGGAACGGAGCACGTGCGGTTTGTGCAGAGACTCATCTACAACCCGGAGGAGTACGCGCGCTTCGACAGCGACGTGGGGAAGTACGTGCCGGTGACGGAGCGGGGGCGGCGCAGTGCGGAGTACTGGAACAGCCAGAAGGAGCTCCTGGAGAGGAGACGGGCCGAGGTGGACACTTACTGCAGGCACAACTACGGAGTGTTTGAGCCCTTCTCAGTGCGCAGGAGCGGAGCCCAGTCTGAGACGGCCCAGAGTAAGATGCTGAGCGGAGTCGGGGGCCTCGTGCTGGGGCTGATCTTCTTGGGGGTCGGCCTCATTGTCCACAAGAGGAGCCAGAAAGGTGA

>anst_DAB_4

ATGGTGTGTGTCTTGCTCCCCAGGGAAGTCTGGATACAAGTTCTGGCTGTGACCCTGCTGGTGCTGAATCCCCAGGTGGCTGCAGGCAGACACAGCCCAGAGCACTTTACAGAGCAGTTGAAGTCCGAATGTTACTTCGAGAACGGGACGGAGCACGTGCGGTTTGTGGAGAGAGGTATCTCCAACGGGGTGGAGATTGTGCGCTTCGACAGCGACGTGGGGGAGTACGTTGCGGTGACGGAGCTGGGGCGGCCGGATGCTGAGTATTGGAACAGCCAGAAGGAGTACATGGACTTGAAACGGGGCCAGGTCGACAATTACTGCAGGCACAACTACGAAGTGATTGAACCCTTCTCAGTGCGCAGGAGCGTCCAGCCCCAGGTGACTGTGTATCCATCCAAGACGGCTCCCCCCGGACACCACAACCTGCTTGTCTGCTCTGTCAGCGGTTTCTATCCCGGGGACATCGAGGTCCGGTGGTTCCTGAACGGGCGGGAGGAGACGGCCGGGGTGGTGTCCACGGGCCTGATGCGCAATGGAGACTGGACCTACCAGACCCTGGTGATGCTAGAGGTGACCCCCCGGCGCGGAGATGTCTACACCTGCCACGTGGAGCACTCCAGCCTTCAGGGACCCGTCCTCTTGTCCTGGAAAGCCCAGTCTGAGTCTGCCCAGAGTAAAATGCTGAGCGGAGTCGGGGGCCTCGTGCTGGGGCTGATCTTCTTTGGGGTCGGCCTCATTGTCCACAAGAGGAGCCAGAAAGGTGA

>anst_DAB_5

ATGGCGTGTGTCCTGCTCCCCAGGGAAGTCTGGATACAAGTTCTGGCTGTGACCCTGCTGGTGCTGAATCCCCAGGTGGCTGCAGACAGACACAGTCCAGAGCACTTCATGCGACAGTTTAAGAGCGAGTGTTACTTCGAGAACGGGACGGAGCACGTGCGGCTTGTGGTGAGACTCATCCACAACCGGGAAGAGATTGCGCGCTTCGACAGCGACGTGGGGAAGTACGTGGCGGTGACGGAGCTGGGGCGGAGCAGTGCGAAGGAACGGGACAGCAATAAGGATTACATGGAGTCATTACGGGCCGCGGTGGACACTTACTGCAGGCACAACTACAGAATATCTGAGCACTTCTTAGTGCGCAGGAGTGTCCAGCCCCAGGTGACTGTGTATCCATCCAAGGTGGCTCCCCCGGGACACCACAACCTGCTTGTCTGCTCCGTCAGCGGTTTCTATCCCGGGGACATCGAGGTCCGGTGGTTCCTGAACGGGCGGGAGGAGACGGCCGGGGTGGTGTCCACGGGCCTGATGGGCAACGGAGACTGGACCTACCAGACCCTGGTGATGCTGGAGATGACCCCCCGGCGCGGAGATGTCTACACCTGCCACGTGGAGCACTCCAGCCTTCAGGGACCCGTCCTCTTGTCCTGGAGAGCCCAGTCTGAGTCTGCCCAGAGTAAGATGCTGAGCGGAGTCGGGGGCCTCGTGCTGGGGCTGATCTTCTTGGGGGTCGGCCTCATTGTCCACAAGAGGAGCCAGAAAGGTGA

>anst_DAB_6

ATGGGGTGTGTCCTGCTCCCCAGGGAAGTCTGGATAGAAGTTCTGGCTGTGACCCTGCTGGTGCTGAATTCCCAGGTGGCTGCAGGCAGACACAGCCCAGAGCACTTCTTAGGTGCAGAGTAAGGCCGAGTGTCACTTCGAGAACGGGACGGAGCACGTGCGGTTTGTGCATAGATACATCTACAACCCGGAAGAGATTGCGCGCTTCGACAGCGACGTGGGGGAGTACGTGGCAGTGACGGAGCTGGGGCGGAGCATTGCTGAGTATTGGAACAGCCAGAAGAATTACATGGAGAGGGCAAGGACCGCAGTGCACTGGTTTTGCAGGGTCTCCTATGAGATTTCTGAGCCCTTCTTAGTGCGCAGGAGCGCATCACAACCTGCCTGTCTGCTCCAATGCTCCGTCAGCTGTTTCTATCCCGGGGACATTGAGGTCCGGTGGTTCCTGAATGGGCGGGAGGAGACGGCAGGGTTGGTGTCCACGGGCCTGATGGGCAATGGAGACTGGACCTACCAGACCCTGGTGATGCTGGAGATGACCCCCCGGCGCGGAGATGTCTACACCTGCCACGTGGAGCACTCCAGCCTTCAGGGACCCGTCCTCTTGGACTGGAAAGCCCAGTCTAAGTCTGCCCAGAATAAGGTGCTGAGTGGAGTCGGGGACCTCCTGCTGGGGCTGATCTTCTTTGGGGTCGGTCTCATTGTCTACAAGAGGAGCCAGAAAGGTGA

>anst_DAB_7

ATGGTGTGTGTCTTGTTCCCCAAGAAAGTCTGGATAGAAGTTCTGACTGTGACCCTCCTGGTGCTGAATTCCCAAGTGGCTGCAGGCAGACACAGTCCAGAGCACTTCACGAAGCAGAGCAAGTTCGAGTGTTACTTCGAGAACGGGACGGAGTACGTGCGGTATGTGCACAGATCTATCTACAACCAGAAAGAGTATTTGCGCTTCGACAGCGACGTGGGGGAGTACGTGGCATTGACCGAGCTGGGGCGGGGCATTGCCGAGAACTATAACAGCCAGAAGGATTACATGAAAAGGAAACGGACCGCAGTGAATTGGTTATGCCGGCACAACTACGGAGAGATTGAGCCCTTCTCAGTGCGCAGGAGCGTCCAGCCCAAGGTGACTGTGTATCCATCCAAGATGGCTCCCCCGGGACACCACAACCTACTTGTCTGCTCTGTCAGAGGTTTCTATCCCGGGGACATCGAGGTCCGGTGGTTCCTGAATGGGAGGGAGGAGACGGCCGGGGTGGTGTCCACGGGCCTGATGGGCAATGGAGACTGGACCTACCAGACCCTGGTGATGCTGGAGATGAGCCCCCGGCGCGGAGATGTCTACACCTGCCACGTGGAGCACTCCAGCCTTCAGGGACCCGTCCTCTTGTCCTGGAAAGCCCAGTCTAAATCTGCCCAGAGAAAGATACTGATTGGAGTTGGGGGCCTGGTGATGGGGCTGATCTTCTTTGGGGTTGGCCTCATTGTCCACAAGAGGAATCAGAAAGGTGA

>anst_DAB_8_partial_exon_2-4

AGCACTTCACTTTGTATTCGACCTCTGAATGTTACTTCAAAAACGGGACGGAGCGCGTGCGGTTTGTGGAGAGATACATCTACAATCGGGAGGAGTACGTGCGCTTCGACAGCGACGTGGGGGAGTACGTGGCGGTGACGGAGCTGGGGCGGCCCACTGCGGAGAAGTGGAACAGCCAGAAGGAGAACCTGGAGGATGAACGGGCCCAGGTGGACATTTACTGCAGATACAACTACGGAATACTTGAGCGCTTCTTAGTGCCCAGGAGCGTCCAGCCCCAGGTGACTGTGTATCCATCCAAGGTGGCTCCCCCCGGACACCACAACCTGCTTGTCTGCTCCGTCCGCGGTTTCTATCCCGGGGACATCGAGGTCCGGTGGTTCCTGAACGGGCGGGAGGAGACGGCCGGGGTGGTGTCCACGGGCCTGATGGGCAACGGAGACTGGACCTACCAGACCCTGGTGATGCTGGAGGTGACTCCCCGGCGCGGAGATGTCTACACCTGCCACGTGGAGCACTCCAGCCTTCAGGGACCCGTCCTCTTGGACTGGAGAGCCCAGTCTGAGTCTGCCCAGAGTAAGATGCTGAGCGGAGTCGGGGGCTTCGTGCTGGGGCTGATCTTCTTGGGGGTCGGCCTCATTGTCCACAAGAGGAGCCAGAAAGGTGA

>anst_DAB_9_partial_exon_2-4

AGCACTTCACGCTGCAGGCAAAGTTCGAGTGTTACTTCGAGAACGGGACGGAGCACGTGCGGCTTGTGGAGAGACACATCTACAATGGGGAGGAGAATGTGCGCTTCGACAGCGACGTGGGGGAGTACGTGGCGGTGACGGAGCGGGGGCGGCCGGAGGCGGAGTCTTGGAACAGCCAGAAGGAGATCCTGGAGGAGAGACGGGCCGCGGTGGACACTTACTGCAGATACAACTACGGAGTGTTTGAGCCCTTCTCAGTGCGCAGGAGCGTCCAGCCCCAGGTGACTGTGTATCCATCCAAGGTGGCTCCCCCGGGACACCACAACCTGCTTGTCTGCTCCGTCAGCGGTTTCTATCCCGGGGACATCGAGGTCCGGTGGTTCCTGAACGGGCGGGAGGAGACGGCCGGGGTGGTGTCCACGGGCCTGATGGGCAATGGAGACTGGACCTACCAGACCCTGGTGATGCTGGAGGTGACCCCCCGGCGCGGAGATGTCTACACCTGCCACGTGGAGCACTCCAGCCTTCAGGGACCCGTCCTCTTGGACTGGAGAGCCCAGTCTGAGTCTGCCCAGAGTAAGATGCTGAGTGGAGTCGGGGGCCTCGTGCTGGGGCTGATCTTCTTGGGGGTCGGCCTCATTGTCCACAAGAGGAGCCAGAAAGGTGA

>anst_DMB_1

ATGAAGTTACTGCACCTACTGCTAGTAGGCTTCAGCCTGGGTTTTTCTGGAGCAGGGGCTTTTGTGACCCACGTGGAGAGTGACTGTGTACTGGATGAGGATGGATCAGTAAAGGACTTCACGTATTGTATCTCCTTCAACAAGAATGTGTTGACCTGCTGGGACTCAGAGATTAAAAAGATGGTCACTGTTGATTATGGGATATTGCACCCATTAGCTGAATATCTTTCTCAATCCCTTAATAACAACAGTGCCTTGATACACCGCCTGAGGAGTGGACTCCAGGATTGTGCCAGTCACACAAAACCCTTCTGGGGGTCATTGACCCAAAGAACACGGTCACCATCAGTGCAAATAGCCCAGACCACACCATTCAACACAAGGGAGCAAGTGATGTTGGCCTGCTATGTATGGGGCTTCTATCCTGCTGATGTGGCCATTTCATGGTTGAAGAATGGGCAGCCAATCCCTGACAGTGGCATTCAGAGGGCTGTACAGTCTAATGGGGACTGGACTTACCAGACACGATCCTACTTGGCCCTTACCCCCTCTAGTGGGGATATTTATACTTGCTATGTAGAGCACAGTGGGACTTCCCAGGCCATCTTTCAGACCTGGACCTCTGGCCTCTCTCTGAAGCAGACCGTGAAGATCTCTGTATCTGTGTTGACTCTGGGACTTGGCTTCATCATCTTCTTCCTTGGCCTGTTTTTCTGCCAAAAAGCCCGCTCCTCCGACTACACTCCTCTCTCGGGGTCCAATTATCCTGAAGGTAA

>Mala_DAB_1

ATGGTGGGTGTCGTACTTCCCAGGGCTTTCTGGACAAGGGTTCTGTCTCTGACCCTGCTGGTGCTGAAGTCCCAGGTGGTCTCAGGCAGACACACCCCAGAGCACTTCACGGAGCACGTGAAGCACGAGTGTCACCACGAGAACGGGACGCAGCGCGTGCGCTACATGGAGAGACACATCTACAACCGGCAGGAGTTCCTGCGCTTCGACAGCGACGTCGGGGAGTACGTGGCGGTGACGGAGCTGGGGCGGCCCATCGCGGAGTCCTGGAACAGCCAGAAGGAGTTCCTGGAGCAGAGACGGGCCGCGGTGGACACTTTCTGCAGACACAACTACGAGATATTTGAGCGCTTCTTAGTGCCCCGGAGCGGTGATTGTGCCCGAGGTGATTGTGTATCCATCCAAGCTGACTCCCCTGGGACACCACAACCTGCTGGTCTGCTCTGTCACTGGTTTCTATCCTGGGGACATTGAAGTCAGGTGGTTCCTGAATGGGCAGGAGGAGACAGCTGGGGTTGTGTCCACAGGCCTGATGAGCAACGGAGACTGGACCTTCCAGATCCTGGTGATGCTGGAAATGACCCCCAAGCGTGGAGATGTCTACACCTGCCATGTGGAGCATTCCAGCCTTGACAGTCCTGTCATCTTGGACTGGAAAGCACAGTCTGAATCTGCCCAGAGTAAGATGCTGAGTGGAGTCGGGGGCCTGGTGTTGGGGCTGATCTTCTTGGGGGTCGGCCTCATTGTCCACCAGAGGAGTCAGAAAGGTGA

>Mala_DAB_2

ATGGTGGGTATAGTATTTTCCAGGGCTTTCTGGACAAGTGTTCTGTCTCTGATCCTGCTGGTGCTGAAGTCCCAGATGGTCTTGGGCAGACACACCCCAGAGCACTTCACGGAGCACGGGAAGCACGAGTGTCACCACGAGAACGGGACGCAGCGCGTGCGCTACATGGACAGATACATCTACAACCGGCAGGAGTACGTGCGCTTCGACAGCGACGTCGGGGAGTTCGTGGCGGTGACGGAGCTGGGGCGGCCCATCGCGGAGTCCTTGAACAGCCAGAAGGAGATCCTGGAGCAGAAGCGGGCAGAGGTGGACACCGTGTGCAGACACAACTACGAGATAGCTGAGCGCTTCTTAGTGCCCCGGAGCGGTGATTGTGCCCGAGGTGATTGTGTATCCATCCAAGCTGACTCCCCTGGGACACCACAACCTGCTCGTCTGCTCTGTCACTGGTTTCTATCCTGGGGACATTGAGGTCAGGTGGTTCCTGAATGGGCAGGAGGAGACAGCTGGGGTTGTGTCCACAGGCCTGATGAGCAATGGAGACTGGACCTTCCAGATCCTGGTGATGCTGGAAATGACCCCCAAGCATGGAGATGTCTACACCTGCCATGTGGAGCATTCCAGCCTTGACAGTCCTGTCATCTTGGACTGGAAAGCACAGTCTGAATCTGCCCAGAGTAAGATGCTGACTGGAGTCGGGGGCCTGGTGTTGGGGCTGATCTTTTTGGGGGTTGGCCTCATTGTCCACCAGAGGAGTCAAAAAGGTGA

>Mala_DAB_3

ATGGGGGCTCAGCATTCCCAAGTTTCGCAACTCTTATTTCTCTTCTGTTCACTCAATAGGCTAAAGGCAAAGAGACATTGGTCAAGACAGGTGAATGTCACCTTGATTAACCCACCTTCTGGTCCTTTACAGTCTAAAGGAGAAAATGTAACCAATGACAAGGGGCATATGAATATTGTTGAAATAGTCACTGGAAAGGCAATAGATAAGGGAGAGAAGATTCCATGGGACATGAGAGAGTTATTGTTCTCTTTTCCCAAGCTTGGGAGACCTGATCCAAAGATTCCAGGGATTGTTTTAAAAGAATACAATAATTTAGATGTTAAAACATTCAGAGAATTAAAATCAGCAACTAGCACATATTGTAATACCAGTCCACATATTTGGAATATAATAGAAATATTTATTGTGCCCAAGGTGATTGTGTATCCATCCAAGCTGACTCCCCTGGGACACCACAACCTGCTCATCTGCTCTGTCACTGGTTTCTATCCTGGGGACATTGAGGTCAGGTGGTTCCTGAATGGGCAGGAGGAGACAGCTGGGGTTGTGTCCACAGGCCTGATGAGCAATGGAGACTGGACCTTCCAGATCCTGGTGATGCTGGAAATGACCCCCAAGCATGGAGATGTCTACACCTGACACGTGGAGCATTCCAGCCTTGACAGTCCTGTCATCTTGGACTGGAAAGCACAGTCTGAATCTGCCCAGAGTAAGATGCTGAATGGAGTAGGGGGCCTGGTGCTGGGGCTGATCTTCTTGGGGGTCAGCCTCATTGTCCACCAGAGGAGTCAGAAAGGTGA

>Mala_DAB_4

ATGGTGGGTATCATATTTTCCAGGGCTTTCTGGACAAGGGTTCTGTCTCTGATCCTGCTGGTACTGAAGTCCCAGATGGTCTTGGGCAGACACACCCCAGAGCACTTCACCTTGTATTGCACATCCGAGTGTCACCACGAGAACGGGACGCAGCGCGTGCGCTTCATGGACAGATACTTCTACAACCGGCAGGAGTACGTGCGCTTCGACAGCGACGTCGGGGAGTTCGTGGCGGTGACGGAGCTGGGGCGGCCCTCCGCGAAGTACTGGAACAGCCGGAAGGAGTTCCTGGAGCAGAGACGGGCCGCGGTGGACACTTTCTGCAGACACAACTACGAGATATTTGAGCGCTTCTTAGTGCCCCGGAGCGGTGATTGTGCCCGAGGTGATTGTGTATCCATCCAAGCTGACTCCCCTGGGACACCACAACCTGCTGGTCTGCTCTGTCACTGGTTTCTATCCTGGGGACATTGAGGTCAGGTGGTTCCTGAATGGGCAGGAGGAGACAGCTGGGGTTGTGTCCACAGGCCTGATGAGCAACGGAGACTGGACCCACCAGATCCTCGTAATGCTGGAAATGACCCCCAAGCATGGAGATGTCTACACCTGCCACGTGGACCACTCAAGCCTTCAGAGTCCTGTCATCTTCGACTGGAAAGCAGAGTCTGAATCTGCCCAGAGTAAGGTGTTGAGTGGAGTCGGAGGCCTGGTGTTGGGGCTGATCTTCTTGGGAGTTGGCCTTTTTGTCCACCAGAGGAGTCAGAAAGGTGA

>Mala_DAB_5

ATGTTAGATATCCTTGATAGGGTAAACCATTATTTGGCATTGGAGCTTATACCAAGAGAATACAAGATTGAAAGAATTTCTAGGACACTAAGCACTTCACGGAGCACGTGAAGCACGAGTGTCACCACGAGAACGGGACGCAGCGCGTGCGCTACATGGACAGATACTTCTACAACCGGCAGGAGTACGTGCGCTTCGACAGCGACGTTCGTGGCGGTGACGGAGCTGGGGCGGCCCGACGCGAAGTACTGGAACAGCCAGAAGGAGTTCCTGGAGCGGAAGCGGGCAGAGGTGGACACCGTGTGCAGACACAACTACGAGATATTTGAGCGCTTCTTAGTGCCCCGGAGCGTTGTGCCCGAGGTGATTGTGTATCCATCCAAGCTGACTCCCCTGGGACACCACAACCTGCTCGTCTGCTCTGTCACTGGTTTCTATCCTGGGGACATTGAGGTCAGGTGGTTCCTGAATGGGCAGGAGGAGACAGCTGGGGTTGTGTCCACAGGCCTGATGAGCAACGGAGACTGGACCCACCAGATCCTCATAATGCTGGAAATGACCCCCAAGCATGGAGATGTCTACACCTGCCACGTGGACCACTCAAGCCTTCAGAGTCCTGTCATCTTCGACTGGAAAGCAGAGTCTGAATCTGCCCAGAGTAAGGTGTTGAGTGGAGTCGGAGGCCTGGTGTTGGGGCTGATCTTCTTGGGAGTTGGCCTTTTTGTCCACCAGAGGAGTCAGAAAGGTGA

>Mala_DBB_1

ATGGTTTATGTTTGGATCTCTTTTGGCTACTGGAAGACTGGTCTGTTAATGACATCAATGGTTTTAAGTCTGCCTGTATCTTGGGCCAGGGACATTCCAGAAGATTTTGTGATCCAGCACAAGGCCGACTGTTACTTCACCAAGGGCACGGAGCGGGTGCGGCTTGTGGATCGATACATCAGCAACGACCAGGAGATTCTCCACTTCGACAGTGAGGAGGGAAAGTACGCGGCGGTCACGGAGCTGGGGCGGCCAGCTGCTGAGTATTTCAACAGTCAGCAGGACCTCCTGGAGGAACGTCGGGCCGCCGTGGACACGGTGTGCAGACACAACTACGAGGCATACAAAGCCTTCACGGTGGAGAGAAGAGTCCAGCCCAGAGTGACCATCTCCCCCTCCAAGACAGAGGCCCTGCAGCACCTGCTGGTCTGCTCTGTCACCGGCTTCTATCCAAGCAAGATCCAGGTCATCTGGTTCAAGAATGGGCAGGAGGAGACAGCTGGGAGTGTGTCCACGGGTGTGATACAGCATGGAGACTGGACCTACCAGATCCTGGTCATGTTGGAAATGGTTCCTCAGAGCAGAGATGTCTACACCTGCAGTGTGGAGCATGCCAGCCTGCAGAGCCCCATCACTGTGGAGTGGAGGTCACAGTCTGAATCTGCCCAGAGCAAATTGCTGAGTGGAGTTGGAGGCTTTGTCCTGGGACTGATCTTCCTCAGTGTAGGACTGATTATCCATTTGAAGAACCAGAAAGGTGA

>Mala_DBB_2

ATGGTTGATATTTGGCTACGCTGGATGGTAATGACATTGATGGTATTAAGTCCATCTGTATCGTGGGCCAGAGACATCCCAGAGGATTTCATGTTCCAGCATAAGGGGGAGTGCTACTTCACCAACGGCACGGAGCGGGTGCGGCTTGTGGTCAGATACATCTACAACGACCAAGATTATGTCCGCTTCGACAGCGACGTGGGGGTGTTCGCGGCGGTCACGGAGCTGGGGCGGCGGGACGCTGAGTATTACAACAGTCAGCAGGACATCCTGGAGGAACATCGAGCCTACGTGGACACGGTGTGCAGACACAACTACGAGGCAGGCAAAGCCTTCACGGTGGAGAGAAGAGCCCAGCCCAGAGTGACCATCTCCCCCTCCAAGACAGAGGCCCTGCAGCACCTGCTGGTCTGCTCTGTCACCGGCTTCTATCCAAGCAAGATCCAGGTCACCTGGTTCAAGAATGGGCAGGAGGAGACAGCTGGGATTGTGTCCACGGGTGTGATGCAGCATGGAGACTGGACCTACCAGATCCTGGTCATGTTGGAAATGGTTCCCCAGAGCAGAGATGTCTACACCTGCAGTGTGGAGCATGCCAGCCTGCAGAGCCCCATCACTGTGGAGTGGAGGCCACAGTCTGAAACTGCCCAGAGCAAATTGCTGAGTGGAGTTGGAGGCTTTGTCCTAGGGCTCATCTTCCTCAGTGTAGGACTGATCGTACACTTGAAGAACCAGAAAGGTGAGGAGCAAGAAGGAAGAGGGTTTGGTTCCATAGAACTCAGGCCAAGGGGGATGGGGGAGATCCATCTCCACAGTGGCTTTTGCTTTGACCTACCACTTACCCCATAG

>Mala_DMB_1

ATGAGGTTACTCCACCTACTACTAGTGGGCTTCAGCCTGGGTTTTTCAGGAGCAGGAGGGAACTTTGTGACCCATGTGGAGAGTGTCTGTGTGCTGGATGAAGATGGATCAGTAAAGGACTTCACATATTGTATCTCCTTCAACAAGGATATGTTGACTTGCTGGAATAATGTGATTAGCAAGATGGAAATTGTTGAATTTAGGATGCTGGAACCATTAGCTGAGTGGCTTGCTGACCACATTAATAGTGATAGTGCCTTCATCCAAAAACTGAGCAATGGATTTCAGGACTGTGCCATTCACACAAAGCCCTTTTGGGGGTCATTGACGCAAAGGACACGGCCACCATCCGTGCTAGTAGCCCAGACTGCACCATTCAACACAAGGGAGCCAGTGATGCTGGTCTGTTATGTATGGGGCTTCTATCCAGCTGACGTGGCCATTGCCTGGTTGAAGAATGGACAGCCTGTCCCGCACAGTAGTATCCAGAGGTCCGTACAGTCCAATGGTGACTGGACTTATCAGACACGATCCTACTTGGCTCTTACCCCCTCTAGTGGGGATATTTACGCTTGCCAAGTAGAGCACAGTGGGACTTCACAGCCCATCTTACAGACCTGGACACCTGGCCTCTCTATGATGCAGACAGTGAAGATCTCTGTATCTGTATTGACTTTGGGCCTTGGTCTCATCTTCTTCTTCCTTGGCTTGGTTGCCTGTCGAAAAGCTGGCTCTTCTGACTACACTATTCTCTCAGGGTCTAATTATCCTGAAGGTAA

>Phta_DNAzoo_DAB_1_partial_exon_2-4

AGCACTTCATGGAACAGAAAAAGGCCGAGTGTCACTTCGAGAACGGGACGGAGCACGTGCGGTTTATGGACAGATACTTCTACAACCGGGAGGAGTACGTGCGCTTCGACAGCGACGTGGGGGAGTACGTGGCGCTGACGGAGCTGGGGCGGCCGGATGCGGAGTATTGGAACAGCCAGAAGGAGACCCTGGAGTACAAACGCGGCCAGGTCGACAATTACTGCAGGCACAACTACGGAGTGGATGAGCCCTTCTCAGTGCGCAGGAGCGGTGACCCCAGGTGACTGTGTATCCATCCAAGATGGCTCCCCCGGGACACCACAACCTGCTTGTCTGCTCTGTCAGCGGTTTCTATCCCGGGGACATCGAGGTCCGGTGGTTCCTGAACGGGCGGGAGGAGACGGCCGGGGTGGTGTCCACGGGCCTGATGGGCAATGGAGACTGGACCTACCAGACCCTGGTGATGCTGGAGATGACCCCCCGGCGCGGAGATGTCTACACCTGCCACGTGGAACACTCCAGCCTTCAGGGACCCGTCCTCTTGTCCTGGAAAACCCAGTCTGAGTCTGCCCAGAGTAAGATGCTGAGCGGAGTTGGGGGCCTCGTGCTGGGGCTGATCTTCTTGGGGGTCGGCCTCATTGTCCACAAGAGGAGCCAGAAAGGTGA

>Phta_DNAzoo_DAB_2_partial_exon_2-3

CCCCAGGTGACTGTGTATCCATCCAAGATGGCTCCCCTGGGGCACCACAACCTGCTGGTCTGCTCCGTCAGCGGTTTCTATCCCGGGGACATCGAGGTCCGGTGGTTCCTGAACGGGCGGGAGGAGACGGCGGGGGTGGTGTCCACGGGCCTGATGGGCAATGGAGACTGGACCTACCAGACCTTGGTGATGCTGGAGATGACCCCCCGGCGCGGAGATGTCTACACCTGCCACGTGGAGCACTCCAGCCTTCAGGGACCCGTCCTCTTGTCCTGGAAAGCCCAGTCTGAGTCTGCCCAGAGTAAGATGCTGAGCGGAGTCGGGGGCCTCGTGCTGGGGCTGATCTTCTTTGGGGTCGGCCTCATTGTCCACAAGAGGAGCCAGAAAGGTGA

>Phta_DNAzoo_DAB_3

ATGGTGTGTGTCTTGCTCCCCAAGGGCATCTGGGCAGAAGTTTTGGCTGTGACCCTGTTGGTGCTGAATCCCCATGTGGTTGCAGGCAGACACAGCCCAAAGCACTTCACAAAACAATCAAAGTGCGAGTGTTACTTTATGAATGGGATGGAGCATGTGCAGTATGTGGAGAGACACATATACAACCAGAAGGAATATGTGCGCTTTGACAGCAATGTGGGGAAATATGCTGCAGTGATGGAGCTGGGCCGACCAGTGGCTGAATACTGGAACAACCATAAGGAGATTCTAGATGACTTACGGGCCCGGGTGGACACTTTGTGCAGACACAACTATCAGATTTTTGAGCCCTTCTTGTTGCCCAGGAGTGGTGATTGAGCCTGAAGTGATTGTGTATCCATCAAAGATGGCTCCCCTGGGACACCACAACCTGCTTGTCTGCTCTGTCAGCGGTTTCTACCCAGGGGACATCGAGGTCCGGTGGTTCCTGAATGGTCAGGAAGAGACGGCCAGAGTGGTGTCCACAGGCTTACTCAGCAATGGGGACTGGACCTACCAGTTACAGGTGATGCTGGAAATGATCCCCAAGAGGGGAGATGTCTACACCTGCCAAGTGGAGCACAGCAGCCTTCAGAGACCCGTCCTCTTGGACTGGAAAGCCCAGTCTGAATCTGCCCAGAGAAAGATGCTGAGTGGAGTTGGGGGCATCGTTCTGGGTTTGATCTTCTTTGGAGTTGGTCTCATTGTCCACAAGAGAAGTCAGAAAGGTGA

>Phta_DNAzoo_DAB_4

ATGGCGTGTGTCTTGTTCCCCAGGGAAGTCTGGATAGAAGTTCTGACTGTGACCCTGCTGGTGCTGAATCCCCAGGTGGCTGCAGGCAGACACAGCCCAGACAACTTCATGGAGCAGACGAAGGCGGAGTGTCACTTTGTCAATGGGATTGAGCACGTGCAGTTTGTGGGGAGACTCATATACAACCGCGAGGAGATCCTCCGCTTTGACAGCGAAGTGGGGAAGTTCGTGGCTTTGACCGAGCTGGGACGGCCCATTGAGGAGCTAATGAACAGTCAACTGGAGGCTCTGGAGCAAGCGCGGGCCCAGGTGGCCGTGTGCAGAGACAACTATATCCTGTGGGAGTCCTTTTGAGCCTGAAGTAACTGTGTATTCATCAAAGATAACTCTCCTAGGATACCCAAACCAGCTTACCTGTTTTATTACTGGTTTTTATCCTGGGGACATTAAGGTCAGATGGTTCCTGAATGGTCAAGAGGAGACAGCTGGGGTTTTGTCCACAGGCCTGATTAACAATGGAGACTGGACCTTTCAGACTCAGGTTATGCTGGAAATGATCCCCAAGCCTGGAGATGTCTACACTTGCCAAGTGGAGCACTCCAGCCTTCAGAACCCTGTCATTGGAGTTTGGGAAGCACAGTATGGATCTGCCCAGAAAAAGATGCTGAGTGGAGTTGGGGGCCTTGTACTAGGGCTGATTTTCCTTGGGGTTGGCCTTGCTGTTTATCTTAAAAGTCAAAAAGGTGA

>Phta_DNAzoo_DAB_5

ATGGAGTGTGTCTTGCTCCCCAGGGAAGTCTGGATAGAAGTTCTGGCTGTGACCCTGCTGGTGCTGAATCCCCAGGTGGCTGCAGGCAGACACAGCCCAGAGCACTTCACGTGGCAGTTTAAGTCCGAATGTTACTTCGAGAACAGGACGGAGCACGTGCGGCATGTGCACAGACTCATCTACAACGGGGAGGAGACTGCGCGCTTCGACAGCGACGTGGGGGCGTACGTGGCGCTGACGGAGCTGGGGCGGCCGGATGCTGAGTATTTCAACGGCCAGAAGGAGCTCCTGGAGAAATTCCGGGCCGAGGTGGACTCTGTGTGCAGGCGCCGCTACGAGTCCTATCACTCTTTGTCAATGCACATGCACGGTGAGTGACTGTGTATCCATCCAAGATGGCTCCCCCGGGACACCACAACCTGCTGGTCTGCTCTGTCAGCGGTTTCTATCCCAGGGACATCGAGGTCCGGTGGTTCCTGAACGGGCGGGAGGAGACGGCCAGGGTGGTGTCCACGGGCCTGATGGGCAATGGAGACTGGACCTACCAGACCCTGGTGATGCTGGAGATGACCCCCCGGCGCGGAGATGTCTACACCTGCCACGTGGAGCACTCCAGCCTTCAGGGACCCGTCCTCTTGTCCTGGACCCAGTCTGAGTCTGCCCAGAGTAAGATGCTGAGCGGAGTCGGGGGCCTCGTGCTGGGGCTGATCTTCTTGGGGGTCGGCCTCATTGTCCACAAGAGGAGCCAGAAAGGTGA

>Phta_DNAzoo_DBB_1

ATGGTTGATGTTTGGATTTCTGCTGAATGTTGGAAGATTGATCTGGTAATGACATCGATGATGTTGATACCTTTATTTTGGGGCTAAGGAAATCCCTCCCTCAAGAGGATTTCATGTACCAGTTCCAGGGTGATTGATACTTTATTAACAGCATGGAGCAAGTCTCAGACACTTCTTCAATGGCCAGGAATTTTCTTCGCTTGGACTGCTATGTGGGTAAGTTTGTGATTGATTGGGATGGAGCTGGGGTGGCCAGATACTAAATATTGGAACACTCAGAAGGAAATCATGAAGGAAGACCAAGCCTCTTTAGACAATGCCTCCACAACTATAAGGTATATATAAGCCTTTCTTATTGGAGAGAATCCAGCCCAGAGTGACCATCTCTCCTTCAAGACAAAGAGGCTTTGCAGTACCTGCTGCTCTGCTCTCTCACCAGCTTCTATCCTAAACAAGATCAAGGTCACCTGATTCAAGAATGGATTAGAGGAAACAGCTGGGATTGTGTCCATAAATCTGATACAGAATGGAGACTGGACCTACCAGACCCTGGTCCTGTTGGAAATGATTCTCCATAGCAGAGATGTCTACACATACAGTGTGGACCATGCCAGTGCACAGAGCTTCATCAGTGTGTAATGGAGGCACAATCTGAATCTACTCAGAACAAATTGATAAGTGGAATTGGAGGCTTTGTCCTGGGGCTGATCTTTTACCTGA

>Phta_DNAzoo_DMB_1

ATGAAGTTACTGCACCTACTGCTAGTAGGCTTCAGCCTGGGTTTTTCTGGAGCAGGGGCTTTTGTGACCCACGTGGAGAGTGACTGTGTACTGGATGAGGATGGATCAGTAAAGGACTTCACATATTGTATCTCCTTTAACAAGAATGTGTTGACCTGCTGGGACTCAGAGATTAAAAAGATGGTCACTGTTGATTATGGGATACTGCATCCATTAGCTGAATATCTTTCTCAATCCCTTAATAACAACAGTACCTTGATACACCGCCTGAGGAATGGACTCCAGGATTGTGCCAGTCACACAAAACCCTTCTGGGGGTCATTGACCCAAAGAACACGGTCACCATCAGTGCAAATAGCCCAGACCACACCATTCAACACAAGGGAGCAAGTGATGTTGGCCTGCTATGTATGGGGCTTCTATCCTGCTGATGTGGCCATTTCATGGTTGAAGAATGGGCAGCCAATCCCTGACAGTGGCATTCAGAGGGCTGTACAGTCTAATGGGGACTGGACTTACCAGACACGATCCTACTTGGCCCTTACCCCCTCTAGTGGGGATATTTATACTTGCTATGTAGAGCACAGTGGGGCTTCCCAGGCCATCTTACAGACCTGGACCTCTGGCCTCTCTCTGAAGCAGACTGTGAAGATCTCTGTATCTGTATTGACTCTGGGACTTGGCTTCATCTTCTTCGTCCTTGGCCTGGTTTTCTGCCAAAAAGCAGGCTCCTCTGACTACACTCCTCTCCCGGGGTCCAATTATCCTGAAGGTAA

>Trvu_DAB_1

ATGGTGTGTGTCTTGCTCCCCAGAGGCATCTGGATAAAAGTGTTGGCTGAGACCCTGCTGGTGCTGAATTCCCAGGTGACCACAGGCAGACATGCCCCAAAGCACTTCATGGAGCAGCTGAAGTCCGAGTGTTACTTTGTGAACGGGGCGGAGACCGTGCGCGATATATAGAGAGACACATCTACAACCGAGAGGAATATGTGCGCTTCGACAGCGACGTGGGGGAGTATGTGGCGTTGACGGAGCTGGGGCGGCGCAGTGCTGACTACTGGAACAGCCAGAAGGAGATCTTGGAGGACAGACGGACCCAGGTGGACACTTTCTGCAGGCTCAACTACGAGGAGATTGAGCCCTTAGTGCACAGGAGCGGTGACCCAAGGTGACTGTGTACCCATCAAAGATGGCTCCCCTGGGACACCACAACCTGCTTGTCTGCTCTGTCACTGGTTTCTATCCTGGGGACATTGAGGTCAGGTGGTTCCTGAATGGACAGGAGGAGACAGCTGGGGTTGTGTCCACAGGCCTGATCAGCAATGGAGACTGGACCTACCAGATCCTGGTGATGCTAGAAATGACCCCCAAGCGTGGAAATGTCTACACCTGCGAAGTGGAGCACTCCAGCCTTCAGAGACCTATTGTCTTGGATTGGAAAGCACAGTCTGAATATGCCCAGAGTTTAATGCTGTGTGAAATCAGGATCTTTGCGTTGGGGCTGATCTTCTTTGGGGTTGGCCTCATAGTCCACATGAGGAGTAAGAAAGGTGA

>Trvu_DAB_10

ATGCTATGTGTCTTGCTGCTCCAGGGCATCTGGACAGAGGTTCTGGCTGTGACTCTGCTGGTGCTGAATTCCCAGGTGGCTGCAGGGAGACATGCCCCAGAGCACTTCACCATGTATTCTACATCTGAGTGTTACTTTGTGAACGGGACGGAGCACGTGCAGTTTGTGGAGAGATACATCTACAACCGGCAGGAGTTTCTGCGCTTCGACAGCGCCGTGGGGGAGTATGTGGCGGTGACGGAGCTGGGGCGGCCCACTGCTGAAAATTGGAACAGAGACAAGGATCTCCTGGAGAGCAGACGGACCGCGGTGGACTGGTTCTGCAGGGTCTGGTATAAGCTGGCTGAACCCTTCTCAGTGCGCAGGCGCGGTGACCCAAGGTGACTGTGTATCCATCAAAGAGGGCTCCCCTGGGACACCACAACCTGCTTGTCTGCTCTGTCACTGGTTTCTATCCTGGGGACATTGAGGTCAGGTGGTTCCTGAATGGGCAGGAGGAGACAGCTGGGGTTGTGTCCACAGGCCTGATAAGCAATGGAGACTGGACCTACCAGATCCTGGTGATGTTGGAAATGATCCCCAAGCATGGAGATGTCTACACCTGCCAAGTGGAGCACTCCAGCCTTCAGAGACCTGTCGTCTTGGACTGGAAAGCACAGTCTGAATCTGCCCAGAGTAAGATGCTGACTGGAGTCGGGGGCCTCGTGCTGGGGCTGATCTTCTTTGGGGTTGGCCTCATTGTCCATAAGAGGAGTCAGAAAGGTGA

>Trvu_DAB_11

ATGGTGTGCGTCTTGCTCCCCGTTGGCTTCTGGATACAAGTGCTGGCTATGACTCTGTTGGTGCTGAATTCCCATGTGGCTGCAGGCAGACACACCCCAGAGCATTTCACTGAACAGGCAAAGGCCGAGTGTCACTTTGTGAACGGGGTGGAGCACGTGCGGTTTGTGGTGAGAGTCATCTACAATCGGGAGGAGATTGTGCGCTTCGACAGCGACATGGGGGAATTTGTGGCTGTGAAGGAGCTGGGGCGGAGGATTGCTGAGCATTGGAACAGCCAGAAGGAGATTCTGGAGAACACACGGGCCTCAGTGGACACATTGTGCAGGCACAATTACGAGTTATCTGAGCCAATGTTACTGCGCAGGAGCGGTGACCCGAGGTGACTGTGTATCCATCAAAGAGGGCTCCCCTGGGACACCACAACCTGCTTGTCTGCTCTGTCAACAGTTTCTATCCTGGGGACATTGAGGTCAGGTGGTTTCTGAATGGGCAGGAGGAGACAGCTGGGGTTGTGTCCACAGGCCTGATCAGCAATGGAGACTGGACCTATCAGATCCTGGTGATGCTGGAAATGACCCCCAAGCATGGAGATATCTATACCTGCCAAGTGGAGCACTCCAGCCTTCAGAGACCTGTCATCTTGGACTGGACACAGTCTCAATCTGCCCAGAATAAGATGCTGAGTGGCGTCGGGGGCCTTGTGCTGGGGCTGATCTTCTTTGGGGTTGGCCTCATTGTCTACAAGAGGAGTAAAAAAGGTGA

>Trvu_DAB_12

ATGTGGTATATCTTGTTCCTCAAGGGCATCTGGACAGAGGTTCTGGCTGTGACTCTGCTGGTGCTGAATTCCCAGGTGGCTGCAGGCAGCCGAGCCCCAGAGCACTTCACGGGACAATTTAAATATGAGTGCCACTTTGTGAACGGGACGGAGCACGTGCGGTTTGTGGAGAGACATGTCTACAACCGACAGGAGGCTGTGCGCTTCGACAGCGACGTGGGGGAGTTTGTGGCGCTGACGGAGCTGGGGAGGCGCCAGGCTGAGCTTTGGAACAGCCAGAAGGATTACATGGAGGGTAGACGGGCCCAGGTGGACACTTGTAGGCACAACTACCAGCTCGTGCCCTTCTAACTGCGCAGGCCCTGTGACCTGAGGTGACTGTGTATCTATCAAAGATGGCTCCCCTGGGACACCACAACCTGCTTGTCTGCTCTGTCACTGGTTTCTATCCTGGGGACATTGAGGTCCAGTGGTTCTTGAACGGGCAGGAGGATACAGCAGGGGTTGTGTCCACAGGCCTGATCAGCAATGGAGACTGGACCTACCAGATCCTGGTGACGCTGGAAATGACCCCCAAGCATGGAGACGTCTACACCTGCCAAGTGGAGCACTCCAGCTTTCAGAGACCTGTCATCTTGGAGTGGAAAGCACATTCTGAATCTGCCCAGAGCAAGATGCTGAGTGGAGTCGGGGGCCTTGTGTTGGGGCTTATCTTCTTTGGGGTTGGCCTCATTGTCCACAAGAGGAGTCAGAAAGGTGA

>Trvu_DAB_2

ATGGTGTGTGTCTTGCTCCTCCAGAGAATCTGGACAGAGGTTCTGGTTGTGACCCTGCTGGTGCTAAATTCCCAGGTGGCTGAAGGCACTCATGTGCCAGAGCACTTCACGGAGCAGCTGAAGTCCGAGTGTCACTTTGTGAACGGGACCGAACACGTGCGGTTTGTGGCGAGATTCATCTACAACCAGGAGGAGTATGTGCGCTTCGACAGCGACGTGGGAGAGTTTGTGGCAGTGACAGAGCTGGGGCGGCGCGATGCCGAGAATTGGAACAACAGGAAGGATGCCTTGGGCCACTTAAGGGCTCAGGTGCGCGCTGCGTGCAAGTACGACTACGAGGTGATTGCGCCCTTCTTAGTGCGCATGCGCGGTGACCCAAGGTGATTGTGTATCCATCAAAGACAGCTCCCCTGGGACACCACAACCTGCTTGTCTGCTCTGTCACTAGTTTCTATCCTGGGGACATTGAGGTCAAGTGGTTCCTGAATGGGCAGGAGGAGACAGCTGGGGTTGTGTCCACAGGCCTGATCAGCAATGGAGACTGGACCTACCAGATCCTGGTGATGCTGGAAATGATCCCCAAGCATGGAGATGTCTACACTTGCCAAGTGGAGCACTCCAGCTTTCAGAGACCTGCCATCTTTGAATGGAAAGCACAGTCTGAATCTGCCCAGAGTAAGATTCTGAGTGGAGTCGGGGGCCTCGTGCTGGGTCTGATCTTCTTTGGGGTTGGCCTCATTGTCCACAAGAGGAGTCTGAAAGGTGA

>Trvu_DAB_3

ATGCTGTGTGTCTTGCTCCCCAAGGGCATCTGGATAGAGGTTCTGGCTGTGACCCTGCTGGTGCTGATTTCCCAGGTGGCTGCAGGCAGACATTCCCCAGGAGTCCTTCATCCAACAGTTTAAACATGAGTGTCACTTTGGGAACGGGACGGAGCACATGCGTCATGTGGATAGATTCATCTACAACGGGGAGGAGTATGTGCGCTACGACAGCGACCTCGGGGAGTATGTGGCGGTGACAGAGCTGGGGCGGCCTGAGGCTGAGTACTGGAACAGCCAGAAGGATCTCATGGAGCGCAAACGGGCAGAGATTGACACCGTGTGCAGGCCCACTTACGAGTCATCTGAGCGTTTCTTACTGCGCGGTGATTAAGCCCGAGGTGACTGTGTATCCATCAAAGATGGCTCCCCTGGGACAACACAACCTGCTTGTCTGCTCTGTCACTGGTTTCTACCCTGGGGACATTGAGGTCCAGTGGTTCGTGAACGGGCAGGAGGAGACAGCTGGGGTTGTGTCCACAGGCCTGATCAGCAATGGAGATTGGACCTACCAGATCCTGGTGATGCTGGAAATGACCCCCAAGCATGGAGATGTCTACACCTGCCAAGTGGAGCACTCCAGCCTTCAGAGACCTGCCATCTTACACTGGAAAGTACAGTCTGAATCTGCCCAGAGTAAGATGCTGAGTGGAGTCGGGGGCCTCGTGCTGGGGCTGATCTTCTTTGGGGTTGGCCTCATTGTCCACAAAAGGAATCAGAAAGGTGA

>Trvu_DAB_4

ATGCTGTGTGTCTTGCTCCACAAGGGCGTCTGGACAGAGGTTCTGGCTGTGACCCTGCTGGTGCTCACTTCCCAGGTGGCTGCAGGCAGACATGCCCCAGAGCACTACACTGAGCAGGCTAAGGGCGAATGTCACTTCATGAACGGGACGGAGCGCGTGCGGCATGTGATGAGATACGTCTATAACCGGGAGGAGTTTCTGCGCTACGACAGCGACGTGGGGGAGTTTGTGGCGGTGACGGAGCTGGGGCGGCGCAGTGCTGAGTATTATAACAGCCAGAAGGAGATCCTGGAGCGCGCACGGGCAATGGTGGACAATTGCAGGCACAACTACCAGGTGGTGCCCTTCTTATTGCGCAGGCCCTGTGACCCGAGGTGACTGTGTATCCATCAAAGACGGCTCCCCTGGGACACCACAACCTGCTTGTCTGCTCTGTCACTGGTTTCTATCCTGGGGACATTGAGGTCAGGTGGTTCCTGAATGGGCAGGAGGAGACAGCTGGGGTTGTGTCCACAGGTCTGATCAGTAATGGAGACTGGACCTACCAGATCCTGGTGATGCTGGAAGTGATCCCCAAGCGTGGAGATGTGTACACCTGCCAAGTGGAGCACTCTAGCCTTCAGAGACCTGTCGTCTTGGAGTGGAGAGCACAGTCTGAATCTGCCCAGAGTAAGATGCTGAGTGGAGTCGGGGGCCTCGTGCTGGGGCTGATCTTCTTTGGGGTTGGCCTCATTGTCCACAGGAGGAGTCAGAAAGGTGA

>Trvu_DAB_5

ATGCTGTGTGTCTTGCTGCCCAAGGGCTTCTGGACAGAGGTTCTGGCTGTGACCCTACTGGTGCTGACTTCCCAGGTGGCTGCAGGAAGACATGCTCCAGAGCACTTCACGGAGCAGCTTAAGGCCGAGTGTCACTTTGTGAACGGGACGGAGAGCGTGCGGTATGTGCTGAGATTCATCTACAACCGCGAGGAGTTTGTGCGCTTCGACAACGCCGTGGGGGAGTTTGTGGCGGTGACGGAGCTGGGGCGGCGCAGTGCTGAGTATTTGAACAGAGACAAGGATTACCTGGAGGGCAGACGGACCGCGGTGGACTGGTTATGCAGGCACAACTACCAGGTGGTGCCCTTCTTACTGCGCGGGCCCTGTGACCCGAGGTGATTGTGTATCCATCAAAGACAGCTCCCCTGGGACACCACAACCTGCTTGTCTGCTCTGTCACTGGTTTCTATCCTGGGGACATTGAGGTCAGGTGGTTCGTGAATGGGCAGGAGGAGACAGCTGGGGTTGTGTCCACAGGCCTGATCAGCAATGGAGACTGGACCTACCAGATCCTGGTGATGCTGGAAATGACCCCCAAGCGTGGAGATGTCTACACCTGCCAAGTGGAGCACTCCAGCCTTCAGAGACCTGTCATCCTAGACTGGAGAGCACAGTCTGAATCTGCCCAGAGTAAGATGCTGAGTGGAGTCGGGGGCCTCGTGCTGGGGCTGATCTTCTTTGGGGTTGGTCTCATTGTCCACAGGAGGAGTCAGAAAGGTGA

>Trvu_DAB_6

ATGCTGTGTGTCTTGCTCCCCAAGGATAGAGTCTGGATAGAGGTTCTGGCTGTGACCCTGCTGGTGCTGACTTCCCAGGTGGCTGCAGGCAGACGTGCCCCAGAGCACTTCACGGAGCAGGGGAAGTCCGAGTGTCACTTTGTGAACGGGACGGAGCGCCTGCGGTTTGTGGATAGATACATCTACAACCGGGAGGAATATGTGCGCTTCGACAGCGACGTGGGGGAGTTTGTGGCGGTGACGGAGCTGGGGCGGCGCAGTGCTGAGTATTATAACAGCCTGAAGGATTACCTGGAGGGTAGACGTGGCCAGGTGGACACTTACTGCAGGCACAACTACGAGGTCATCGAGCCCTTCTCAGTGCGCAGGCGCGCCCGAGGTGACTGTGTATCCATCAAAGACGGCTCCCCTGGGACACCACAACCTGCTTGTCTGCTCTGTCACTGGTTTCTATCCTGGGGACATTGAGGTCCGGTGGTTCCTGAACGGGCAGGAGGAGACAGCTGGGGTTGTGTCCACAGGCCTGATCAGCAATGGAGAGTGGACCTACCAGATCCTGGTGATGCTGGAAATGACCCCCAAGCGTGGAGACGTCTACACCTGCCAAGTGGAGCACTCCAGCCTTCAGAGACCTGTCATCTTAGACTGGAGAGCACAGTCTGAATCTGCCCAGAGTAAGATGCTGAGTGGAGTCGGGGGCCTCGTGCTGGGGCTGATCTTCTTTGGGGTTGGCCTCATTGTCCACAGGAGGAGTCAGAAAGGTGA

>Trvu_DAB_7

ATGGTGTGTGTCTTGCTTCTCAGGGGTGTCTGGATAGAAGTTCTGGCTTTGACTCTGCTGGTGCTGAATTCCCAAGTGGCTGCAGGCAGACATGCCCCAGAGCATTTCACGGAGCAGGTGAAGGGCGAATGTTCCTTTGTGAACCGGACGGAGCACACGCGGTTTGTGCTGAGGGCCATCTACAACCGACAGGAGTACGCGCGCTTCGACAGCGAGGTGGGGGTGTTTGTGGCTACTACAGAGCTCGGGCGGCTCACTGCTCAGTTTGCTAACAGCCACAAGGAGTTCATGGATCACTTACGGGCTGCGGTGGACACTTATTGCAGGCACAACTACGAGGTGTTTGAGCCCGCTTCAGTGCCCAGGAGCGCCTGAGGTGACTGTGTATCCATCAAAGATGGCTCCCCTGGGACACCACAACCTACTTGTCTGCTCTGTCACTGGTTTCTATCCTGGGGAAATTGAAGTCCAATGGTTCCTGAATGGACAGGAGGAGACAGAGGGGGTTGTGTCCACAGGCCTGATCAGCAATGGAGACTGGACCTACCAGATCCTGGTGATGCTGGAAATGACCCCCAAGCGTGGAGATGTCTACACCTGCCAAGTGGAGCACTCCAGCCTTCAGAGACCTGTCATCTTTGACTGGAAAGCACAGTCTCAATCTGCCCAGAATAAGATGCTGAGTGGAATCGGGGTCCTTGTGCTAGGGCTGATCTTCTTTGTGGTTGGCCTCATTGTGCACAAGAGGAATCAGAAAGGTGA

>Trvu_DAB_8

ATGGCATATGTCTTGCTCCCCAAGAGCATCTGGACAGAAGTTCTGGTTGTGACCCTATTGGTGCTGAATTCCCAGGTGGCTGCAGGCAGACATGCCCCAGAGCACTTCATGGAGCAGAGAAAGGCAGAGTGTTACTTTGTGAATGGGACAGAGCATGTGAGGTATGTGCTGAGATGCATCCACAATCGGGAGGAGATTTTGCGCTTCGACAGTGACGTGGGGGTGTTTGTGGCGATAACGGAGCTGGGGCGGCCCGAGGCTGAATACTGGAACACCCAAAAGGAGATACTGAAATACAGACGCGACCAAGTGAACACTTACTGCAGGCACAACTACCAGTGGACTGAGGCCTTCTCAAAGAGCTGGAGCCCTGAGCCCCAGGTGATTGTGTATCCATCAAAGATGGCTCCCCTGGGACACCACAACCTGCTTGTCTGCTCTGTCACTGGTTTCTATCCTGGGGACATTGAGGTCAAGTGGTTCCTGAATGGTCAGGAGCAGACAACTGGGGTTGTGTCTACAGGCCTGTTAAGCAATGGAGACTGGACCTACCAGATCCTGGTGATGCTGGAAATGACCCCCAAGCATGGAGATGTCTACACCTGCCAAGTGGAGCACTCTAGCCTTCACAGACCTGTCGTCTTTGACTGGAAAGCACAGTCTGAATATGCCCAGATTAAGATGTTGAGTGGAGCTGGTGGCCTTGTGCTGGGTCTGATCTTCTTTGTGGTTGGCCTCATTGTCCACACGAGGAGCCAAAAAGGTGA

>Trvu_DAB_9

ATGCTGTGTGTCTTGCTCCCCAAGGGCGTCTGGACAGAGGTTCTGGCTGTGACCCTGCTGGTGCTGATTTCTCAGGTGGCTGCAGGCAGACATGCCCCAGAGCACTACACTGAGCAGGCTAAGGGCGAATGTCACTTCGTGAACGGGACGGAGCGCGTGCGGCATGTGATGAGATACGTCTATAACCGGGAGGAGTTTCTGCGCTACGACAGCGACGTGGGGGAGTTTGTGGCGGTGACGGAGCTGGGGCGGCGCGATGCTGAGAAATGGAACAGCCAGAAGGAGATCCTGGAGCGCGCACGGGCAATGGTGGACAATTGCAGGCACAACTACCAGTTGGTGCCCTTCTTATTGCGCAGGCCCTGTGACCCGAGGTGACTGTGTATCCATCAAAGACGGCTCCCCTGGGACACCACAACCTGCTTGTCTGCTCTGTCACTGGTTTCTATCCTGGGGACATTGAGGTCCGGTGGTTCCTGAATGGGCAGGAGGAGACAGCTGGGGTTGTGTCCACAGGCCTGATCAGCAATGGAGACTGGACCTACCAGATCCTGGTGATGCTAGAAATGACCCCCAAGCGTGGAGATGTCTACACCTGCCAAGCGGAGCACTCCAGTCTTCAGAGACCTGTCGTCTTGGAGTGGAGAGCACAGTCTGAATCTGCCCAGAGTAAGATGCTGAGTGGAGTCGGGGGCCTCGTGCTGGGGCTGATCTTCTTTGGGGTTGGCCTCATTGTCCACAGGAGGAGTCAGAAAGGTGA

>Trvu_DBB_1

ATGGTTAACGTTTGGATCTCTGCTGACTGTTGGAAGATTGGTCTGTTAATAACATTGATGGTGTTGAGTATACCTGCATCTTGGGCCAGGGACATCCCAGGGGATTTCGTGTTTCAGCACAAGGGTGACTGTTACTTCACCAACGGCTCGGAGCGGGTCCGGCTTGTGGCTCGATACATCTACAATGACCAGGAATATGCCCGCTTCGACAGCGCCGTGGGGGAGTTCGAGGCCGTGTCCGAGCTGGGGCGGCGTCATGCTGAGGATTTTAACAGTCAGAAGGAGCTCCTGGAGCAACATCGAGCCTACGTGGACACGGTGTGCAGACACAACTACGAGGTACACAAGCCCTTCGCTATGGACAGAAGAGTCCAGCCCAGAGTGACCATCTCCCCCTCCAGGACGGAGGCCCTGCAGCACCTGCTGGTCTGCTCTGTCTCTGGCTTCTATCCAAGCAAGGTCAAGGTCACCTGGCTCAAGAATGGGCAGGAGGAGACGGCTGGGGTTGTGTCCACGGGGGTGATACAGAACGGAGACTGGACCTACCAGACCCTGGTCATGTTGGAAATGACTCCCCAGAGCAGAGATGTCTACACCTGCAGCGTGGAGCATGCCAGCCTACAGAGCCCCATCAGGGTGGAATGGAGGGCACAGTCTGAATCTGCCCGGAGCAAACTGCTGAGTGGAATTGGAGGCTTTGTCCTGGGGCTGATCTTCCTTGGGGTAGGACTGATCATCCACCTGAAGAACCAGAAAGGTGA

>Trvu_DBB_2

ATGGTTGATGTTTGGATCTCTGCTGGTTACTGGAAGATTGGTCTGTTGATGACATTGACGGTGTTGAGTCTATCTGCATCTTGGGCCAGGGACATCCCAGAGGATTTCATGCGTCAGCACAAGGCTGAGTGTTACTTCACCAACGGCACGGAGCGGGTCCGGTTTGTGGAAAGATACATCTACAATGACCAGGAGGATGTCCGCTTCGACAGCGAAGTGGGGGAGTACGTGGCCGTGACGGAGCTGGGGCGGCCCGATGCTGAGTACTGGAACAGTCAGAAGGAGCTCCTGGAGGAAAAACGAGCCGAAGTGGACACACTGTGCAGACACAACTACGAGGCAGGCAAGTCCTTCACGGTGGACAGAAGAGTCCAGCCCAGAGTGACCATCTCCCCCTCCAGGACGGAGGCCCTGCAGCACCTGCTGGTCTGCTCTGTCTCTGGCTTCTATCCAAGCAAGGTCAAGGTCACCTGGCTCAAGAATGGGCAGGAGGAGACGGCTGGGGTTGTGTCCACGGGTGTGATGCAACATGGAGACTGGACCTACCAGACCCTGGTGATGTTGGAAATGATGCCCCAGAGCAGAGATGTCTACACTTGCAGCGTGGAGCATGCCAGCCTACAGAGCCCCATCAGGGTGGAATGGAGGGCACAGTCTGAATCTGCCCGGAGCAAACTGCTGAGTGGCATTGGAGGCTTTGTCCTGGGGCTGATCTTCCTTGGGGTAGGACTGATCATCCACCTGAAGAACCAGAAAGGTGA

>Trvu_DCB_1

ATGGTGTGTGTGGAGTTTCTGGGAAGCCCCTGTAGGACAGTCCTATTGATAGAGCTGAGCACGCCCACAGCCTGGGGCAGGGAAATTCCAGAGAATTACCTACATCAGGTGAAGTCCGAGTGTCACATGGCCAATGGAACCCAACGGGTGCACATCGTGGGAAGACTCGTCTACATCCAAGTGGAGTTTGTGTGCTTTGACAGTGATGTGAGACTATTTGAGGCAAGAATGGAGCTGTGGAAATCCCAAGTCCAGAAATGGAACAGTCAGAAGGAGATAGTGAAGCATGCAAGGTCCATAGTGAATTTGTGCAGACAAAACTATCTTTTATATGATAAATTCATAGTGCAAAGGAAAGTCCAGCCCCAAGTGAAGGTTTTCCCCTCAAAGATACAACCAGTTGGGCACCACAACCTGCTCCTCTGTTCTGTGACCAGTTTCTGTCCTGGTGAGATCAAGGTCAGCTGGTTCAGGAACACAAAAGAAGAGAAAGCTGGAGTTCTGTCCACAGGCTGAATCCAGAATGGTGACTGGACCTTCCAAACCCTTGTGATGTTGGAAATGACTCCCCTCCCCAAAGAGGAGATGTCTTTACTTGCCATGTAGACCATGTCAGCTTGCAGAGCCTTGTCACTGTAGACTGAAGAGCGCTGGCTGAATCTGCCCAGACTCAGATGCTGACTGGGCTTGGTGCTTGGACTGCTCTTACTTGGAGTGGGACTTTTCATCCTCCTCAGAAACTTGAAAGATTCCTACTCTGTGACCAAAGAAGATTCAGATTTGGAAGGGATTGTGAACATTGCCCCATTGCAACAAGATTTTCCCAGAGCTGTTACCCAGTCCTAG

>Trvu_DMB_1

ATGAGGTTACTACACCTGCTACTGGTGGGCTTCAGCCTGGGTTTTCCAGGAGCAGGGGGCTTTGTGACCCATGTGGAGAGTGGCTGTATGCTGGATGAAGAAGGATCAGCAAAGGACTTCACATATTGTATCTCCTTCAACAAGGATGTGTTGACCTGCTGGGACTCGGAGACTAACAAGATGGTCACTGTCGATTTTGGAATACTGCATCAATTGGCTGAGGAGATTTCTGTTGCCCTTAATAACGATAGTGCTCTGATGGACCGCCTGAGCAAAGGATTCCAGGACTGTGCCAGTCACACAAAGCCCTTCTGGGGCTCGTTGACCCAAAGGACGCGGCCACCATCAGTGCAAATAGCTCAGACCACACCATTTAACACAAGGGAGCCAGTGATGCTGGCCTGTTATGTATGGGGCTTCTATCCTGCTGATGTGGCTGTTTCATGGCTGAAGAATGGGCAGCCTATTCCAGACAGTGGCATCCAGAGGGCTGTACAATCCAATGGGGACTGGACTTATCAGACACGATCCTACTTGGCCCTTACTCCCTCTAGTGGGGATATTTATACTTGCCATGTAGAGCACAGTGGGACTTCCCAGAGCATCTTACAGACCTGGACACCTGGCCTCTCTCTGAAGCAGACAGTGAAGATCTCTGTATCTGTGCTGACTCTATGCCTTGGTCTCGTCGTCTTCTTCCTTGGCCTGGTTGCCTGCCGAAAAGCTGGCTCCTCTGACTACACTCCTCTCTCGGGGTCCAATTATCCTGAAGGTAA

>Pscu_DNAzoo_DAB_1

ATGGTGTGTGTGTTGCTCCTCAGAGGCATCTGGACAGAGGTTCTGGCTGTGACCCTGTTGGTGCTGACTTTCCAAGTGACTGCAGGCAGACATGCCCCAGAGCACTTCACCCAGCAGGCCAAGTGCGAGTGTTACTTTGAGAACGGGACGCAGCACGTGCGGTTTATGGTGAGACACATCACCAACGGGGTGGAGAATGTGCGCTTCGACAGCGACGTGGGGGAGTTTGTGGCGCTGACGGAGCTGGGGCGGCGCGATGCTGAGCTTTGGAACAGCCAGAAGGATTACCTGGAGGACGCACGGGCCGCAGTGGACACTTTGTGCAGATACAACCACAAGTTGTCTGAGCCCTTAGTGCGCAGGCGCGGTGATTGAGCCCAAGGTGATCGTGTATCCATCAAAGATGGCTCCCCTGGGACACCACAACCTGCTTGTCTGCTCTGTCACTGGTTTCCTGGGGACATTGAGGTCAGGTGGTTCCTGAATGGGCAGGAGGAGACAGCTAGGGTTGTGTCCACAGGCCTGATCAGCAATGGAGACTGGACCTACCAGATCCTGGTGATGCTGGAAATGACCCCCAAGCTTGGAGATGTCTACACCTGCCAAGTGGAGCACTCCAGCCTTCAAAGACCTGTCGTCTTGGACTGGAAAGCACAGTCTGAATCTGCCCAGAGTAAGATGCTGAGTGGAGTTGGGGGCCTGGTGCTGGGTCTGATCTTCTTTGGGGTTGGCCTCATTGTCTACAAGTGGAGGCAGAAAGGTGA

>Pscu_DNAzoo_DAB_2

ATGATGTGTATCTTGCTCCTCAGGGGTGTCTGGATAGAGGCTCTGGTTGTGACACTGCTGGTGCTGAATTCCCAGGTGGCTGCAGGCAGACATGCTCAAGAGCACTTCACGGAGCAGGTGAAGGGCGAATGTTACTTTGAGAACAGGACGGAGCACGTGCGGTTTGTGCTGAGGGCCATCTACAACCGGGAGGAGTACGCGCGCTTCGACAGCGACGTGGGGGAGTTTGTGGCGGTGACGGAGCTGGGGAGGCTCACTGCGGAGTATGGGAACTCCCAGAAGGAGTTTATGGACCACTTACGGACTGCGGTGGACAGTTATTGCACGTACAACTACGAGGGAATTGAGCCCTTCTCAGTACCCAGGAGGACTCAACCAGAGGTGATTGTGTATCCATCAAAGATGGCTCCCTTGGGACACCACAACTTGCTTGTCTGCTCTGTCAGTGGTTTCTATCCTGGGGACATTGAGGTCAGGTGGTTCCTGAATGGGCAGGAGGAGACAGCTGGGGTTGTGTCCACAGGCCTGATCAGCAATGGAGACTGGACCTACCAGATCCTGGTGATGCTAGAAATGACCCCCACGCGTGGAGATGTCTACACCTGCCAAGTGGAGCACTCCAGTCTTCAGAAACCTGTCATCTTTAACTGGAAAGCACAGTCTGGATCTGCCCAGAATAAGATGCTGAGTGGAGTCGGGGTCCTTGTGCTGGGTCTGATCTTCTTTGGAATTGGCCTCATTGTCCACAAGAGGAATCAGAAAGGTGA

>Pscu_DNAzoo_DAB_3

ATGCTGTGTGTGTGGACAAAAGTTCTGATGATGACCCTGCTGGTGCTGAATTCCCTGGTGGTTGCAGGCAGAGACAGCCCAAAGCACTTTATGGAGCAGATGAAGGCCGAGTGTCACTTTGTCAATGGGACTGAGCATGTGCGATTTGTGGGGAGACTCATCTACAACAGCCAGGAGATTCTGCGTTTCGACAGCAACTTGGGGAAATTTGTGGCCTTGACCGAGCTGGGGCGGCCCATTGCAGAGCTAATGAACAGCCTGCTGGAGGCCCTGGAGCAAGCGCGGGCCCAGGTGGCCTGGTGCAGAGACAACTATAGGTTGTTGAAGTCCTGGATGCAGAGGAGGGGTGAGTGAGCCTGAAGTAACTGTGTATCCATCAAAGATGGCTCCCCTAGGATACCCAAACCAGCTTGTCTGTTTTGTGACTGGTTTCTATCCTGGGGACATTGAGGTCAAGTGGTTCTTGAATGATCAGGAGGAGACAGCTGGGGTTGTGTCCACAGGCCTGATTAGCAATGGAGACTGGACCTACCAGATCCTGGTGATGCTGGAAATGACCCCTAAGCGTGGAGATATCTACACCTGCCAAGTAGAGCATTCCAGCCTTCAGAATCCTGTCATTGTGGTCTGGGAAGCACAGGCCACGTCTGCCCAAGGAAAGATGCTAAGTGGAATTGGAAGCCTTGTGCTAGGGCTAATCTTCCTGGGGATTGGCCTTGCTGTTCACCTTAGGAGTCAGAGAGGTGA

>Pscu_DNAzoo_DCB_1_partial_exon_2-5

AGAATTACTTACATCAGGTGAGGTCTGAGTATTCCATGACCAATGGAACCCAACAAGTGCACTTCGTGGGAAGACTCATCTACAACTGGGTAGAGTTCGTGTGCTTTGACAGTGACGTGGGGCTATTTGAGGTAAGAATGGAGCTGTGGAAATCCCAAGTCCAGAAATGAAACAGTCAGAAGGAGATAGTTGAGCATGCAAGGTCCATAGTGAATGTGTGCAGACACAACTATCCTTTATATGATACATAGTGCAAATAAAAGCCCTGAGTGAAGGTTTTCCCCTCAAAGGTATAACCACTTGGGCACCACAATCTGCTCCTCTGTTCCGTGACCAGTTTCTATCCTGGTGAGATCAAGATTAGCTGGTTCAGGAATGCGAAAGAAAAGACTGGGGTCATGTCCACAGGCCGAATCCAGAATGGTGACTGGACCTTCCATTCCTTGGGATGCTGGAAATGACCCCCCAAAGAGGAGATATCTTTACTTGTCATGTGGACCATGTCAGCTTGCAGAGCCCTGTCACCTTAGACTAGAAGCACAGTCTGAATCTGCCCGGACTAAGATGCTGACTGGAATTGGGGACCTGGTGCTTGGACTGTTTTTACTTGGAGTGGAACTTGTCATCCACCTCAGATATTTGAAAGATTCCTGTTCTAGGACCAAAGAAGATTAAAATTTGGAAAGGATTGTGAACTTTGCAAGAAGATTTTCCCAGAGCTGTTGCCCAATCCTAG

>Pscu_DNAzoo_DMB_1

ATGAGGTTACTCCACCTGTTACTAGTGGGCTTCAGTCTGAGTTTTTCAGGAGCAGGGGGCTTTGTGACCCATGTGGAGAGTGGCTGTATGCTGGATGAAGAAGGATCAGTAAAGGACTTCACATATTGTATCTCCTTCAACAAGGTTGTGTTGACCTGCTGGAACTCAGAGACTAACAAGATGACCACTGTTGATTTTGGAATTCTATATCCATTAGCTGAGCAGCTTTCTGGAGCCCTCAGTAATGATAGTGCTTTTATAGACCACCTGAGCAAAGGACTACAGGACTGTGCTAGTCACACGAAGCCCTTCTGGGGATCACTGACCCAAAGGACACGGCCACCATCAGTGCAAGTAGCCCAGAGTACACCATTTAACACAAGGGAGCCAGTGATGCTGGCCTGTTATGTATGGGGTTTCTATCCTGCTGATGTGGCCATTTCATGGTTGAAGAATGGGCAGCCTATCCCACACAGTGGCATCCAGAAGGCTGTACAATCCAATGGAGACTGGACTTACCAGACACGATCCTACTTGGCCCTTACCCCCTCTAGTGGGGATATTTACTCTTGTCATGTAGAACACAGTGGGACTTCCCAGATCATCTCACAGACCTGGACACCTGGCCTCTCTCTGAAGCAGACAATGAAGATCTCTGTATCTGTACTGACTCTAAGCCTTGGCCTCATCTTCTTCCTCGTTGGCCTGGTTGCCTGCCGAAAAGCTGGCTCCTCTGATTACACTCCTCTCTCGGGGTCCAATTATCCTGAAGGTAA

>Smcr_DNAzoo_DAB_1

ATGGTGTGTGTCTTGTTCCCCAAGGGCATCTGGATAGAAGTTCTGGCTGTGACCCTGCTGTTGCTGAATCCCCAAGTAGTTACAGGCAGAAACACACCAAAGCACTTCACAAAACAGTCAAAGTGTGAGTGTTACTTTGTAAATGGGATGGAGCATGTGCAGTATGTGGAGAGACACGTGTACAACCAGAAGGAATATGTGCGCTTTGACAGCAACGTGGGGAAGTATGCTGCAGTGATGGAGCTGGGCTGACCAGAGACTGAATACTGGAACAACCGTAAGGAGATTCTAGATGACTTACGGGCCCGGGTGGACACTTTGTGCAGACACAACTACCAGGTTATTGAGCCCTTCTTGTTGCCCAGGAGTGGTGATTGAACCTGAGGTGATTGTGTATCCATCAAAGATGGCTCCTCTGAGATATCATAACCTGCTTGTCTGCTCTGTCAGCGGTTTCTACCCTGGGAACATCGAGGTCCGGTGGTTCCTGAATGGGCAGGAAGAGACAGCTGGGGTGGTGTCCACAGACTTAATCAACAATGGGGACTGGACTTACCAGTTACAGGTGATGCTGGAAATGATCCCCAAGAGTGGAGATGTCTACACCTGCCAAGTGGAGCACTCCAGCCTTCAGAGACCCATCCTCTTGGACTGGAAAGCTCAGACTGAATCTGCCCAGAGAAAGATGCTGAGTGGAGTTGGGGGCATCGTTCTGGGTGTGATCTTCTTTGGAGTTGGTCTCATTGTCCACCAGAGAAGTCGGAAAGGTGA

>Smcr_DNAzoo_DAB_2

ATGATTGTGTGTGTCTTTCTCCACAAGGAAGTCTGGATAGAAGTTCTGGCTGTGACCCTGCTGGTGCTGAATTCCCAGATGGCTGCAAACAGACACAGCCCAGAGCACTTCACGGAGCAGACAAAATCCGAGTGTTACTTCGAGAACGGGACGGAGCACGTGCGGTTTGTGGAAAGACACATCTCCAACGGGGTGGAGTACGTGCGCTTCGACAGCGACGTGGGGAAGTACGTGGCTCTGACGGAGCTGGGGCGGGGCAGTGCGGAGCACTGGAACAGCCAGAAGGAGATCATGAAGTACAGACGGGCCGCGGTGGACACTTACTGCAGGCCTAACTACGAAGGGTCTGAGCCCTTCTTAGTGCCCAGGAGCGGTGACCCCAGGTGACTGTGTATCCATCCAAAATGGCTCCCTTGGGACACCACAACCTGCTTGTGTGCTCTGTGGGCGGCTTCTATCCTGGGGACATAGAGGTCCGGTGGTTCCTGAATGGGCGGGAGGAGACGGCCGGCGTGGTGTCCACAGGCCTGGTGGGCAATGGAGACTGGACCTACCAGACCCTGGTGATGCTGGAGATGACGCCCCGGCGGGGAGACGTCTACACCTGCCACGTGGAGCACTCCAGCCTTCAGAGACCCTTCCTCTTGGACTGGAAAGCCCAGTCAGAGTCTGCCCAGAATAAGATGCTGAGTGGAGTTGGGGGCCTTGTGCTGGGGCTGATCTTCTTTGGGGTTGGCCTCATTGTCCACAAGAAGAGCCAGAAAGGTGA

>Smcr_DNAzoo_DAB_3

ATGGTGTGTGTCTTGCTCCCCAAGGAAGTCTGGATACAAGTTCTGGCTGTGACCCTGCTGGTGCTGAATCCCCAGGTGGCTGCAGGCAGACACAGCCCAGAGCACTTCACCAAGGAGTTTAAGTACGAGTGTTACTTTGAGAACGGGATAGAGCAGGTGCGGCTTGTGGTGAGACACATCTACAACCGGGAGGAGTTTGTCCGCTATGACAGCGACGTGGGGAAGTACGTGGAGGTGACGGAGCTGGGGCGGGGCATTGCCGAGTACTTCAACAGCCAGAAGGAGAAACTGGAGCAGAGACAGGCCGAAGTGGACACTGTGTGCAGGCACAACTACGGTGTATCTGAGCTCTTCTTAGTGGGCAGGCGCGGTGATCCAGCCCCAGGTGACTGTGTATCCATCCAAGATGGCCGCCCCGGGACAGCACAACCTGCTGGTGTGCTCCGTGGGCGGCTTCTATCCCGGGGACGTCGAGGTCCGCTGGTTCCTGAATGGGCGGGAGGAGACGGCCGGCGTGGTGTCCACGGGCCTGGTGGGCAATGGAGACTGGACCTTCCAGACCCTGGTGATGCTGGAGATGACGCCCCGGCGGGGAGACATCTACACCTGCTTCGTGGAGCACTCCAGCCTTGAGGGAGCCGTCCTCGTGGACTGGAGAGCCCAGTTTGAGTCTGCCCAGAGTAAGATGCTGAGCGGAGTCGGGGGCCTCGTGCTGGGGCTGATCTTCCTGGGGGTCGGCCTCATTGTCCACAAGAGGAGCCAGAAAGGTGA

>Smcr_DNAzoo_DAB_4

ATGGTGTGTGTCTTGCTCCCCAAGGAAGTCTGGATACAAGTTCTGGCTGTGACCCTGCTGGTGCTGAATCCCCAGGTGGCTGCAGGCAGACACAGCCCAGAGCACTTCACGGAGCAGGTAAAGCACGAATGTCACTTCGAGAACGGGACGGAGCACGTGCGGTTTCTGGACAGATACTTCTACAACCGGGAGGAGTACGTGCGCTTCGACAGTGACGTGGGGAAGTATGTGGAGGTGACAGAGCTGGGGCGGGGCATTGCCGAGCACTTAAACAGCCAGAAGGAACTCCTGGAGCAGAAACGGGCCGCAGTGGACACTTACTGCAGGCACAACTACGGGGTTGTTGAGCCCTTCTTAGTGCGCAGGCGCGGTGATCCAGCCCCAGGTGACTGTGTATCCATCCAAGATGGCCGCCCCGGGACAGCACAACCTGCTGGTGTGCTCCGTGGGCGGCTTCTATCCCGGGGACGTCGAGGTCCGCTGGTTCCTGAATGGGCGGGAGGAGACGGCCGGCGTGGTGTCCACGGGCCTGGTGGGCAATGGAGACTGGACCTTCCAGACCCTGGTGATGCTGGAGATGACGCCCCGGCGGGGAGACGTCTACACCTGCCACGTGGAGCACTCCAGCCTTGAGGGAGCGGTCCTCGTGGACTGGAGAGCCCAGTCTGAGTCTGCCCAGAGTAAGATGCTGAGCGGAGTCGGGGGCCTCGTGCTGGGGCTGATCTTCCTGGGGGTCGGCCTCATTGTCCACAAGAGGAGCCAGAAAGGTGA

>Smcr_DNAzoo_DAB_5

ATGGTGTGTGTCTTGCTCCCCAAGGAAGTCTGGATACAAGTTCTGGCTGTGACCCTGCTGGTGCTGAATCCCCAGGTGGCTGCAGGCAGACACAGCCCAGAGCACTTCACGGAGCAGGTAAAGCACGAGTGTCACTTTGAGAACGGGACGGAGCACGTGCGGTTTCTGGACAGATACTTCTACAACCGGGAGGAGTACGTGCGCTTCGACAGCGACGTGGGGAAGTACGTGGCGGTGACGGAGCTGGGGCGGCGCAGTGCGGAGTACTGGAACAGCCAGAAGGAACTCCTGGAGCAGAAACGGGCCGCGGTGGACACTTACTGCAGGCACAACTACGGGATTGAGCCCTTCTTAGTGCGCAGGCGCGGTGATCCAGCCCCAGGTGACTGTGTATCCATCCAAGATGGCCGCCCCGGGACAGCACAACCTGCTGGTGTGCTCCGTGGGCGGCTTCTATCCCGGGGACGTCGAGGTCCGCTGGTTCCTGAATGGGCGGGAGGAGACGGCCGGCGTGGTGTCCACGGGCCTGGTGGGCAATGGAGACTGGACCTTCCAGACCCTGGTGATGCTGGAGATGACGCCCCGGCGGGGAGACGTCTACACCTGCCACGTGGAGCACTCCAGCCTTGAGGGAGCCGTCCTCGTGGACTGGAGAGCCCAGTCTGAGTCTGCCCAGAGTAAGATGCTGAGCGGAGTCGGGGGCCTCGTGCTGGGGCTGATCTTCCTGGGGGTCGGCCTCATTGTCCACAAGAGGAGCCAGAAAGGTGA

>Smcr_DNAzoo_DAB_6

ATGGTGTGTGTCTTGCTCCCCAAGGAAGTCTGGATACAAGTTCTGGCTGTGACCCTGCTGGTGCTGAATTCCCAGGTGGCTGCAGGCAGACACAGCCCAGAAGACTTCATGATTCAGGTAAAGTACGAGTGTCACTTCGAGAACGGGACGGAGCACGTGCGGCTTGTGGCGAGAGGCATCTACAACCGGGAGGAGTGCGTGCGCTTCGACAGCGACGTGGGGAAGTTCGTGGCGGTGACGGAGCTGGGGCGGCGCAGTGCGGAGCGTGATAACAGCGTGAAGGAGAACCTGGAGAAGGCAAGGGCCGCGGTGGACACTTACTGCAGGCACAACTACAGGGTGCTTGAATTCTTTTTAGTGCCCAGGAGCGGTGATCCAGCCCCAGGTGACTGTGTATCCATCCAAGATGGCCGCCCCAGGACAGCACAACCTGCTGGTGTGCTCCGTGGGCGGCTTCTATCCCGGGGACGTCGAGGTCCGCTGGTTCCTGAATGGGCGGGAGGAGACGGCCGGCGTGGTGTCCACGGGCCTGGTGGGCAATGGAGACTGGACCTTCCAGACCCTGGTGATGCTGGAGATGACGCCCCGGCGGGGAGACGTCTACACCTGCCACGTGGAGCACTCCAGCCTTGAGAAAGCCGTCCTCGTGGACTGGAGAGCCCAGTCTGAGTCTGCCCAGAGTAAGATGCTTAGCGGAGTCGGGGGCCTCGTGCTGGGGCTGATCTTCCTGGGGGTCGGCCTCATTGTCCACAAGAGGAGCCAGAAAGGTGA

>Smcr_DNAzoo_DAB_7

ATGGTGTGTGTCTTGCTCCCCAAGGAAGTCTGGATACAAGTTCTGGCTGTGACCCTGCTGGTGCTGAATCCCCAGGTGGCTGCAGGCAGACACAGCCCAGAGCACTTCATGCTGCAGGTAAAGCACGAGTGTCACTTCGAGAACGGGACGGAGCACGTGCGGTTTCTGGAAAGACACTTCTACAACCGGGAGGAGCTCCTGCGCTTCGACAGCGACGTGGGGAAGTTCGTGGCGGTGACAGAGCTGGGGCGGGGCATTGCCGAGTACTTCAACAGCCAGAAGGAGATCCTGGAGCAGAGACGGGCCGTGGTGGACACTGCGTGCAAGCACAACTACGGGGTATCTGAGCCCTTCTTAGTGCGCAGGCGCGGTGATCCAGCCCCAGGTGACTGTGTATCCATCCAAGATGGCCGCCCCGGGACAGCACAACCTGCTGGTGTGCTCCGTGGGCGGCTTCTATCCCGGGGACGTCGAGGTCCGCTGGTTCCTGAATGGGCGGGAGGAGACGGCCGGCGTGGTGTCCACGGGCCTGGTGGGCAATGGAGACTGGACCTTCCAGACCCTGGTGATGCTGGAGATGACGCCCCGGCGGGGAGACGTCTACACCTGCCACGTGGAGCACTCCAGCCTTGAGGGAGCGGTCCTCGTGGACTGGAGAGCCCAGTCTGAGTCTGCCCAGAGTAAGATGCTGAGCGGAGTCGGGGGCCTCGTACTGGGGCTGATCTTCCTGGGGGTCGGCCTCATTGTCCACAAGAGGAGCCAGAAAGGTGA

>Smcr_DNAzoo_DBB_1

ATGGTTGATGTTTGGATTTCTGCTGGCTGTTGGAAGATTGATCTGGTAATGACATCGATGATGTTGATATCTTTATCTTGGGCCAAGGAAATCCCAGAGGATTTGTGTATCAGTCCCAAGGTGACTGATGCTTTACTAACAGCACAGAGCAAGTGTCAGGTACTTCTACAATGAAGAGGAATTTTCTCCCCTTAGATTGCTATGTGGATAAGTTTGTGATTGTGATGGAGCTGGGGTGGCCAGATACTAAATATTGGAACACTCAGAAGGAAATCATGAAGGAAGACCAAGCCTCTGTGGACACTATACATGCACAACTACAAGGCATATAAGCCTTTCTCATTGGAGAGAATCCAGCCCAGAGTGACCATCTCTCCTTTTAAGACAGAGACAGAGGCCTTGCAGCACCTGCTGCTCTGCTCTGTCACTAGCTTCTATCCTAAACAAGATCAAGATCACCTGATTCAAGAATGGATAAGAGGAGACAGCTGGGATTGTGTCCATAAATCTGATACAGAATGGAGACTGGACCTACCAGACCCTGGTCATGTTGGAAATGATTCTCCAGAGCAGAGATGTCTACACCTACAGTGTGGACCATGCCAGTGCACAGAGCTTTATCAATGTGGAATGGATCTGAATCTACTCAGAGCAAATTGCTAAGTGGAATTGGAGGGTTTGTTCTGGGACTAATCTTTCACCTGA

>Smcr_DNAzoo_DMB_1

ATGAAGTTACTGAATCTACTGCTAGTAGGCTTCAGCCTGGGTTTTTCTGGAACAGGGGCTTTTGTGACCCATGTGGAGAGTGACTGTGTACTGGATGAGGATGGATCAGTAAAGGACTTCACATATTGTATCTCCTTCAACAAGGATACGTTGACCTGCTGGGACTCAGAGACTAAAAAGATGGTCGCTGTTGATTATGGATTACTGCGTCCATTAGCTGAGCAGCTTTCTCAAATCCTTAATACTGACCATACCTTGATACACCACCTGAGCAATGGACTCCAGGATTGTGCTAATCATACAAAACCCTTCTGGGGGTCATTGACCCGAAGAACACGGTCGCCATCAGTACAAATAGCCCAGACCACACCATTCAACACAAGGGAGCAAGTGATGTTGGCCTGTTATGTATGGGGCTTCTATCCTGCTGATGTGGCCATTTCATGGTTGAAGAATGGGCAGCCAATCCCTTACAGTGGCATCCAGAGGGCTGTACAGTCTAATGGGGACTGGACTTACCAGACACGATCCTACTTGGCCCTTACCCCCTCTAGTGGGGATATTTATACTTGTTATGTAGAGCACAGTGGGACTCCCCAGGCCATCTTACAGACCTGGACCTCTGGCCTCTCTCTGAGGCAGACTGTGAAGATCTCAGTATCTGTATTGACTCTGGGACTTGGAGTCATCTTCTTCTTCCTTGGCCTGTTTTTCTGCCAAAAAGCAGGCTCCTCTGACTACACTCCTCTCTCGGGGTCCAATTATCCTGAAGGTAA

>Pegu_DAB_1_partial_exon_2-4

CACTTTATGGAGCAGGTGAAGTGCGAGTGTCTCTTCGAGAACGGGATGCAGCACGTGCAGTACGTGGAGAGACACTACAACCGGGAGGAGTTTGCACGCTATGACAGAGAGGTCAGGGAGTACGTGGCGCTGACCTCCCTGGGACAAAGGATGCGGAGTACTGGAACAGAGCCGAGAACCTGGAGCCGAGGCAGGCGGAGGTGGACACTGTGTGCAGACACAACTACCAGACAGCTCAGTGCTTCTTAGTTTGCAGGCGTGGTGACCTGAGGTGACTGTGTATCCATCCGAGCTGGCTCCCCTGGGACACCACAACCTGCTTGTCTGCTCTGTCACTGGTTTCTATCCTGGAGACATTGAGCTCAAGTGGTTCCTGAATGGGCAGGAGGAGACAGTTGGGGCTGTGTCCACAGGCCTGCTGAGCAATGGAGACTGGACCTACCAGATCCTGGTGATGCTGGAAATCCCCCCCCCCAAGCATGGAGATATCTACACCTGCCATGTGGAGCACTCCAGCCTTCAGAGACCTGTCATCTTGGAATGGAGAGCACAGTCTGAATCTGCCCAGAGTAAGATGCTGAGTGGAGTTGGGGGTCTGGTGCTGGGGCTGAGCCTCACTGTCCACTAGAGGAGTCAGAAAGGTGA

>Pegu_DAB_2_partial_exon_2-4

AGCACTTCACAGAGCAGTTTAAGTTCGAGTGTCACTTCGAGGACGCCGCGCGGCACGTGCGCTACGTGTACCGGAGCATCCACAACCGGCAGGAGATCCTGCGCTTCGACAGTGACGTCGGGGTCTTCGTGGCTGTGACCGAGCTGGGGCGGCCGGAGGAGGAGGCCTGGAACACCCCCGAGGTCCTGGAGCAGAGACGGGCCAAGGTGGACACCTACTGCAGGCTCAACTATGCTCTGTTTGAGCCCTTCTCAGTGCGCAGGAACCGTGATGGAACCTGAGGTGACGGTGTATCCATCCAAGCTGGCTCCCCTGGGACACCACAACCTGCTTGTCTGCTCTGTCACTGGTTTCTATCCCGGGGACATTGAGGTCCGCTGGTTCCTGAATGGGCAGGAGGAGACAGCTGGGGTTGTGTCCACAGGCCTGATAAGGAATGGAGACTGGACATACCAGATTCTGGTGATGCTGGAAATGACTCCCAAGCAAGGAGATGCCTACACCTGCCACGTCGAGCACTCCAGCCTTCAGAGTCCTGTCATTTTGGAATGGAAAGCACAGTCTGAATCTGCCCAGAGTAAGATGCTGAGTGGAGTTGGGGGTCTGGTGCTGGGGCTGATCTTCTTGGGGGTCGGCCTCATTGTCCACCAGAGGAGTCAGAAAGGTGA

>Pegu_DAB_3

ATGGTGGGTGTTTTGCTCCCCAAGGGCATCTGGAGAGAAGTCCTGGTTGTGACCCTACTGGTCCTGGATTCCCAGGTGGCCTCAGGCAGACACACCCCAGAACACTTCTCACTGCAGGCCAAGTCCGAGTGTTACTTTATGAGCGGGCTGCAGCCAGTGCGTTTTGTGGACAGATTCATCTACAACGGGCAGGAGATGGTGCGCTTCGACAGCGACGTCGGGAAGTACGAGGCGCTGATGGAGCTGGGGCGCGAGACCGCTGAGCACTGGAACAGGAAGCAGCGTCTGGACTACGCCCGGGCCGCAGTCCGCACGCTGTGCAGATGCAACTCCTTAGCGTTTGAGCCCTTCTCTAGGAGCTGGAAAGTTGAGCCTGAGGTGGTTGTGTATCCATCCAAGCTGGTTCTCCTGGGACACCACAACCTGCTTGTCTGCTCTGTCACTGGTTTTTATCCTGGGGACATTGAGGTCAGGTGGTTCCTAAATGGACAGGAGGAGACAATTGGGGTTGTGTCCACAGGCCTGATCAGCAATGGAGACTGGAGCTACCAGGTCCTGGTGATGCTGGAAATGACCCCCAAAAGTGGAGATGTCTATACCTGCCATGTGGAGCACTCCAGCCTTCAGAGACCTGTTATTTTGGACTGGAAAGCACAATCTGAATCTGCCCACAATAAGATGCTGAGTGGAGTTGGAGGTCTGGTGCTGGGACTTATCTTCTTGGGGATCGGCTTCATTGTCCATCAGAGGAGGAAGAAGGGTGA

>Pegu_DBB_1

ATGGTTGATATTAGGCTATGCTGGATGTTAATGACATTGATGGTATTAAGTCCACCTGTATCATGGGCCAGAGACATCCCAGAGAACTACGTGTTCCAGCACAAGGGTGAGTGCTACTTCACCCACGGCTCGGAGCGCGTGCGCTTGCTGGAGAGACACATCTACAATGACCAGGAGATCTTGCGCTTCGACAGCGCCGTGGGGCAGTACGTGGCGGTCACGGAGCTGGGGCGGCCGGAGGAGGAGAAATGGAACAGTCAGCAGAACATCCTGGACGCAAAGCGAGCCGAGTTGGACACGGTGTGCAGACACAACTACGAGATAGACAAGTCCCTCGCGGTGGACAGAAGGGTCCAGCCCAGAGTGACCATCTCCCCCTCCAAGACAGAGGCCCTGCAGCACCTGCTGGTCTGCTCTGTCACTGGCTTCTATCCAAGCAAGATCCAGGTCACCTGGTTCAAGAATGGGCAGGAGGAGACGGCTGGGATTGTGTCCACGGGAGTGATGCAGCATGGAGACTGGACCTACCAGACCCTGGTCATGTTGGAGATGGTTGCCCAGAGTGGAGACGTCTACACCTGCCGTGTGGAGCACGCCAGCCTGACCAGCCCCGTCACTGTGGAATGGAGGTCACAGTCTGAAGCTGCCCAGAGCAAATTGCTGAGCGGAGTGGGAGGCTTCGTCCTGGGGCTGATCTTCCTCAGTGTAGGACTGATCATCCACCTGAAGAACCAGAAAGGTGA

>Pegu_DMB_1

ATGAGGTTTCTCCACCTACTACTAGTGGGCTTCAACCTGGGTTTTTCAGGAGCAGGAGGGAACTTTGTGACTCATGTGGAGAGTGGCTGTGTGCTGGATGAAGATGGATCGGTAAAGGATTTCACATATTGTATCTCCTTCAACAAAGATGTGTTGACTTGCTGGAATAACGTGATTAGCAAGATGGAAATTGTTGAATTTGGGATGCTGGAACCATTAGCTCATTGGCTTGCTGACCACATTAATAACGATAGCGCCTTCATCCAAAAACTGAACAGTGGATTCCAGGACTGCGCCATTCACACAAAGCCCTTTTGGGGGTCATTGACGCAAAGGACACGGCCACCATCTGTGCTAGTAGCCCAGACTACACCATTCAACACAAGGGAGCCAGTGATGTTGGCATGTTATGTATGGGGCTTCTATCCAGCTGATGTGGCCATTGCTTGGTTGAAGAATGGACAGCCTGTCCCACACAGTAGTATCCAGAGGTCTGTACAGTCCAATGGTGACTGGACTTATCAGACACGATCCTACTTGGCTCTTACCCCCTCTAGTGGGGATATTTACACTTGCCAAGTAGAGCACAGTGGAACTTCACAGCCCATCTTACAGACCTGGACACCTGGCCTCTCTATGATGCAGACAGTGAAGATCTCTGTATCTGTATTGACTTTGGGCCTTGGCCTCATCTTCTTCTTCCTTGGCTTGGTTGCCTGTCGAAAAGCTGGCTCTTCTGACTACACTCTTCTCTCAGGGTCCAATTATCCTGAAGGTAA

>Magi_DNAzoo_DAB_1

ATGCTGTGTGTCTCCCTCTCCAGAGGCATCTGGACAGAGGTCCAGATCTGTGATCCTGCTGGCACTGAATTCCCAGGTGGCTGCAGGCAGACATGCCCCAGAGCACTTAGTGTTGCAGGGCAAGTGCGAATGTCACTTTGTGAACGGGACGCAGCACGTGCGGTTCGTGGTGAGATACATCTACCACCGGCAGGAGATCGTGCGCTTCGACAGCGCCGTGGGGGAGTTTGTGCCGCTGACGGAGCTGGTGCTGACCTTTGCTGAGGGTTGGAACAAACGGGAGGAGATCCTGGAGCGCGCCCGGGCCTCAGTGGACACTTTGTGCAGGCACAACTACGAGTTGTCTGAGCCCTTCTTACTCCCCAGGCGCTGTGATTGAGCCCGAGGTGACTGTGTATCCATCAAAGCTGGCTCCCCTGGGACACCACAACCTGCTTGTCTGCTCTGTCACTGGTTTCTATCCTGGGGACATTGAGGTCAGGTGGTTCCTGAATGGGCAGGAAGAGACAGCTGGGGTTGTGTCCACAGGCCTGGTCAGCAATGGAGACTGGACTTACCAGATGTTGGTGATGCTGGAAATGACTCCCAAGCATGGTGATGTCTACACATGCCAAGTGGAGCACTCCAGCCTTCAGAAACCTGTCATCTTGGACTGGAAAGCACAGTCTGAATCTGCCCAGAGTAAGATGCTGAGTGGAGTCGGGGGCCTTGTGCTGGGGCTGATCTTCTTTGGGGTTGGCCTCATTGTCCACAAGAGGAGTCAGATAGGTGA

>Magi_DNAzoo_DBB_1

ATGGTTGATGTTTGGATCTCTGATGGCTGCTGGAAGATTGGTCTGTTAATGACTTCAATGCTGTTGAGTTTATCTGCATCTTGGGCCAGGGACATCCCAGAGGATTTCGTGTATCAGTACAAGGGCGAGTGTTACTTCACCAACGGCTCGGAGCGGGTGCGCCTTGTGTATAGACAGATTTACAATGGAGAGGAGAATGTCCGCTTCGACAGCGACGTGGGGCACTTCGTGCCCGTGACGGAGCTGGGGCGGCCCGATGTTGAGTACTGGAACAGTCTGAAGGAGCGCCTGGAGGAATACCGAGCCTACGTGGACACGGTGTGCAGACACAACTACGAGGCATATAAGGACTTCACGTTGGACAGAAGAGTCCAGCCCAGAGTGACCATCTCCCCCTCCAAGACAGACGCCCTGCAGCACCTGCTGGTCTGCTCTGTCACTGGCTTCTATCCAAGCAAGATCAAGGTCACCTGGTTCAAGAATGGGCAGGAGGAGACAGCTGGGGTTGTGTCCACGGGTGTGATACAACATGGAGACTGGACCTACCAGACCCTGGTCATGTTGGAAATGACTCCCCAGAGCAGAGATGTCTACACCTGCAGTGTGGAGCATGCCAGCCTACAGAGCCCCATCAGTGTGGAATGGAGGGCACAGTCTGAATCTGCCCAGAGCAAACTGCTGAGTGGAATTGGAGGCTTTGTCCTGGGGCTGATCTTCCTCAGTGTAGGATCGATCATCCACCTGAAGAACAAGAAAGGTGA

>Magi_DNAzoo_DBB_2

ATGGTTGATATTTGGATCTCTGCTGGCTGCTGGAAGATTGGTCTGTTAATGACATCAATGCTGTTGAGTTTATCTGCATCTTGGGCCAGGGACATCCCAGAGGATTTCGTGTATCAGTACAAGTTTGAGTGTTACTTCAGCAACTGCACGGGGCGGGTGCGCCTTGTGGTTAGAGACATCTACAATGGCGAGGAGGATGCCCGCCTCGACAGTGAAGTGGGGCAGTTCGTGGCCCTGAGGGATCCGGGGCGGCCCGATGCTGAGCAATGGAACGGTCAGAAGGAGATCCTGGAGAAATATCGAGCCAACGTGGACACGCTGTGCAGACACAACTACGAGGCTTATAAGCGCTTCACATTGGACAGAAGAGTCCAGCCCAGAGTGACCATCTCCCCTTCCAAGACAGATGCCCTGCAGCACCTGCTGGTCTGCTCTGTCTCTGGCTTCTATCCAAGCAAGATCAAGTTGACCTGGTTCAAGAATGGGCAAGAGAAGACAGCTGGGATTGTGTCCACGGGTGTGATACAACATGGAGACTGGACCTACCAGACCCTGGTCATGTTGGAAATGACTCCCCAGAGCAGAGATGTCTACACCTGCAGTGTGGAGCATGCCAGCCTACAGAGCCCCATCAGTGTGGAATGGAGGGCACAGTCTGAATCTGCCCAGAGCAAACTGCTGAGTGGAATTGGAGGCTTTGTCCTGGGGCTGATCTTCCTCAGTGTAGGGCTGATCATCCACCTGAAGAACAAGAAAGGTGA

>Magi_DNAzoo_DBB_3

ATGGTTGATGTTTGGATCTCTGCTGGCTGCTGGAAGGTTGGTTTGTTAATGACATCAATGCTGTTGAGTTTATCTGCATCTTGGGCCAGGGACATCCCAGAGGATTTCGTGTATCAGAACAAGGGTGAGTGTTACTTCACCAACGGCACGGAGCGGGTGCGCCTTGTGTATAGACAGATTTACAATGGAGAGGAGAATGTCCGCTTCGACAGCGACGTGGGGCACTTCGTGGCGGTGACGGAGCTGGGGCTGTGCGATGTTGAGTACTGGAACAGTCAGGAGGCGCTCCTGGAGGAATATCGAGCCTACGTGGACACGCTGTGCAGACGCAACTACCGGAGAAATAAGCCCTTCACGGTGGACAGAAGAGTCCAGCCCAGAGTGACCATCTCCCCCTCCAAGACAGACGCCCTGCAGCACCTGCTGGTCTGCTCTGTCACTGGCTTCTATCCAAGCAAGATCAAGGTCACCTGGTTCAAGAATGGGCAGGAGAAGACAGCTGGGATCGTGTCCACGGGTGTGATACAACATGGAGACTGGACCTACCAGACCCTGGTCATGTTGGAAATGGCTCCCCAGAGCGGAGATGTCTACACCTGCAGTGTGGAGCATGCCAGCCTACAGAGCCCCATCAGTGTGGAATGGAGGGCACAGTCTGAATCTGCCCAGAGCAAACTGCTGAGTGGAATTGGAGGCTTTGTCCTGGGGCTGATCTTCCTCAGTGTAGGGCTGATCATCCACCTGAAGAACAGGAAAGGTGA

>Magi_DNAzoo_DBB_4

ATGGTTGATGTTTGGATCTCTGCTGGCTGCTGGAAGATTGGTCTCTTAATGACATCAATGCTGTTGAGTTTATCTGCATCTTGGGCCAGGGACATCCCAGAGGATTTCGTGTTGCAGTACAAGGCTGAGTGTTACTTCACCAACGGCTCGGAGCGGGTGCGGTTTGTGGTTAGACTCATGTACAATGGCGAGGAGAATGTCCGCTTCGACAGCGACGTGGGGCACTACGTGGCCGTGACGGAGCTGGGGCGGCCCGATGCTGAGTACTGGAACGGTCAGAAGGATTCCCTGGAGGAACACCGAGCCTACGTGGACACGCTGTGCAGACACAACTACCAGACAAATAAGCCCTTCACGGTGGACAGAAGAGTCCAGCCCAGAGTGACCATCTCCCCCTCCCAGACAGACGCCCTGCAGCACCTGCTGGTCTGCTCTGTCACTGGCTTCTATCCAAGCAAGATCAAGGTCACCTGGTTCAAGAATGGGCAGGAGGAGACAGCTGGGATCGTGTCCACGGGTGTGATACAACATGGAGACTGGACCTACCAGACCCTGGTCATGTTGGAAATGACTCCCCAGAGCAGAGATGTCTACACCTGCAGTGTGGAGCATGCCAGCCTACAGAGCCCCATCAGTGTGGAATGGAGGGCACAGTCTGAATCTGCCCAGAGCAAACTGCTGAGTGGAATTGGAGGCTTTGTCCTGGGGCTGATCTTCCTCAGTGTAGGGCTGATCATCCACCTGAAGAACAAGAAAGGTGA

>Magi_DNAzoo_DBB_5

ATGGTTGATATTTGGATCTCTGCTGGTTACTGGAAGATTGGTCTGTTAATGACATCAATGCTGTTGAGTTTATCTGCATCTTGGGCCAGGGACATCCCAGAGGATTTCGTGTTTCAGGACAAGGCTGAGTGTTACTTCACCAACGGCACGGAGCGGGTGCGGTATGTGGCTAGAGACTTCTACAATGGCGAGGAGACTGCCCGCTTCGACAGCGACGTGGGGCACTTCGTGGCGGTGACGGAGCTGGGGCGGCGCGATGTTGAGTACTGGAACGGTCAGGAGGAGATCCTGGAGAGATTTCGAGCCTACGTGGACACGCTGTGCAGACACAACTACGAGGTACACAAGCCCTTCACGTGGGACAGAAGAGTCCAGCCCAGAGTGACCATCGCCCCCTCCAAGACAGACGCCCTGCAGCACCTGCTGGTCTGCTCTGTCACTGGCTTCTATCCAAGCAAGATCAAGGTCACCTGGTTCAAGAATGGGCAGGAGGAGACAGCTGGGATCGTGTCCACGGGTGTGATACAACATGGAGACTGGACCTACCAGACCCTGGTCATGTTGGAAATGACTACCCAGAACAGAGATGTCTACACCTGCAGTGTGGAGCATGCCAGCCTACAGAGCCCCATCAGTGTCAAATGGAGGGCACAGTCTGAATCTGCCCAGAGCAAACTGCTGAGTGGAATTGGAGGCTTTGTCCTGGGGCTGATCTTCCTCAGTGTAGGGCTGATCATCCACCTGAAGAACAAGAAAGGTGA

>Magi_DNAzoo_DCB_1

ATGGAACATGTGGAGTTTCTGGGAGGCCCTGTATGACAGTCATACTGATAGTGCTGAGCACACCCACAGACTGAGGTAGGGACATTCCAAGTAAAGAATTACCTACATCAGATGGGGTCTGAGGGTCACATGATCAATGAAACCCAACAAGACTCATCTACAACCAGGTGGAGTTTGTGCACTTTGATAGTGATGTGGGACTATTTCAGACAAGCTGTGGAAATCCCAAGTCCAGAAATGAAACAGTAAGAAGGAGATAGTCAAGCATGCAAGGTCCATAGTGAATGTGTGCAGAGACAATTTTCTTTTATATGACAAATTCACACCTCTAGTGAAAGTTTTACCACCAAAGATACAACCACCTGGACACCACAACCTGCTCCTCTGTTCTGTGATATTTCTATCCTGGTGAGATCAAGGTCAATTGATTCAGAATCGCGAAAGAAGAGAAGACTGGAATCCTGTCCACAGACCGAATTCAGCACAGTGCCTGCACCTTCCAAACCCTTGTGATACTGGAAATGGCCCTCCAAAGAGGAGATGTCTTTACTTGCCATGTGGACCATGCCAGCTTGCAGAATCCTGTCACTGTAAACTGGAGACCACATTCTGAATCTTCCTGGACTAAGAGGCTGACTGGAATTGAGGCCTTGGTATTTGGACTGATCTCACTTGGAGTGAGACTTTACTTGTCATCCACCTCAGAAATTTGAAAGATTCTTATTCTGGTACCAAAGAAGACCCAGATTTTGAAGGGATTGTGAACATTGCCTCATTGCAACAAGATTTTCCCAGAACTGTTGCCCAGTCCTAG

>Magi_DNAzoo_DMB_1

ATGACATTACTCTACCTGCTACTAGTGGGCTTCAGCCTGGGTTTTTCAGGAACAGGGGGCTTTGTGACTCATGTGGAGAGTGACTGTATACTGGATGAAGAAGGATCCGTAAAGGACTTCGAATATTGCATCTCCTTCAACAAGGATGTGTTGACCTGCTGGAACTCAGATACTAGCAAGATGGTCACTGTTGAATTTGGAATACTGCGTCCATTAGCTGACTGGCTTTCTGACACCCTCAATAATGATACTGCTTTGATAAACCGCCTGAGTGAAGGATTCCAAAACTGTGCCAGTCATACAAAGCCCTTCTGGGGATCACTGACCCACAGGACACGGCCACCATCAGTGCAAGTAGCTCAGGTCACACCATTCAACACAAGGGAGCCAGTGATGCTGGCCTGTTATGTATGGGGCTTCTATCCTGCTGATGTGGCCATTTCGTGGTTGAAGAATGGGCAGCCTGTCCCACACAGTGGCATCCAGAAGGCTGTACAATCCAATGGGGACTGGACTTATCAGACACGATCCTACTTGGCCCTTACCCCCTCTAGTGGGGATATTTACACTTGCTTTGTAGAGCACGGTGGGACTTCCCAGACCATCTTACAGACCTGGACACCTGGCCTCTCCCTGAAGCAAATAGTGAAGATCTCTGTATCTGTACTGACTCTGAGCTTTGGTCTTATCTTCTTCCTCCTTGGCCTGGTTGCACGTAGAAAAGCTGGCTCCTCTGACTACTCTCCTCTCTCGGGGTCCAATTATCCTGAAGGTAA

>Davi_DNAzoo_DAB_1

ATGGGGTGTGTCTTGCTCCCCAAGGAAGTCTGGATAGAAGTTCTGGCTGTGACTCTGCTGGTGCTGAATGCCCAAGTGGCTGCAGGCAGACACAGCCCAGAGGACTTCATGGTGCAGGAAAAGTACGAGTGTCACTTTGAGAATGGGATGGAGCACGTGCGGTATGTGCACAGAGACTTCTACAACCGGGAGGAGATTACACGCTTCGACAGCGACGTGGGGAAGTATGTGGCGCTGACGGAGCTGGGGCGGCGGGATGCTGAGTATTGGAACAGCCAGAAGGAGATCCTGGAGCGGAGACCGGCTTTGGTGGACACTTTGTGCAGACACAACTATGTGGGGATTGAGCCCTTCTCATTGCGCAGGAGCGGTGACCTGAGGTGACTCTGTATCCATCAAAGATGGCTCCCCCGGGACACCACAACCTGCTCTTCTGCTCCATCAGTGGTTTCTATCCTGGGGACATTGAGGTCTGGTGGTTCCTGAATGGGCGGGAGGAGACGGCAGGGGTGGTGTCCACCGGCTTGATGGGCAATGGAGACTGGACTTACCAGACATTGGTGATGCTGGAGATGACTTCCCAGCATGGAGATGTCTACACCTGCCACGTGAAGCACTCCAGCCTTCAGAGACCCATCATCTTGGACTGGAAAGCCCAGTCTGAGTCTGCCCAGAGTAAGATGCTGAGCGGAGTTGGGGGCCTTGTTCTGGGGCTGATCTTCTTGGGGGTTAGCCTCATTGTCCACAAGAGGAGCCAGAAAGGTGA

>Davi_DNAzoo_DBB_1

ATGGTTGATGTTTGGATTTCTGCTGGCTCTTGCAAGATTGATCTGGTAATGACATCAATGATGTTGATACCTTTATCTTGGGCCAAAGAAATCCCAAAGGATTTCGTGTACCAGTTCCAGGGTGACTGATACTTTACTAACAGCATGGAACAAGTGTCAGATACTTCTACAATGGCCAGGAATTTTCTCTGCTTGGACTGCTATATGGGTAAGTGTGTGATTGTGATGGAGCTGGGGTGGCCAGATACTGAATACTGGAACACTCAGAAGGAAATCATGAAGGAAGACCAAGTCTCTGTGGACACTATGCATCTACAACTTCCAGCCCAGAGTGACCATCTCTCCTTTCAAGACAGAGGACCAGGCCTTGCAGCACCTGCTGCTCTGCTCTCACCAGCTTCTATCCTAAACAAGATCAAGGTCACCTGATTCAAGAATAGATCAGAGGAAACATTGTCCATAATAATCCCACAATGGGATTGTGTCCATAAATCTGATACAGAATGGAGACTGGATCTACCAGACCTTTGTCGTGTTGGAAATGATTCTCCATAGCAGAGATGTCTACACATACAGTGTGGACCATGCCAGTGCACAGAGCTTCATTAGTGTGTAATGGATCTGAATCTACTCAGAGCAAATTGCTAAGTGGAATTGGAGGCTTTGTCCTGGGGCTGGTCT

>Davi_DNAzoo_DMB_1

ATGAAGTTACTGCACCTACTGCTAGTAGGCTTCAGCCTGGGTTTTTCTGGAGCAGGGGCTTTTGTGACCCACGTGGAGAGTGACTGTGTACTGGATGAGGATGGATCAGTAAAGGACTTCACATATTGTATCTCCTTCAACAAGAATGTGTTGACCTGCTGGGACTCAAAGATTAAAAAGATGGTCACTGTTGATTATGGTCTACTGAAGCCACTTGCTGAATATCTTTCTCAATCCCTTAATAACAACAGTGCCTTGATACACCACCTGAGCAATGGATTCCAGGATTGTGCCAGTCACACAAAACCTTTCTGGGGGTCATTGACCCAAAGAACACGGTCACCATCAGTGCAAATAGCCCAGACCACACCATTCAACACAAGGGAGCAAGTGATGTTGGCCTGCTATGTATGGGGCTTCTATCCTGCTGATGTGGCCATTTCATGGTTGAAGAATGGGCAGCCAATCCCTAACAGTGGCATTCAGAGAGCTGTACAGTCTAATGGGGACTGGACTTACCAGACACGATCTTACTTGGCCCTTACCCCCTCTAGTGGGGATATTTATACTTGCCATGTAGAGCACAGTGGGAGTTCCCAGGCCATCTTACAGACCTGGACCTCTGGCCTCTCTCTGAAGCAGACCGTGAAGATCTCTGTATCTGTATTGACTCTGGGACTTGGCCTCATCTTCTTCTTCCTTGGCCTGGTTTTCTGCCAAAAAGCAGGCTCCTCTGACTACACTCCTCTCTCGGGGTCCAATTATCCTGAAGGTAA

>Pogi_DNAzoo_DAB_1

ATGCCATGTGTCTCCTTCTCCAGAGGCATCTGGACAGAGGTTCTGGCTGTGACCCTGCTGGTGCTAACTTCCCAGGTAGTTGCAGGCAGACATGCCCCAGAGCACTTCACGGAGCAGTTGAAGTTCGAATGTTACCTTGTGAACTGGACGGAGCACGTGCGGTTTGTGCTCAGATACATCTACAACCGGGAGGAGTACGTGCGCTTCGACAGCGACGTGGGGGAATTCCTGGCGGTGACAGAACTGGGCCGGCGCAGCGCTAAGTATTACAACGGACTGGAGGATGAACTGGAGCAGAAACGGGCCTACGTGGACACTTTGTGCAGGCACAACTACGGGGTTTTTCACAGATTCTTAGCCCCCAGGCGCGTTGAGCCCAAGGTGATTGTGTTTCCATCAAAATTGGCTCCTCTGGGACACCACAACCTGCTTGTCTGCTCTGTCACTGGTTTCTATCCTGGGGACATTGAGGTCAGGTGGTTCCTGAATGGGCAGGAGGAGATAGCAGGGATTGTGTCCACAGGCCTGATCAGCAATGGAGACTGGACTTACCAGATCTTGGTGATGCTGGAAATGACCCCCAAGCGTGGTGATGTCTACACCTGCCAAGTGGAACACTCCAGCCTTCAGAGACCTATCGTCTTGGACTGGAAAGCACAGTCTGAATCTGCCCGGAGCAAGATGCTGAGTGGAGTTGGGGGACTCCTGCTGGGGCTGTTATTCTTTGGAGTTGGCCTCATTGTCCTCAAGAGGAGTCAGAAAGGTGA

>Pogi_DNAzoo_DAB_2

ATGCTGTGTGTCTCCCTCTCCACAGGTATCTGGACAGAGGTTCTGGCTCTGACCCTGCTGGCACTAAATTCCAAGGTGGCTGCTGGCAGACATGCCCCAGAGCACTTCGCGGTGCAGGCAAAGTCCGAGTGTTACTTTGTGAACGGGACGCAGCACGTGCGGTTCATGGACAGATACTTCTACAACCGGGAGGAGGCTGCGCGCTTCGACAGCGACGTGGGGGAGTATGTGGCGGTGTCGGAGCTGGGCCATCCTGATGCTGAGTATTGGAACAGCCAGAAGGAGATCCTGGAGCAGAAACGGGCCTACGTGGACACTTTGTGCAGGAGCAACTACGAGTTGTCTGAGCGCTTCTTGGTCCCCAGGCGCGGTGATTCAGCCTGAGGTGACTGTGTATCCATCAAAGGTGGCTCCCCTGGGACACCACAACCTGCTTGTCTGCTCTGTCACTGGTTTCTATCCTGGGGACATCGAGGTCAGGTGGTTCATGAATGGGCAGGAGGAGACAGCTGGGGTTGTATCTACAGGCCTGGTCAGCAATGGAGATTGGACTTACCAGATCTTGGTGATGCTGGAAATGACCCCCAAGCATAGTGATGTCTACACCTGCCAAGTGGAGCACTCCAGTCTTCAGAAACCTGTCATCTTGGACTGGAAAGCACAGTCTGAATCTGCCCAGAGTAAGATGCTGAGTGGAGTCGGGGGGCTTGTGCTGGGGCTGATATTCTTTGGGGTTGGCCTCATTGTCCACAAGAGGAGTCAAAAAGGTGA

>Pogi_DNAzoo_DAB_3

ATGCTGTGTGTCTTGCTCCCCAGGGGCATCTGGACAGAGGTTCTGGCTGTGACCCTGCTGGTACTGACTTCCCAGGTGACTGCAGGCAGACATGCCCCAAAGCACTTCACGGAGCAGGTGAAGGGTGAGTGTCACTTTGAGAACGGGACGCAGCACGTGCGGTTTGTGGTGAGACATATCACGAACCGGCAGGAGAACGTGCACTTCGACAGCGACGCGGGGGAGTTTGTGGCGGTGACGGAGCTGGGCCGGGGGGATGCCGAGCTTTGGAACAGCCAGAAGGACTACGTGGAGCAAGCACGGGCTGCGGTGGACACTTTGTGCAGGCGCAACTACAAGATACTTGAGCCCTTCTTAGTCTCCAGGCGCGGTGATTGAGCCCGAGGTGACTGTGTATCCATCAAAGCTGGCTGCCCTGGGACACAACAACCTGCTTCTCTGCTCTGTCACTGGTTTCTATCCTGGGGACATTGAGGTCAGGTGGTTCCTGAATGGGCAGGAGGAGACAGTTGGGGTTGTGTCCACAGGCCTGATCAACAATGGAGACTGGACTTACCAGATCTTGGTGATGCTGGAAATGACCCCCAAGAGTGCTGATGTCTACACCTGCCAAGTGGAGCACTCCAGCCTTCAGAAACCTGTCATCTTGGATTGGAAAGCACAGTCTGAATCTGCCCAGAGTAAGATGCTGAGTGGAGTCGGGGGTCTCGTGCTGGGGCTGATCTTCTTTGGGGTTGGCCTCATTTTCCACAAGAGGAGTCCGAAAGGTAA

>Pogi_DNAzoo_DAB_4

ATGCTGTGTGTCTCCCTCTCTAGAGGCATCTGGATAGAGGTTCTGGCTGTGACCCTGCTGGTGCTGAATTCCCAGGTGACTGCAGACAGACATGCCCCAGAGCACTTCACGGAGCAGGCGAAGGCCGAGTGTCACTTTGAGAACGGGACGCAGCACGTGCGGTTTGTGTTGAGAGACATCACGAACCGGCAGGAGAACGTGCGCTTCGACAGCGACATCCGAGAGTTCGTGGCGGTGTCGGAGCTGGGGCGGAGCGCGGCTGAGCAGTGGAACAGCCAGAAGGATTTCCTGGAGCGCAGACGGGCGAAGGTGGACACTTACTGCAGGCACAACTACGAGCTGTCTGAGCGCTTCTTAGTCCCCAGGCGCGGTGATTGAGCCTGAGGTCACTGTGTATCCATCAAAGCTGGCTCCCCTGGGACACCACAACCTGCTTGTCTGCTCTGTCACTGGTTTCTATCCTGGGGACATTGAGGTCAGGTGGTTCTTGAATGGGCAGGAGGAGACAACTGGGGTTGTGTCCACAGGCCTGGTCAGCAATGGAGACTGGACTTATCACATGTTGGTGATGCTGGAAATGACCCCCAAGCATGGTAATGTCTACACATGCCAAGTAGAGCACTCCAGCCTTCAGAAACCTGTCATCTTGAATTGGAAAGCACAGTCTGAATCTGCCCAGAGTAAGATTCTGAGTGGACTTGGGGGGCTCATGCTGGGGCTGATATTCTTTGGGATTGGCCTACTTGTCTACAAGAGGAGTAAGAAAGGTGA

>Pogi_DNAzoo_DAB_5

ATGCTTTTTGTCCCCCTCTACAGAGGCATGTGGACAGAAGTCCTGGCTGTGACCCTGCTGGCATTGAATTCCCAGGTGGCTGCAGGCAGACATGTCCCAGAGCACTTCACGGAGCAGGTGAAGGGCGAATGTCACTTTGTGAACGGGACGCAGTACGTGCGGTTTGTGCTGAGACACTTCTACAACCGGCAGGAGAATGTGCGCTTCGACAGCGACGTGGGGGAGTTTGTGGCGCTAAAGGAGCTGGGCCGTCCTGATGCCGAGCTTTGGAACAGCCAGAAGGACTACGTGGAGCGCGCACGGGATGCGGTGGACACTTTGTGCCGGCACAACTACAGGGTGTTTGAGCCCTTCTTACTCTCCAGGCGTGGTGATTCAGCTTGAGGTGACTGTGTATCCATCAAAGCTGGCTCCCCTGGGACACCACAACTTGCTTGTCTGCTCTGTCACTGGTTTCTATCCTGGGGACATTGAGGTCAGATGGTTCCTGAATGGGCAGGAAGAGACAGCTGGGATTGTGTCTACAGGCCTGATCAGCAATGGAGACTGGACTTACCAGATCTTGGTGATGCTGGAAATGACCCCCATGCATGGTGATGTCTACACATGCCAAGTGGAGCACTCCAGCCTTCAGAAACCTGTCATCTTGGACTGGAAATCACAGTCTGAATTTGCCCAGAGTAAGATGCTGAGTGGAGTCGGTGGCCTTGTGCTGGGGCTGATCTTCTTTGGGGTTGGCCTCATTGTCCACAAGAGGAGTCAGAAAGGTGA

>Pogi_DNAzoo_DAB_6

ATGGTGTGTATCTTGTTCCCCCAGGGTATCTGGATAGAGGTATTGGTTGTGACACTGCTGGTGCTGAAAGCTCAGGTGACTGTAGGCAGACATGCCCCAGAGCACTTCACGGAGCAGGTGAAGGGCGAATGTTACTTTGTGAACGGGATGGAGCACGTGCGGTTTGTGCTGAGGGGCATCTACAACCGCGAGGAGTACGTGCGCTTCGACAGCGACGTGGGGGAGTTTGTGGCAGTGACGGAACTGGGGCGTCGCACTGCGGAGTTCGGTAACAGCCAGAAGGAGTTCATGGACCACTTACGGGCGTCGGTGGATACTTACTGCAGGCACAACTACGAGGGGATCGAGCCCTTCACAGTGCCCAGGAGTGGTGATTCAGCCTGAGGTGACTGTGTATCCATCAAAGGTGGCTCCCCTTGGACACCACAACTTGCTTGTCTGCTCTGTCACTGGTTTCTATCCTGGGGACATTGAGGTCAGGTGGTTCCTGAATGGGCAAGAGGAGACAACTGGGGTTGTGTCCACAGGCCTGATCAGCAATGGAGACTGGACTTACCAGATCCTGGTGATGCTGGAAATTATCCCCAAGCATGGAGATGTCTACACTTGCCAAGTGGAACACTCCAGCCTTCGGAGACCTATTATTTTTGATTGGAAAGCACGGTCTGAATCTGCCCAGAGTAAGATGCTGAGTGGAGTTGGGGGGCTCGTGCTGGGGCTGATATTCTTTGGGGTTGGCCTCATTGTCCACAAGAGGAGTCAGAAAGGTGA

>Pogi_DNAzoo_DBB_1

ATGGTTGATGTTTGGACCTCTGCTGGCTGCTGGAAGATTGGTCTGTTAATGACCTCAGTGCTGTTGAGTTTATCTGCATCTTGGGCCAGGGACATCCCAGAGGATTTCGTGTATCAGTACAAGGCTGAGTGTTACTTCACCAACGGCACGGAGCGCGTGCGCTTTGTGGTTAGAGTCATGTACAATGGCGAGGAGAATGCCCGCTTCGACAGCGACGTGGGGCACTTCGTGGCGGTGACGGAGCTGGGGCGGCCAAGTGCTGAGTACTGGAACAGTCAGGAGGACATCATGGAGCAACATCGAGCCTACGTGGACACGCTGTGCAGACACAACTACGAGGCAACTAAGCCCTTCACGTTGGACAGAAGAGTCCAGCCCAGAGTGACCATCTCCCCCTCCAAGACAGAGGCCCTGCAGCACCTGCTGGTCTGCTCTGTCACTGGCTTCTATCCAAGCAAGATCAAGGTCACCTGGTTCAAGAATGGGCAGGAGGAGACAGCTGGGATCGTGTCCACGGGTGTGATACAACATGGAGACTGGACCTATCAGACCCTGGTCATGTTGGAAATGATTCCCCAGAGCAGAGACGTCTACACCTGCAGTGTGGAGCATGCCAGCCTGCAGAGCCCCATCATTGTGGAATGGAGGGCACAGTCTGAATCTGCCCAGAGCAAACTGCTGAGTGGAATTGGAGGCTTTGTCCTGGGGCTGATCTTTCTCAGTGTAGGATCAATCATCCACCTGAAGAACAAGAAAGGTGA

>Pogi_DNAzoo_DBB_2

ATGGTTGATATTTGGACCTCTGCTGGCTGCTGGAAGATTGGTCTGTTAATGACCTCAATGCTGTTGAGTTTATCTGCATCTTGGGCCAGGGACATCCCAGAGGATTTCGTGTTTCAGTACAAGGCTGAGTGTTACTTCACCAAGGGCACGGAGCGCGTGCGCCTTGTGTTTAGAGTCATGTACAATGCCGAGGAACATGTCCGCTTCGACAGCGACGTGGGGCACTTCGTGGCCCTGACGGAGCTGGGGCGGCCAATTGCTGAGAACTGGAACCGTCAGGAGGACAGCCTGGAGGAATATCGAGCCTACGTGGACACCGTGTGCAGACACAACTACGAGGCAACTAAGCCCTTCACGTTGGACAGAAGAGTCCAGCCCAGAGTGACCATCTCCCCCTCCAAGACAGAGGCCCTGCAGCACCTGCTGGTCTGCTCTGTCACTGGCTTCTATCCCAGCAAGATCAAGGTCACCTGGTTCAAGAATGGGCAGGAGGAGACAGCTGGGATCGTGTCCACGGGTGTGATACAACATGGAGACTGGACCTACCAGACCCTGGTCATGTTGGAAATGACTCCCCAGAGCAGAGATGTCTACACCTGCAGTGTGGAGCATGCCAGCCTACAGAGCCCCATCAGTGTGGAATGGAGGGCACAGTCTGAATCTGCCCAGAGCAAACTGCTGAGTGGAATTGGAGGCTTTGTTCTGGGGCTGATCTTCCTCAGTGTAGGGCTGATCATCCACCTGAAGAACAAGAAAGGTGA

>Pogi_DNAzoo_DBB_3

ATGGTTGATGTTTGGACATCTGCTGACTGCTGGAACATTGGTCTCTTAATGATATCAATGCTGTTGAGTTTATCTGCATCTTGGGCCAGGGGCATCCCAGAGGATTTCGTGTTTCAGTACAAGTTTGAGTGTTACTTCACCAACGGCACGGAGCGGGTGCGCCTTGTGGTTAGGGGGATCTACAATGGCGTGGAGAATGTCCGCTTCGACAGCGACGTGGAGCAGTTCCTGGCGGTGACGGAGCTGGGGAGGCCCGATGCTGAATACTGGAACAGTCAGGAGGAGATCCTGGAGGAATATCGAGCCTACGTGGACACGCTGTGTAGACACAACTACAACACTGACAAGCCCTTCACGGTGGACAGAAGAGTCCAGCCCAGAGTGATCATCTCCCCCTCCAAGACAGAGGCCCTGCAGCACCTGCTAGTCTGCTCTGTCACTGGCTTCTATCCCAGCAAGATCAAAGTCATCTGGTTCAAGAATGGGCAGGAGGAGACAGCTGGGATCGTGTCCACGGGTGTGATACAACATGGAGACTGGACCTACCAGACCCTGATCATGTTAGAAATGACTCCCCAGAGCAGAGATGTCTACACCTGCAGTGTGGAGCATGCCAGCCTACAGAGCCCCATCATTGTGGAATGGAGGGCACAGTCTGAATCTGCCCAGAGCAAATTGCTGAGTGGAATTGGAGGCTTTGTGCTGGGGCTGATCTTCCTCATTGTAGGGTTGATCATCCACTTGAAGAACAAGAAAGGTGA

>Pogi_DNAzoo_DCB_1

ATGTGGAGTTTCTGGGAGGCCCTGTGTGACAGTCACACAGATAGTGCTGAGCACACCCACAGACTGAGGTAGGGACATTCCAAGTAAAGAATTACCTACATCAGATGGGGTCTGAGGGTCGCATGATCAATGGAACCCAACAAGACTCATCTACAACCAGGTGGAGTGTGTGCACTTTAACAGTGATGTGGGACTATTTCAGACAAGATGTGGAAATTCCAAGTCCAGAAATGAAACAGTAAGAAGGAGATAGTCAAGTATGCAAGGTCCATAGTGAATGTGTGCAGAGACAATTTTCTTTTATATGACAAATTCACAGGGCAAAGGAAAGTCCAGCCCCTAATGAAAGTTTTACCCCCAAAGATACAACCACTTGGGCACCACAACCTGCTCCTCTGTTTTGTGATGTTTCTATCCTGGTGAGATCAAGATCAATTGATTCAGGATTGCAAAAGAAGAGAAGACTGGAGTCCTGTCCACAGACCAAATTCAGCACAGTGACTGGACCTTCTAAACCTTTGGGATGCTGGAAATGGCCCCCCAAAGAGGAGACGTCTTTACTTGCCATGTGGTCCATGTCAGCTTGCAGAGCCCTGTCACTGTAAAATGGAGAGCATATTCTGAATCTTCCTGGACTAACAGGCTGACTGGAATTGAGGGTTTGGTATTTGGACTGATCTTACTTGGAGTGAGACTTT

>Pogi_DNAzoo_DMB_1

ATGACATCACTCTACCTGCTACTAGTGGGCTTCAGCCTGGGTTTTTCAGGAACAGGGGGCTTTGTGACTCATGTGGAGAGTGGCTGTATACTGGATGAAGAAGGATCCGTAAAGGACTTTGAATATTGTATCTCCTTCAACAAGGATGTGTTGACCTGCTGGGACCCAGATATTAACAAGATGGTCACTGTTGATTTTGGAATACTGCATCCATTAGCTGAAGAGCTTTCTGATGCTCTCAGTAATGATACTGCTTTGATAAACCGCCTGAGCCAAGGATTCCAGGACTGTGCCAGTCACACAAAGCCCTTCTGGGGAGCACTGACCCACAGGACACGGCCACCATCAGTGCAAGTAGCTCAGGTCACGCCATTCAACACAAGGGAGTCAGTGATGCTGGCCTGCTATGTATGGGACTTCTATCCTGCTGATGTGGCCATTTCATGGTTGAAGAATGGGCAGCCTGTCCCACACAGCGGCATCCAGAAGGCTGTACAATCCAATGGGGACTGGACTTATCAGACGCGATCCTACTTGGCTCTTACCCCCTCTAGTGGGGATATTTACACTTGCTCTGTAGAGCACAGTGGGACTTCCCAGACTATCTTACAGACCTGGACACCTGGCCTGTCCCTGAAGCAGATAGTGAAGATCTCTGTATCGGTATTGACTCTGAGCTTTGGTCTTATCATCTTCTTCCTTGGCCTGGTTGCACGCAGAAAAGCTGGCTTCTCTGACTACTCTCCTCTCTCGGGGTCCAATTATCCTGAAGGTAA

>modo_DNAzoo_DAB_1

ATGGTGAGCGCGCAGCCTCTCGGGGGCATCTGGATGGAGGTTCTGGTGGTGACTCTGCTGGTGCTGACAGCCCAGGTGGCTGCGGACAGACGCCCCCCAAAGCACTTTACAGAGTATTTCATATCGGAGTGCTACTTCATCAACGGGACGGAGCAGGTGTGGCTTGTGCAGAGATACATCACTAACGGAGAGGAAACCGTGCGCTTCGACAGCAACGTGGGAGTGTTCGAGGCGGTGACAGAGTCGGGGCGGCCGGATGCTGAGCATTGGAACAGCCAGAAGGAGTTCCTGGAGAACTTACGGGCCGCAGTGGACACTGCCTGCAGGCACAACTACAAGATCTCTGAGCCCTTCTTAGTGCGCAGGCGCGGTGATGGAGCCGGAGGTCCTGGTGTATCCATCCAAGACGGCTCCTGTGGGCCACCACAACCTGCTGGTGTGCTCTGTGAGCGGCTTCTACCCCGGGGCCGTGGCGGTCACGTGGTCCGTGAATGGGCAGGAGCAGAGGGCTGGGGTCGTGTCCACAGGCCTGATGCGCAATGGCGACTGGACCTTCCAGACCCTGGTGATGCTGGAGGTGACCCCCCAGCGCGGAGATGTCTACACCTGCCACGTGGAGCACTCCAGCCTGCAGAAGCCTGTCCTGGTGGCCTGGAGTGCCCAGTCAGAGTCTGCCCAGAGTAAGATGCTGAGTGGTGTCGGGGGCTTCGTGCTAGGCCTCATCTTCTTTGGGGTTGGCCTCATTGTCCACATGAGGAGTCAGAAAGGTGA

>modo_DNAzoo_DBB_1

ATGTTTGCTGTTTGTATCTCTGCTAGTTTCTGGAAAATTGGTCTGTTAATGACATTGATGCTGTTTTGTCTACCAGTCTCTTGGGCCAGGGACATCCCAGAGGATTTTGTGATCCAGAGCAAGGGTCTGTGTTACTTCACCAACGGCTCCGAGCAGGTGCGGCTTGTGGTGAGATACATCTACAATGACCAGGAGATTGCCCGCTATGACAGTAAGCTGGGGAAATACGTGGCTGTGACAGAGCTGGGGCGGCCAACCGCCGAGTATTGGAACAGCCAGAAAGACATCCTGGAGCGAAGAGAAGCCGAAGTGGATACGGTGTGCAGACACAACTACGAGATAGACAAGCACATAATTCTGGCGAAAAGGGAGCCCAGCCCCACGTGACCGTCTCCCCCTCCAAGCAAGAGGCCCAGCAGCACCTGCTTGTCTGCTCTGTGACTGGCTTCTATCCAAGCAAGATCAAGGTCACATGGTTGAAGAACGGGCAGGAGCAGACAGCTGGGGTTATCTCCACGGAGGTGATCCAGAATGGAGACTGGACCTATCAGACCCTGGTCATGTTGGAGACGATTCCCCAGAGCAGAGACATCTACACCTGCAGTGTGGAGCATGCCAGCCTCCAGAGCCCCATCACTGTGGAATGGAGTGGGCTCAGTCTGGATCTGCCCAGAGCAAACTTGTGAGTGGAGTTGGAGGCTTTGTCCTGGGGCTGATCTTCCTCAGTGTAGGACTGATCCTCCACCTGAAGAGCCAGAAAGGTGA

>modo_DNAzoo_DBB_2

ATGGTTGCTGTTTGGATCTCTACTAATTTCTGGAAAATTGGTCTGTTAATGACATTGATGATGTTTTGCCTACCAGTCTCTTGGGCCAGGGACATCCCAGAGGATTTTGTGTACCAGTACAGAGCCCTGTGTTACTTCACCAACGGCTCCAAGCAGGTGCGGCTTGTGAATAGACACTTCTACAATGACCAGGAGACTGTCCGCTATGACAGTAAGCTGGGGAAACACGTGGCTGTGACAGAGCTGGGGCGGCGAGACGCGGAGTATTGGAACAGCCGGAAAGACATCCTGGAGCGAAAACGAGCCTCATTAGACACGGTGTGCAGACACAACTACGAGGCATACAAGCCCTTCACTTTGGAAAGAAGAGcccagCCCCGCCTGACCGTCTCCCCCTCCAAGCAAGAGGCCCAGCAGCACCTGCTTGTCTGCTCTGTGACTGGCTTCTATCCAAGCAAGATCAAGGTCACATGGTTGAAGAACGGGCAGGAGCAGACAGCTGGGGTTATCTCCACGGGGCTGATCCAACATGGAGACTGGACCTATCAGACCCTGGTCATGTTGGAGACGATTCCCCAGAGCAGAGACATCTACACCTGCAGTGTGGAGCATGCCAGCCTCCAGAGCCCCATCGCTGTGGAATGGAGGGCCCAGTCTGGATCTGCCCAAAGCAAACTTGTGAGTGGAGTTGGAGGCTTTGTCCTGGGGCTGATCTTCCTCAGTGTAGGACTGATCCTCCACCTGAAGAGCCAGAAAGGTGA

>modo_DNAzoo_DCB_1

ATGGTGTTTTTGGAGTTGCTGGGAGGTCCCTCTGTGACAGTCCTGTTGATGGTGCTGGATACACTCACAGTCTGGAGCACGGACATTCCAGGTAAAGAATTACCTACATCAAGTGAGGTCTGAGTGTCATATGACCAATGGAACCCAGCGGGTACACTTCGTGGGAAGACTCATCTATGACCGGGAAGAGTTTGTGCGCTTTGACAGTGATGTGGGTCTATTTGAGGCAAGAACGGAACTGTGGAAATCCCAGGTCCAGAAATGGAACAGTCAAAAGGAAATAGTTGAACGAGCACGGTCCATAGTGAATGTGTGCAGACACAACTACCAATTCTATAATAAAACTATAGTGCAGAGAAAAGTCAAGCCCCGAGTGAAAGTTTTCCCAGCAAAAACGCAACCACTCGGGCACCACAACTTGCTCCTCTGTTCTGTGACCAGTTTCTATCCTGGTGAGATAAAGGTCAGCTGGttcagaaatgcaaaagaagagaaATCTGGAGTCCTGTCTACAGGCCAAATCCGGAATGGTGACTGGACCTTCCAGACCCTTGTAATGCTGGAAATGACCCCCCAAAGGGGAGATGTCTTTACTTGCCGTGTGGACCATGTCAGCTTGCAGAGCCCTGTCTTCGTGGATTGGAGAGCACAGTCAGAATCTGCCCAGACTAAGATGCTGATTGGAGTTGGGAGCCTGGTGTTTGGAATGATTTTACTTGGAGTGGGACTTGTCATCAGCCTCAGAAGCTCAAAAGATTCCTATTCTGGAACCAAAGAAGACTCGTGCTTAGAAGGAATTGTAAACAGTGACCCACAGCAACAAAATTTTCCTAGAGCTCTTTTCCAATCCTAA

>modo_DNAzoo_DMB_1

ATGAAGTTACTTCTGCCACTATTAATGGGCCTCGGCTTGGGTTTTTCAGGAGCAGCAGAGGGCTTTGTGACCCATGTGGAGAGTAGCTGTGTGCTGGATGAAGATGGATCGGCAAAGGACTTCTCATATTGTATCTCCTTCAACAAGGCTATGCTAACCTGCTGGGACTCAGAGACTAAAATGATGGTTACTGTTAACTTTGGGATATTGCAAGGAATAGCTGAACAGATTACCAATTACACTAACCATCAGAGCAACTTTATATCTCGCCTGAGCAATGGACTTCAAGACTGTGCCAGTCACACAAAGCCTTTCTGGGGGGCATTGACGCAGAGGACACGACTACCATCAGTGCAAATAGCCCAGGTCAAACCATTCAACACAAGGGAGTCAGTGATGCTGGCCTGTTATGTATGGGGCTTCTATCCTGCTGATGTGGCCATTTCATGGTTGAAGAATGGACAGCTTATTCCACAAAGTGGTATTCAGAGAGCTGTACAGTCCAATGGGGATTGGACTTATCAGACAAGATCCTACTTGGCCCTTACCCCCTCTACTGGAGATATTTACTCTTGCCTTGTAGAGCATAGTGGGACTTCTCAGGCCATCTTACAGACCTGGACACCTGGCCTCTCTCCGAAGCAGACATTGAAAATCTCCGTATCTGTATTTACTCTGTGCTTTGGTCTCATCATCTTCTTCCTTGGCCTGGTGTCCTGCCGAAAAGCTGGCTCTTCTGATTACACTGTTCTCTTGGGGTCCAATTATCCTGAAGGTAA

>Phgy_DNAzoo_DAB_1_partial_exon_3-4

TTAAGCCAGAGGTGATTGTGTATCCATCAAAGATGGCTCCCCTAGGACACCACAACCTGCTTGTCTGCTCTGTCCCTGGTTTCTATCCTGGGGACATTGAGGTCAGGTGGTTCCTGAATGGGCAGGAGGAGAGAACTGGGGTTGTGTCCACAGGCCTGATCAGCAATGGAGACTGGACCTACCAGATCTTGGTGATGCTGGAAATGATCCCCAAGCGTGGAGATGTCTACACCTGCCAAGTGGAGCACTCCAGCCTTCACAGACATGCTATGTTTCACTGGAAAGCACAGTCTGAATCTGCCCAGATTAAGATGCTCAGTGGAATTGAAGGCCTTGTGCTGGGGCTGATCTTCTTTGGGGTCGGCCTCATTGTCTACAAGAGAAGTCAGAAAGGTGA

>Phgy_DNAzoo_DAB_2_partial_exon_3-4

TTAAGCCCAAGGTGACTGTGTATCCATCAAAGATGGCTCCCCTGGGACACCACAACCTGCTTTTCTGCTCTGTCATGGGTTTCTATCCTGGGGACATTGAAGTCCAGTGGTTCCTGAATGGTAGGAAGAGATACCTGGGGTGGTGTCCATAGGCCTGATCAGCAGTGGAAACTGGACCTACCAGATCCTGGTGATGCTGGAAATGACCCCCAAGCATGGAGATGTCTACACCTGCCAAGTGGAGCACTCCAGCCTTCAGAGACCTGTCATCTTGGACTGGAAACTGCAGTCTGAATCTGCCCAGAGTAAGGTGCTGAGTGGAGTTGGGGGCCTCGTGCTGGGGCTGATCTACTTTGGGGTTGGCCTCATTGTCCACAGGAGGAGTCAGAAAGGTGA

>Phgy_DNAzoo_DAB_3_partial_exon_3-4

TTGAGCCCGAAGTGATGGTGTATCCATCGAAGATGGCTCCCCTGGGACACCACAACCTGCTTTTCTGCTCTGTCATGGGTTTCTATCCTGGGGACATTGAAGTCCAGTGGTTCCTGAATGGGTAGGAGGAGATAGCTGGGGTTGTGTCCACAGGCCTGATCAGCAATAGAGACTGGACCTACCAGATCCTGGTGATGCTGGAAATGACCCCCAAGGGTGGAGATGTCTACACCTGCCAAGTGGAGCACTCCAGCCTTCAGAGACCTGTCGTCCTGGACTGGAAACTGCAGTCTGAGTCTGCCCAGAGTAAGATGCTGAGTGGAGTCAGGGGCCTCGTGCTGGGCCTGATCTTCTTTGGGGTTGGCCTCATTGTCTACAGGAGGAGTCAGAAAGGTGA

>Phgy_DNAzoo_DAB_4_partial_exon_2-4

AGCATTTCACCGAGCAGGTGAAGGGCGAATGTTCCTTTGTGAACGGGACGAAATACACGCGGTTTGTGCTGAGGGCCATCTACAACCGACAGGAGTACGCGCGCTTCGACAGCGAGGTGGGAGTGTTTGTGGCTACTACAGAGCTGGGGCGGCTCACTGCTCAGTTTGCTAACAGCCAGAAGGAGTTCATGGATCACGTACGGGCTGCGGTGGACACTTATTGCAGGCACAACTACCAGGTGTTTGAGCCCGCTTCAGTGCCCAGGAGCGGTGACTGAGCCTGAGGTGACTGTGTATCCATCAAAGACTGCTCCCCTGGGACACCACAACCTGCTTGTCTGCTCTGTCACTGGTTTCTATCCTGGGGAAATTGAAGTCCGATGGTTCCTGAATGGACAGGAGGAGACAGAGGGGGTTGTGTCCACAGGCCTGATCAGCAATGGAGACTGGACCTACCAGATCCTGGTGATGCTGGAAATGACCCCCAAGCGTGGAGATGTCTACACCTGCCAAGTGGAGCACTCCAGCCTTCAGAGACCTGTCATCTTTGACTGGAAAGTACAGTCTCAATCTGCCCAGAGTAAGATGCTGAGTGGAGTCGGGGGCCTCGTGCTGGGCCTGATCTTCTTTGTGGTTGGCCTCGTTGTGCACAAGAGGAGTCAGAAAGGTGA

>Phgy_DNAzoo_DAB_5_partial_exon_2-4

AGCACTTTATGGAGCAGATGAAGACTGAGTGTCACTTTGTCAATGGGACTGAGCATGTGCGGTTTGAGGGGAGACTCATCTACAACTGCCAAGAGATCCCGTGCTTCGACAGCCACGTGCAGAGGTTTGTGGCCTTAACCAAGCTGGGGTGGTCCATTGCGGGAGGTAATGAACTGCCTGTTGGAGGCCTTGGAGCAAGCACAGGCCCAGGTGGCCAGGTGCAGGAACAACTATAGGTTGTTGGAGTCCTTCTGGATACAGAGGAGGGTTGATGGAGCTGGAAGTGATTGTGTGTCCATCAAAGATAGCACCCCTAGAACACCCAAACCAGCTTGTCTATTTTGTTATTGGTTTCTATTGTGGGGACATTGAGGTCAGGTGGTTCCTGAATGGTCAGGAGGAGACAGCTGGGGTTGTGTCCACAGGCCTGATTAACAATGGAGACTGGACCTACCAGACTCTGGTGATGCTAGAAATGATCCCCAAGAGTGGACATGTCTACACCTGCCAAGTGGAGCACTCCAGCCTTCAGAACCCTGTCATTATGGTCTGGGAAGGACCATCTACATCTGCCCAAGGAAAAATGCTGAGTGGATTTAGGGGTTTTGTGCTAGGGCTGATCTTCCTTGGGGTTTGCCTTACTGTTCTCCCTAGAAGTCAGAAATGTGA

>Phgy_DNAzoo_DAB_6_partial_exon_2-4

CGCACTTCACAGAGTAGGTTGTCAGTGTTACTTTGTGAATGGGACAGAGCACGTGCAGTTTGCGGAGAGATACATCAACAACTGAGAGGAGAGTGTGTGCTTCGACAACGACGTGGGAGAATATGTGCCGGTGACGGAGCCGGGGCGGACCGATGCTGAAAACTGGAACAGCCGGAAGGAGATCCTGGAGCAAAAACGGACCTCTGTGGACTGGTTCTGCAGGGTCTGCTACAAGGTGTCTAAGACAGTCACAGTGATTGAACCTGAGGTGACTGTGTGTCCATCAAAGAAGGCTCCCTTGGGACACCACAACCTGCTTGTCTGCTCTGTCAATGGTTTCTGTTCTGGTGACATTGAGGTCAAGTTGTTCCTGAATGGGCAGGAGGAGACAGAGGGGGTTGTGTTCACAGGCCTGATCAGCAATTGAGACTGGACCTACCAGATCCTGGTGATGCTGGAAATGACCCCCAAGCGTGGAGATGTCTACACCTGCCAAGTGGAGCACTCTAGCCTTCACAGACCTGTTGTCTTTGACTAGAAAGCATAGTCTGAATCTGCCCAGAGTAAGATGCTGAGTGGAATAGGAGGCCTCGTGCTGGGCCTGATTTTCTTTGAGGTTGGCCTTATCATCCACAAGAGGAGTCAGAAAGGTGA

>Phgy_DNAzoo_DAB_7_partial_exon_2-4

AGCACTTTATGGAGCAGATGAAGGCTGAATGTCACTTTGTCAATGGGACTGAGCACGTGCAGTTTGTGGGGAGACTCATCTACAACCGCCAAGAGATCCTGCGCTTTGACAGCCACGTGGGGAAGTTCTTGGCCTTGACTGAGCTGTGGCGGCCCATTGCGGAGCTAATGAACAGCCTGCTGGAAGCCCTGGAGCAAGCACGAGCCCAGGTGGCCAGGTGCAAAGACAACTATAGGTTGCTGAAGTCCTTCTGGATGCTGAGGAAGGGTGATTGAGCCTGAAGTAACTGTGTATCCATCAAAGATGGCTCCCCTAGGATACCCGAACCAGCTTGTCTGTTTTATTACCGGTTTCTATCCTGGGGACATTGAGGTCAAGTGGTTCTTGAATGGTCAGGAGGAGACAGCTGGGGTTGTGTCCACAGGTCTGATTAGCAATGGAGACTGGACCTTCCAGATTCTGGTGATGCTGGAAATGATCCCCAAGCATGGAGATGTCTACACCTGCCAAGTGGAGCACTCCAGCCTTCAGAACCCTGTCATTGTGGTCTGGGAAGCACAGTCCAGATCAGCCCAAGGAAAGATGCTGAGTGGAGTCGGGGGCCTTGTGCTAGGGCTGATCTTCCTTGGGGTTGGCCTTACTGTTCACCTCAGGAGTCAGAAAGGTGA

>Phgy_DNAzoo_DBB_1

ATGGCTAACGCTTGGATCTCTGCCGGCTGTTGGAAGATTGGTCTGTTAATGACCTCGATGGTGTTGAGTCTACCTGCATCTTGGGCCAGGGACATCCCGGAGGATTTCGTGCTTCAGCACAAGTGTGAATGTTACTTCACCAACGGCTCGGAGCGGGTGCGGCATATGTCTAGATACTTCTACAACTACCAGGAATACGTCCGCTACGACAGCGCCGTGGGGGAGTTCGTGGCCGTGACGGAGCTGGGGCGGCCCTCTGCTGAGTACTGGAACAGTCAGAAGGACATCCTGGAGCAAATGCGAGCCGAGGTGGACACGGTGTGCAGACACAACTACGAGGCGACCAAGCCCTACACGGTGAACAGAAGAGTCCAGCCCAGAGTGACCATCTCCCCCTCCCGGAGAGAGGCCCTGCAGCACCTGCTGGTCTGCTCTGTCTCTGGCTTCTACCCAAGCAAGGTCAAGGTCACCTGGCTCAAGAATGGGCGGGAGGAGACGGCCGGGGTTGTGTCCACGGGTGTGATACAGAACGGAGACTGGACCTACCAGACCCTGGTCATGTTGGAAATGACTCCCCAGAGCAGAGATGTCTACACCTGCAGCGTGGAGCACGCCAGCCTACAGAGCCCCATCAGTGTGGAATGGAGGGCACAGTCTGAATCTGCCCGGAGCAAATTGCTCAGTGGAA

>Phgy_DNAzoo_DCB_1

ATGGTGTGTGTGGAGTTTCTGGGAAGCCCCTGTAGGACAGTCCTATTGATAGACAGATTGTGAGCACGCCCACAGCCTGGGGCAGGGAAATTCCAGGTAAAGATTTACGTACATCAGGTGAAGTCCAAGTGTCACGTGGCCAATGGAATCCAACGGGTGCACTTCATGGGAAGACTCACCTACAACCAAGTGGAGTTTGTGTGCTTTGACAGGGATGTGGGACTATTTCAGGCAAGAATGGAGCTGTGGAAATCCCAAGTCCAGAAATGGAACAGTCAGAAGGAGACAGTCAAGCATGCAAGGTCCATAGTGAATGTGTGCAGACACAACTATCTTTTATACGATAAATTCATAGTGCAAAGGAAAGCCCGAGGTGATGGTGTATCCATCGAAGATGGCTCCCCTGGGACACCACAACCTGCTTGTCTGCTCTGTCACTGGTTTCTACCCTGGGGACATTGAGGTCAGGTGGTTCCTGAACGGGCAGGAGGAGACAGCTGGGGTTGTGTCCACAGGCCTGATCAGCAATGGAGACTGGACCTACCAGATCCTGGTGATGCTAGAAATGACCCCCAAGCGTGGAGATGTCTACACCTGCCAAGTGGAGCACTCCAGCCTTCAGAGACCTGTCGTCTTGGACTGGAAAGCACAGTCTGAGTCTGCCCAGAGTAAGATGCTGAGTGGAGTCGGGGGCCTTGTGCTGGGCCTAATCTTCTTTGGGGTTGGCCTCATTGTCCACAGGAGGAGTCAGAAAGGTGA

>Phgy_DNAzoo_DMB_1

ATGAGGTTACTCCACCTGCTACTAGTGGCCTTCAGCCTGGGTTTTCCAGCAGCAGGGGGCTTTGTGACCCATGTGGAGAGTGGCTGTATGCTGGATGAAGAAGGATCAGCAAAAGACTTCACATATTGTATCTCCTTCAACAAGTATGTGTGGACCTGCTGGGACTCGGAGACTAACAAGATGGTCACTGTCAATTTTGGAATACTGCATCAATTGGCTGAGGAGGTTTCTGCTGCCCTCAATAATGACAGTGCTTTGATAGACCGCCTGAGCAAAGGATTCCAGGACTGTGCCAGTCACACAAAGCCCTTCTGGGGCTCATTGACCCAAAGGACGCGGCCACCATCAGTGCAAATAGCTCAGACCACACCATTTAACACAAGGGAGCCCGTGATGCTGGCCTGTTATGTATGGGGCTTCTATCCTGCTGATGTGGCCGTTTCATGGTTGAAGAATGGGCAGCCTATCCCAGACAGTGGCATCCAGAAGGCTGTACAATCCAATGGGGACTGGACTTATCAGACTCGATCCTACTTGGCCCTTACTCCCTCTAGTGGGGATATTTATACTTGCTACGTAGAGCACAGTGGGACTTCCCAGATCATCTTACAGACCTGGACGCCTGGCCTCTCTCTGAAGCAGAGGGTGAAGATCTCTGTATCCGTGCTGACTCTATGCCTTGGTCTCGTCTTCTTCTTCCTTGGCCTGGTTGCCTGCCGAAAAGCTGGCTCCTCTGACTACACTCCTCTCTCGGGGTCCAATTATCCTGAAGGTAA

>phci_DAB_1

ATGCTATGTGTGTGGACAAAAGTTCTGATGATGACCCTGCTAGTGCTGAATTCCCTAGTGGCTGCAGGCAGAGAAACTCCAGAGCACTTTATGGAGCAGATGAAGGCTGAGTGTCACTTTGGCAATGGGATTGAGCACGTGCGGTTTGTGGGGAGACTCATCTACAACCTCCAGGAGATCTTGCGCTTCGACAGTGATGTGGGGGAGTTCGTGGCCTTGACGGAGCTGTGGCGGCCCATTGCAGAGCTAATGAATAGCCTGGTGGAGGCCCTGGAGCAAGCGCGGGCCCAGGTGGCCATGTGCAGGGACAACTATAGGTTGTTGGAGTCCTGGATGCAGAAGAAGGttgAGCCTCAAGTGACTGTGTATCCATCAAAGATTGCTACCCTAGAATACCCAAACCAGCTTATCTGTTTTGTTACTGGTTTCTATCCTGGGGACATTGAGGTAAGGTGGTTCCTGAATGGTCAGGAGGAGACATTTGGGGTTATGTCCACAGGCCTGATTAGCAATGGAGACTGGACCTACCAGATCCTGGTGATGCTGGAAATGATCCCCAAGTATGGACAAGTCTACACCTGCCAAGTGGAGCACTCCAGCCTTCAGAACTCTGTCATTGTGGTCTGGGAAGCACCGTCCAGCTCTGACCAGAGAAAGATGCTGAGTGGACTTGGGGGCCTTGTGCTAGGGCTGATCTTCCTTGGGGTTGGCCTCGCTGTTCACCTTAGGAGTCTGAAAGGTGA

>phci_DAB_2

ATGGTGTGTGTGCTGTTCCCCGGGGGAGTCTGGACAGAGGTTCTGGCTGTGACCCTGCTGGTGCTGAATTCCCAGGTGGCTGCAGGCAGACATGCCCCAAAGCACTTCACGGAGCAGTTAAGTCCGAGTGTTACTTCGAGAACGGGACGGAGCGCGTGCGGTTTGTGGAGAGATACATCCACAACCGGGAGGAGTACGTGCGCTTCGACAGCGACGTGGGGGAGTTTGTGGCGGTGACGGAGCTGGGACGGCCCGATGCTGAGCGTTGGAACAGCCAGAAGGAGATCCTGGAGGACGAACGGGCCCGGTGGACACTTGTGCAGGCACAACTACGGGGTGTCTGAGCCCTTCTTAGTGCGCAGGAGCGttGAGCCTGAGGTGATTGTGTATCCATCAAAGATGGCTCCCCTGGGACACCACAACCTGCTTGTCTGCTCTGTCAGTGGTTTCTATCCTGGGGACATTGAGGTCAGGTGGTTCCTGAATGGGCAGGAGGAGACGGCTGGGGTTGTGTCCACAGGCCTGATCAGCAATGGAGACTGGACCTACCAGCTCCTGGTGATGCTGGAAATGACCCCCAAGCGTGGAGACGTCTACACCTGCCAAGTGGAGCACTCCAGCCTTCAGAAACCTGTTGTCTTGGACTGGAAAGCACAGTCTGAATCTGCCCAGAGTAAGATGCTGAGTGGAGTCGGGGGCCTCGTGCTGGGCCTGATCTTCTTTGGGGTTGGCCTCTTTGTCTACAAGAGGAGTCAGAAAGGTGA

>phci_DAB_3

ATGGCGTGTGTGCTGCTCCCCAGGGGCATCTGGACAGAGGTTCTGGCTGTGACCCTGCTGGTGCTGAATTCCCAGGTGGCTGCAGGCAGACATGCCCCAGAGCACTTCACGGAGCAGATGAAGGCCGAGTGTTACTTCGAGAACGGGACGGAGCGCGTGCGGTTTGTGGAGAGATACATCCACAACCGGGAGGAGTACGTGCGCTTCGACAGCGACGTGGGGGAGTATGTGGCGGTGACGGAGCTGGGACGGCTCAGTGCTGAGCGTTTGAACAGCCAGAAGGAGATCCTGGAGGACGAACGGGCCGCGGTGGACACTTGTGCAGGCACAACTACGGGGTGTCTGAGCCCTTCTTACTGCGCAGGAGCGttGAGCCCGAGGTGATTGTGTATCCATCAAAGATGGCTCCCCTGGGACACCACAACCTGCTTGTCTGCTCTGTCAGTGGTTTCTATCCTGGGGACATTGAGGTCAGGTGGTTCCTGAATGGGCAGGAGGAGACGGCTGGGGTTGTGTCCACAGGCCTGATCAGCAATGGAGACTGGACCTACCAGCTCCTGGTGATGCTGGAAATGACCCCCAAGCGTGGAGACGTCTACACCTGCCAAGTGGAGCACTCCAGCCTTCAGAAACCTGTTGTCTTGGACTGGAAAGCACAGTCTGAATCTGCCCAGAGTAAGATGCTGAGTGGAGTCGGGGGCCTCGTGCTGGGCCTGATCTTCTTTGGGGTTGGCCTCTTTGTCTACAAGAGGAGTCAGAAAGGTGA

>phci_DAB_4

ATGGTGTGTGTGCTGTTCCCCAGGGGAGTCTGGACAGAGGTTCTGGCTGTGACCCTGCTGGTGCTGAATTCCCAGGTGGCTGCAGGCAGACATGCCCCAAAGCACTTCACCGAGCAGGCTAAGTCCGAGTATCACTTTGAGAACAGGAGGGAGCACGTGCGGTTTGTGGATAGATACATCCACAACCGCGAGGAGTTTATGCGCTTCGACAGCTACCTGGGGGAGTATGAGGCGCTGACGGAGCTGGGGCGGCCCAGTGCTGAGTATTATAACAGCCGCAAGGAGATTCTGGAGCAGAGACGAGCGGCAGTGGACTGGTTTTGCAGGGTCTGCTACAAGGTGTCTGAGCTCTTTTTAGTGCACAGGAGCGttgAGCCCGAGGTGATTGTGCATCCATCAAAGATGGCTCCCCTGGGACACCACAATCTGCTTATCTGCTCTGTCAGTGGTTTCTATCCTGGGGACATTGAGGTCAGTCGTTACTGAATGGGCAGGAGGAGACAGCTGGGGTTGTGTCCACAGGCCTGATCAGCAATGGAGACTGGACTTACCAGCTCCTGGTGATGCTGGAAATGACCCCCAAGCATGGAGATGTCTACACCTGCCAAGTGGAGCACTCCAGCCTTCAAAGACCTGTCATGTTGGATTGGAAAGCACAGTCTGAATCCGCCCAGAGTAAGATGCTGAGTGGAGTGGGAGTCCTAGTGCTGGGCCTGATCTGCGTTTGGGGTTGGCCTCATTGTCCACAAGAAGAGTCAGAAAGGTGA

>phci_DAB_5

ATGGTGTGTGTGCTGCTCCCCAGGGGCGTCTGGACAGAGGTTCTGGCTGTGACCCTGCTGGTGCTGAATTCCCAGGTGGCTGCAGGCAGACATGCCCCAAAGTACTTCACGAGGCTGTTAAAGTTCGAGTGTTACTTTGAGAACGGGACGGAGCACGTGCGGTATGTGGAGAGACACATCCACAACCGGGAGGAGCTCATGCGCTTTGACAGCGACGTGGGAGAGTATGTGGTGCTGATGGAGCTGGGGCTGCGCGAAGCTGAGCATAGGAACAGTCAGAGAGAGATCCTGGAGAACGAACGGGCAGTGGTGGACACTTACTGCAGGCCCAACTACGAGGCTGCTGAGATCTTCTTACTGGGCAGGAGCTttGAGCCCGAGGTGATTGTGTATCCATCAAAGATGGCTCCTCTGGGACACCACAACCTGCTTGTCTGCTCTGTCAGTGGTTACTATCCTGGGAACATTGAGGTCAGGTCGTTACTGAATGGGCAGGAGGAGACAGCTGGGGTTGTGTCCACAGGCCTGATCAGCAATGGAGACTGGACTTACCAGATCCTGGTGATGCTGGAAATGACCCCCAAGCGTGGAGATGTCTACACCTGCCAAGTGGAGCACTCCAGCCTTCAAAGACCTGCCATCTTGGACTGGAAAGCACAGTCTGAATCTGCCCAGAATAAAATGCTGAGTAGAGTCGGGGGCCTCCTGCTGGGCCTGATGTTCTTTGGGGTTGGCCTCATTGTCCACAACAGGAGTCAGAAAGGTGA

>phci_DBB_1

ATGGTTGATGTTTGGATCTGTGCTGGCTGTTGGAAGATTGGTCTGTTAATGACATCACTGGTGTTGAGTCTACCTGCATCTTGGGCCAGGGACATCCCAGAGGATTTCGTGTTTCAGCACAAGGCTGAGTGTTACTTCACCAACGGCACGCAGCTGGTGCGGCATGTTTGTAGATATATCTACAATGATGAGGAATACGCTCACTTCGACAGCGTCTTGGGGGAGTTCGTGGCTGTGACAGAGTTGGGGCAGCCAAGCGCTGAGCGTTGGAACAATCAGAAGGACTTCCTGGAGAAAGAACAAGCCGTTGTGGACGCGGTGTGCAGATACAACTACGAGACAGACAAGTCTTTAGAGGTGGACAGAAGAGTCCAGCCCAGAGTGACCATCTCCCCCTCCAAGACAGAGGCCCTGCAGCACCTACTAGTCTGCTCTGTCACTGGCTTCTATCCAAGCAAGATCAAGGTCACCTGGCTCAAGAATGGGCAGGAGGAGACAGCTGGGGTTGTGTCCAGGGGTGTTATACAAAATGGAGACTGGACCTACCAGACCCTGGTCATGTTGGAAATGATTCCTCAGAGCAGAGATGTCTACACCTGCAGTGTGGAGCGTGCCAGCCTTCAGAGCCCCATCAATGTGGAATGGAGGGTACAGTCTGAATCTGCCCAGAACAAATTGTTGAGTGGAATTGGAGGCTTTGTCCTGGGGCTGATCTTCCTAGGTGTAGGACCGATCATCCACCTGAAGAACCAGAAAGGTGA

>phci_DBB_2

ATGGTTGATGTTTGGATCTCTGCTGGCTGCGGGAAGATTGGTCTGTTAATGACATCGATGGTGTTGAGTCTACCTTCTTCTTGGGCCAGGGAGATCCCAGAGGATTTCGTGCTTCAGTTCAAGGCTTACTGTTACTTCACCAACGGCACGCAGCTGGTGCGGCATGTGTGTAGATATATCTACAATGATGAGGAATACGCTCACTTCGACAGCGACGTGGGGGAGTTCGTGGCTGTGACGGAGCTGGGGCGGCCCGATGCTAAGTACTGGAACAGTCAGAAGGAAATCCTGGAGGAACAACGAGCCATTGTGGACACGGTGTGCCGACACAACTACGAGATAGATAAGCCCTTCACGGTGGACAGAAGAGTCCAGCCCAGAGTGACCATCTCCCCCTCCAAGACAGAGGCCCTGCAGCACCTGCTGGTCTGCTCTGTCACTGGCTTCTATCCAAGCAAGATCAAGGTCACCTGGCTCAAGAATGGGCAGGAGGAGACAGCTGGGGTTGTGTCCACGGGTGTGATACAACATGGAGACTGGACCTACCAGATCCTAGTCATGTTGGAAATGATTCCCCAGAGCAGAGACATCTACACCTGCAGTGTGGAGCATGCCAGCCTGCAGAGCCCCATCAGTGTGGAATGGAGAGCACAGTCTGACTCTGCCCAGAGCAAATTGCTGAGTGGAATTGGAGGCTTTGTCCTGGGGCTGATATTGCTAGGTGTAGGACTGTTCATCCACCTCAAGAACCAGAAAGGTAA

>phci_DBB_3

ATGGTTGATGTTTGGATCTCTGCTGGCTGCGGGAAGATTGGTCTGTTAATGACATCGATGGTGTTGAGTCTACCTGCTTCTTGGGCCAGGGACATCCCAGAGGATTTCGTGTATCAGTTCAAGGGAGAGTGTTACTTCACCAACAGCACGGAGCGGGTGCGGCTTGTGGCCAGACGCTTCTACAATGACCAGGAAATTGTCCGCTTCGACAGCGACGTGGGGGAGTTCGTGGCTCTGACGGAGCTGGGGCGGCCCGATGCTAAGTACTGGAACAGTCAGGAGGAAATCCTGGAGGAATATCGAGCTTACGTGGACACGGTGTGCAGACACAACTACGAGATAGAGAAGCCCTTCGCGGTGGACAGAAGAGTCCAGCCCAGAGTGACCATCTCCCCCTCCAAGACAGAGGCCCTGCAGCACCTGCTGGTGTGCTCTGTCACTGGCTTCTATCCAAGCAAGATCAAGGTCACCTGGCTCAAGAATGGGCAGGAGGAGACAGCTGGGGTTGTGTCCACGGGTGTGATACAACATGGAGACTGGACCTACCAGATCCTAGTCATGTTGGAAATGATTCCCCAGAGCAGAGACATCTACACCTGCAGTGTGGAGCATGCCAGCCTGCAGAGCCCCATCAGTGTGGAATGGAGAGCACAGTCTGACTCTGCCCAGAGCAAATTGCTGAGTGGAATTGGAGGCTTTGTCCTGGGGCTGATCTTCCTAGGTGTAGGACTTTTCATCCACCTCAAGAACCAGAAAGGTAA

>phci_DCB_1

ATGGTATGTGTGGAGCTTCTGGGAAGCCTCTGTATGACAGTCTTATTAATGGTGCTGAGCAGGCCCACAGCCTGGGGCAGGGACATTCCAGGTAAAGAATTACCTACATCAGGTGAGGTCTGAGTGTCACATGACCAATGGAACCCAACGGGTCCGCTTTGTGGGAAGACTCATCTATGACCGGAAGGAGTTTGTGCACTTTGACAGTGACGTGGGACTATTTGAGGCAAAAATGGAGTTGTGGAGATCCCAAGTCCAGAAATGGAACAGTCAGAAGGAAATAGTCAAGACTGCAAGGTCCATAGTGAATGTGTGCAGACACAATTACCTTTTATATGATAAACTCATAGTGCAAAGGAAAGccccgaGTGAAGGTTTTCCCCTCAAAGATACAACCACTTGGGCACCACAACCTGCTCCTCTGTTCCGTGACCAGTTTCTATCCTGGTGAGATCAAGGTCAGCTGGTTCAGGaatgcaaaagaagaaaagtctGGAGTCCTGTCCACAGGCCAAATCCAGAATGGTGACTGGACCTTCCAGACCCTTGTGATGCTAGAAGTGACCCCCCAAAGAGGAGATGTCTTTACTTGCCATGTGGACCATGTCAGCTTGCAGAGCCCTATCACCGTAGACTGGAGAGCACAGTCTGAATCTGCCCGGACTAAGATGCTGACTGGAATTGGGGGCTTGGTGCTTGGACTGATCTTACTTGGAGTGGGACTTGTCATCCACCTTAGAAGTTTGATTCCTATTCTGCGACCAAAGAAGACTCAAATTTGGAAGGGATTGTGAACATTGCCCCATTGCAACAAGATTTTCCCAGAGATGTTGCCCAGTCCTAG

>phci_DMB_1

ATGAGGTTACTCCACCTACTACTAGTGGGCTTCAGCCTGGGTTTTTCAGGAGCAGCAGGGGGCTTTGTGACCCATGTGGAGAGTGGCTGTATGCTGGATGAAGAAGGATCAGTAAAGGACTTCACATATTGTATCTCCTTCAACAAGGATGTGCTGACCTGCTGGGACTCAGAGGCTAACAAGATGGCTGCTGTTGATTTTGGGATCCTGAATCCATTAGCTAAAAAGCTTTCTGAAATCCTCAGTAACGATAGTGCTTTGATGGACCGTCTGAGCAAAGGACTCCAGGACTGCGCCATTCACACAAAGCCCTTCTGGGGAGCGTTGACCCAAAGGACACGGCCACCATCAGTGCAAATAGCCCAGACCACACCTTTCAACACAAGGGAGTCAGTGATGCTGGCCTGTTATGTATGGGGCTTCTATCCTGCTGATGTggccattttatggttgaagaatGGGCAGCCTATCCCAAACAGTGGCATCCAGAAGGCTGTACAATCCAATGGGGACTGGACTTATCAGACACGATCCTACTTACCCCTTACCCCCTCTAGTGGGGATATTTACACTTGCCATGTAGAGCACAGTGGAACTTCCCAGCCTATCTTGCAAACCTGGACACCTGGCCTCTCTCTGAAGCAGACAGTGAAGATCTCTGTGTCTGTATTGACTCTGAGCCTTGGACTCACCTTCTTCTTCCTTGGCCTGGTTGCCTGCCGAAAAGCTGGCCCCTCTGacTACACTCCTCTCTCGGGGTCCAATTATCCTGAAGGTAA

>Gyle_DNAzoo_DAB_1

ATGGAATGTGCGTTGCTCCCCATGGGCATCTGGACAGAGGTTCTGGCTGTGATCTTGCTGGTGCTGACTTCCCAAGGGACTGCAGGCAGACATGCCCCAGagcACTTCACGGAGCAGTTCAAGGGCGAGTGTTACTTTGTGAATGGGACGCAGCACGTGCGTCACCTGGAGAGATACATCTACAACCGGGAGGAGTACGCGCGCTTCGACAGCGACGTCGGGGAGTACGAGGCGGTGACGGAGCTGGGGCGGCGCAGTGCTGAGTACTGGAACAGCCAGAAGGAGCTCCTGGAGCAGAAACGGGCAGAGGTGGACACTTTCTGCAGGCCCTGGTACCAAGAGTCCTTAGTGCGCAGGCGCGGTgaTTGAGCCTGAGGTGATTGTGTATCCATCAAAGATGGCTCCCCTGGGACACCACAACCTGCTTGTCTGCTCTGTCACTGGTTTCTACCCTGGGGACATTGAGGTCAGGTGGTTCCTGAATGGGCAGGAGCAGACAGCTGGGGTTGTGTCTACAGGCCTGATCAGCAATGGAGACTGGACCTACCAGATCCTGGTGATGCTGGAAATGACCCCCAAGCATGGAGATGTCTACACCTGCCAAGTGGAGCACGCCAGCCTTCAGAAACCTGCTACCTTTTACTGGAAAGCACAGTCTGAATCTGCCCAGAGTAAGATGCTGAGTGGAGTCGGGGGCCTGGTGCTGGGGCTGATCTTCTTTGGGGTTGGCCTCATTGTCCACAAGAGGAGCCAGAAAGGTGA

>Gyle_DNAzoo_DAB_2

ATGGTGCGTGTAATGCTCCCCCAGGGCATCTGGACAGAGGCTCTGGCTGTGACCCTGCTGGTGCTGACTTCCCAGGTGGCTGCAGGCAGACATGTCCCAGagCACTTCATGGAGCAGGTGAGGTCCGAGTGTCACCATGAGAACGGGACGCAGCACGTGCGGTATGTGCAGAGATACATCTACCACCGGGAGGAGTACGTGCGCTTCGACAGCGACGTTGGGGTGTTTGAGGCGGTGACAGAACTGGGGCGGCGCAGTGCTGAGTACTGGAACAGCCAGAAGGAGATCCTGGAGCAGAAACGGGCCGCGGTGGACACTTTCTGCAGGCACAACTACCAGGGGTCTGAGCCCTTCTTAGTGCGCAGGCGCGGTGAtTGAGCCTGAGGTGATTGTGTATCCATCAAAGATGGCCCCCCTGGGACACCACAACCTGCTTGTCTGCTCTGTCACTGGTTTCTATCCTGGGGACATTGAGGTCAAGTGGTTCCTGAATGGGCAGGAGGAGACAGCTGGGGTTGTGTCCACAGGCCTGATCAGCAATGGAGACTGGACCTACCAGATCCTGGTGATGTTGGAAATGACCCCCAAGCATGGAGATGTCTATACCTGCCAAGTGGAGCACTCCAGCCTTCAGAAACCTGTCATCTTGGACTGGAAAGCACAGTCTGAATCTGCCCAGAGTAAGATGCTGAGTGGAGTCGGGGGCCTGGTGCTGGGGCTGATCTTCTTTGGGGTTGGCCTCATTGTCCACAAGAGGAGTCAGAAAGGTGA

>Gyle_DNAzoo_DAB_3

ATGGTGTGTATCTTGCTCCTCCAGGGCATCTGGACAGAGATTCTGGCTGTGACCCTGCTGGTGCTGACTTCCCAAGTGACTGCAGGCAGACATGCCCCAGAGCACTTCAAGGAGCAGATGAAGTCCGAGTGTTACTTTGAGAACGGGACGCAGCACGTGCGGTTTGTGAAGAGACACATCACCAACGGGGTGGAGACTGTGCCCTTCGACAGCGCCGTGGGTGTGTTTGAGGCGCTGACGGAGCTGGGGCGGGAGGATGCTGAGCATTGGAACAGCCAGAAGGATTACCTGGAGCAGAAACAGGGCCAGGTGGACAATTACTGCAGGCAAAACTACCAGGTGTTGAAGCCCTTAGTGCGCAGGCGCGGTGACCAGAGGTGATTGTGTATCCATCAAAGATGGCCCCCCTGGGACACCACAACCTGCTTGTCTGCTCTGTCACTGGTTTCTACCCTGGGGACATTGAGGTCAGGTGGTTCCTGAATGGGCAGGAGCAGACAGCTGGGGTTGTGTCCACAGGCCTGATCAGCAATGGAGACTGGACCTACCAGATCCTGGTGATGCTGGAAATGACCCCCAAGCGTGGAGATGTCTACACCTGCCAAGTGAAGCACTCCAGCCTTCAGAAACCTGTCATCTTGGACTGGAAAGCACAGTCTGAATCTGCCCAGAGTAAGATGCTGAGTGGAGTCGGGGGCCTGGTGCTGGGGCTGATCTTCTTTGGAGTTGGCCTCATTGTCCACAAGAGGAGTCAGAAAGGTGA

>Gyle_DNAzoo_DAB_4

ATGGTGTGTGTCTTGCTCCCCAAGGGTGTCTGGATAGAAGTTCTGGTTGTGACTTTGCTGGTGCTGAATTCTCAGGTGGCTGAAGGGAGACATGCCCCTGAGCACTTCACCGAGCAGAGAAAGGCCGAGTGTCACTTTGTGAATGGGACAGAGCACATACGGTATGTGCTGAGATGCATCCACAACAGGGAAGAGATTGTGCGCTTCGACAGCGACGTGGGGGAGTTTGTGGCGCTGACCGAGCTGGGGCGGCCCGAGGCTGAGTATTGGAACAGCCAGAAGGAGATCGTGGAGTACAGACGCGACCTGGTGAACACTTACTGCCGGCCCAACTACCGGAGGATTGAGTACTTTTTAAAGcgcaggagaggtgacTCAGCCAGAGGTGATTGTGTATCCATCAAAGATGGCTCCCCTGGGACACCACAACCTGCTTGTTTGCTCTGTCACTGGTTTCTACCCTGGGGACATTGAGGTCAGGTGGTTCCTGAATGGGCAGGAGGAGACAGCTGGGGTTGTGTCCACAGGCCTGATCAGCAATGGAGACTGGACCTACCAGACCCTGGTGATGTTGGAAATGACCCCCAAGCATGGAGATGTCTACACCTGCCAAGTGGAGCACTCCAGCCTTCAGAAACCTGTCATCTTGGATTGGAAAGCACAGTCTGAATGTGCCCAGAGTAAGATGCTGAGTGGAGTCGCTGTCCTCACCTTGGGCCTGATCTTCTTTGGGGTTGGCCTTATTGTCCACAAGAGGAGTCAGAAAGGTGA

>Gyle_DNAzoo_DAB_5

ATGGTATGTCTCTTGCTCCCCACAGGCTTCTGGATACAAATTCTGGCTGTGACCCTGTTGTTACTGAATTCTCAGATGATTGCAGGCAGACACATGGCAGGGCACTTCATGGGGCAGGCAAAGGCCGAGTGTCACTTTGTGAACGGGACGGAGAACGTGCGGTTTGTGGTGAGATTCATCTACAATCGTGAGGAGTACGCGCGCTTTGACAGCGccgtggggttttttttggcggTGACAGAACTGGGGCGGCCCGATGCTGAGCTTTGGAACAGCCAGAAGGAGCTCCTGGAGAACACACGGGCCTCGGTGGACACTTTGTGCAGGCATAACTACGAGTTGTCTGAGCTTGTCTTACGGCGCTGGAAAGtTGAGCCCAAGGTGATTGTGTATCCATCAAAGATGACTCCCCTGGGACACCACAACCTGCTTGTCTGCTCTGTCACTGGTTTCTATCCTGGGGACATTGAGGTCAGGTGGTTCCTGAATGGGCAGGAGGAGACAGCTGGGGTTGTGTCCACAGGCCTGATCAGCAATGGAGACTGGACCTACCAGACCCTGGTGATGTTGGAAATGACCCCCAAGCATGGAGATATCTACACCTGCCAAGTGGAGCACTCCAGCCTTCAGAAACCTGTCATCTTGGATTGGAAAGCACAGTCTGAATCTGCCCAGAGTAAGATGCTGAGTGGAGTTGGGGGCTTCATGCTGGGCCTGATCTTCTTTGGGGTTGGCCTCATTGTCCACAAGAGGAGTTAGAAAGGTGA

>Gyle_DNAzoo_DAB_6

ATGGTGTGGGTCTTGCTCCCCCCGGGTGTCTGTATAGAGGCTCTGGTTGTGGCTCTGCTGGTGCTGAATTCCCAGGTGGCTGCAGGCAGACACACTCCAGACCACTTCATGGAGCAGGTGAAGGGTGAATGTTACTTTGTGAACGGGACGCAGCACGTGCGGTTTGTGCTGAGGGCCATCTACAACCGGGAGGAGTACGCGCGCTTCGACAGCGCCGTGGGGGAGTTCGTGGCGGTGACGGAGCTGGGGCGGCCCACTGCTGAGAAAGTGAAAAGACGGAAGGATTACCTGAAGCGCCTACGGGCCGCGGCGGACAATTGCAGGCAAAACTACGAGGTGTTGGAGTCCTTCTTAGTGCAAAGGCGCGGTGAttGAGCCTGAGGTGATTGTGTATCCATCAAAGATGGCCCCCCTGGGACACCACAACCTGCTTGTCTGCTCTGTCACTGGTTTCTACCCTGGGGACATTGAGGTCAGGTGGTTCCTGAATGGGCAGGAGGAGACAGCTGGGGTTGTGTCCACAGGACTGATCAGCAATGGAGACTGGACCTACCAGATCCTGGTGATGCTGGAAATGACCCCCAAGCATGGAGATGTCTACACCTGCCAAGTGGAGCACTCCAGCCTTCAGAAACCTGTCATCTTTGACTGGAAAGCACAGTCTGAATCTGCCCAGAATAAGATGCTGAGTGGAGTCGGGATCCTTGTGCTGGGGCTGATCTTCTTTGGGGTTGGCCTCATTGTCCACAAGAGGAGTCAGAAAGGTGA

>Gyle_DNAzoo_DAB_7

ATGCTGTGTGTGTGGACAAAAGTTCTGATGATGACCCTGCTGGTGCTGAATTCCCTCCTGGCTGAAGGCAGAGACAGCCCAGAGCACTTTATGGAGCAGACGAAGGCTGAGTGTCACTTTATCAATGGGACTGAGCATGTGCGATTTGTGGGGAGACTCATCTACAACAGACAGGAGATTCTGCGCTTTGACAGCGACGTGGGGGAGTTTGTGGCCTTGACTGAGCTGGGGCGGCCCATCGCGGAGCTAATGAACAGTGTGCTGGAGGCCCTGGAGCAAGCACGGGCCCAGGTGGCCTGGTGCAGACACAGCTATAGGTTGTTGGAGTCCTGGATGAAGAGGAGGGGTGAttgAACCTGAAGTAACTGTGTATCCATCAAAGATGGCTCCCCTAGGATACCCAAACCTGCTTGTCTGTTTTGTTACTGGTTTCTATCCCGGGGACATTGAAGTCAAGTGGTTCTTGAATGATCAGGAGGAGACAGCTGGGGTTGTGTCAACAGGCCTGATCAGCAATGGAGACTGGACCTACCAGATCCTGGTGATGCTGGAAATGACCCCTAAGCATGGAGATATCTACACCTGCCAAGTGGAGCATGCCAGCCTTCAGAAACCTGtcattgtggtctgggAAGCACAGTCCACATCTGCCCAAGGAAAGATGCTGAGTGGAGTTGGAAGCCTTGTGCTAGGGCTGATCTTCCTGGGGATTGGCCTTGCTGTTCACCTTAGGAGTCAGAGAGGTGA

>Gyle_DNAzoo_DAB_8

ATGCTATGTGTCTTGCTCCCCAAGGACATCTGGATAGAGGTTCTGGCTGTGACCCTGCTGGTACTAACTTCCCAGGTGGCTACAGGCAGACATGCCTCAGAGCACTTAGTGCAGGCTAAGTCCGAGTGTTACTTTGAGAACGGGACACAGCACGTGCGATTTGTGGAGAGACACATCTACAACCGGGAGGAGTACGTGCGCTTCGACAGCGCCGTGGGGGAGTACGTGGCGGTGACCGAGCTGGGGCGGCGCAGTGCTGAGCTTTGGAACAGTCAGAAGGAGCTCCTGGAGCAGAAACGGGCTCGAGTGGACACTTACTGCAGGCACAACTACCAGGAGATTGAGCCCTTAGTGCGCAGGAGCGtTGAGCCAAAGGTGATTGTGTATCCATCAAAGATGGCCCCCCTGGGACACCAcaatctgcttgtctgctctgtCACTGGTTTCTATCCTGAGGACATTGAGGTCAGGTGGTTCCTGAATGGGCAGGAGCAGACAGCTGGGGTTGTGTCCACAGGCCTGATCAGCAATGGAGACTGGACCTACCAGATCCTGGTGATGTTGGAAATGACCCCCAAGCGTGGAGATGTCTACACCTGCCAAGTGGAGCACTCCAGCCTTCAGAAACCTGTCATCTTGGATTGGAAAGCACAGTCTGAATCTGCCTGGAGTAAGATGCTGAGTGAAATCAGAAAGCTTTTATTGGGGCTGATCTTCTTTGGGGTTGGCCTCATTGTCCACATGAGGAGTAAGAAAGGTGA

>Gyle_DNAzoo_DBB_1

ATGGTTGATGTTTGGATCTCTGCTGGCACCTGGAAGATTGATCTGTTAATTACACTAACAGTGTTGAGTCTACCTGTATCTTGGACCAGGGACAGCCCAGAGGATTTCGTGTATCAGCACAAGGGGGAGTGTTACTTCACCAACGGCTCGGAGCGGGTGCGGTTTGTGGCTAGATACATCTACAATGACCAGGAGGATGTCCGCTTCGACAGCGACGTGGGGGTGTTCGTGGCTGTGACGGAGCTGGGGCGGCCCGATGCTGAGTCCTTCAACAGTCAGAAGGAGATCCTGGAGGAACATCGAGCCTACGTGGACGCGCTGTGCAGACACAACTACGAGATAGACAAGCGCTTCACGTTGGACAGAAGAGtCCAGCCCAGAGTGACCATCTCCCCCTCCAAGACAGAGGCCCTGCAGCACCTGCTGGTCTGCTCTGTCACTGGCTTCTATCCAAGCAAGATCAAGGTCACCTGGCTCAAGAATGGGCAGGAGGAGACAGCTGGGGTTGTGTCCACGGGTGTGATACAGCATGGAGACTGGACCTACCAGACGCTGGTCATGTTGGAAATGACTCCCCAGAGCAGAGATGTCTACACCTGCAGTGTGGAGCATGCCAGCCTACAGAGCCCCATCAGTGTGGAATGGAGGGCACAGTCTGAATCTGCCCAGAGCAAATTGCTGAGTGGAATTGGAGGCTTTGTCCTGGGGCTGATCTTCCTAGGTGTAGGCCTGATCATCCACCTGAAGAACCAGAAAGGTGA

>Gyle_DNAzoo_DBB_2

ATGGTTGATGTTTGGATCTCTGCTGGCAGCTGGAAGATTGATCTGTTAATGACACTGACAGTGTTGAGTCTACCTGCATCTTGGACCAGGGACATCCCAGAGGATTTCGTGTATCAGTACAAGGCGGAGTGTTACTTCACCAACGGCTCGGAGCGGGTGCGGTTTTTGTATAGAGACATCTACAATGGCCAGGAGGATGTCCGCTTCGACAGCGACGTGGGGGTGTTCGTGGCTGTGACGGAGCTGGGGCGGCCCGATGCTGAGTACTGGAACAGTCAGAAGGAGATCCTGGAGGAAAAACGAGCCACCGTGGACACGGTGTGCAGACACAACTACGAGGTATACAAGCCCTTCACGTTGGACAGAAGAGtCCAGCCCAGAGTGACCATCTCCCCCTCCAGGACAGAGGCCCTGCAGCACCTGCTGGTCTGCTCTGTCACTGGCTTCTATCCAAGCAAGATCAAGGTCACCTGGCTCAAGAATGGGCAGGAGGAGACAGCTGGGGTTGTGTCCACGGGTGTGATACAGCATGGAGACTGGACCTACCAGACGCTGGTCATGTTGGAAATGACTCCCCAGAGCAGAGATGTCTACACCTGCAGTGTGGAGCATGCCAGCCTACAGAGCCCCATCAGTGTGGAATGGAGGGCACAGTCTGAATCTGCCCAGAGCAAATTGCTGAGTGGAGTTGGAGGCTTTGTCCTGGGGCTGATCTTCCTAGGTGTAGGCCTGATCATCCACCTGAAGAACCAGAAAGGTG

>Gyle_DNAzoo_DBB_3

ATGGTTGATGTTTGGGTCTCTGCTGACAGCTGGAAGATTGATCTGTTAATGACTTTGATGGTGTTGAGGCTACCTGCATCTTGGGCCAGGGACGTCTCACATAAATCTACAATGGCCAGGAATTTGCTCGCTTCCACAGTGACATCGGGGAGTTCGTGGCTGTGATGGAGCTGGGCCGGCCAAGAGCTAAGTACTAGAACAGTCAGAAGGAGACCCTGGAGAACGCACGAGCCACCGTGGACGCGCTGTGCAGATACAACTAGAGATTGACAAGCGCTTAGCGGTGGACAGAAGAGtcCAGCCCAGAGTGACCATCTCCCCCTCCAAGACAGAGGCCCTGCAGCACCTGCTGGTCTGCTCTGTCACTGGCTTCTATCCAAGCAAGATCAAGGTCTCCTGGCTCAAGAATGGGCAGGAGGAGACAGCTGGGGTTGTGTCCACGGGTGTGATACAGCATGGAGACTGGACCTACCAGATGCTGGTCATGTTGGAAATGACTCCCCAGAGCAGAGATGTCTACACCTGCAGTGTGGAGCATGCCAGCCTACAGAGCCCCATCAGTGTGGAATGGAGGGCATAGTCTGAATCTGCCCAGAGCAAATTGCTGAGTGGAATTGGAGGCTTTGTCCTGGGGCTGATCTTCCTCAGTGTAGGCCTGATCATCCACCTGAAGAACCAGAAAGGTGA

>Gyle_DNAzoo_DMB_1

ATGAGGTTACTCCACCTGTTACTAGTGGGCTTCAGTCTGGGCTTTTCAGGAGCAGGGGGCTTTGTGACCCATGTGGAGAGTGGCTGTATGCTGGATGAAGAAGGATCAGTAAAGGACTTCACATATTGTATCTCCTTCAACAAGGCTGTGTTGACCTGCTGGAACTCAGAGACTAACATGATGACCACTGTTGATTTTGGAATACTATATCCATTAGCTGAGGAGATTTCTGCAAGCCTCAATAATGACAGTGCTTTTATAGACCACCTGCGCAAAGGACTGCAGGACTGTGCTAGTCACACAAAGCCCTTCTGGGGATCACTGACCCAAAGGACACgGCCACCATCAGTGCAAGTAGCCCAGACCACACCATTTAACACAAGGGAGCCAGTGATGCTGGCCTGTTATGTATGGGGCTTCTATCCTGCTGATGTGGCCATTTCATGGTTGAAGAATGGCCAGCCTATCCCACACAGTGGCATCCAGAGGGCTGTACAATCCAATGGAGACTGGACTTATCAGACACGATCCTACTTGGCCCTTACCCCCTCTAGTGGGGATACTTACACTTGTCATGTAGAGCACAGTGGGACTTCCCAGATCATCTTACAGACCTGGACACCTGGCCTCCCTCTGAAGCAGATAGTGAAGATCTCTGTATCTGTATTGACTCTGAGCCTTGgcatcatcttcttcctccttggCCTGGTTGCCTGCCGAAATGCTGGCTCCTCAGaCTACACTCCTCTCTCGGGGTCCAATTATCCTGAAGGTAA

>Pegr_tran_DAB_1

ATGGTGCGTGTAATGCTCCTCCAGTACATCTGGACAGAGGTTCTGCCTGTGACCCTGCTGGTGCTGACTTCCCAAGGGACTGAAGGCAGACATGCCCCAGAGCACTTCATTGAGTATTCTACCTCGGAGTGTCACTTTGAGAACGGGACGCAGCACGTGCGGTTTATGGATAGATACTTCTACAACCGGGAGGAGTTAGTGCGCTTCGACAGCGACGTGGGTGTGTTTGTGGCGGTGACGGAGCTGGGGCGGCCGGATGCTGAGTACTGGAACAGCCAGAAGGAGTACCTGGAGCGTGGACGGACCGCGGTGGACACTGTCTGCAGGCACAACTACGAGCTGTCTGAGCCCTTCTTAGTGCACAAGCGCGTTGAGCCCGAGGTGATTGTGTATCCATCAAAGATGGCTCCCCTGGGACACCACAACCTGCTTGTCTGCTCTGTCACCGGTTTCTATCCTGGCGACATTGAGGTCAAGTGGTTCCTGAATGGGCAGGAGGAGACGGCTGGGGTTGTGTCCACAGGCCTGATCAGCAATGGAGACTGGACCTACCAGATCCTGGTGATGCTGGAAATGACCCCCAGGCGTGGAGATGTCTACACCTGCCAAGTGGAGCATGCCAGCCTTCAGAAACCTGTCATCTTGGACTGGAGTGA

>Pegr_tran_DAB_2

ATGGTGCATGTGTTGCTCCCCACAGGCATCTGGACAGAGGTTCTGCCTGTGACCCTGCTGCTGCTGACTTCCCAGGTGGCTGCAGACAGACATGTCCCAGAGCACTTCACGGTGCAGGCTAAGTGCGAGTGTCACTTTGAGAACGGGACGCAGCACGTGCGGTTTATGGATAGATACTTCTACAACCGGGAGGAGTTAGTGCGCTTCGACAGCGACGTGGGTGTGTTTGTGGCGGTGACGGAGCTGGGGCGGCCGGATGCTGAGTACTGGAACAGCCAGAAGGAGTACCTGGAGCGTGGACGGACCGCGGTGGACACTGTCTGCAGGCACAACTACGAGCTGTCTGAGCCCTTCTTAGTGCACAAGCGCGTTGAGCCCGAGGTGATTGTGTATCCATCAAAGATGGCTCCCCTGGGACACCACAACCTGCTTGTCTGCTCTGTCACCGGTTTCTATCCTGGCGACATTGAGGTCAAGTGGTTCCTGAATGGGCAGGAGGAGACGGCTGGGGTTGTGTCCACAGGCCTGATCAGCAATGGAGACTGGACCTACCAGATCCTGGTGATGCTGGAAATGACCCCCAGGCGTGGAGATGTCTACACCTGCCAAGTGGAGCATGCCAGCCTTCAGAAACCTGTCATCTTGGACTGGAGTGA

>Pegr_tran_DBB_1

ATGGTTGATGTTTGGATCTCTGCTGGTAGCTGGAAGATGGATCTGTTAATGACATTGATGGTGTTGCGTCTACCTGCATATTGGACCAGGGACATCCCAGAGGATTTCGTGTTTCAGCACAAGTGTGACTGTTACTTCACCAACGGCACGGAGCGGATGCGGTTTGTGGCTAGATACATCTACAATGACCAGGAATATGCCCGCTTCGACAGTGACGTGGGGGAGTTCGTGGCCGTGATGGAGCTGGGGCGGCCCGCTGCTGAGTGCTGGAACAGTCAGAAGGAGATCCTGGAGGAACATCGAGCCTACGTGGACACGCTGTGCAGACACAACTACAAGGCTTACATGCCCTTCACGGTGGACAGAAGAGTCCAGCCCAGAGTGACCATCTCCCCCTCCAAGACAGAGGCCCTGCAGCACCTGCTGGTCTGCTCTGTCACTGGCTTCTATCCAAGCAAGATCAAGGTCACCTGGTTCAAGAATGGGCAGGAGGAGACAGCTGGGGTTGTGTCCACCGGTGTGATACAGCATGGAGACTTGACCTACCAGACCCTGGTCATGTTGGAAATGACTCCCCAGAGCAGAGATGTCTACACCTGCAGCGTGGAGCATGCCAGCCTACAGAGCCCCATCAGTGTGGAATGGATTTTAG

>Pegr_tran_DBB_2

ATGGTTGATGTTTGGATCTCTGCTGGTAGCTGGAAGATGGATCTGTTAATGACATTGATGGTGTTGCGTCTACCTGCATATTGGACCAGGGACATCCCAGAGGATTTCGTGTTTCAGCACAAGTGTGACTGTTACTTCACCAACGGCACGGAGCGGATGCGGTTTGTGGCTAGATACATCTACAATGACCAGGAATATGCCCGCTTCGACAGTGACGTGGGGGAGTTCGTGGCCGTGATGGAGCTGGGGCGGCCCGCTGCTGAGTGCTGGAACAGTCAGAAGGAGATCCTGGAGGAACATCGAGCCTACGTGGACACGCTGTGCAGACACAACTACAAGGCTTACATGCCCTTCACGGTGGACAGAAGAGTCCAGCCCAGAGTGACCATCTCCCCCTCCAAGACAGAGGCCCTGCAGCACCTGCTGGTCTGCTCTGTCACTGGCTTCTATCCAAGCAAGATCAAGGTCACCTGGTTCAAGAATGGGCAGGAGGAGACAGCTGGGGTTGTGTCCACCGGTGTGATACAGCATGGAGACTTGACCTACCAGACCCTGGTCATGTTGGAAATGACTCCCCAGAGCAGAGATGTCTACACCTGCAGCGTGGAGCATGCCAGCCTACAGAGCCCCATCAGTGTGGAATGGATTTTAG

>Pegr_tran_DMB_1

ATGAGGTTACTCCACCTGTTACTAGTGGGCTTCAGTCTGGGTTTTTCAGGAGCAGGGGGCTTTGTGACCCATGTGGAGAGTGGCTGTATGCTGGATGAAGAAGGATCAGTAAAGGACTTCACATATTGTATCTCCTTCAACAAGGCTGTATTGACCTGCTGGAACTCAGAGACTAACAAGATGACCACTGTTGATTTTGGAATACTATATCCATTAGCTGAGGAGATTTCTGCAAGCCTCAATAATGATAGTGATTTTATAGACCACCTGAAAAAAGGACTCCAGGACTGTTTTAGTCACACAAAGCCCTTCTGGGGATCACTGACCCAAAGGACACGGCCACCATCAGTGCTAGTAGCCCAGACCACACCATTTAACACAAGGGAACCAGTGATGTTGGCCTGTTATGTATGGGGCTTCTATCCTGCTGATGTGGCCATTTCATGGTTGAAGAATGGGCAGCCTATCCCACACAGTGGCATCCAGAGGGCTGTACAATCCAATGGGGACTGGACTTATCAGACACGATCCTACTTGGCCCTTACCCCCTCTAGTGGGGATATTTACACTTGTCATGTAGAGCACAGTGAGACTTCCCAGATCATCTTACAGTCCTGGACACCTGGTCTCCCTCTGAAGCAGACAGTGAAAATCTCTGTATCTGTATTTACTCTGAGCCTTGGCCTCATCTTCTTCCTCCTTGGCCTGGTTGCCTGCCGAAAAGCTGGCTCCTCTGACTACACTCCTCTCTCTGGGTCCAATTATCCTGAAGGTAG

>Pegr_tran_DMB_2

ATGAGGTTACTCCACCTGTTACTAGTGGGCTTCAGTCTGGGTTTTTCAGGAGCAGGGGGCTTTGTGACCCATGTGGAGAGTGGCTGTATGCTGGATGAAGAAGGATCAGTAAAGGACTTCACATATTGTATCTCCTTCAACAAGGCTGTATTGACCTGCTGGAACTCAGAGACTAACAAGATGACCACTGTTGATTTTGGAATACTATATCCATTAGCTGAGGAGATTTCTGCAAGCCTCAATAATGATAGTGATTTTATAGACCACCTGAAAAAAGGACTCCAGGACTGTTTTAGTCACACAAAGCCCTTCTGGGGATCACTGACCCAAAGGACACGGCCACCATCAGTGCTAGTAGCCCAGACCACACCATTTAACACAAGGGAACCAGTGATGTTGGCCTGTTATGTATGGGGCTTCTATCCTGCTGATGTGGCCATTTCATGGTTGAAGAATGGGCAGCCTATCCCACACAGTGGCATCCAGAGGGCTGTACAATCCAATGGGGACTGGACTTATCAGACACGATCCTACTTGGCCCTTACCCCCTCTAGTGGGGATATTTACACTTGTCATGTAGAGCACAGTGAGACTTCCCAGATCATCTTACAGTCCTGGACACCTGGTCTCCCTCTGAAGCAGACAGTGAAAATCTCTGTATCTGTATTTACTCTGAGCCTTGGCCTCATCTTCTTCCTCCTTGGCCTGGTTGCCTGCCGAAAAGCTGGCTCCTCTGACTACACTCCTCTCTCTGGGTCCAATTATCCTGAAGGTAA

>Lahi_DAB_1

ATGCTATGTGTCTTACTCCCCCAAGGTATCTGGACAGAGGTTCTGGCTGTGATCCTGCTCGTGCCGAATTCCCAGATGGCTGCAGGCAGGCATGCCTCAGAGCACTTCATGGAGCAGAAAAGGCCGAGTGTGACTTTGTGAACGGGACGCAGCTCGTGCGGTTTGTGGAGAGGCACATCTACAACCGGGAGGAGTTCGGGCGCTTTGATAGCGACGCGGGGGAGTTTGTGGGGGTGTCGGAGCTGGGCTGGCGCGGTGCGGACAGCTGGAACAGCAGGAAGGAGCTCCTGGGGCAGAAACGCGCCCGAGGGGACACTTTCTGCAGGCACACCCACAAGGTGATCGAGCCCTTCTCAGTGCGCAGACCCGTTGAACCTGAGGTGACTGTGTATCCATCAAAGATGGCTCCCTCGGGACACCACAACCTGCTTGTCTGCTCTGTTACTGGTTTTTATCCTGGGGACATTGAGGTCAGGTGGTTCCTGAACGGGCAGGAGGAGACAGCTGGGGTTGTGTCCACAGGCCTGGTCAGCAATGGAGACTGGACTTACCAGATCTTGGTGATGCTGGAAATGACCCCCAAGTGTGGTGATGTCTACAGCTGCCAAGTGGAGCACTCCAGCCTTCAGAAACCTGTCATCTTGGACTGGAAAGCACAGTCTGACTCTGCCCAAAATAATATGCTGCATGAACTCAGGAGCCTTGTATTGGGACTGATCTTCTTTGGAGTTGGCCTCATGGTCTACATGA

>Lahi_DAB_2

ATGGTGTGTGTCTTGTTCCCCCAGGGTATCTGGATGGAGGTTCTGACACTGCTGGTGCTGAATGCTCAGGTGACTGTAGGCAGACATGCCCCAGAGCACTTCACGGAGCAGGTGAAGGGCGAATGTTACTTTGTGAACGGCACTGAGCACGTGCGGTTTGTGATGAGGGCCATCTACAACCGGGAGGAGTACGTGCGCTTCGACAGCGACGTGGGGGAGTTTGTGGCGATGACGCCGCTGGGGCGCCTCACGGCGGAGTTTGGTAACAGCCAGAAGGAGTTCATGGACCACTTACGGGAGGCGGTGGACACTTACTGCAGGCACAACTACGAGGGGATCGAGCTCTTCACAGTGCCCAGGAGCGGTGATTCAGCCTGAGGTGACTGTGTATCCATCAAAGCTGGCTCCCCTGGGACACCACAACCTGCTTGTCTGCTCTGTCACTGGTTTCTATCCTGGGGACATTGAGGTCAAGTGGTTCCTGAATGGGCAAGAGGAGACAGCTGGGGTTGTGTCCACAGGCCTGATCAGCAATGGAGACTGGACTTACCAGATCCTGGTGATGCTGGAAATGACCCCTAAGCATGGAGATATCTACACCTGCCAAGTGGAGCACTCCAGCCTTCGGAGACCTATTATTTTTGACTGGAAAGCACAGTCTGAATCTGCCCAGAATAAGATGCTGAGTGGAGTTGGGGTTCTTGTGCTGGGGCTGATCTTCTTTGGGGTTGGCCTCATCGTCCACAAGAGGAGTCAGAAAGGTGA

>Lahi_DAB_3

ATGGCATGTGTCTTGCTTCACAGAGGCATCTGGACAGAGGTTCTGACTGTGACTCTGCTGGTGCTGAATTCCCAGGTGACTGCGGACAGACATGCCCCAGAACACTTCATGGAGCAGAGAAAGGTCGAGTGTCACTTTGTGAATGGGACAGAGCATGTGCGGTATGTACTGAGATGCATCCATAATAGGGAGGAGATCCTGCGCTTCGACAGTGATGTGGGGAAGTTTGTGGCACTGACAGAGCTGGGCCGGCCTGAGGTTGAGTATTGGAATAGCCAGAAGAACATCCTGAAGTATAGACAAGATCAAGTGAACACTTACTGCAGGCCCAACTACCAGGCTGTTAAAGTCTTTTCACAGAGCAGGAACGTGAACCTGAGGTGATTGTGTATCCATCAAAAATGGCTCTCCTGGGATATCACAACCTGCTTGTCTGCTCTGCCACTGGTTTCTATCCTGGAGACATTGAGGTCAAGTGGTTCCTGAATGGGCAAGAGGAGACAGCTGGGGTTGTGTCCACAGGCCTGATCAGCAATGGAGACTGGACCTACCAGATCTTGGTGATGCTAGAAATGACCCCCAAGCATGGAGATGTCTACACCTGCCAAGTGAAGCACTCTAGCCTTCAGAGACCTGTCACCTTGGACTGGAAAGCACACTCAGAATCTGCCCGGAGTAAGATGCTGAGTGGAGTTGGGAGCCTCTTGCTGGGCCTGATCTTCTTGGGGGTTGGCCTCATTGTCCACAAGAGGAGTCAGAAAGGTGA

>Lahi_DAB_4

ATGTCTTGCTCCCCAGGGGCATCTGGACAGAGGTTCTGGCTGTGACCCTGCTGGTGCTGACTTCCCAGGTGGTTGCAGGCAGAAATGCCCCAAAGCACTTCACGGAGCAGGTGAAGTCCGAGTGTCACTTTGTGAACGGGACGCAGCACGTGCGGTTTGTGAAGAGATACATCTACAACCGGGAGGAGTACGTGCGCTTCGACAGCGACAGCGCCGTGGGGGAGTTCGTGGCGGTGACGGAGCTGGGGCGCCGCGATGCTTTGAGCCCGAGGTGATTGTGTATCCATCAAAGCTGGCTCCCCTGGGACACCACAACCTGCTTGTCTGCTCTGTCACTGGTTTCTATCCTGGGGACATTGAGGTCAGGTGGTTCCTGAATGGGCAGGAGGAGACAGCTGGGGTTGTGTCCACAGGCCTGGTCAGCAATGGAGACTGGACTTACCAGATCTTGGTGATGCTGGAAATGACCCCCAAGCGTGGTGATGTCTACACCTGCCACGTGGAGCACTCCAGCCTTCAGAAACCTGTCATCTTGGACTGGAAAGCACAGTCTGAATCTGCCCAGAGTAAGATGCTGAGTGGAGTCGGGGGGCTCGTGCTGGGGCTGATCTTCTTTGGGGTTGGCCTCATTGTCCACAAGAGGAGTCAGAAAGGTG

>Lahi_DAB_5

ATGCTCTGTGTCTCCGTCTCCAGAGGCATCTTGACAGAGGTCCTGGCTATGACCCTGCTGGCACTGAATTTCCAGGTGGCTGCAGGCAGACATGTCCCAGCTGATGGTCTCTTCTTAGGCTTCCTCTTTCAGGAACTTTCTGCTGAACCTGCCCCTGTCAACCTCCCTCAGCTCTTGGGTATTTTCTCCTCTCTTGGTTCTCTGCTTACCTGTCTGGTCACTTTTTCTCAGTTCCGCTGACGGAGCTGGGGCGGGGGATTGCGGAGGGTTGGAACAAACGGAAGGAGCTCCTGGAGGACGAACGGGCCTACGTGGACACTTTCTGCAGGCACAACTACGGGGTGTTTGAGCCCTTCTCAGTGCCCAGGCGGGGTGATTGAGCCCGAGGTGATTGTGTATCCATCAAAGCTGGCTCCCCTGGGACACCACAACCTGCTTGTGTGCTCTGTCACTGGTTTCTATCCTGGAGACATTGAGGTCAGGTGGTTCCTGAATGGGCAGGAGAAGACAGCTGGGGTTGTGTCCACAGGCCTGGTCAGCAATGGAGACTGGACTTACCAGATGTTGGTGATTCTGGAAATGACCCCCAAGCGTGGTGATATCTACACCTGCCAAGTGGAGCACTCCAGCCTTCAGAAACCTGTCATCTTGGACTGGAAAGCACAGTCTGAATATGCCCAGAGTAAGATGCTGAGTGGAGTCGGGGGGCTCATGCTGGGGCTGATATTCTTTGGGGTTGGCCTCATTGTCCACAGGAAGAGTCAGAAAGGTGA

>Lahi_DAB_6_partial_exon_2-4

AGCACTTCACGGAGCAGGCAAAGTATGAGTGTTACTTTGTGAACGGGACGCAGCACGTGCGGTTTGTGGAGAGATATATCTACAACCGGGAGGAGTTTATGCGCTTCGACAGCGACGTCGGGGTGTACGTGGCGCTGACGGAGCTGGGGCGGGGGATTGCTGAGGGTTGGAACAGCCAAAAGGATTACATGAAGAGCAGACGGGGCGTGGTGGACACTTTGTGCAGACCCAACTACGGGGTGTCTGAGCCCTTAGTGCGCAGGCGCGGTGATTCAGCCCAAAGTGATTGTGTATCCTTCAAAGATGGCTCCCCTGGGACACCACAACTTGCTTGTCTGCTTTGTCACTGGTTTCTATCCTGGGAACATTGAGGTCAGATGGTTCCTGAATGGGCAGGAGAAGACAGCTGGGGTTGTGTCCACAGGCCTGATCAGCAATGGAGACTGGACTTACCAGATCTTGGTGATGCTGGAAATGACCCCCAATCGTGGTGATGTCTATACCTGCCAAGTGGAGCACTCCAGCCTTCAAAAACCCGTCATCTTGGACTGGAAAGCACAGACTGAATCTGCCCAGAGTAAGATGCTGAGTGGAGTTGGGAGCCTCATTTTGGGGCTGAGCTTCTTTGGAATTGGCCTGATAGTCCACAAGAGGAGTCAGAAAGGTGA

>Lahi_DBB_1

ATGCTTGATGTTTGGATCTTTGTTGGTTACTGGAAGATTGGTCTGTTAATGACTTCGATGCTGTTCAGTCTGTCTGCATCTTGGGCCAGGGACGTCCCACAGAATTTCTTGTATCAGGACAAGGCTGAGTGTTATTTCACCAACGGCATGGAACTGGTGCTCTTTGTGGTTAGATAGTCTACGAAGACCAGAAATTTCTCCGCTTTGACAGCGACGTGGGGAAGTTCCTGGCGGAGGGAGCTGGGCGGAGTCAGGCTGAGTACTGGAACAGTCTGGAGGAGATGCTGGAGCAAAATCAAGCCGCCGTAGAAACGCTGTGCAGATACAACTACGACGTTGGCAAGCCTTTCATGGTAGACGGAAGAGTACCAGACCCTGGTCATGTTGGAAATCACTCCCCAGAGCAGAGATGTCTACAGCTGCAGTGTGGAGCATGCCAGCCTACAGAGCCCCATCAGTGTGGAATGGAGGGCACAGTCTGAATCTGCCCAGAGCAAACTGCTGAGTGGAATTGGAGGCTTTGTCCTGGGGCTGATCTTCCTCAGTGTAAGACTGATTATCCACCTGAAGAACAAGAAAGGTGA

>Lahi_DCB_1

ATGGAACATGTGGAGTTTCTGGGAGACCCTGTATGACAGTCATACTGATAGTACTGAGCACACCCACAGACTGAGGCAGGGACATTCCAAAGAATTACCTACATCAGATGGGGTCTGAGGGTCACATGATCAATGGAACCCAACAAGACTCATCTACAACCAGGTGGAGTTTGTGCACTTTGATAGTGATATGGGACTATTTCAGACAAGCTGTGGAAATCCCAAGTCCAGAAATGAAACAGTAAAAAGGAGAAAGTCAAGCATGCAAGGTCCATAATGAATGCGTGCAGAGGCAATTTTCTTTTATATGACAAATTCATAGTGCAAAGGAAAGCCCCTAGTGAAAGTTTTACCCCCAAAGATACAACCACCTGGGCACCACAACCTGCTCCCCTGTTCTGTGATGTTTCTGTGCTGGTGAGATCAAGATCAATTGATTCAGGATCACAAAAGAAGAGAAGACTTGAATCCTGTCCACAGATCGAATTCAGCACAGTGACTGGACCTTCCAAACCCTTGTGATGCTAGAAATGGCCCCCCAAAGAGAAGATGTCTTTACTTTCCATGTGGACCATGTCAGCTTGCAGAGCTCTGTCATTGTAAACTGGAGAGCACATTCTGAATCTTCCTGGACTAAGAGGCTCACTGGAATTCAGGGCTTGGTATTTGGACTGATCTTACTTGGAACTTGTCAGCCACCTCAGAAATTTGAAAGGTTCCTATTCTGGTACCAAAGAAGACCCAGATTTTGAAGGGATTGTGAACATTGCCTCATTGCAACAAGATTTTCCCAGAACTGTTGCCCAGTCCTAG

>Lahi_DMB_1

ATGACATTACTCTACCTGCTACTAGTGGGCTTCAGCCTGGGTTTTTCAGGAACAGGGGGCTTTGTGACTCATGTGGAGAGTGACTGTATACTGGATGAAGAAGGATCCGTAAAGGACTTCCAATACTGCATCTCCTTCAACAAGGATGTGTTGACCTGCTGGGACTCAGATACTAGCAAGATGGTCACTGTTGATTTTGGAATACTGCATCAGTTAGCTGACCAGCTTTCTGACGCCCTCAATAATGATACTGCTTTGATAAACCACCTGAGCGAAGGATTCCAAAACTGTGCCAGTCACACAAAGCCCTTCTGGGGATCACTGACCCACAGGACACGGCCACCATCAGTGCAAGTAGCTCAGGTCACACCATTCAACACAAGGGAGCCAGTGATGCTGGCCTGTTATGTATGGGGCTTCTATCCTGCTGATGTGGCCATTTCGTGGTTGAAGAATGGGCAGCCTGTCCCACACAGTGGCATCCAGAAGGCTGTACAATCCAATGGGGACTGGACCTATCAGACACGATCCTACTTGGCCCTTACCCCCTCTAGTGGGGATATTTACACTTGCTTTGTAGAGCATGGTGGGACTTCCCAGGCCATCTTACAGACCTGGACACCTGGCCTCTCCCTGAAGCAAATAGTGAAGATCTCTGTATCTGTACTGACTCTGAGCTTTGGTCTTATCTTCTTCTTCCTTGGCCTGGTTGCACGTAGAAAAGCTGGCTCCTCTGACTACTCTCCTCTCTCGGGGTCCAATTATCCTGAAGGTA

>Noty_DNAzoo_DAB_1

ATGGTATGTGTCTTGCATGTTAAGAGGATCTGGATAGAGATTCTGGCTGTGACCCTGCATGTGATGACTTCCCAGGTGGCTACAGGCAGACACATCCCAGAGCACTTCACATTGTATACTACTTCTGAGTGTTACTTCGTGAACGGCACGGAGCACGTGCGGTATGTGCAGAGATTCATCTACAGCCCGGAGGAGTACGCGCGCTTCGACAGTGACGTGGGGAAGTATGTGGCGCTGACCGAGCTGGGGCGGCCCCAAGCGCAGTATTGGAACAGCCAGGAGGAGCTCCTGGAGGACAAACGGGCCCGGGTGGACACTTACTGCAGGCACAACTACCAGGTGTCTGAGCCCTTCTTAGTGCGCAGGCGCGGTGATGGAGCCCGAGGTGATTGTGTATCCATCCAAGATGGCTCCCCTGGGACACCACAACCTGATTGTCTGCTCTGTCAGTGATTTCTATCCTGGGGATATTGAGGTCAGGTGGTTCCTGAATGGGCAGGAGGAGACAGCGGGGTTGTCCACAGGCTTGATCAGCAATGGAGACTGGACCTACCAGACCCTGGTGATGCTGGAAATGACCCCCAAGCATGGAGATGTCTACACCTGCCAAGTGGAGCACTCCAGCCTTCAGACACCTGTTGTTGTGGACTGGAAAGACCAGTCTGAATCTTGCTCCAGAGTAAGATGCTGAGTGGAGTCGGGGCCTGGTGCTGGGGCTGATCTTCTTTGGGGTTGGCCTCATTGTCCACAAGAGGAGTCAGAAAGGTGA

>Noty_DNAzoo_DAB_2

ATGGTGTGTGTTTTGTTCTCCAAGGGTGTCTGGACAGAGGTTCTGGCTGTGACCCTGCTGGTGCTGAATTCCCAGGTGGCTGCAAGCAGACACACCCCAGGTAAAGCATTTCATGGAGCAGGCCAAGTGAGTGTTACTTCGTGAACGGTACGGAGCACGTGCGGTTTGTGGAGAGAACATACACAACCGGGAGGAGTATGCGCGCTTCGACAGTGACGTGGGGAAGTACGTGGCGCTGACCGAGCTGGGGCGGCACCAGGCGGAGTATTTGAACAGCCAGGAGGATATTCTGGAGGACAGAAACGGGCCTCGGTGGACACTTACTGCAGGCACAACTACCAGGTGTCTGAGCCCTTCTTAGTGCGCAGGCGCGGTGATGGAGCCCGAGGTGATTGTGTATCCATCCAAGATGGCTCCCCTGGGACACCACAACCTGATTGTCTGCTCTGTCAGTGATTTCTATCCTGGGGACATTGAGGTCAGGTGGTTCCTGAATGGGCAGGAGGAGACAGCTGGGGTTGTGTCCACAGGCTTGATCAGCAATGGAGACTGGACCTACCAGACCCTGGTGATGCTGGAAATGACCCCCAAGCATGGAGATGTCTACACCTGCAAGTGGAGCACTCCAGCCTTCAGAAACCTGTTGTTAAGCCCAGTCTGAATCTGCCCAGAGTAAGATGCTGAGTGGAGTCGGGGGCCTGGTGCTGGGGCTGATCTTCTTTGGGGTTGGCCTCATTGTCCACAAGAAGAGTCAGAAAGGTGA

>Noty_DNAzoo_DBB_1

ATGGTTGATATTTGGATCTCTGCTGGCTGCTGGAAGATTGGTCTGTTAATGACATCAGTTCTGTTAAATCTACCTGTATATTGGGCCAGGGATATCCCAGATGATTTTGTGATCCAGGAAAAGGGTGACTGTTACTTCACCAACGGCACGGCGAAGGTGCGGCACGTGTATAGATACATCTACAGTGACCAGGAGATTGTCCGCTTCGACAGCGACGTGGGGAAGTTCGTGGCCCTCGCGGAGCTGGGGCGGCCCGATGCTGAGTATTGGAACAGTCCGGAGGACTTCCTGGAGCGTCAGCGAGCCTGCGTGGACGAGCTGTGCAGACACAATTAGGACACCGACAAGCCCTTCACGGTGGATAGAAGAGTCCGCCCCACTGTGACCATCTCCCCCTCTAAGTCAGAGCCCTCGCAGCACCTACTGGTCTGCTCTGTCACTGACTTCTATCCAAGCAAAGATCAAGGTCAGCTGGTTCAAGAATGGGCAGGAGGAGACAGCTGGGGTCATGTCCACAGGAGTGATGCAGAATGGAGACTGGACCTACCACACCCTGGTCATGTTGGAAATGACTCCCCAGAGAAGAGATATCTACGCCTGCAGCGTGGAACATGCCAGCCTAGAGAGCCCCATCACTGTGGAGTGGAGGGCACAGTCTGAATCTGCCCAGAGCAAATTGCTGAGTGGAGTTGGAAGCTTTGTCCTGGGGCTGATGTTTCTCAGTGTAGGACTGATCATCTACCTGAAAAACCCGAAAGGTAA

>Noty_DNAzoo_DBB_2

ATGGTGAATATTTGGATCTCCTCTGGCTGCTGGAAGATTGGCATGTTAATCCTTGATGGCATTAAATCTACCTGTACCTTGGGCCAGGGACATCCCAGGATTTCGTGATTCAGGCCAAGGGTGACTGTGATTTCATCAACAGCACGGAGTAGGTGCGGCATGTGTATAGATACATCTACAATGACCAGGAGACCGTTCGCTTCGACAGCGACGTGGGGAAGTTCGAGGCCATCACGGAGCTGGGGCGACGACAGGCTGAGGATTGGAACAGACAGAAGGACATGGTGGAGCATCATCGAGCCTGCGTGGACGAGGTGTGCAGACACAACTATGAGATAGAAAAACCCTTCACGGTGAACAGAAGAGGTGATCAAGCCCACAGTGACCATCTCCCTCTCTAAGACATAGGCCCTGCATCACCTGCTGGTCTGCTCTGTCACCGGCTTCTATCCAAGCAAGATCAAGGTTAGCTGGTTCAAAAATGGGCAGGAGGAGACAGCTGGAATTATGTCCACGGGTGTGATGCAGAATGGAGACTGGACCTACCAGACCCTGGTCATGTTGGAAACTACTCCCCAGAACAGAGATGTCTACAACTGCAGTGTGGAGCATGCCAGCCTAGAGAGCCCCATCACTGTGGAATGGAGGGCACGGTCTGAATCTGCCCAGAGCAAATTGCTAAGTGGAGTTGGAAGCTTTGTCCTGGGGCTGATCTTCTTCAGTGTAGGACTGATCATTTACCTGAAGAACCAGAAAGGTGA

>Noty_DNAzoo_DBB_3

ATGGTTAATATTTGGATCTCTGCTGGCTACTGGAATATTGGTCTGTTGATGACATCCACGGTGTTAAGTCTACCTGTATCTTGGGCCAGAGATGTCCCGGAGGATTTCGTGATCCAGGGCAGGTATTACTGCTACTTCACCAACGGCACGGAGAAGGTGCGGTTTGTGTATAGATACATCTACAATGACCAGGAGATTGTCCGCTTCGACAGCGACGTGGGGAAGTTCGTGGCCATCACGGAGCTGGGGCGGCCCGATGCTGAGTATTGGAACAGTCAGGAGGACACGGTGGAGGAATATCGAGCCTACGTGGACACGGTGTGCACACACAACTACAAGACAGAAAAGCCCATACCGTTGGATAGAAGAGTCCGGCCCACAGTGACCATCTCCCCTCTAAGTCCGAGGGCGCAGCACCTGCTGGTCTGCGCTGTCACTGACTTCTATCCAAGGAAGATCAAGGTCAGCTGGGCCAAGAATGGGCAGGAGGAGACAGCTGGGGTTGTGTCCACGGGTGTGATCCAGAATGGAGACTGGACCTACCAGACCCTGGTCATGTTGGAGATGACTCCCAGAGCAGAGATGTCTACACCTGCAGCGTGGAGCATGCCAGCCTCAAGAGCCCCATCACTGTGGAGTGGAGGGCACGGTCTGAATCTGCCCAGAGCAAATTGTTGAGTGGAGTTGGAGGCTTTGTCCTGGGGCTGATGTTCCTCAGTGTAGGACTGATCATCCACCTGAAGAACCAGAAAGGTAA

>Noty_DNAzoo_DMB_1

ATGAGGTTACTTTACCTACTATTAGTGGACTTCAGCCTGGGTTTTTCAGGACATTTTTCAGCAGGGGGCTTTGTTACCCATGTGGAGAGTGGCTGTGTACTGGATGAAGATGGATCAACAAAAGACTTCACATATTGTGTCTCCTTCAACAAGGATGTGTTGAGCTGCTGGAACTCAGAGATTAAGCAGATGGTCACCGTTGACTTTGGGATGCTGAATCCATTAGCTGACTACCTTTCTAAAAATTTAAATAATAGGAGTACCTTGATAGACTACCTGAGCAAAGGACTCCAGAACTGTGCCAGTCACACAAAGCCCTTCTGGGGGTCATTGACCCAAAGGAAACGACCACCATCAGTGCAAGTAGCCCAGGTCACACCATTCAACACAAGGGAGCCAGTGATGCTGGCTTGTTATGTATGGGGTTTCTATCCCCCTGATGTGGCCATTTCATGGTTGAAGAATGGGGAGCCTATTCCACACAGTGTCATTCAGAGGAATGTACAGTCCAATGGGGACTGGACTTATCAGACACGATCCTATTTGCCTCTTACCCCCTCTAGTGGAGATATTTACACTTGTCACGTAGAGCACAGTGGGATTTCCCACCCCATCTTACAGACCTGGACACCTGGCCTATCTCTGCAGCAGATGGTGAAGATCTCTGTTTCTGTATTGACTCTGAGCCTTGGTCTCATCTTCTTCTTCCTTGGTCTGGTTACTTGCCGAAAAGTTAGCTCCTCTGGCTACACTCCTATCTCAGGGTCCAATTATCCTGAAGGTAA

>Drgl_DAB_1

ATGGTGTGTGTCTGGCTCCCCAAGTGCGTCTGGATAGAAGTTCTGGCTGTGACGCTGCTGGTGCTGAATTCCCAGGTGGCTGCAGGCAGACAGGTCCCAAAGCACTTCACTGAGCAGGGTAAGGCTGAATGTCACTTTGAGAACGGGACGGAGCGCGTGCGGCTCGTGGAGAGACACGTGTACAACCGGGAGGAGTACGTGCGCTTCGACAGCGACGTCGGGGAGTTCGTGGCGGTGACGGAGCTGGGGCGGCCTGATGCTGAGTATTGGAACAGCCAGAAGGAGATCCTGGAGGACAGACGGGCCGCTGTGGACAATTACTGCAGGCACAACTACGAGATTCTTGATCGCTTCTTGGTGCCCCGGCGCGGTGAttGAGCCCGAGGTGACTGTGTATCCATCCAAGATGGCTCCCCTGGGACACCACAACCTGCTTGTCTGCTCTGTCACTGGTTTCTACCCTGGGGACATTGAGGTCAGGTGGCTCCTGAATGGGCAGGAGGAGACAGCTGGGGTTGTGTCCACAGGCCTGATCAGCAATGGAGACTGGACCTACCAGGTCCTGGTGATGCTGGAGATGACCCCCAGGCGTGGGGATGTCTACACCTGCCACGTGGAGCACTCCAGCCTGCAGAGACCTGTCACCTTGGACTGGAAAGCACAGTCTGGATCTGCCCAGAGCAAGATGCTGAGTGGAGTCGGGGGCCTCGTGCTGGGGCTGATCTTCTTTGGGGTTGGCCTCGTTGTCCACAGGAGGAGTCAGAAAGGTGA

>Drgl_DAB_2

ATGGTGTGTGTCTGGCTCCCCAAGGGCATCTGGATAGGAGTTCTGGCTATGACCCTGCTGATGCTGAATTCCCAGGTGACTGCAGGCAGACAGGTCCCAGAGCACTTCACTCGGCAGAGTAAGGCTGAATGTCACTTTGAGAAGGGGACGGAGCGCGTGCGGCTCGTGGAGAGATATTTCTACAACCGGGAGGAGTACGTGCGCTTCGACAGCGACGTCGGGGAGTTCGTGGCGGTGACGGAGCTGGGGCGGCCTGATGCTGAGTATTGGAACAGCCAGAAGGAGATCCTGGAGGACAGACGGGCCGCTGTGGACACTTACTGCAGGCACAACTACGAGATTCTTGATCGCTTCTTGGTGCCCCGGCGCGGTGAttGAGCCCGAGGTGACTGTGTATCCATCCAAGATGGCTCCCCTGGGACACCACAACCTGCTTGTCTGCTCTGTCACTGGTTTCTACCCTGGGGACATTGAGGTCAGGTGGCTCCTGAATGGGCAGGAGGAGACAGCTGGGGTTGTGTCCACAGGCCTGATCAGCAATGGAGACTGGACCTACCAGGTCCTGGTGATGCTGGAGATGACCCCCAGGCGTGGGGATGTCTACACCTGCCACGTGGAGCACTCCAGCCTGCAGAGACCTGTCACCTTGGACTGGAAAGCACAGTCTGGATCTGCCCAGAGCAAGATGCTGAGTGGAGTTGGGGGCCTCGTGCTGGGGCTGATCTTCTTTGGGGTTGGCCTTTTTGTCCACAGGAGGAGTCAGAAAGGTGA

>Drgl_DAB_3

ATGGTGTGTGTCTGGCTCCCCAAGGGCATCTGGATAGGAGTTCTGGCTGTGACCCTGCTGATGCTGAATTCCCAGGTAACTGCAGGCAGACAGGTCCCAGAGCACTTCACCGAGCAGTTGAAGGCGAATGTTACTTTGAGAACGGACGGAGCGCGTGCGGTTTGTGGCGAGGTCCATCAGCAACGGGTAGAGACTGTGCGCTTCGACAGCGACGTCGGGGAGTTTCGTGGCGGTGACGGAGCTGGGGCGGCCTGATGCTGAGCTTTGGAACAGCCAGAAGGAGGAACTGGAGCGGAGACCGGGCCGAGGTGGACACTTACTGCAGGCACAACATACGTGGTTGTTTGAGCCCGTCTTTGGTGCCCGGCGCGGTGAttGAGGCCCGAGGTGACTGTGTATCCATCCAGATGGCTCCCCTGGGACACCACAACCTGCTTGTCTGCTCTGTCACTTGGTTTCTACCCTGGGGACATTGAGGTCAGGTGGCTCCTGAATGGGCAGAGGAGACAGCTGGGGTTGTGTCCACAGGCCTGATCAGCAATGGAGACTGGACCTACCAGGTCCTGGTGATGCTGGAGATGACCCCCAGTGCGTGGGGATGTCACACCTGCCACGTGGAGCACTCCAGCCTGCAGAGACCTGTTCACCTTGGACTGGAAAGCACAGTCTGGATCTGCCCAGAGCAAGGTGCTGAGTGGAGTCGGGGGCCTCGTGCTGGGGCTGATCTTCTTTGGGGTTGGCCTCGTTGTCCACAGGAGGAGTCAGAAAGGTGA

>Drgl_DAB_4

ATGGTGTGTGTCTGGCTCCCCAAGGGCATCTGGATAGGAGTTCTGGCTGTGACCCTGCTGATGCTGAATTCCCAGGTGACTGCAGGCAGACAGGTCCCAGAGCACTTCACTCGGCAGGGTAAGGCTCAATGTTACTTTGAGAAGGGGACGGAGCACGTGCGGCTCGTGGAGAGACACGTGTACAACCGGGAGGAGTTTCTGCGCTTCGACAGCGACGTCGGGGAGTTCGTGGCGGTGACGGAGCTGGGGCGGCCTCAGGCTGAGAAATGGAACAGCCAGAAGGAGATCCTGGAGGACAGACGGGCCCAGGTGGACAATTACTGCAGGCACAACTACGAGGTGTTTGAGCCCTTCTTGGTGCCCCGGCGCGGTGAttGAGCCCGAGGTGACTGTGTATCCATCCAAGATGGCTCCCCTGGGACACCACAACCTGCTTGTCTGCTCTGTCACTGGTTTCTACCCTGGGGACATTGAGGTCAGGTGGCTCCTGAATGGGCAGGAGGAGACAGCTGGGGTTGTGTCCACAGGCCTGATCAGCAATGGAGACTGGACCTACCAGGTCCTGGTGATGCTGGAGATGACCCCCAGGCGTGGGGATGTCTACACCTGCCACGTGGAGCACTCCAGCCTGCAGAGACCTGTCACCTTGGACTGGAAAGCACAGTCTGGATCTGCCCAGAGCAAGATGCTGAGTGGAGTCGGGGGCCTCGTGCTGGGGCTGATCTTCTTTGGGGTTGGCCTCGTTGTCCACAGGAGGAGTCAGAAAGGTGA

>Drgl_DAB_5

ATGGTGTATGTCTTGCTCCCCAAAGGTATCTGGATAGAAGTTCTGGCTGTGACCCTGCTCTTGCTGAATTCCCAGGTGGCTGCAGTCAGACAGACCCCAGAGCACTTCACTGAACAGTTGAAGTTTGAGTGTCACTTTGAGAACGGGACGGAGCGCGTGCGGCTTGTGGTGAGGTACATCTACAACCGGGAGGAGCACGTGCGCTTCGACAGCGACGTCGGGAAGTTCTTGGCGCTGACGGAGCTGGGGCGGCCCAGTGCTGAGAAGAGGAACAGCCAGAAGGAGGAACTGGAGCAATTTCAGGCCCAGGTGGACACTGTCTGCAGGCACAACCACGTGGTGTTTGAGCCCTTCTTAGTGCGCAGGAGCGGTAAttGAGCCTGAGGTGACTGTGTATCCATCCAAGATGGCTCCCCTGGGACACCACAACCTGCTTGTCTGCTCTGTCACTGGTTTCTACCCTGGGGACATTGAGGTCAGGTGGCTCCTGAATGGGCAGGAGGAGACAGCTGGGGTTGTGTCCACAGGCCTGATCAGCAATGGAGACTGGACCTACCAGGTCCTGGTGATGCTGGAGATGACCCCCAGGCGAGGGGATGTCTACACCTGCCACGTGGAGCACTCCAGCCTGCAGAGACCTGTCACCTTGGACTGGAAAGCACAGTCTGGATCTGCCCAGAGCAAGATGCTGAGTGGAGTCGGGGGCCTCGTACTGGGGCTGATCTTCTTTGGGGTTGGCCTTGTTGTCCACAGGAGGAGTCAGAAAGGTGA

>Drgl_DAB_6

ATGGTGTGTGTCTGGCTCCCCAAGGGCATCTGGATAGGAGTTCTGGCTGTGCCCCTGCTGGTGCTGGTTTCCCAGGTGGCTGCAGGCAGACAGGTCCCAGAGCACTTCACTGAGTATTCTACATCTGAGTGTTACTTTGTGAATGGGACGGAGCGCGTGCGGTATATGGACAGATATTTCTATAACCGGGAGGAGTACGTGCGCTTCGACAGCGACATCGGGGAGTACCTGGCGGTGTCGGAGCTGGGGCGGGGAATTGATAACTACCTGAACAGCCAGAAGGAGATCCTGGAGGACAGACGGGACTCAGTGCACACTTTCTGCCGGCACAACTACAAGGTGTTTGATCCCTTCCTAGTGCCCCGGCACGGTGAttGAGCCTGAGGTGACTGTGTATCCATCCAAGATGGCTCCCCTGGGACACCACAACCTGCTTGTCTGCTCTGTCACTGGTTTCTACCCTGGGGACATTGAGGTCAGGTGGCTCCTGAATGGGCAGGAGGAGACAGCTGGGGTTGTGTCCACAGGCCTGATCAGCAATGGAGACTGGACCTACCAGGTCCTGGTGATGCTGGAGATGACCCCCAGGCGTGGGGATGTCTACACCTGCCACGTGGAGCACTCCAGCCTGCAGAGACCTGTCACCTTGGACTGGAAAGCACAGTCTGGATCTGCCCAGAGCAAGATGCTGAGTGGAGTCGGGGGCCTCGTGCTGGGACTGATCTTCTTTGGGGTTGGCCTTGTTGTCCACAGGAGGAGTCAGAAAGGTGA

>Drgl_DAB_7

ATGGTGTGTGTCTGGCTCCCCAAGGGCATCTGGATAGGAGTTCTGGCTGTGCCCCTGCTGGTGCTGGTTTCCCAGGTGGTTGCAGGCAGACAGGTCCCAGGGCACTTCACAGAGTATTCTACATCTGAGTGTTACTTTGAGAACGGGACTGAGCGCGTGCGGTTTGTGGAGAGACACATCAGCAACGGGGTAGAGACTGTGCGCTTCGACAGCGACATCGGGGAGTACGTGGCGGTGACGGAGCTGGGGCGGCCTGATGCTGAGCTTTGGAACAGCCAGAAGGATTCCATGGAGCAGAGACGGGCCGCTGTGGACACTTTCTGCAGGCACAACTACAAGTTGTCTGAGAGCTTCCTAGTGCCCCGGCGCGGTGAttGAGCCTGAGGTGACTGTGTATCCATCCAAGATGGCTCCCCTGGGACACCACAACCTGCTTCTCTGCTCTGTCACTGGTTTCTACCCTGGGGACGTTGAGGTCAGGTGGCTCCTGAATGGGCAGGAGGAGACAGCTGGGGTTGTGTCCACAGGCCTGATCAGCAATGGAGACTGGACCTACCAGGTCCTGGTGATGCTGGAGATGACCCCCAGGCATGGGGATGTCTACACCTGCCACGTGGAGCACTCCAGCCTGCAGAGACCTGTCACCTTGGACTGGAAAGCACAGTCTGGATCTGCCCAGAGCAAGATGCTGAGTGGAGTCGGGGGCCTCGTGCTGGGACTGATCTTCTTTGGGGGTGGCCTTGTTGTCCACAGAAGGAGCCAGAAGGTGA

>Drgl_DAB_8

ATGGTGTGTGTCTGTTTCCCCAAGGGCATCTGGATAGAAGTTCTGGCTGTGACCCTGTTGATGCTGAATTCCCAGTTGGCTGCAGGCAGACAGGTCCCAGAGCACTTCACTGAGCAGTTTAAGTATGAGTGTCACTTTGAGAACCGGACAAAGCGCTTGGGGCTTGTGGAGAGACACGTGTACAACCGGGAGGAGTTGGTGCGCTTCGACAGCGACATTGGGGAGTACGTGGCGGTTACCGAGCTGGGGCGGCGCATTGCTGAGGACTATAACAGCCAGAAGGAGTTCATGGAGCAGAGACGGGCCGCTGTGGACACTTACTGCAGGCACAACTACGAGATTCTTGATCACTTCTTGGTGCCCCGGCGCGGTGAttGAGCCCGAGGTGACTGTGTATCCATCCAAGATGGCTCCCCTGGGACACCACAACCTGCTTGTCTGCTCTGTCACTGGTTTCTACCCTGGGGACATTGAGGTCAGGTGGCTCCTGAATGGGCAGGAGGAGACAGCTGGGGTTGTGTCCACAGGCCTGATCAGCAATGGAGACTGGACCTACCAGGTCCTGGTGATGCTGGAGATGACCCCCAGGCGTGGGGATGTCTACACCTGCCATGTGGAGCACTCCAGCCTGCAGAGACCTGTCACCTTGGACTGGAAAGCACAGTCTGGATCTGCCCAGAGCAAGATGCTGAGTGGAGTCGGGGGCCTCGTGCTGGGGCTGATCTTCTTTGGGGTTGGCCTTGTTGTCCACAGGAGGAGTCAGCAAGGTGA

>Drgl_DBB_1

ATGGTTGATGTTTGGATCTCTCCTGGCTGCTGGAAGATTAGTCTGTTAATGACTCTGATGGTGCTGAGTTTACCTGTATCTTGGGCCAGGGACATTCCAAAGGATGCCGTGATCCAGGGCAAGGGTGACTGTTACTTCACCAACGGCACGGAGCGAGTGCGGTTTGTGGCGAGATACATCTACAATGACCAGGAATATGTCCGCTTCGACAGCGACGTGGGGAAGTTCGTGGCTGTCACGGAGCTGGGGCGGCCCGATGCTGAGTATTGGAACAGTCAGCAGGAGCTCATGGAGGAACATCGAGCCTACGTGGACACGCTGTGCAGACACAACTACAATAATAACAGGCCCTTCACGGTGGAGAGAAGAGtcCAGCCCAGGGTGACCATCTCCCCCTCCAAGACAGAGGCCCTGCAGCACCTGCTGGTCTGCTCTGTCACTGGCTTCTATCCAAGCAAGATCAAGGTCAGCTGGTTCAAGAATGGGCAGGAAGAGACAGCTGGGGTTGTGTCCACGGGTGTGATACAGAATGGAGACTGGACCTACCAGACCCTGGTCATGTTGGAAGTGATTCCCCAGAGCAGAGATGTCTACACCTGCAGCGTGGACCATGCCAGCCTACAGAGCCCCGTCACTGTGGAATGGAGGGCACAGTCTGAATCTGCCCAGAGCAAATTGCTGAGTGGAATTGGAGGCTTTGTCCTGGGGCTGATCTTCCTCAGTGTAGGACTGATCATCCACCTGAAGAACCAGAAAGGTGA

>Drgl_DCB_1_partial_exon_3-4

agtgaatGTTTTCCCCTCAAAGATACAACCACTTGGGTACCACAACCTGCTCATCTGTTCTGTGACCAGTTTCTTTCCTGGTGAGATCAAGGTCAGCTGATTCAAGAATGCAAAAGAAGAAGAGGCTGGAGTCCTGTCCACAGGCCAAATCCAGAATGGTGACTGGACCTTCCAGACCCTTGTGATGCTGGAAATAACCCCCCAAAGTGAATATGTCTTTACTTGTCATGTGGACCACATCAACTTGCAGAGCCCTGTCACCGTAGATTGGAGAGCACAGTCTGAATCTGCCTGGACTAAGATGTCGACTGGAGTTGGGGATTTGGTGCTTGGACTGATTTTACCTAGAGTGGGATTTGTCATCTGCCTCATAAATTTGAAAGTTAA

>Drgl_DMB_1

ATGAGGTTACTCCACCTACTACTAGTGGGCTTCAGCCTGGGTTTTTCAGGAGCAGCAGGGGGCTTTGTGACCCATGTGGAGACTGGCTGTGTGCTGGATGAAGATGGATCAGTAAAGGACTTCACATATTGTGTCTCCTTCAACAAGGATGTGTTGACCTGCTGGGACTCAGAGACTAACAAGATGGTCCCTGTTGACTTTGGGATACTGCATATGGTAGCTGAAGAGCTTTCTAAAGATCTCAATAAAGATAGTGACTTGAGAAACCGCCTGAGTAATGGATTCCAGGACTGTGCCAGTCACACAAAGCCCTTCTGGGGGCCATTGACCCACAGAACACGGCCACCATCAGTACTAGTAGCCCAGACCACGCCATTCAACACAAGGGAGCCGGTGATGCTGGCCTGTTATGTATGGGGCTTCTATCCTGCTGATGTGGCCATTTTTTGGTTGAAGAATGAGCAGCCTATCCCATACAGTGGCATCCAGAGGGCTGTACAATCCAATGGAGACTGGACTTACCAGACACGCTCTTACTTGGCCCTTACTCCCTCTAGTGGTGATATTTACACTTGCCGTGTAGAGCACAGTGGGAGTTCTCAGCCCATCTTAGAGACCTGGACACCTGGCCTCTCTCTGAAGCAGATAGTGAAGGTCTCTGTATCTGTAGTGACTCTGTGCCTTGGcctcatcttcttcttccttgGCCTGGTTATCTGCCGAAAAGCTGGCTCCTCTGaCTACACTCCTCTCTCAGGGTCCAATTATCCTGAAGGTAA

>Bupa_DAB_1_partial_exon_3-4

TTGAGCCCGAGGTGACTGTGTATCCATCAAAGACAGCTCCCCTGGAACACCACAACCTGCTTGTCTGTTCTGTCACTGGTTTCTATCCTGGGGACATTGAGGTCAGGTGGTTCCTGAATGGACAGGAGGAGACAGAAGGAGTTGTGTCCACAGGCCTGATCAGCAATGGAGACTGGACCTATCAGATCCTGGTGATGCTGGAAATGACCCCCACGAATGGGGATGTCTACACCTGCCAAGTGGAACACTCCAGCCTTCAGAGACCTGTCGTCTTGGACTGGAAAGCACAGTCTGAATATGGTCAGAGTAAGATGCTGAGTGGAATTGGGGGCCTTGTGTTGGGCCCTGATCTTCTTTGGGGTTGGCCTCATTGTCCACAAGAGGAGTCAGAAAGGTGA

>Bupa_DAB_2

ATGGCGTGTGTCTTGCTTTCTAGGGGTGTCTGGATAGAAGTTCTGGTTGTGATTCTGCTGGTGCTGAATTCCCAGGTGGCTGCAGGCAGACATGCCCCAGATCACTTCACGGTGCAGGTGAAAGGCGAATGTTACTTTGTGAATGGGACAGGGCACGTGCGGTTTGTGCTGAGAGGCATCTACAACCGGGAAGAGTACGTGCGCTTCGACAGCGACGTGGGGGATTTCGTGGCGATAACGGAACTGGGGCGGCGCACTGCTGCCTACGGTAAGAGCCAGAAGGAGTTCATGGACCACTTACGGTCTGCGGTGGACACTTACTGCAGGCACAACTACGAGGGATTCGAACCCTTCACAGTGCCCAAGAGTGGTGATTGAGCCCAAGGTGACCATGTATCCATCCAAGATGGCTCCCCTGGGACACCACAACCTGCTTGTCTGCTCTGTCACTGGTTTCTATCCTGGGGACATTGAAGTCAGGTGGTTCCTGAATGGGGCAGGAGGAGACAGCTGGGGTTGTGTCCACAGGCCTTATCAGCAATGGAGACTGGACCTACCAGACACTGGTGATGCTGGAAATGACCCCCAAGCGTGGAGATGTCTACACCTGCCAAGTGGAGCACTCTAGCCTTCAGAGACCTGTCATCTTCGACTGGAAAGCACAGTCTGAATCTGCCCGGAATAAGATGCTGAGTGGAGTTGGGGTCCTTTTGCTGGGGCTGATCTTCTTTGGGGTTGGCCTCATTGTCCACAAGAGGAGTCAGAAAGGTGA

>Bupa_DAB_3_partial_exon_3-4

CTGAGCCTGAGTTGACTGTATATCCATCAAAGATGGCTCTCCTGGGACACCACAACCTGCTTGTCTGCTCTGTCACTGGTTTCTATCCTGGGGACATTGAGGTCAGATGGTTCCTCAATGGGCAGGAGGAGACAGAAGGAGTTGTTTCTACAGGCCTGATCAGCAATGGAGATTGGACCTACCAGACCCTGGTGATGCTGGAAATGATCCCAAAGCATGGAGATGTCTACACCTGCCAAGTGGAGCACTCCAACCTTCAGAGACCTGTCATCTTGGACTGGAAAGCACAGTCTGAATCTGCCCAGAATACGATGCTGACTGGAGTTGGGGGCCTTGTGCTGGGCCTGATCTTCTTTGGGGTTGGTCTCATTGTCTACTTGAGGAGTCAGAAAGGTGA

>Bupa_DAB_4_partial_exon_3-4

TTGAGCCCGAGGTGACTGTGTATCCATCAAAGATAGCTGCCCCTGGAACACCACAACCTGCTTGTCTGCTCTGTCACTGGTTTCTATCCTGGGAACATTGAGGTCAGGTGGTTCCTGAATGGGCAGGAGGAGACAGCTGGGGTTGTGTCCACAGGCCTGATCAGCAATGGAGACTGGACCTACCAGACCCTGGTGATGCTGGAAATGACCCCCAAGCGTGGAGATGTCTACACCTGCCAAGTGGAGCACCCCAGCCTTCAGAGACCTGTCATCTTGGACTGGAAAGCACAGTCTGAATCTGCCCAGAGTAAGATGCTGAGTGGAGTCGGGGGCCTTGTGCTGGGCCTGATCTTCTTTGGGGTTGGCCTCATTGTCCACAAGAGGAGTCAGAAAGGTGA

>Bupa_DAB_5_partial_exon_1

ATGGTGTGTGTGTTGCTCCCCAAGGACATCTGGATAGAACTGTTGGCTGTGACCCTGCTGGTGCTGAATTCCCAGGAGGCCACAGACAGACATGCCCCAA

>Bupa_DAB_6

ATGCTGTGTGTGTGGACAAAAATTTTGATGATGACCCTGCTGATGCTGAATTCCTTGGGGGCTGCAGACAGAGCACTTTATGGAGCAAATGAAGGCTGAGTGTCACTTTGTCAATGGGACTGAGCACGTGCGGTTTGTGGGGAGACTCATCTACAACCGCCAGGAGATCCTGCGCTTCGACAGTGACATGGGGGAGTTCGTGGCCTTGACGGAGCTGGGGCAGCCCATTGCAGAGCTAATGAACAGCCTGCTGGAGGCTCTGGAGCAAGCGCGGGCCCAGGTGGCCACGTGCAGGAACAACTATAGGTTGTTGGAGTCCTCCTGGATGCAGAGAAGGGGTGAGTTTGAGCCTGAATTGACTGTGTATACATCAAAGACAGCTCCCCTAGGACAACCAAACCAACTTGTCTGTTTTGTTACTGGTTTCTATCCTGGAGACATTGAGGTCAGGTGGTTCCTGAATGGCCAGGAGGAGACAGCTGGGGTTGTGTCCACAGGCCTGATCAGCAATGGAGACTGGACCTACCAGATCCTGGTGATGCTGGAAATGACTCCCAAGCGTGGAGATGTCTATACCTGCCAAGTGGAGCACTCCAGCCTCCAGAGACCTGTCATTGTGGTCTGGGAAGCACAGTCCACATCTGCCCAAGGAAAGATGCTGAGTGGAGTCGGGGTCCTTGTGCTAGGACTACTCTTCCTTGGGGTTGGCCTCACTGTTCACCTTAGGAGTGAGAAAGGTGA

>Bupa_DAB_7_partial_exon_2-4

AGCACTTCATGGAGCAGCGGAAGTACGAGTGCTACTTTGTGAACGGAACGGAGCACGTGCGGTATATGGAGAGACACATCTACAACAGGGAGGAGTATGTGCGCTTCGACAGCGACATGGGGGAGTACGTGGCAGTGACCGAGCTGGGGCGGCTGGAGGCTGAGTATTGGAATACCCGTAAGGAGCTCTTGGAGCTGAAACGGGCCGCAGTGGACACTTTCTGCAGACACAACTACGAGAGGTTCGAGCCCTTCTCAGTGCGGAGGCGCGGTGATTGAGCCTGAGGTGACTGTGTATCCATCAAAGATGGCTCCCCTGGGACACCACAACCTGCTCGTCTGCTCTGTCACTGGTTTCTATCCTGGGGACATTGAGGTCAGGTGGTTCCTGAATGGGCAGGAGGAGACAGCTGGGGTTGTGTCCACAGGCCTGATCAGCAATGGAGACTGGACCTACCAGACCCTGGTGATGCTGGAAATGACCCCCAAGCATGGAGATGTCTATACCTGCCAAGTGGAGCACTCCAGCCTTCAGAGACCTATCATCTTGGACTGGAAAGCACAGTCTGAATCTTCCCAGAGTACGATGCTAAGTGAAATTAGGAGCCTTGTGCTGGGGGTGATCTTCTTTGGGGTTGGCCTCATTGTCCACATGAGAAGTAAGAAAGGTGA

>Bupa_DBB_1

ATGGTTGATGTTTGGATCTCTGATGACTACTGGAAGATTAGTCTGTTAACGACATTGATGGTGTTGAGTCTACCTGCATCCTGGGCTAGGGACATCCCAGCAGCTATGAGTCTTGGGATGCTTGGCTTTCGTGTTAGATACATCATACCAATGACCAGGAATATGTCCGCTTCGACAGCGACGTGGGGAACGCCTAACCCACGTGTGCGTGCTGTGACGGAGCTGAGGGCGGCCCAGAAAAAACTGCTGAGTATTGGAACAGTCAGAAGGACTTATTGATCCTGGAATAAGGAAAAACGAGCGCAACGTGGACCATGGCGTGTGACTCAGGTATCACAACTACGAGGTTGGCAAGCCCTTCACGTTGGACAGAAGAGTCCAGCCCAGAGTGACCATCTCCCCCTCCAGGACAGAGGCCCTGCAGCACCTGCTGGTCTGCTCTGTCACTGGCTTCTATCCAAGCAAGGTCAAGGTCACCTGGTTCAAGAATGGGCAGGAGGAGACAGCTGGGGTTGTGTCCACGGGTGTGATACAACATGGAGACTGGACCTACCAGACCGTGGTCATGTTGGAAATGACCCCCCAGAGCAGAGATGTCTACACCTGCAGCGTGGAACATACCAGCCTAGAGAACCCCATCAATGTGGAATGGAGGGCACAGTCTGAATCTGCCCAGAGCAAATTGCTGAGTGGAATTGGAGGCTTTGTCCTGGGGCTGATCTTCCTCAGTGTAGGGCTGATCATCCACCTGAAGAACCAGAAAGGTGA

>Bupa_DCB_1

ATGGTGTGTATGGAATTTCTAGGAGGCCCAGTATAGTCCTATTGATAGTGCTGAGCACACCTACAGCCTGAGGCAGACATTGCAGGTAAAGAATTACCTACAACAGGTGAGGTCCGAGTGTCACATGACCAATGGAACCCAACGAATGCATTTTGTGGGAAGACTCATCTATGACCAGGCAGAGTTTGTGCGTTTTGACAGTGATGTGGGACTATCTGAAGCAAGAATGGAGCTGTGGAAATCCCAAGTCCAGAAATGGAACAGTCAGAAGGAGACAGTCAAGTGTGCAAGGTTCATAGTGAATATGTGCAGACACAACTATCTTTTATATGATAAATTCATAGTGCAAAGTAAAACTACTTTTGTAAAGAGCCTGTGCAATGAATCCATTTGTAATTAGAAAAGTTGCCTGCTAATTCCCTTCAGTATCATTGAATGCTAATGAGCCAGAACTGGGATAATTCAGCACCATGGACTAAATGGACTTAAATGGCTACTCAGCTGCCTATGTCTGACTCAAGTCCAGTTTAGACCCTAGTGATGCTGGAAATGACTCCCAGGCATAAGGATATCTATACCTACCATGTAAATCATTCCAGCATTCAGAGACCTGTCATCCTAGACTGGAAAGCATAGTCTGAATCTGCCCAGAGTAAGATGCTGAGACATATGGAGGCCTTGTGCTGGGGCTGATCTTCCTTGGGGTTCACCTCATTGTCCACCTGAAGAGTCAGAAAAGTG

>Bupa_DMB_1

ATGAGGTTCCTCCACCTGCTACTAGTGGGCTTCAGCCTGGGCTTTCCAGGAGCAGCAGGGGGCTTTGTGACCCATGTGGAGAGTGTCTGTATACTGGATGAAGAAGGATCAGTAAAGGACTTCAGCTATTGTATCTCCTTCAACAAGGATGTGTTGACCTGCTGGGACTCAGAGACTAACAAGATGGCCACTGCTGATTTTGGAATACTGCATCTGTTAGCTGAGCAGCTTTCTAATGCCCTCAATAAAGATGATGCTTTGATAAACCGCCTGAGCAAAGGATTCCAGGACTGTGCCAGTCACACAAAGCCCTTCTGGGGATCATTGACCCAACGGACGCGGCCACCATCAGTGCAAGTAGCCCAGACCACACCATTTAATACAAGAGAGCCAGTGATGCTGGCCTGTTATGTATGGGGCTTCTATCCTGCTGATGTGGCTGTTTCATGGCTGAAGAATGGGCAGCCTGTCCCACACAGTGGCATCCAGAAGGCAGCACAATCCAATGGGGACTGGACTTATCAGACACGCTCCTACTTGGCCCTTACCCCCTCTAGTGGGGATATTTACACTTGCCATGTAGAGCACAGTGGGACTTCCCAGACCATCTTACAGACCTGGACACCTGGCTTCTCTTTGAAGCAGATAGTGAAGATTTCTGTATCTGTACTGACTCTGAGCCTTGGCCTTGTCTTCTTCTTCCTTGGCCTGGTTGCCTGCCGAAAAGCTGGCTCCTCTGACTACACTCCTCTCTCGGGGTCCAATTATCCTGCAGGTAA

>Daha_DNAzoo_DAB_1

ATGGTGTGTGTCTTGCTCTTCAAGGGCATCTGGACAGAAGTTCTGGCTGTGACCCTGCTGGTGCTGAATCCCCAGGTGGCTGCAGGCAGAAACACCCCAAAACACTTCACAAAACAGTCAAAGTGCGAATGTTACTTTGTGAATGGGATGGAGCATGTGCAGTATGTGGAGAGACACATGTACAACCAGAAGGAATATGTGCTCTTTGACAGCAATGTGGGGAAGTATGTTGCAGTGATGGAGCTGGGCCGACCAGAGGCTGAATACTGGAACAACCATAAGGAGATTCTAGATGACTTACAGGCCCGGGTGGACACTTTGTGCAGACACAACTACCAGGTTATTGAGCCCTTCTTGTTGCCCAGGAGTGTTGAGCCTGAAGTGATTGTGTATCCATCAAAGATGGCTCCCTTGGGACACCACAACCTGCTTGTCTGCTCTGTCAGTGGTTTCTATCCTGGAGACATCGAGGTCCAGTGGTTCCTGAATGGGCAGGAAGAGACGGCTGGGGTGGTGTCCACAGGCTTAATCAGCAATGGGGACTGGACCTATCAGTTACAGGTGATACTGGAAATGATCCCTAAGAGTGGAGATGTCTACACCTGCCAAGTGGAGCACTCCAGCCTTCAGAGACCCATCATCTTGGACTGGAAAGCCCAGTCTGAATCTGCCCAGAGAAAGATGCTGAGTGGAGTTGGGGGCATCATTCTGGGTTTGATCTTCTTTGGAGTTGGTCTCATTGTCCACAAGAGAAGTCGGAAAGGTGA

>Daha_DNAzoo_DMB_1

ATGAAGTTACTGCATCTACTGCTAGTAGGCTTCAGCCTGGGTTTTTCTGGAGCAGGGGCTTTTGTGACCCACGTGGAGAGTGACTGTGTACTGGATGAGGATGGATCAGTAAAGGACTTCACATATTGTATCTCCTTCAACAAGAATGTATTGACCTGCTGGGACTCAAAGACTAAAAAGATGGTCACTGTTGATTATGGTCTACTGCAGCCATTTGCTGAATATCTTTCTCAATCCCTTAATAACAACAGTGCCTTGATACACCACCTGAGCAATGGATTCCAGGATTGTGCCAGTCACACAAAACCTTTCTGGGGGTCATTGACTCAAAGAACACGGTCACCATCAGTGCAAATAGCTCAGACCACACCATTCAACACAAGGGAGCAAGTGATGTTGGCCTGCTATGTATGGGGCTTCTATCCTGCTGATGTGGCCATTTCATGGTTGAAGAATGGGCAGCCAATCCCTAACAGTGGCATTCAGAGAGCTGTACAGTCTAATGGGGACTGGACTTACCAGACACGATCCTATTTGGCCCTTACCCCCTCTAGTGGGGATATTTATACTTGCCATGTAGAGCACAGTGGGAGTTCCCAGGCCATCTTACAGACCTGGACCTCTGGCCTCTCTCTGAAGCAGACCGTGAAGATCTCTGTATCTGTATTGACTCTGGGACTTGGCCTCATCTTCTTCTTCCTTGGCCTGGTTTTCTGCCAAAAAGCAGGCTCCTCTGACTACACTCCTCTCTCGGGGTCCAATTATCCTGAAGGTAA

>Myfa_DNAzoo_DAB_1_partial_exon_3-4

TTCAGCCCGAGGTGACTGTGTATCCATCAAAGATGGCTCCCCTGGGACACCACAACCTGCTTGTGTGCTCCGTCAGCGGTTTCTATCCTGGGGACATCGAGGTCCGGTGGTTCCTGAATGGGCAGGAGGAGACGGCTGGATTGGTGTCTACAGGCCTGATCAGCAATGGAGACTGGACCTACCAGATCCTGGTGATGCTGGAGATGATCCCCAAGCATGGAGATGTCTACACCTGCCAAGTGGAGCACACCAGCCTTCAGAGACCTATCCTCTTGACCTGGAAAGCCCAGTCTGAATCTGCCCAGAGTAAGATGCTGAGTGGAGTTGGAGGCCTGATGCTGGGGCTGATCTTCTTTGGGGTCGGCCTCATTGTCTACAAGAGGAGCCAGAAAGGTGA

>Myfa_DNAzoo_DAB_2_partial_exon_3-4

TTGAGCCTGAGGTGATTGTGTATCCATCAAAGATGGCTCCCCTGGGATACCATAACTTGCTTGTCTGCTCTGTCAGTGGTTTCTATCCTGGGGACATCGAGGTCCGGTGGTTCTTGAATGGGCAGGAAAAGATGGTTGGGGTGGTGTCCACAGGTCTGATCAGCAATGGAGACTGGACCTACCAGATACAAGTGATGCTGGAAATGACCCCCAAGTGTGGAGATGTCTACACCTGCCAAGTGGAGCACACCAGCCTTCAGAGACCCATCCTCTTGGACTGGAAAGCACAGTCCGAATCTGCCCAGAGAAAAATGATGAGTGGAGTTGGGTGCATCATTGTAGGTTTGATCATCTTTGGGGTTGGCCTCATTGTCCACAAGAGAAGTCAGAAAGGTGA

>Myfa_DNAzoo_DAB_3_partial_exon_3-4

TTCAGCCCGAGGTGACTGTGTATCCATCAAAGATGGCTCCCCTGGGACACCACAACCTGCTCGTGTGCTCCGTCAGCGGTTTCTATCCTGGGGACATCGAGGTCCGGTGGTTCCTGAATGGGCAGGAGGAGACGGCTGGGGTGGTGTCCACAGGCCTGATCGGCAATGGAGACTGGACCTACCAGATCCTGGTGATGCTGGAGATGACCCCCAAGCATGGAGATGTCTACACCTGCCAAGTGGAGCACACCAGCCTTCAGAGCCCCGTCCTCTTGGACTGGAAAGCCCAGTCTGAATCTGCCCAGAGTAAGATGCTGAGTGGAGTCGGGGGCCTGGTGCTGGGCCTGATCTTCTTTGGGGTCGGACTCATTGTCCACAAGAGGAGCCAGAAAGGTGA

>Myfa_DNAzoo_DMB_1_partial_exon_2-5

GGGCTTTTGTGACCCATGTGGAGAGTGACTGTGTGCTGGATGAAGATGGATCAGTAAAGGACTTCACATATTGTATCTCTTTCAACAAGGAGATGTTGACCTGTTGGGACTCAGAGACTAAAAAGATGGTCACTATTGATTATGGGATACTGCATCCATTAGCTGACCAGCTTTCTCAATTCCTTAATAACAACAGTGCCTTGATATACCACCTGAGCAATGGACTCCAGGATTGTGCTAGTCACACAAAACCCTTCTGGGGGTCGTTGACTCGAAGGACACGGTCGCCATCAGTGCAAATAGCCCAGACCACACCATTCAACACAAGAGAGCCAGTGATGTTGGCCTGCTATGTATGGGGCTTCTATCCTGCTGATGTGGCCATTTCATGGTTGAAGAATGGGCAGCCAATCCCTTACAGTGGCATCCAGAGGGCTGTTCAGTCCAATGGGGACTGGACTTACCAGACACGATCCTACTTGGCTCTTACCCCCTCTAGTGGGGATATTTACACTTGCCATGTAGAGCACAGTGGGACTTCCCAGCCCATCTTACAGACCTGGACGTCTGGCCTCTCTCTGAAGCAGACTGTGAAGGTCTCTGTATCTGTATTGACTCTGGGACTTGGCTTCATCTTCTTTTTCCTTGGTCTGGTTTTCTGCCAAAAAGCAGGCTCCTCTGACTACACTCCGCTCTCAGGGTTCAATTATCATGAAGGTAA

>Myfa_DAB_1

ATGGTGTGTGTCTTGCTCCCCAAGGAAGTCTGGATACAAGTTCTGGCTGTGACCCTGCTGGTGCTGACTTCCCAGGTGGCTGCAGGCAGACACAGCCCAGGGCACTTCACGGAACAGGCAAAGGTCGAGTGTCACTTCGAGAACGGCACGGAGCACGTGCGCCTTTTGGAGAGATACTTCTACAACCGGGAGGAGTATGTGCGCTTCGACAGCGACGTGGGGAAGTTTGTGGCGGTGACGGAGCTGGGGCGGCCAGATGCTGAGTATTGGAACAGCCAGAAGGACATCCTGGAGCAGAAACGGGCCGCGGTGGACACTTACTGCCGGCACAACTACGAGGTGTCTCGGCCCTTCTTAGTGCGCAGGAGCGTTCAGCCCGAGGTGACTGTGTATCCATCAAAGATGGCTCCCCTGGGACACCACAACCTGCTCGTGTGCTCCGTCAGCGGTTTCTATCCTGGGGACATCGAGGTCCGGTGGTTCCTGAATGGGCAGGAGGAGACGGCTGGGGTGGTGTCCACAGGCCTGATCGGCAATGGAGACTGGACCTACCAGATCCTGGTGATGCTGGAGATGACGCCCCAGCGGGGAGATGTCTACACCTGCCAAGTGGAGCACTCCAGCCTTCAGAGCCCCATCCTCTTGGACTGGAAAGCCCAGTCTGAATCTGCCCAGAGTAAGATGCTGAGTGGAGTCGGGGGCCTGGTGCTGGGCCTGATCTTCTTTGGGGTTGGACTCATTGTCCACAAGAGGAGCCAGAAAGGTGA

>Myfa_DAB_2_partial_exon_2-4

AGCACTTCATGCTGCAGTTTAAGGCTGAATGTTACTTTGAGAACGGGACGGAGCACGTGAGGCATGTGCACAGAGACATCTACAACCGGGAGGAGATTGTGCGCTTCGACAGCGACGTGGGGAAGTTTGTGGCGGTGACGGAGCTGGGGCGGCCAGATGCTGAGTATTGGAACAGCCAGAAGGATTACATGGAGAATTTACGGGCGGAGGTAGACACTGTGTGTAGGTACAACTACAAGTTGGATTACCCCTTGTCAGTGCACATGCATGTTCAGCCCGAGGTGACTGTGTATCCATCAAAGATGGCTCCCCTGGGACACCACAACATGCTTGTCTGCTCCGTCAGCGGTTTCTATCCTGGGGACATCGAGGTCCGGTGGTTCCTGAATGGGCAGGAGGAGACGGCTGGATTGGTGTCTACAGGCCTGATCAGCAATGGAGACTGGACCTACCAGATCCTGGTGATGCTGGAGATGATCCCCAAGCATGGAGATGTCTACACCTGCCAAGTGGAGCACACCAGCCTTCAGAGACCTATCCTCTTGACCTGGAAAGCCCAGTCTGAATCTGCCCAGAGTAAGATGCTGAGTGGAGTTGGAGGCCTGATGCTGGGGCTGATCTTCTTTGGGGTCGGCCTCATTGTCTACAAGAGGAGCCAGAAAGGTGA

>Myfa_DAB_3_partial_exon_2-4

AGCACTTCATGCTGCAGTTTAAGGCTGAATGTTACTTTGAGAACGGGACGGAGCACGTGAGGCATGTGCACAGAGTCATCTACAACCAGGAGGAGTATGTGCGCTTCGACAGCGACGTGGGGAAGTTTGTGGCGGTGACGGAGCTGGGGCGGCCAGATGCTGAGTATTGGAACAGCCAGAAGGATTACATGGAGAATTTACGGGCGGAGGTAGACACTGTGTGTAGGTACAACTACAAGTTGGATTACCCCTTGTCAGTGCACATGCATGTTCAGCCCGAGGTGACTGTGTATCCATCAAAGATGGCTCCCCTGGGACACCACAACATGCTTGTCTGCTCCGTCAGCGGTTTCTATCCTGGGGACATCGAGGTCCGGTGGTTCCTGAATGGGCAGGAGGAGACGGCTGGATTGGTGTCTACAGGCCTGATCAGCAATGGAGACTGGACCTACCAGATCCTGGTGATGCTGGAGATGATCCCCAAGCATGGAGATGTCTACACCTGCCAAGTGGAGCACACCAGCCTTCAGAGCCCCGTCCTCTTGACCTGGAAAGCCCAGTCTGAATCTGCCCAGAGTAAGATGCTGAGTGGAGTTGGAGGCCTGATGCTGGGGCTGATCTTCTTTGGGGTCGGCCTCATTGTCTACAAGAGGAGCCAGAAAGGTGA

>Myfa_DAB_4

ATGGTGTGTCTTGCTCACCAGGGCTATCTGGACAGAGTTTCTATCTATGACCCTCCTGATGTTGAATTCTCAGGTGGTTGCAGGCAGACACAGCCCAGTGGGGGTGACCGTCACGGCTGACCCGCCCCTGGGTGATCCCTCAGAGCACTAAGTCCGAGTGCTACTTCGAGAATGGGATGGAGCACGTACGGTTTGTGCTCAGATACATCTACAACCGGGAGAAATATGTGCATTTCGACAGCGAAGTTGGGGTGTATGTGGCGGTGACGGAGTTGGGGCGGCCGGTGGCTGAGGATTGGAACAGCCAGAAGGAGCGCCTGGAGCGCGCATGGTCTGTGGTGGACTGGTTATGCAGCTGCTGGTACAATTCAGCCCGAGGTGACTGTGTATCCATCAAAGATGGCTCCCCTGGGACACCACAACCTGCTCGTGTGCTCCGTCAGCGGTTTTTATCCTGGGGACATTGAGGTCCGGTGGTTCCTGAATGGGCAGGAGGAGACGGCTGGGGTGGTGTCCACAGGCCTGATCGGCAATGGAGACTGGACCTACCAGACCCTGGTGATGCTGGAAATGACCCCCAAGCATGGAGATGTCTACACCTGCCAAGTGGAGCACTCCAGCCTTCAGAGCCCCGTCCTCTTGGACTGGAAAGCTCAGTCTGAATCTGCCCAGAGTAAGATGCTGAGTAGAGTCGGGGGCCTGGTGCTGGGGCTGATCTTCTTTGGGGTCGGCCTCATTGTCCACAAGAGGAGCCAGAAAGGTAA

>Myfa_DBB_1

ATGGTTGATGTTTGGATTTCTGCTGGCTGTTGGAAGATTGGTCTGGTAATGACATTGATGATGTTGATACTTTTATCTTGGGCCAGGGAAATCCCAGGTAAAGGATTTCATGTACCAGTTCCAGGGTGACTGTTACTTTACTAACAGCACAGAATAAGTGCCAGATACTTCTACCATGATCAGGAATTTTCTCCACTTGAATTGCTATATGGAAAAGTTTGTGACTGTGACAGATCTGGGGTAGCCAGATACTGAATATTGGAACAGTCAGAAGGAAATTATGAAGGAAGACTGAGCTTCTGTGGACACTAAGCATCCACAACTACAAGGTATACAAGCCTTTCTTATTGAAGAGAAGTGGTCTAGCCCAGAGTGACCATCTCTCCTTTCAAGACAGAGGCCCTTATATCCCAAAGAGATCTTAAAGGAGGGAAAGGGACCCACATGTGCAAGAATGTTTGTGGCTGCCCTTTTTGTAGTGGCAAGAAACTGGAAACTGAACGGATGCCCATCAATTGGAGAGTGGCTGAATAAATTATGGTATATGAACGTTACGGAATATTATTGTTATGTAAGAAATGACCAGCAGGACGATTTCAGAGAGGCCTGGAGAGACTTGCATGAACTGATGCTGAGGGCACAATTTGACTCTACCCAGAACAAATTGCTAAGTGGAATTGGAGACTCTGTCCTGGGGCTGATCTTTCACCTGAAAACCCAAAAAGGTAAAGAGCTGGGGCCAAAGGTAG

>Psco_DNAzoo_DAB_1

ATGCTGTGTGTGTGGACTAAAGTTCTGATGATGACCCTGCTGGTGCTGAATTCCCTGGTGGTTGCAGGCAGAGACAGCCCAAAGCACTTTATGGAGCAGATGAAGGCCGAGTGTCACTTTGTCAATGGGACTGAGCATGTGCGATTTGTGGGGAGACTCATCTACAACAGCCAGGAGATTCTGCGTTTCGACAGCAACTTGGGGAAGTTTGTGGCCTTGACGGAGCTGGGGCGGCCCATTGCAGAGCTAATGAACAGCCTGCTGGAGGCCCTGGAGCAAGCGCGGGCCCAGGTGGCCTGGTGCAGAGACAACTATAGGTTGTTGGAGTCCTGGATGCAGAGGAGGGGTGATTGAGCCTGAAGTAACTGTGTATCCATCAAAGATGGCTCCCCTAGGATACCCAAACCAGCTTGTCTGTTTTGTTACTGGTTTCTATCCTGGGGACATTGAGGTCAAGTGGTTCTTGAATGATCAGGAGGAGACAGCTGGAGTTGTGTCCACAGGCCTGATTAGCAATGGAGACTTGACCTACCAGATCCTGGTGATGCTGGAAATGACCCCTAAGCGTGGAGATATCTACACCTGCCAAGTAAAGCATTCCAGCCTTCAGAATCCTGTCATTGTGGTCTGGGAAGCACAGTCCACATCTGCCCAAGGAAAGATGCTGAGTGGAATTGGAAGCCTTGTGCTAGGGCTAATCTTCCTGGGGATTGGCCTTGCTGTTCACCTTAGGAGTCAGAGAGGTGA

>Psco_DNAzoo_DAB_2

ATGATGTGTATCTTGCTCCTCAGGGGTGTCTGGATAGAGGCTCTGGTTGTGACTCTGCTGGTGCTGAATTCCCAGGTGGCTGCAGGCAGACATGCTCAAGAGCACTTCACGGAGCAGGTGAAGGGCGAATGTTACTTTGAGAACAGGACGGAGCACGTGCGGTTTGTGCTGAGAGCCATCTACAACCGGGAGGAGTACGTGCGCTTCGACAGCGACGTGGGGGAGTTTGTGGCGGTGACCGAGCTGGGGCGGCTCACTGCGGAGTATGGGAACTCCCAGAAGGAGTTTATGGACCACTTACGGACTGCGGTGGACAGTTATTGCATGTACAACTACGAGAGAATCGAGCCCTTCTCAGTACCCAGGAGGAAGGTGATTGTGTATCCATCAAAGATGGCTCCCTTGGGACACCACAACTTGCTTGTCTGTTCTGTCAGTGGTTTCTATCCTGGGGACATTGAGGTCAGGTGGTTCCTGAATGGGCAGGAGGAGACAGCTGGGGTTGTGTCCACAGGCCTGATCAGCAATGGAGACTGGACCTACCAGATCCTGGTGATGCTAGAAATGACCCCCAAGCGTGGAGATGTCTACACCTGCCAAGTGGAGCACTCCAGTCTTCAGAAACCTGTCATCTTTAACTGGAAAGCACAGTCTGGATCTGCCCAGAATAAGATGCTGAGTGGAGTCGGGGTCCTTGTGCTGGGTCTGATCGTCTTTGGGATTGGCCTCATTGTCCACAAGAGGAATCAGAAAGGTGA

>Psco_DNAzoo_DAB_3

ATGGTGTGTGTGTTGCTCCTCAGAGGCATCTGGACAGAGGTTCTGGCTGTGACCCTGTTGGTGCTGACTTTCCAAGTGACTGCAAGCAGACATGCCCCAGCACTTCGCCCAGCAGGCCAAGTGCGAGTGTTACTTTGAGAACGGGACGCAGCACGTGCGGTTTATGGTGAGACTCATCAACAACGGGGTGGAAAATGTGCGCTTCGACAGCGACATGGGGGAGTTTGTGGCGCTGACGGAGCTGGGGTGGTGCGATGCTGAGCTTTGGAACAGCCAGAAGGATTACCTGGAGGACGCACGGGCCACAGTGGACACTTTGTACAGATACAACCACAAGTTGTCTGAGCCCTTAGTGCGCAGGCGCGGTGATTGAGCCCAAGGTGATTGTGTATCCATCAAAGATGGCTCCCCTGGGACACCACAACATGCTTGTCTGCTCTGTCACTGGTTTCCTGGGGACATTGAAGTCAGGTGGTTCCTGAATGGGCAGGAAGAGACAGCTAGGGTTATGTCCACAGGCCTGATCAGCAATGGAGACTGGACCTACCAGATCCTGGTGATGCTGGAAATGACCCCCAAGCTTGGAGATGTCTACACCTGCCAAGTGGAGCACTCCAGCCTTCAAAGACCTGTCGTCTTGGACTGGAAAGCACAGTCTGAATCTGCCTAGAGTAAGATGCTGAGTGGAGTTGGGGGCCTGGTGCTGGGTCTGATCTTCTTTGGGGTTGGCGTCATTGTCTACAAGTGGAGGCAGAAAGGTGA

>Psco_DNAzoo_DAB_4

ATGGTGCATGTCTTGCTCCCCCAGGGCATCTGGACAGAGGTTCTAGCTGTGACTCTGCTGGTGCTGACTTCCCAGGTAGCTGCAGGCAGACATGCCCCAGAGCACTCTGTGGAGCAGGGAAAGATTGAGTGTTACCATGTGAACGGGACGCAGCACGTGCGTCTTGTGGATAGATACTTCTACAACCGAGAGGAATATGTGCGCTTCGACAGCAACGTGGGGGAGTTTGTGGCGGTGACGGAGCTGGGGCGGCCCGACGTTGAGTATTATAACAGCCAGAAGGAGCTCCTGGAGCGGAAACGGGCGAGGCGGACACGGTGTGCAGGCACAACTATGAGGTGCTGGAGCTCTTCTTAGTGCCCAAGCGCGGTGATTAAGCCTGAGGTGATTGTGTATCCATCAAAGATGGCGCCCCTGGGACACCACAACCTGTTTGTCTGCTCTGTTACTGGTTTCTATCCTGGGGACATTGAAGTCAGGTGGTTCCTGAATGGGCAGGAGGAGACAGCTGGGGTTGTGTCCACAGGCCTGATAAGCAATGGAGACTGGACCTACCAGATCCTGGTGATGCTGGAAATGACCCCCAGGCATGGAGATGTCTACACCTGCCAAGTGGAGCACTCCAGCCTTCAGAAACCTGTTGTCTTGGACTGAGGAGCACAGTCTGAATCTGCCCAGAGTAAGATGCTGAGTGGAGTCGGGGGCCTGGTGCTGGGGCTGATCTTTTTTGGGATTGGCCTCATTGTCCACAAGAGGAGTCAGAAAGGTGA

>Psco_DNAzoo_DAB_5_partial_exon_2-4

AGCACTTCACGGAGCTGGTTAAGGCCGAGTGTTACTTTGAGAACGGGACTGAGCAAGTGCGGTTTGCGGAGAGACACATCTACAACGGGGTGGAATTTATGCGCTTCGACAGCGACTTGGGGGAGTATGTGGCGGTGACAGAGCTGGGGCAGCACGAGGCTCAGTATAGGAACAGCCTGAAGGATAGACTGGAGTACGCAAGGGCAGCAGTGGACACTTTCTGCCGGCACAACTACAAGTGCGCTGAACCCTTCTCAGTGCACAGGCTCGTTAAGCCTGAGGTGATTGTGTATCCATCAAAGATGGCTCCCCTGGGACACCACAGCCTGCTTGTCTGCTCTGTCACTGGTTTCTATCCTGGGGACATTGAAGTCAGGTGGTTCCTGAATGGGCAGGAGGAGACAGCTGGGGTTGTGTCCACAGGCCTGATCAGCAATGGAGACTGGACCTACCAGATCCTGGTGATGCTGGAAATGACCCCCAGGCGTGGAGATGTCTACACCTGCCAAGTGGAGCACTCCAGCCTTCAGAGACCTGTCATCTTAGACTGGAGAGCACAGTCTGAATCTGCCCAGAGTAAGATGCTGAGTGGAGTTGGGGGCCTGGTGCTGGGGCTGATCTTCTTTGGGGTTGGCCTCATTGTCCACAAGAGGAGTCAGAAAGGTGA

>Psco_DNAzoo_DAB_6_partial_exon_2-4

AGCACTTCACGGAGCAGAGAAAGGCCGAGTGTCACTTTGTGAATGGGAGAGAGCACGTACGATATGTGCTGAGATGCATCCACAACCGGGAGGAGATTGTGCGCTTCGACAGCGCAGTGGGGGAGTTTGTCGCGCTGACCGAGCTGGGGCGGCCGGAGGCTGAGTATTGGAACAGCCAGAAGGAGATCCTGGAATACAGACGCGACCTGGTGAATACTTACTGCAGGCACAACTACCAGGGGATTGAATACTTTTCAAAGAGCAGGAGAGGTGACCTGAGGTGATTGTGTATCCATCAAAGATGGCTCCCCTGGGACACCACAACCTGCTTGTCTGCTCTGTCACTGGTTTCTATCCTGGGGACATTGAGATCAGGTGGTTCCTGAATGGACAGGAGGAGACAACAGGTGTTGTGTCCACAGGTCTGACCAGCAATGGAGACTGGACCTACCAGATCATGGTGATGCTGGAAATTACGCCTAAGCATGGAGATATCTACACCTGCCAAGTGGAGCACTCCAGCCTTCAGAAACCTGTTGTCTTGGACTGGAAAGCACAGTCTGAATCTGCCCAGAGTAAGATGCTGAGTGGAGTTGCTGTCCTCACATTGGGCCTGATCTTCTTTGGGGTTGGCCTCATTGTCCACAAGAGGAGTCAGAAAGGTGA

>Psco_DNAzoo_DAB_7_partial_exon_2-4

AGCACTTCATGGAGCAGCTGAAGTCCGAGTGTTACTTTGAGAACAGGACGGAGCACGTGCGGTATGTGGAGAGACTCATCTACAACCGGGAGGAGTCCGCGCTACGACAGCCACGTGGGGGAATATGTGGCGGTGACAGAGCTGGGGCGGCTGAGGCAGAGTACTGGAACATCCAGAAAGAGCTCCTCGAGTACAAACGCGGCCAGGTGGACAATTACTGCCGGCACAGCTATGAGGAGATTGAGCCCTTAGTGCGCAGGCGCGGTAATAGAGCCCGATGTGATTGTGTAACCATCAAAGATGGTTCCCCTGGGACACCACAACCTGCTTGTCTGCTCTGTCACTGGTTTCTATCCTGAGGACATTGAGGTCAGGTGGTTCCTGAATGGGCAGGAGGAGACAGCTGGGGTTGTGTCCACAGGCCTGATCAGCAATGGAGACTGGACCTACCAGATCCTGGTGATGCTGGAAATGACCCCCAAGTGTGGAGATGTCTACAACTGCCAAGTGGAGCACTCCAGCCTTCAGAGACCTGTCATCTTGGACTGGAAACACAGTTGCCCAGAGTAAGATGCTGAGTGAAATCAGGAGCCTTGTACTGGGGCTGATCTTTGGAGTTGGCCTCATTGTCCACATAATGAGTAAAAGAGGTGA

>Psco_DNAzoo_DAB_8_partial_exon_2-4

AGCACTTCATGGAGCAGCTGAAGTCGGAGTGTTACTTTGAGAACGGGACGGAGCACGTGCGCTGTGTGGAGAGACTCATCTCCAACCGGGAGGAGTCCGCGCGCTACGACAGCCACGTGGGGGAGTACGTGGCGGTGACGGAGCTGGGGCGTCCCGAGGCAGAGTATTGGAACAGCCAGAAGGAGCCCCGGGAGTACAAACGCGGCCAGGTGGACAATTACTGCCGGCACAGCTATGAGGAGATTGAGCCCTTACTGCGCAGGCGCGTTGAACCCGAGGTGATTGTGTATCCATCAAAGATGGCTCCCCTGGGACACCGCAACCTGCTTGTCTGCTCTGTCACTGGTTTCTATCCTGGGGACATTGAGGTCAGGTGGTTCCTGAAAGGGCAGGAGGAGACAACTGGAGTTGTGTCCACAGGCCTGATCAGCAATGGAGATTGGACCTACCAGATCCTGGTGATGCTAGAAATGGCTCCAAGTGTGGAGATGTCTACACCTGCCAAGTGGAGCACTCCAGCCTTCAGGAACCTGTCCTCTTGGACTGGAAAGCTCAGTCTGAATCTGCCCAGAGTAAGATGCTGAGTGAAATCAGGAGCCTTGTACTGGGGCTGATCTTCTTTGGAGTAGGCCTCATTGCCACATGATGAATAAGAGAGGTGA

>Psco_DNAzoo_DCB_1_partial_exon_2-5

GAGAATTACTTACATCAGGTGAGGTCTGAGTGTTTCATGACCAATGGAACCCAACAAGTGCACTTCGTGGGAAGACTCATCTACAACTGGGTAGAGTTCGTGTGCTTTGACAGTGACGTGGGGCTATTTGAGGTAAGAATGGAGCTGTGGAAATCCCAAGTCCAGAAATGAAACAGTCAGAAGGAGATAGTTGAGCGTGCAAGGTCCATAGTGAATGTGTGCAGAGACAACTATCCTTATATGATACATAGTGCAAAGGAAAGTCCAGCCCTGAGTGAAGGTTTTCCCCTCAAAGGTATAACCACTTGGGCACCACAATCTGCTCCTCTGTTCCGTGACCAGTTTCTATCCTGGTGAGATCAAGATTAGCTGGTTCAGGAATGCAAAAGAGAAGACTGGGGTCATGTCCACAGGCCGAATCCAGAATGGTGACTGGACCTTCCATTCCTTGGGATGCTGGAAATGACCCCCCAAAGAGGAGATGTCTTTACTTGTCATGTGGACCATGTCAGCTTGCAGAGCCTTGTCCCCTTAGACTAGAGAGCACAGTCTGAATCTGCCCGGACTAAGATGCTGACTGGAATTGGGGGCCTGGTGCTTGGACTGTTTTTACTTGGAGTGGAACTTGTCATCCACCTCAGAAATTTGAAAGATTCCTGTTCTGGGACCAAAGAAGATTCAAATTTGGAAAGGATTGTGAACTTTGCAAGAAGATTTTCCCAGAGCTGTTGCCCAGTCCTAA

>Psco_DNAzoo_DMB_1

ATGAGGTTACTCCACCTGTTACTAGTGGGCTTCAGTCTGAGTTTTTCAGGAGCAGGAGGCTTTGTGACCCATGTGGAGAGTGGCTGTATGCTGGATGAAGAAGGATCAGTAAAGGACTTCACATATTGTATCTCCTTCAACAAGGATGTGTTGACCTGCTGGAACTCAGAGACTAACAAGATGACCACTGTTGATTTTGGAATTCTATATCCATTAGCTGAGCAGCTTTCTGGAGCCCTCAATAATGATAGTGCTTTTATAGACCACCTGAGCAAAGGACTACAGGACTGTGCTAGTCACACGAAGCCCTTCTGGGGATCACTGACCCAAAGGACATGGCCACCATCAGTGCAAGTAGCCCAGACTACACCATTTAACACAAGGGAGCCAGTGATGCTGGCCTGTTATGTATGGGGCTTCTATCCTGCTGATGTGGCCATTTCATGGTTGAAGAATGGGCAGCCTATCCCACACAGTGGCATCCAGAAGGCTGTACAATCCAATGGGGACTGGACTTACCAGACACGATCCTACTTGGCCCTTACCCCCTCTAGTGGGGATATTTACTCTTGTCATGTAGAGCACAGTGGGGCTTCCCAGATCATCTCACAGACCTGGACACCTGGCCTCTCTCTTAAGCAGACAATGAAGATCTCTGTATCTGTATTGACTCTGAGCCTTGGCCTCATCTTCTTCCTCCTTGGCCTGGTTGCCTGCCGAAAAGCTGGCTCCTCTGATTACACTCCTCTCTTGGGGTCCAATTATCCTGAAGGTAA

>Sebr_DNAzoo_DAB_1

ATGCTGTGTGTCTTACTCCCCCAAGGTATCTGGACAGAGGTTCTGGCTGTGATCCTGCTCGTGCCAAATTCCCAGATGGCTGCAGGCAGGCATGCCTCAGGTAAAGCACTTCATGGAGCAGAAAAAGGTGGAGTGTCACTTTGTGAAGGGACGCAGCACGTGCGGTTTGTGGAGAGACACATCTAAACCGGGAGAAGTTTGGGCGCTTCGACAGCGCCGCTGGGGAGTTTGTGGCGGTGTCGGAGCTGGGGTGGCCGGGTGCGGAGAGTTGGAACAGCGGGAAGGAGCTCCCGGAGCAGGAACGCGCCCGAGTCACACTTTCCGCAGGCACACCTACAAGGTGATCGAGCCCTTCTCAGTGCGCAGGCCTGGGGATTGAGCCCGAGGTGATTGTGTATCTATCAAAGCTGGCTCCCCTGGGACACCACAACCTGCTTGTCTGCTCTGTCACTGGTTTCTGTCCTGGGGACATTGAGGTCAGGTGGTTCCTGAATGGGCAGGAGGAGACAGCTGGGGTTGTGTCCACAGGCTTGGTCAGCAATGGAGACTGGACTTACCAGATCTTGGTGATGCTGGAAATGACCCCCAAGCATGGTGATGTCTACACCTGCCAAGTGGAGCATTCCAGCCTTCAGAAACCTGTCATCTTGGACTGGAAAGCACAGTCTGAATCTGCCCAGAGTAATATGCTGCGTGAACTCAGGGGCCTTGTATTGGGACTGATCTTCTTTGAGGTTGACCTCATGGTCTACATAAGGAGTAAGAAAGGTGA

>Sebr_DNAzoo_DAB_2

ATGGTGTGTGTCTTGTTCCCCCAGGGTATCTGGATGGAGGTTCTGACACTGCTGGTGCTGAATGCTCAGGTGACTGTAGGCAGACATGCCCCAGGTAAAGCACTTCACGGTGCAGGTGAAGGGCGAATGTTACTTTGTGAATGGCACTGAGCACGTGCGGTTCGTGATGAGGGGCATCTACAACCGGGAGGAGTACGTGCGCTTCGACAGCGACGTGGGGGAGTTTGTGGCCATGACGCCGCTGGGGCGCCTCACTGCGGAGTTTGGTAACAGCCAGAAGGAGTTCATGGACCACTTACGGGAGGCGGTGGACACTTACTGCAGGCACAACTACGAGGGGATCGAGATCTTCACAGTGCGCAGGAGCGGTGATTCAGCCTGAGGTGACTGTGTATCCATCAAAGCTGGCTCCCCTGGGACACCACAACCTGCTTGTCTGCTTTGTCACTGGTTTCTATCCTGGGGACATTGAGGTCAAGTGGTTCCTGAATGGGCAAGAGGAGACAGCTGGGGTTGTGTCCACAGGCCTGATCAGCAATGGAGACTGGACTTACCAGATCCTGGTGATGCTGGAAATGACCCCTAAGCATGGAGATATCTACACCTGCCAAGTGGAGCACTCCAGCCTTCGAAGACCTATTATTTTTGACTGGAAAGCACAGTCTGAATCTGCCCAGAGTAAGATGCTGAGTGGAGTTGGGGTTCTTGTGCTGGGGTTGATCTTCTTTGGGGTTGGCCTCATCGTCCACAAGAGGAGTCAGAAAGGTGA

>Sebr_DNAzoo_DAB_3

ATGGCATGTGTCTTGCTTCGCAGAGGCATCTGGACAGAGGTTCTGACTGTGACTCTGCTGGTGCTGAATTCCCAGGTGACTGCAGACAGACATGCCCCAGAACACTTCATGGAGCAGAGAAAGGTCGAGTGTCACTTTGTGAATGGGACAGAACACGTGCGGTATGTACTGAGATGCATCCATAATAGGGAGGAGATCCTGCGCTTCGACAGTGATGTGGGGAAGTTTGTGGCACTGACAGAGCTGGGCCGGCCTGAGGTTGAGTATTGGAACAGCCAGAAGAACATCCTGAAGTATAGACAAGATCAAGTGAACACTTACTGCAGGCCCAACTACCAGGCTGTTAAAGTCTTTTCACAGAGCAGGAACGGTGACTGAACCTGAGGTGATTGTGTATCCATCAAAAATGGCTCCCTTGGGATATCACAACCTGCTTGTCTGCTCTGCCACTGGTTTCTATCCTGGAGACATTGAGGTCAAGTGGTTCCTGAATGGGCAAGAGGAGACAGCTGGGGTTGTGTCCACAGGCCTGATCAGCAATGGAGACTGGACCTACCAGATCTTGGTGATGCTAGAAATGACCCCCAAGCATGGAGATGTCTACACCTGCCAAGTGAAGCACTCTAGCCTTCAGAGACCTGTCACCTTGGACTGGAAAGCACACTCAGAATCTGCCCAGAGTAAGATGCTGAGTGGAGTTGGGAGCCTCTTGCTGGGCCTGATCTTCTTGGGGGTTGGCCTCATTGTCCACAAGAGGAGTCAGAAAGGTGA

>Sebr_DNAzoo_DAB_4

ATGCTGTGTGTCTTCCTTTCCAGAGGCATCTGGACAGAGGTTCTGGCTATGACCCTGCTGGTGCTGTATTCCCAGGAGGGTGCAGGCAGAGATGCACCAGAGCACTTCACTGAGCAGATGAAGTCCGAATGTTACTTTGCGAACGGGACGCAGCACGTGCGGTTTGTGGAGAGATACATCTACAACCGGGAGGAGTTTCTGCGCTTTGACAGCGACGTCGGGGAGTATGTGGCGCTGACGGAGCTGGGGCGGGGGACTGCTGAGCACTGGAACAACCAAAAGGATTACATGAAGAGCAGACGGGGCATGGTAGACACTTTGTGCAGACCCAACTATAGGGTGTCTGAGCCCTTCTTAGTCCCCAGGCGCGGTGATTCAGCCTGAAGTGATTGTGTATCCTTCAAAGATGGCTCCCCTGGGACACCACAACTTGCTTATCTGCTTTGTCACTGGTTTCTGTCCTGGGAACATTGAGGTCAGGTGGTTCCTGAATGGGGAGGAGAAGACAGCTGGGGTTGTGTCCACAGGCCTGATCAGCAATGGAGACTGGACTTACCAGATCTTGGTGATGCTGGAAATGACCCCCAATCGTGGTGATGTCTACACCTGCCAAGTGGAGCACTCCAGCCTTCAAAAACCTGTCATCTTAGACTGGAAAGCACAGTCTGAATCTGCCCAGAGTAAGATGCTGAGTGGAGTTGGGAGCCTCATTCTGGGGCTGATCTTCTTTGGAATTGGCCTCATTGTCCACAAGAGGAGTCAGAAAGGTGA

>Sebr_DNAzoo_DAB_5

ATGCTCTGTGTCTCCCTCTCCAGAGTCATCTGGATGGAGGTCCTGGCTGTGACCCTGCTGGCACTGAATTCCCAGGTGGCTGCAGGCAGACATGTCCCAGAGCACTTCGTGTTGCAGGGCAAGTCCGAGTGTCACTTTGTGAACGGGACGCAGCACGTGCGGTTTATGGACAGATACTTCTACAACCGGGAGGAGATCGTGCGCTTCGACAGCGCCGTGGGGGAGTTTGTGGCGCTGACGGAGCTGGGGCGGGGGATTGCTGAGTATTCTAACAGCCAGAAGGATTACCTGGAGCGCACCCGGACCGCAGTGGACAGCTACTGCAGATACAACTACTGGGTGATTGAGCCCTTCTTATTCCCCAGGCGCGGTGACTGAGCCTGAGGTGATTGTGTATCCATCAAAGCTGGCTCCCTTGGGACACCACATCCTGCTTGTCTGTTCTGTCACTGGTTTCTATCCTGGGGACATTGAGGTCAGGTGGTTCCTGAATGGGCAGGAGAAGACAGCTGGGGTTGTGTCCACAGGCCTGGTCAGCAATGGAGACTGGACTTACCAGATGTTGGTGATGCTGGAAATGACCCCCAAGCGTGGTGATGTCTACACCTGCCAAGTGGAGCACTCCAGCCTTCAGAAACCTGTCATCTTGGACTGGAAAGCACAGTCTGAATCTGCCCAGAGTAAGATGCTGAGTGGAGTCGGGGGGATCATGCTGGGGCTGATCTTCTTTGGGGTTGGCCTCATTGTCCACAAGAGGAGTCAGAAAGGTGA

>Sebr_DNAzoo_DAB_6_partial_exon_2-4

AGCACGTCATGGAGCAGGTGAAGGCAGAGTGTCACTTTGTGAAGGGGGCGGAGCACGTGCGGCTGGTGCAGAGATACATCCACCACCCCCAGGACTATGCTCGCTTCCATGCTAAGGGGAGGGAGGGGGGCAGTTTGTGAGGGTCACGGAGCTGGGGCGCCGGGAGCCTGGGGATTTGAACCGCCGGAAGGAGATCCTGGAGGACGAACGAGCCCGGTGGACACTTTCTGCAGGCACAGCTGCAAGGGGTCTGAGCGCTTCTTGGTGCACAGGCAGGGTTGAGCCTGAGGTGATTGTGTATCCATCAAAGATGACTCCCCTGGGACCCCACAATCTGCTTGTCTGCTCTGGCACTGGTTTCTATCCTGTGAAAAGTGAGGTCAGGTGGTTCCTGAATGGGCAGGAGGAGACAGCTGGGGTCATGTCCACAGGCCTTATTAGTCATGGAGACTGGATCTGATGCTGGAAATGACCCCCAAGCATGGAGATATCTACACCTGCCAAGTGGAGCTCTCTAGCCTTCAGAAACCTGTCATTTTGGACTGGAAAGCAGTCTAAATCTGCCCAGAGTAAGATGCCCAGTGGAGTCGACGGCCTCGTGCTGGGGCTGATGTTCTTTGGGGTTGGCCTCATTGTCCACAAGAGGAGTCAGAAAGATGA

>Sebr_DNAzoo_DBB_1

ATGGTTGATGTTTGGATCTCTGCTGGCTGCTGGAAGACTCGTCTGTTAATGACATCAATGCTGTTGAGTTTATCTACATCTTGGGCCAGGGACATCCCAGAGGATTTCGTGTATCAGTACAAGGGTGAGTGTTACTTCACCAACGGCTCGGAGCGGGTGAGGTTTGTGTTTAGATCCATCTACAATGGCGAGGAGGATGTCCGCTTCGACAGCGACGTGGGGCACTTCGTGGCCCTGACGGAGCTGGGGCGGCCCGATGCTGAGTACTGGAACGGTCAGAAGGAGAGCCTGGAGGAATACCGAGGCTACGTGGACACGGTGTGCAGACACAACTACGAGGCATATAAGCCCTTCACGTTGGACAGAAGAGTCCAGCCCAGAGTGACCATCTCCCCCTCCAAGACAGACGCCCTGCAGCACCTGCTGGTCTGCTCTGTCACTGGCTTCTATCCAAGCAAGATCAAGGTCACCTGGTTCAAGAATGGGCAGGAGGAGACAGCTGGGATCGTGTCCACGGGTGTCATACAACATGGAGACTGGACCTACCAGACCCTGGTCATGTTGGAAATGACTCCCCAGAGCAGAGATGTCTACACCTGCAGTGTGGAGCATGCCAGCCTGCAGAGCCCCATCAGTGTGGAATGGAGGGCACAGTCTGAATCTGCCCAGAGCAAACTGCTGAGTGGAATTGGAGGCTTTGTCCTGGGGCTGATCTTCCTCAGTGTAGGATCGATCATCCACCTGAAGAACAAGAAAGGTGA

>Sebr_DNAzoo_DBB_2

ATGCTTGATGTTTGGATCTTTGCTGGTTACTGGAAGATTGGTCTGTTAATGACTTCGATGCTGTTCAATCTGTCTGCATCTTGGGCCAGGGACATCCCATAGAATTTCGTGTATCAGAACAAGGCTGAGTGTTATTTCACCAACGGCATGGAACGGGTGTGCTTTGTGGTTAGATTGTCTACGATGACCAGAAATTTCTCCGCTTTGACAGAGACGTGCGGAAGTTCCTGGCGGAGGGAGCTGGGGCGGAGTCAGGCTGAGTACTGGGACAGTCTGGAGGAGATGCTGGAGCAAAATCAAGCCGCCGTAGACACGCTGTGCAGATACAACTATGACATTGGCAAGCCGTTCATGGTGGACAGAAGAGTCCAGCCCAGAGTGACCATCTCCCCCTCCAAGACAGACGCCCTGCAGCACCTGCTGGTCTGCTCTGTCACTGGCTTCTATCCAAGCAAGATCAAGGTCACCTGGTTCAAGAATGGGCAGGAGGAGACAGCTGGGATCGTGTCCACGGGTGTGATACAAAACGGAGACTGGACCTACCAGACCCTGGTCGTGTTGGAAATGACTCCCCAGAGCAGAGATGTCTACACCTGCAGTGTGGAGCATGCCAGCCTGCAGAGCCCCATCAGTGTGGAATGGAGGGCACAGTCTGAATCTGCCCAGAGCAAACTGCTGAGTGGAATTGGAGGCTTTGTCCTGGGGCTGATGTTCCTCAGTGTAGGACTGATCATCCACCTGAAGACCAAGAAAGGTGA

>Sebr_DNAzoo_DCB_1

ATGGAACATGTGGAGTTTCTGGGAAGCCCTGTATAACAGTCATACTGAGAGTGCTGAGCACACCCACAGACTGAGGTAGGGACATTCCAAGTAAAGAATTACCTACATCAGATGGGGTCTGAGGGTCACATGATCAATGGAACCCAACAAGACTCATCTACAACCAGATGGAGTTTGTGCACTTTGACAGTGACGTGGGACTCTTTCAGACAAGCTGTGGAAATCCCAAGTCCAGAAATGAAACAGTAAGGAAGAGAAAGTCAAGCATGCAAGGTCCATAATGAATGTGTTCAGAGACAGTTTTCTTTTATATGACAAATTCACAGTGTAAAGGAAAGCCCCTAGTGAAAGTTTTACCCCCAAAGATACAACCACCTGGGCACCACAACTTGCTCCCCTGTTCTGTGATGTTTCTATCCTGGTGAGATCAAGATCAATTGATTCAGGATCACAAAAAAAGAGAAGACTGGAATCCTGTCCACAGATCGAATTCAGCACAGTGACTGGACCTTCCAAACCCTTGTGATGCTAGAAATGGCCCCCCAAAGAGGAGATGTCTTTACTTTCCATGTGGACCATGTCAGCTTGCAGAGTCCTGTCATTGTAAACTGGAGAGCACATTCTGAATCTTCCTGGACTGAGAGGCTCACTGGAATTCAGGGCTTGGTATTTGGACTGATCTTACTTGGAGTGAGGCTTACTTGTCATCCACCTCAGAAATTTGAAAGATTCCTATTCTGGTACCAAAGAAGACCCAGATTTTGAAGGGATTGTGAACACTGCCTCATTGCAACAAGATTTTCCCAGAACCATTGCCCAGCCCTAG

>Sebr_DNAzoo_DMB_1

ATGACATTACTCTACCTGCTACTAGTGGGCTTCAGCCTGGGTTCAGGGGGCTTTGTGACTCATGTGGAGAGTGACTGTATACTGGATGAAGAAGGATCTGTAAAGGACTTCGAATATTGCATCTCCTTCAACAAGGATGTGTTGACCTGCTGGGACTCAGATATTAGCAAGATGGTCACTGTTGATTATGGAATACTGCATCCATTAGCTGACTGGCTTTCTGGCACCCTCAATAATGATACTGCTTTGATAAACCGCCTGAGCGAAGGATTCCAAAACTGTGCCAGTCACACAAAGCCCTTCTGGGGATCACTGACCCACAGGACACGGCCACCATCAGTGCAAGTAGCTCAGGTCACACCATTCAACACAAGGGAGCCAGTGATGCTGGCCTGTTATGTATGGGGCTTCTATCCTGCTGATGTGGCCATTTCGTGGTTGAAGAATGGGCAGCCTGTCCCACACAGTGGCATCCAGAAGGCTGTACAATCCAATGGGGACTGGACTTATCAGACACGATCCTACTTGGCCCTTACCCCCTCTAGTGGGGATATTTACACTTGCTTTGTAGAGCACGGTGGGACTTCCCAGACCATCTTACAGACCTGGACACCTGGCCTCTCCCTGAAGCAAATAGTGAAGATCTCTGTATCTGTACTGACTCTGAGCTTTGGTCTTATCTTCTTCTTCCTTGGCCTGGTTGCACGTAGAAAAGCTGGCTCCTCTGACTATTCTCCTCTCTCGGGGTCCAATTATCCTGAAGGTAA

>maru_DNAzoo_DAB_1

ATGCTGTGTGTCTTGCTCCCCAGAGACATCTGGACAGAGGTTCTGGCTATGACCCTGCTGGTGCTGACTTCCCAGGTGGCTGCAGGCAGACATGCCCCAAAACCTTTTACGAAGCAGGGGAAGTATGAGTGTCACTTTGTGAATCAGACACAGCACGTGCGGTACGCGGAGAGACTCGTCTACAACCGACAGCGACGTCGGGTAGTACGTGGCGCTGATGGAGCTGGGGCAGCCAAGTGCTGAGAGTTTGAACAAACGGAAGGAGCTGCTGCAGCAGAGACGGGCTTTGGTGGACACTTTCTGCAGGCACAACTACGAGATACTTGAGCGCTTCTTAGTCCCCAGGCGCGGTGATTGAGCCAGAGGTGATTGTGTATCCATCAAAGTTGGCTCCCCTGGGACAGCACAACCTGCTTGTCTGCTCTGTCACTGGTTTCTATCCTGGGGACATTGAGGTCAGGTGGTTCCTGAATGGGCAGGAGGAGACAGCTGGGGTTGTGTCCACAGGCCTGGTCAGCAATGGAGACTGGACTTACCAGATCTTGGTGATGCTGGAAATGACCCTCAAGCGTGGTGATGTCTACACCTGCCAAGTGGAGCACTCCAGCCTTCAGAAACCTGTCATCTTGGACTGGAAAGCACAGTCTGAATCTGCCCAGAGTAAGATGCTGAGTGGAGTCGGGGGCCTTGTGCTGGGGCTGATCTTCTTTGGGGTTGGCCTCATTGTCTACAAGAGGAATCAGAAAGGTGA

>maru_DNAzoo_DAB_2

ATGCTGTGTGTCTTCCTTTCCAGAGGCATCTGGACAGAGGTTCTGGCTATGACCCTGCTGGTGCTGTATTCCCAGGAGGGTGAAGGCAGAGATGCACCAGAGCACTTCAGTGAGCAGATGAAGTCCGAGTGTTACTTTGTGAACGGGACGCAGCACGTGCGGTTTGTGGAGAGATACATCTACAACCGGGAGGAGTTTGTGCGCTTCGACAGCGCCGTGGGGGAGTATGTGGCGCTGACCGAGCTGGGGCGGGGGACTGCTGAGCATTGGAACAGCCAGAAGGATTACATGGAGAGCAGACGGGGCATGGTGGACACTTTGTGCAGACCCAACTACAGGGTGTCTGAGCCCTTCTTACTCCCCAGGCGCGGTGATTCAGCCCGAAGTGATTGTGTATCCTTCAAAGATGGCTCCCCTGGGACACCACAACTTGCTTATCTGCTTTGTCACTGGTTTCTATCCTGGGAACACTGAGGTCAGGTGGTTCCTGAATGGGCAGGAGAAGACAGCTGGGGTTGTGTCCACAGGCCTGGTCAGCAATGGAGACTGGACTTACCAGATCTTGGTGATGCTGGAAATGACCCCCAATCGTGGTGATGTCTACACCTGCCAAGTGGAGCACTCCAGCCTTCAAAAACCTGTCATCTTAGACTGGAAAGCACAGTCTGAATCTGCCCAGAGTAAGATGCTGAGTGGAGTTGGGAGCCTCATTCTGGGGCTGATCTTCTTTGGAATTGGCCTCATTGTCCACAAGAGGAGTCAGAAAGGTGA

>maru_DNAzoo_DAB_3

ATGGCATGTGTCTTGCTTTGCAGAGGCACCTGGACAGAGGTTCTGACTGTGACTCTGCTGGTGCTGAATTCCCAGGTGACTGCAGACAGACATGCCCCAGAACACTTCATGGAGCAGAGAAAGGTCGAGTGTCACTTTGTGAATGGGACAGAGCACATGCGGTATGTACTGAGATGCATCCATAATAGGGAGGAGATCCTGCGCTTCGACAGTGATGTGGGGAAGTTTGTGGCACTGACAGAGCTGGGCCGGCCTGAGGTTGAGTACTGGAACAGCCAGAAGAACATCCTGAAGTATAGACAAGATCAAGTGAACACTTACTGCAGGCCCAACTACCAGGCTGTTAAAGTCTTTTCACAGAGCAGGAACGGTGACTGAACCTGAGGTGATTGTGTATCCATCAAAAATGGCTCCCCTGGGATATCACAACCTGCTTGTCTGCTCTGCCACTGGTTTCTATCCTGGAGACATTGAGGTCAAGTGGTTCCTGAATGGGCAAGAGGAGACAGCTGGGGTTGTGTCCACAGGCCTGATCAGCAATGGAGACTGGACCTACCAGATCTTGGTGATGCTAGAAATGACCCCCAAGCATGGAGATGTCTACACCTGCCAAGTGAAGCACTCTAGCCTTCAGAGACCTGTCACCTTGGACTGGAAAGCACACTCAAAATCTGCCCAGAGTAAGATGCTGAGTGGAGCTGGGAGCCTCTTGCTGGGCCTGATCTTCTTGGGGGTTGGCCTCATTGTCCACAAGAGGAGTCAGAAAGGTGA

>maru_DNAzoo_DAB_4

ATGGTGTGTGTCTTGTTCCGCCAGGGTATCTGGATGGAGGTTCTGACATTGCTGGTGCTGAATGCTCAGGTGACTGTAGGCAGACATGCCCCAGGTAAAGCACTTCACGGTGCAGGTGAAGGGCGAATGTTACTTTATGAACGGCACTGAGCACGTGCAGTTTGTGATGAGGGGCATCTACAACCGGGAGGAGTACGTGCGCTTCGACAGCAACGTGGGGGAGTTTGTGGCGATGACGCCGCTGGGGCGCCTCACGGCGGAGTTTGGTAACAGCCAGAAGGAGTTCATGGACCACTTACGGGAGGCGGTGGACACTTACTGCAGGCACAACTACGAGGGGATCGAGCTCTTCACAGTGCCCAGGAGCGGTGATTCAGCCTGAGGTGACTGTGTATCCATCAAAGCTGGCTCCCCTGGGACACCACAACCTGCTTGTCTGCTCTGTCACTGGTTTCTATCCTGGGGACATTGAGGTCAAGTGGTTTCTGAATGGGCAAGAGGAGACAGCTGGGGTTGTGTCCACAGGCCTGATCAGCAATGGAGACTGGACTTATCAGATCCTGGTGATGCTGGAAATGACCCCCAAGCATGGAGATATCTACACCTGCCAAGTGGAGCACTCCAGCCTTCGGAGACCTATTATTTTTGACTGGAAAGCACAGTCTGAATCTGCCCAGAGTAAGATGCTGAGTGGAGTTGGGGTTCTTGTGCTGGGGTTGATCTTCTTTGGGGTTGGCCTCATCATCCACAAGAGGAGTCAGAAAGGTGA

>maru_DNAzoo_DAB_5

ATGCTGTGTCTTACTCCCCCCGGGTATCTGGACAGAGGTTCGGGCTGTGATCCTGCTCATCCGAATTCCTGGATGGCTGCAGGCAGGCATGCCTCAGGTAACACTTCATGGAGCAGAAAAGGCCGAGTGTGACTTTGTGAACGGGACGCAGCACGTGCGGTATGTGGAGAGACACATCTAAACCGGGAGGAGTTTGGGCGCTTTGAGGGCGACGCGGGGGTTTGTGGCGGTGTCGGAGCTGGGGTGGCGCGGTGCGCAGAGTTGGAAGGAGCTCCTGGAGCAGAAACGCGCCCGAGTGGACACTTTCTGCAGGCACACCTACAAGGTGATCGAGCCCTTCTCAGTGCTCAGGCCCGGGGATTGAACCTGAGGTGATTGTGTATCCATCAAAGCTGGCTCCCCTGGGACACCACAACCTGCTTGTCTGCTCTGTCACTGGTTTCTATCCTGGGGACGTTGAGGTCAGGTGGTTCCTGAATGGGCAGGAGGAGACAGCTGGGGTTGTGTCCACAGGCCTGGTCAGCAATGGAGACTGGACTTACCAGATGTTGGTGATGCTGGAAATGACCCCCAAGCATGGTGATGTCTACACCTGCCAAGTGGAGCACTCCAGCCTTCAGAAACCTGTCATCTTGGACTGGAAAGCACAGTCTGAATCTGCCCAAAGTAATATGCTGCATGAATTCAGGAGCCTTATATTGGGACTGATCTTCTTTGGGGTTGACCTCATGGTCTACATAAGGAGTGAGAAAGGTGA

>maru_DNAzoo_DBB_1

ATGGTTGATGTTCGGATCTCTGCTGGCTGCTGGAAGATTGGTCTGTTAATGACATCAATGCTGTGGAGTTTATCTGCATCGTGGGCCAGGGACATCCCAGAGGATTTCGTGTATCAGTACAAGGGCGAGTGTTACTTCACCAACGGCACGGAGCGGGTGCGGTTTGTGTTTAGAGACATGTACAATGGCGAGGAGGATGTCCGCTTCGACAGCGACGTGGGGCACTTCGTGGCCGTGAGGGAGCTGGGGCGGCCCGATGCTGAGTACTGGAACAGTCAGGAGGATTCCCTGGAGGAATATCGAGGCTACGTGGACACGGTGTGCAGACACAACTACGAGGTATACAAGCCCTTCACGGTGGACAGAAGAGTCCAGCCCAGAGTGACCATCTCCCCCTCCAAGACAGACGCCCTGCAGCACCTGCTGGTCTGCTCTGTCACTGGCTTCTATCCAAGCAAGATCAAGGTCACCTGGTTCAAGAATGGGCAGGAGGAGACAGCTGGGATCGTGTCCACGGGTGTGATACAACATGGAGACTGGACCTACCAGACCCTGGTCATGTTGGAAATGACTCCCCAGAGCAGAGACGTCTACACCTGCAGTGTGGAGCATGCCAGCCTACAGAGCCCCATCAGTGTGGAATGGAGGGCACAGTCTGAATCTGCCCAGAGCAAACTGCTGAGTGGAATTGGAGGCTTTGTCCTGGGGCTGATCTTCCTCAGTGTAGGATCGATCATCCACCTGAAGAACAAGAAAGGTGA

>maru_DNAzoo_DCB_1

ATGGAACATGTGGAGCTTCTGGGAGGCCCTGTATGACAGTCATACTGATAGTGCTGAGCACACCCACAGACTGAGGTAGGGACATTCCAAATAAAGAATTACCTACATCAGATGGGGTCTGAGGGTCACATGATCAATGGAACCCAACAAGACTCATCTACAACCAAGTGGAGTTTGTGCACTTTGATAGTGATGTGGGACTATTTCAGACAAGCTGTGGAAATCCCAAGTCCAGAAATGAAACAGTAAAAAGGAGAAAGTCAAGCATGCAAGGTCCATAATGAATGTGTGCAGAGACAATTTTCTTTTATATGACAAATTCACACCCCTAGTGAAAGTTTTACTCCCAAAGATACAACCACCTGGACACCACAACCTGCTCCCCTGTTCTGTGATGTTTCTATCCTGGTGAGATCAAGATCAATTGATTCAGGATCACAAAAGAAGAGAAGACTGGAATCCTGTCCACAGATTGAATTCAGCACAGTGACTGGCCCTTCCAAACCCTTGTGATGCTAGAAATGGCCCCCCAAAGAGGAGATGTCTTTACTTGCCCTGTGGACCATGTCAGCTTGCAGAGCCCTGTCACTGTAAACTGGACACATTCTGAATCTTCCTGGACTAAGAGGCTGACTGGAATTCAGGGCTTGGTATTTGGACTGATCTTATTTGGAACTTGTCATCCACCTCAGAAATTTGAAAGATTCCTATTCTGGTACCAAAGAAGACCCAGATTTTGAAGGGATTGTGAACATTGCCTCATTGCAACAAGATTTTCCCAGAACTGTTGCCCAGTCCTAG

>maru_DNAzoo_DMB_1

ATGACATTACTCTACCTGCTACTAGTGGGCTTCAGCCTGGGTTTTTCAGGAACAGGGGGCTTTGTGACTCATGTGGAGAGTGACTGTATACTGGATGAAGAAGGATCCGTAAAGGACTTCCAATATTGCATCTCCTTCAACAAGGATGTGTTGACCTGCTGGGACTCAGATACTAGCAAGATGGCCTCTGTTGAATTTGGAATACTGCATCGATTAGCTGACTGGCTTTCTGACATCCTCAATAATGATACTACTTTGATAAACCGCCTGAGCGAAGGATTCCAAAACTGTGCCAGTCACACAAAGCCCTTCTGGGGATCACTGACCCACAGGACACGGCCACCATCAGTGCAAGTAGCTCAGGTCACACCATTCAACACAAGGGAGCCAGTGATGCTGGCCTGTTATGTATGGGGCTTCTATCCTGCTGATGTGGCCATTTCGTGGTTGAAGAATGGGCAGCCTGTCCCACACAGTGGCACCCAGAAGGCTGTACAATCCAATGGTGACTGGACTTATCAGACACGATCCTACTTGGCCCTTACCCCCTCTAGTGGGGATATTTACACTTGCTTTGTAGAGCATGGTGGGACTTCCCAGACCATCTTACAGACCTGGACACCTGGCCTCTCCCTGAAGCAAATAGTGAAGATCTCTGTATCTGTACTGACTCTGAGCTTTGGTCTTATCTTCTTCTTGCTTGGCCTGGTTGCACGTAGAAAAGCTGGCTCCTCTGACTACTCTCCTCTTTCGGGGTCCAATTATCCTGAAGGTAA

>Maeu_DNAzoo_DAB_1

ATGATGTGTGTGTGTAACTCTCCCAGGGTATGTGGACAGAGATTCTGGCTGTGACCCTGCTGGTGCTGAATTCCCAGGTGGCTGCAGGTAACATGCTCCAGAGCACGTCATGGAGCAGGTGAAGGCGGAGTGTCACTTTGTGAAGGGGACGGAGCACGTGCGGCTTGTGCAGAGATACATCCACCACTCCCAGGACTACAAGGGCTTCCATACTAAGGGGATGGAGGGGGCAGTTTGTGACGGTCACGGAGCTGGGGCGCCGGGAGCCTGAGGATTTGAACCGCCGGAAGGAGATCCTGGAGGACGAACCGGCCGACTGGACACTTTTTGCAGGCACAACTACAAGGAGTCTGAGAGCTTCTTGGTGCACAGGCAGGGTGAGTATGGATTGTGTATCCATCAAAGATGGCTCCCCTGGGACACCACAACCTGCTTGTCTGCTCTGTCCCTGGATTCTATCCTGTGAAAAGTGAGGTCAGGTGGTTCCTGAATGGGCAGGAGGAGAAAGCTGGGGTCATGTCCACAGCCTTATCAGTAATGGAGACTGGATCTATCAGATCCTAGTGATGCTGGAAATGACCCCCAAGCATGGAGATGTCTATACCTGCCAAGTGGAGCACTCTAGCCTTGAGAAAACTGTCATTTTGGACTGGAAAGCAGTCTGAATCTGCCCAGAGTAAGATGCCCAGTGGAGTCGAGGGCCTCATGCTGGGTTGGCCTCATTGTCTACAAGAGGAATCAGAAAGATGA

>Maeu_DNAzoo_DAB_2

ATGGCATGTGTCTTGCTTCGCAGAGGCATCTGGACAGAGGTTCTGACTGTGACTCTGCTGGTGCTAAATTCCCAGGTGACTGCAGACAGACATACCCCAGAACACTTCATGGAGCAGAGAAAGGTCGAGTGTCACTTTGTGAATGGGACAGAGCACGTGCGGTATGTACTGAGATGCATCCATAATAGGGAGGAGATCCTGCGCTTCGACAGTGATGTGGGGAAGTTTGTGGCACTGACAGAGCTGGGCCGGCCTGAGGCTGAGTATTGGAACAGCCAGAAGAACATCCTGAAGTATAGACAAGATCAAGTGAACACTTACTGCAGGCCCAACTACCAGGCTGTTAAAGTCTTTTCACAGAGCAGGAACGGTGACTGAACCTGAGGTGATTGTGTATCCATCAAAAATGGCTCCCCTGGGATATCACAACCTGCTTGTCTGCTCTGCCACTGGTTTCTATCCTGGAGACATTGAGGTCAAGTGGTTCCTGAATGGGCAAGAGGAGACAGCTGGGGTTGTGTCCACAGGCCTGATCAGCAATGGAGACTGGACCTACCAGATCTTGGTGATGCTAGAAATGACCCCCAAGCACGGAGATGTCTACACCTGCCAAGTGAAGCACTCTAGCCTTCAGAGACCTGTCACCTTGGACTGGAAAGCACACTCAAAATCTGCCCAGAGTAAGATGCTGAGTGGAGTTGGGAGCCTCTTGCTGGGCCTGATCTTCTTGGGGGTTGGCCTCATTGTCCACAAGAGGAGTCAGAAAGGTGA

>Maeu_DNAzoo_DAB_3

ATGGTGTGTGTCTTCTTCCCCCAGGGTATCTGGATGGAGGTTCTGACACTGCTGGTGCTGAATGCTCAGGTGACTGTAGGCAGACATGCCCCAGGTAAAGCACTTCACGGTGCAGGTGAAGGGCGAATGTTACTTTGTGAACGGCACTGAGCACGTGCGGTTTGTGATGAGGGGCATCTACAACCGGGAGGAGTATGTGCGCTTCGACAGCGACGTGGGGGAGTTTGTGGCAATGACGCCGCTGGGGCGCCTCACGGCGGAGTTTGGTAACAGCCAGAAGGAGTTCATGGACCACTTACGGGAGGCGGTGGACACTTACTGCAGGCACAACTACGAGGGGATCGAGCTCTTCACAGTGCCCAGGAGCGGTGACTGAGGTGACTATGTATCCATCAAAGCTGGCTCCCCTGGGACACCACAACCTGCTTGTCTGCTCTGTCACTGGTTTCTATCCTGGGGACATTGAGGTCAAGTGGTTCCTGAATGGGCAAGAGGAGACAGCTGGGGTTGTGTCCACAGGCTTGATCAGCAATGGAGACTGGACTTACCAGATCCTGGTGATGCTGGAAATGACCCCCAAGCATGGAGATATCTATACCTGCCAAGTGGAGCACTCCAGCCTTCGGAGACCTATTATTTTTGACTGGAAAGCACAGTCTGAATCTGCCCAGAGTAAGATGCTGAGTGGAGTTGGGGTTCTTGTGCTGGGGTTGATCTTCTTTGGGGTTGGCCTCATCATCCACAAGAGGAGTCAGAAAGGTGA

>Maeu_DNAzoo_DBB_1

ATGCTTGATGTTTGGATCTTTGCTGGTTACTGGAAGATTGGTCTGTTAATGACTTCAATGCTGTTCAGTCTTTCTGCATCTTGGGCCAGGGACATCCCACGAGAATTTCGTGTATCAGAACAAGGCTGAGTGTTATTTCACCCACGGCATGGAACAGGTGCGCTTTGTGGTTAGATAGTCTACGATGACCAGAAATTTCTCCGCTTTGACAGCAACGTGGGGAAGTTCCTGGAGGTGATGGAGCTGGGGCGGAGTCAGGCTGAGTACTGGAACAGTCTGGAGGAGATGCTGGAGCAAAATCAAGCAGCCGTAGACACGCTGTGCAGATACAGCTACGACATTGGCAAGCCTTTCATGGTGGACAGAAGAGTCCAGCCCAGAGTGACCATCTCCCCCTCCAAGACAGATGCCCTGCAGCACCTACTGGTCTGCTCTGTCACTGGCTTCTATCCAAGCAAGATCAAAGTCACCTGGTTCAAGAATGGGCAGGAGGAGACAGCTGGGGTTGTGTCCACGGGTGTGATACAACATGGAGACTGGACCTACCAGACCCTGGTCATGTTGGAAATGACTCCCCAGAGCAGAGATGTCTACATCAGCAGTGTGGAGCATGCCAGCCTACAGAGCCCCATCAGTGTGGAATGGAGGGCACAGTCTGAATCTGCCCAGAGCAAACTGCTGAGTGGAATTGGAGGCTATGTCCTGGGGCTGATCTTCCTCAGTGTAGGACTGATTATCCACCTGAAGAACAAGAAAGGTGA

>Maeu_DNAzoo_DBB_2

ATGGTTGATGTTTGGATCTCTGCTAGCTGCTGGAAGATTGGTCTCTTAATTACATCAATGCTGTTGAGTTTATCTGCATCTTGGGCCAGGGACATCCCAGAGGATTTCGTGTATCAGTACAAGGGCGAGTGTTACTTCACCAACGGCACGGAGCGGGTGCGGCTTGTGTTTAGATCCATATACAATGGCGAGGAGGATGCCCGCTTCGACAGCGACTTGAGGCACTTCGAGGCCGTGACGGAGCTGGGGCGGCCCGATGCTGAGTACTGGAATGGTCAGGAGGATTCCCTGGAGGAATACCGAGGCTACGTGGACACGCTGTGCAGACGCAACTACGAGGTAACTAAGCGGTTCACGGTGGACAGCAGAGTCCAGCCCAGAGTGACCATCTCCCCCTCCAAGACAGACGCCCTGCATCACCTGCTGGTCTGCTCTGTCACTGGCTTCTATCCAAGCAAGATCAAGGTCACCTGGTTCAAGAATGGGCAGGAGGAGACAGCTGGGGTTGTGTCCACGGGTGTGATACAACATGGAGACTGGACCTATCAGACCCTGGTCATGTTGGAAATGGCTCCCCAGAGCAGAGATGTCTACACCTGCAGTGTGGAGCATGCCAGCCTACAGAGCCCCATCAGTGTGGAATGGAGGGCACAGTCTGAATCTGCCCAGAGCAAACTGCTGAGTGGAATTGGAGGCTTTGTCATGGGGCTGATCTTCCTCAGCATAGGGCTGATCATCCACCTGAAGAACAAGAAAGGTGA

>Maeu_DNAzoo_DCB_1

ATGGAACATGTGGAGTTTCTGGGAGACCCTGTATGAAAGTCATACTGATAGTGCTGAGCACACCCACAGACTGAGGTAGGGACATTCCAAAGAATTACCTACATCAGATGAGGTCTGAGGGTCACATGATCAATGGAACCCAACAAGACTCATCTACAACCAGGTGGAGTTTGTGCTCTTTGATAGTGATGTGGGACTATTTCAGACAAGCTGTGGAAATCCCAAGTCCAGAAATGAAACAGTAAGAAGGAGATAGTCAAGCATGCAAGGTCCATAGTGAATGTGTTCAGAGACAATTTTCTTTTATATGACAAATTCACAGTCCAGCCTCTAGTGAAAGTTTTACCACCAAAGATACAACCACCTGGGCACCACAGCCTGCTCCCCTGTTCTGTGATGTTTCTATCCTGGTGAGATCAAGATCAATTGATTCAGGATCACAAAAGAAGAGAAGACTGGAATCCTGTCCACAGATTGAATTCAGCACAGTGACTGGACCTTCCAAACCCTTGTGATGCTAGAAATGGCCCCCCAAAGAGGAGATGTCTTTGCTTGCCATGTGGACCATGTCAGCTTGCAAAGCCCTGTCATTGTAAACTGGAGAGCACATTCTGAATCTTCCTGGACTAAGAGGCTCACTGGAATTCAGGGCTTGGTATTTGGACTGATCTTACTTTGAACTTGTCATCCACCTCAGAAATTTGAAAGTCTGGTACCAAAGAAGACCCAGATTTTGAAGGGATTGTGAACATTGCCTCATTGCAACAAGATTTTCCCAGAACTGTTGCCCAGTCCTAG

>Maeu_DNAzoo_DMB_1

ATGACATTACTCTACCTGCTACTAGTGGGCTTCAGCCTGGGTTTTTCAGGAACAGCAGGGGGCTTTGTGACTCATGTGGAGAGTGGCTGTATACTGGATGAAGAAGGATCCGTAAAGGACTTCGAATATTGCATCTCCTTCAACAAGGATGTGTTGACCTGCTGGGACTCAGATACTAGCAAGATGGTCACTGTTGAATTTGGAATACTGCGTCCATTAGCTGACTGGCTTTCTGACACCTTCAATAATGATACTGCTTTGATAAACCGCCTGAGTGAAGGATTCCAAAACTGTGCCAGTCACACAAAGCCCTTCTGGGGATCACTGACCCACAGGACACGGCCACCATCAGTGCAAGTAGCTCAGGTCACACCATTCAACACAAGGGAGCCAGTGATGCTGGCCTGTTATGTATGGGGCTTCTATCCTGCTGATGTGGCCATTTCGTGGTTGAAGAATGGGCAGCCTGTCCCACACAGTGGCATCCAGAAGGCTGTACAATCCAATGGGGACTGGACTTATCAGACACGATCCTACTTGGCCCTTACCCCCTCTAGTGGGGATATTTACACTTGCTTTGTAGAGCACGGTGGGACTTCCCAGACCATCTTACAGACCTGGACACCTGGCCTCTCCCTGAAGCAAATAGTGAAGATCTCTGTATCTGTACTGACTCTGAGCTTTGGTCTTATCTTCTTCTTCCTTGGCCTGGTTGCACGTAGAAAAGCTGCCTCCTCTGACTACTCTCCTCTCTCGGGGTCCAATTATCCTGAAGGTAA

>Saha_DAB_1

ATGGTGTGTGTCTTGCTCTTCAAGGGCATCTGGACAGAAGTTCTGGCTGTGACCCTGCTGGTGCTGAATCCCCAGGTGGCTGCAGGCAGAAACACCCCAAAACACTTCACAAAACAGTCAAAGTGCGAGTGTTACTTTGTGAATGGGATGGAGCATGTGCAGTATGTGGAGAGACACATGTACAACCAGAAGGAATATGTGCACTTTGACAGCAATGTGGGGAAGTATGCTGCAGTGATGGAGCTGGGCCGACCAGAGGCTGAATACTGGAACAACCATAAGGAGATTCTAGATGACTTACGGGCCCGGGTGGACACTTTGTGCAGACATAACTACCAGGTTATTGAGCCTTTCTTGTTGCCCAGGAGTGtTGAGCCTGAAGTGATTGTGTATCCATCAAAGATAGCTCCCTTGGGACACCACAACCTGCTTGTCTGCTCTGTTAGCGGTTTCTATCCTGGGGACATCGAGGTCCGGTGGTTCCTGAATGGGCAGGAAGAGATGACTGGGGTGGTGTCCACAGGCTTAATCAGCAATGGGGACTGGACCTATCAGTTACAGGTGATACTGGAAATGATCCCTAAGAGTGGAGATGTCTACACCTGCCAAGTGGAGCACTCCAGCCTTCAGAGACCCATCATCTTGGACTGGAAAGCCCAGTCTGAATCTGCCCAGAGAAACATGCTGAGTGGAGTTGGGGGCATCATTCTGGGTTTGATCTTCTTTGGAGTTGGTCTCATTGTCCACAAGAGAAGTTGGAAAGGTGA

>Saha_DAB_2

ATGGGGTGTGTCTTGCTCCCCAAGGAAGTCTGGATAGAAGTTCTGGCTGTGACCCTGCTGGTGCTGAATGCCCAAATGGCTGCAGGCAGACACAGCCCAGAGGACTTCATGGTGCGGGAAAAGTACGAGTGTCACTTTGAGAATGGGATGGAGCACGTGCGGTATGTGCACAGAGACTTCTACAACCGGGAGGAGATTACGCTCTTCGACAGCGATGTGGGGAAGTATGTGGCGCTGACGGAGCTGGGGCGGCCGGATGCTGAGTATTGGAACAGCCAGAAGGAGATCCTGGAGCGGAGACGGGCTTCGGTGGACACTTTGTGCAGACACAACTATGTGGGGATTGAGCCCTTCTCATTGCCGCAGGAGCGGTGAtCCAGCCCCAGGTGACTCTGTATCCATCAAAGATGGCTCCCCCAGGACACCACAACCTGCTCTTCTGCTCCATCAGCGGTTTCTATCCTGGGGACATTGAGGTCTGGTGGTTCCTGAATGGGCGGGAGGAGACGGCAGGGGTGGTGTCCACCGGCTTGATGGGCAATGGAGACTGGACCTAGCAGACACTGGTGATGCTGGAGATGACTCCCCAGCATGGAGATGTCTATACCTGCCACGTGAAGCACTCCAGCCTTCAGAGACCCATCATCTTGGACTGGAAAGCCCAGTCTGAGTCTGCCCAGAGTAAGATGCTGAGCAGAGTTGGGGGCCTTGTTCTGGGGCTGATCTTCTTGGGGGTTGGCCTCATTGTCCACAAGAGGAGCCAGAAAGGTGA

>Saha_DAB_3

ATGGGGTGTGTCTTGCTCCCCAAGGAAGCCTGAATAGAAGTTCTGGCTGTGACCCTATTGGTGCTGAATCCCCAGGTGGCTGCAGGCAGACACAGCCCAGAGCACTTCACCTTGCAGTTTAAAGCAGAGTGTTACTTCGAGAACGGGACGGAGCACGTGCGGCATGTGCACAGAGCCATCTACAACCGGCAGGAGTACGCGCGCTTCGACAGCGACTTGGGGAAGTTTGTGGCGGTGACGGAGCTGGGGCGGTGCAGTGCAGAGTATTGGAACAGCCAGAAGGAGATCCTGGAGAACGTGCGGGCGGAGGTGGACACTGTGTGCATATACAACTACAAGTCCTATAACTCCTTGTCAATGCATATGCACGCTCACCCCAAGGTGACTGTGTATCCATCCAAGATGGCTCCCCCGGGACACCACAACTTGCTTGTCTGCTCTGTCAGCGGTTTCTATCCTGGGGACATCAAGGTCCAGTGGTTCCTGAATGGGCGGGAGGAGACGGCAGGGGTGGTGTCCACGGACTGATAGGCAATGGAGACTGGACCTACCAGACGCTGGTGATGCTGGAGATGACCCACCGGCGTGGAGATGTCTACACCTGCCAAGTGGAGCACTCCAGCCTTCAGGGACCTGTCCACTTGGACTGGAAAGCCCAGTCTGAGTCTGCCCAGAGTAAGATGCTGAGTGGAGTCGGGGGCCTCGTGCTGGGGCTGATCTTCTTGGGGGTCGGCCTCATTGTCCACAAGAGGAGTCAGAAAGGTGA

>Saha_DAB_4

ATGGTGTGTGTCTTGCTCCCCAAGGAAGTCTGGATAGAAGTTCTGGCTGTGACCCTGCTGGTGCTGAATTCCCAGGTGGCTGCAGGCAGACATAGCCCAGAGCACTTCACGGAGCAGTTTAAGGGCGAGTGTTACTTCGAGAACGGGACGGAGCACGTGCGGCTTGTGGTGAGAACCATCTACAACCGGCAGGAGTTTATGCGCTTCGACAGCGACGTGGGGAAGTTTGTGGCGGTGACGGAGCTGGGGCGGCGCAGTGCTGAGCATTTGAACAGCCAGAAGGAGATCCTGGAGCGGAAACGGGCCGAGGTGGACACTGTGTGCAGGCACAACTACGGGGTGATTGAGCCCTTCTTAGTGCGCAGGCGCGGTGAtCCAGCCCGAGGTGACTGTGTATCCATCCAAGATGGCTCCCCTGGGACACCACAACCTGCTTGTCTGCTCCGTCAGCGGTTTCTATCCCGGGGACATCGAGGTCCGGTGGTTCCTGAATGGGCGGGAGGAGACGGCAGGGGTGGTGTCCACGGGCCTGATGGGCAATGGAGACTGGACCTACCAGATCCTGGTGATGCTGGAGATGACCCCCCGGCACGGAGATGTCTACACCTGCCACGTGGAGCACGCCAGCCTTCAGGAACCCGTCCTCTTGGACTGGAAAGCCCAGTCTGAGTCTGCCCAGAGTAAGATGCTGAGCGGAGTCGGGGGCCTCGTGCTGGGGCTGATCTTCTTTGGGGTCGGCCTCATTGTCCACAAGAGGAGCCAGAAAGGTGA

>Saha_DBB_1

ATGGTTGATGTTTGGATTTCTGCTGCCTCTTGCAAGATTGATCTGGTAATGACATCAGTGATGTCGATACCTTTATCTTGGGCCAAGGAAATCCcagAGGATTTCGTGTTCCAGTTCCAGGGTGACTGATACTTTACTAACAGCATGGAACAAGTGTCAGATACTTCTGCAATGGCCAGGAATTTTCTCTGCTTGGACTGCTATATGGGTAAGTGTGTGATTGTGATGGAGCTGGGGTGGCCAGATACTGAATACTGGAACACTCagaaggaaatcatgaaggaagaCCAAGCCTCTGTGGACACTATGCATCCACAactataagatatatatataagcCTTTCTCTTGGAGAGAATCCAGCCCAGAGTGACCATCTCTCCTTTCAAGACAGAGgacctaggCCTTGCAGCACCTGCTGCTCTGCTGTCTCACCAGCTTCTATCCTAAACAAGATCAAGGTCACCTGATTCAAGAATAGATCAGAGGAAACAGCTGGGATTGTGTCCATAAATCTGATACAGAATGGAGACTGGACCTACCAGACCCTTGTCATGTTGGAAATGATTCTCCATAGCAGAAATGTCTACACATACAGTGTGGACCATGCCAGTGCACAGAGCTTCATCAGTGTGTAATGTCTGAATCTACTCAGAGCAAATTGCTAAGTGGAATTGGAGGCTTTGTCCTGGAGCTGA

>Saha_DMB_1

ATGAAGTTACTGCACCTACTGCTAGTAGGCTTCAGCCTGGGTTTTTCTGGAGCAGGGGCTTTTGTGACCCACGTGGAGAGTGACTGTGTACTGGATGAGGATGGATCAGTAAAGGACTTCACATATTGTATCTCCTTCAACAAGAATGTGTTGACCTGCTGGGACTCAAAGATTAAAAAGATGGTCACTGTTGATTATGGTCTATTGCAGCCATTTGCTGAATATCTTTCTCAATCCCTTAATAACAACAGTGCCTTGATATACCACCTGAGCAATGGACTCCAGGATTGTGCCAGTCACACAAAACCTTTCTGGGGGTCATTGACCCAAAGAACACgGTCACCGTCAGTGCAAATAGCCCAGACCACACCATTCAACACAAGGGAGCAAGTGATGTTGGCCTGCTATGTATGGGGCTTCTATCCTGCTGATGTGGCCATTTCATGGTTGAAGAATGGGCAGCCAATCCCTAACAGTGGCATTCAGAGAGCTGTACAGTCTAATGGGGACTGGACTTACCAGACACGATCTTACTTGGCCCTTACCCCCTCTAGTGGGGATATTTATACTTGCCATGTAGAGCACAGTGGGAGTTCCCAGGCCATCTTACAGACCTGGATGGCCTCTCTCTGAAGCAGACCGTGAAGATCTCTGTATCTGTATTGACTCTGGGACTTGGcctcatcttcttcttccttgGCCTGGTTTTCTGCCAAAAAGCAGGCTCCTCTGACTACACTCCTCTCTCGGGGTCCAATTATCCTGAAGGTAA

>Mafu_DAB_1_partial_exon_2-3

AGGATTTCGTGTATCAGTACAAGGCTGAGTGTTACTTCACCAACGGCACGGAGCGGGTGCGGTTTGTGGTTAGAGACATCTACAATGGCGGGGAGGTTTTGCGCTTCGACAGCGACGTGGGGCACTTCGTGGCCCTGACGGAGCTGGCGCGGCCCGAGGCTGAGTATAGGAACAGTCAGGAGGCGCTCCTGGAGAAAGCACGAGCCGCTGCGGACACGCTGTGCAGACACAACTACGAGGCAACTAAGCCCTACACCACCTGCTGGTCTGCTCTGTCACTGGCTTCTATCCAAGCAAGATCAAGGTCACCTGGTTCAAGAATGGGCAGGAGGAAACAGCTGGGATCGTGTCCACTGGTGTGATACAACATGGAGACTGGACCTACCAGACCCTGGTCATGTTGGAAATGACTCCCCAGAGCAGAGATGTCTACACCTGCAGTGTGGAGCATGCCAGCCTACAGAGCCCCATCAGTGTGGAATGGA

>Mafu_DAB_10_partial_exon_3-4

AGTGACCATCTCCCCCTCCAAGACAGACGCCCTGCAGCACCTGCTGGTCTGCTCTGTCACTGGCTTCTATCCAAGCAAGATCAAGGTCACCTGGTTCAAGAATGGGCAGGAGGAGACAGCTGGGATCGTGTCCACGGGTGTGATACAACATGGAGACTGGACCTACCAGACCCTGGTCATGTTGGAAATGACTCCCCAGAGCAGAGATGTCTACACCTGCAGTGTGGAGCATGCCAGCCTACAGAGCCCCATCGGGCACAGTCTGAATCTGCCCAGAGCAAACTGCTGAGTGGAATTGGAGGCTTTGTCCTGGGGCTGATCTTCCTCAGTGTAGGGCTGATCATCCACCTGAAGAACAAGAAAGGTGA

>Mafu_DAB_11_partial_exon_3-4

CACCTGCTGGTCTGCTCTGTCACTGGCTTCTATCCAAGCAAGATCAAGGTCACCTGGTTCAAGAATGGGCAGGAGGAGACAGCTGGGATTGTGTCCACGGGTGTGATACAACATGGAGACTGGACCTACCAGACCCTGGTCATGTTGGAAATGACTCCCCAGAGAGGAGATGTCTACATCTGCAGTGTGGAGCATGCCAGCCTACAGAGCCCCATCAGTGTGGAATGGAGGGCACAGTCTGAATCTGCCCAGAGCAAACTGCTGAGTGGAATTGGAGGCTTTGTCCTGGGGCTGATATTCCTCAGTGTAGGACTGATCATCTACCTGAAGAACAAGAAAGGTGA

>Mafu_DAB_12_partial_exon_3-4

GGTGATTGTGTATCCATCAAAGCTGGCTCCCCTGGGACACCACAACCTGCTTGTCTGTTCTGTCACTGGTTTCTATCCTGGGGATATTGAGGTCAGGTGGTTCCTGAATGGGCAGGAGGAGACAGCTGGGATTGTGTCCACAGGCCTGGTCAGCAATGGAGACTGGACTTACCAGATCTTGGTGATGCTGGAAATGACCCCCAAGCATGGTGATGTCTACACATGCCAAGTGGAGCACTTCAGCCTTCAGAGACCTGTCATCTTGGACTGGAAAGCACAGTCTGAATCTGCCCAGAGTAAGATGCTAAGTGGAGTTGGGGGGCTCGTGCTGGGGCTGATCTTCTTTGGGGTTGGCCTCATTGTCCACAAGAGGAGTCAGAAAGGTGA

>Mafu_DAB_13_partial_exon_3-4

CTGGACTTTCCAGATCTTGGTGATGCTGGAAATGACCCCCAAGCTTGGTGATGTCTACACCTGCCAAGTGGAGCACTCCAGCCTTCAGAAACCTGTCATCTTGGATTGGAAAGCACAGCCTGAATCTGCCCAGAGTAAGATGCTTAGCGGAGTTGGGGGCCTCGTGCTGGGACTGATCTTCTTTGGGGTTGGCCTCATTGTCCATAAGAGGAGTCAGAAAGGTGA

>Mafu_DAB_14_partial_exon_2-4

ATGCTGTGTGTCTCTCTTTTCAGAGGCATCTGGACAGAGGTTCTGACTGTGACCTTGCTGGTGCTGAATTCCCAGGTGACTGCAGGCAGACATGCTCCAGAGCTCTTCAGGGAATTGGTAAAATCCGAATGTCACTTTGTGAACGGGACGCAGCACGTGCGGTTTGTGCACAGATACATCTACAACCGGGGGGAGTATGCGCGCTTTGACAGTGACGTGGGGTGACGGAGCTGGGGAGGCGGGATGCTGAGAGGTGGAACCTCTGGAAAGAGCTCCTGGAGCGCGAACGCGCCTACGTGAACACTTTCTGCAGGCACAACTACAAGCTGTCTGAACCCTACTTAGACCCCAGGCGCGGTGATTCAGCCTGAGGTGACTGTGTATCCATCAAAGCTGGCTCCCCTGGGACACCACAACCTGCTTGTCTGCTCTGTCAGTGGTTTCTATCCTGGGGACATTGAGGTCAGGTGGTTCCTGAATGGGCAGGAGGAGACAGCTGGGGTTGTGTCCACAGGCCTGATCAGCAATGGATACTGGACTTTCCAGATCTTGGTGAT

>Mafu_DAB_15_partial_exon_1-2

ATGCTGTGTGTCTTCCTTTCCAGAGGCATCTGGACAGAGGTTCTGGCTATGACCCTGCTGGTGCTGTATTCCCAGGAGGGTGCAGGCAGAGATGCACCAGAGCATTTCATGGAGCAGATGAAGTCCGAGTGTTACTTTGCGAACGGGACGCAGCACGTGCGGTTTGTGGAGAGATACATCTACAACCGGGAGGAGTTTGTGCGCTTCGACAGCGCCGTGGGGGAATACGTGGCGCTGACGGAGCTGGGGCGGGGGACTGCTGAGCATTGGAACAGCCAAAAGGATTACATGGAGAGCAGACGGGGCATGGTGGACACTTTGTGCAGACCCAACTACAGGGTGTCTGAGCCCTTCTTACTCCCCAGGCGCGGTAA

>Mafu_DAB_16

ATGCTATGTGTCTCACTCCCCAGGGGCATTTGCAAAGAGATGCTGCTTGTGACTCTACTGGTGCTCAGTTCTCAGGTGGCTGCAGGCAGACATGCTCCAGCACTTCACTGAGCTGAAGAAACCGAGTGTTCCTTTGTGAACAGGACTCAGCTCGTGCGGTATTTGGAGAGACACATCCACGACCGGGAGGAGTTTCTGCGCTTCGACAGCGACGTGGGGGAGCATGGGGCGCTGACGGAGCTAGGGCGGGGATTGCCGAAGATTGGAACAGTCAGAAGGAAACCCTGGATTATAAACTTGGCCAGGTGGTTAACTAAGGCACAACTACGAGGCGACTGAGCCCTACTTAGTGCGCAGGCGCGGTGAGTTTGAGCCAGAGGTGACTGTGTATCCATCAAAGATGGCTCCCTTGGGACACCACAACCTGCTTGTCTGCTCTGTCACTGGTTTCTATCCTGGAGACACTGAGGTCAGGTGGTTCCTGAATGGGCAAAAACGACAGCTGGGGTTGTGTCCACAGGCCTGATCAGCAATGGAGACTGGACTTACCAGATCCTGGTGATGCTGGAAATGACCCCCAAGCATGGAGATGTTTATACCCGCCAAGTGGAGCACTCCAGCCTTCAGAGACCTTTTATTTTTGACTGAACACAGTCTGACTCTGCCCAGAGTAAGATGCTGAGTGGAGTTGGGGGGCTCATGCTGGGGCTGATCTTCTTTGGGGTTGGCCTCATGGTCCACAAGAGGAGTCAGAAAGGTGA

>Mafu_DAB_17_partial_exon_3-4

TTAAGCCTGAGGTGATTGTGTATCCATCAAAGCTGGCTCCCCTGGGACACCACAACCTGCTTGTCTGCTCTGTCACTGGTTTCTATCCTGGGGACATTGAGGTCAGGTGGTTCCTGAATGGGCAGGAGGAGACAGCTGGGGTTGTGTCCACAGGCCTGGTCAGCAATGGAGACTGGACTTACCAGATCTTGGTGATGCTGGAAATGACCCCCAAGCGTGGTGATGTCTACTCCTGCCACGTGGAGCACTCCAGCCTTCAGAAACCTGCCATCTTGAACTGGAAAGCACAGTCTGAATCTGCCCAGAGTAAGATCCTGAGTGGAGTTGGGGGCCTTGTGCTTGGCCTGATTTTCTTTGGGGTTGGCCTCATTGTCCACAAGAGGAGTCAGAAAGGTGA

>Mafu_DAB_18_partial_exon_3-4

GTGTATCCATCAAAGCTGGCTCCCCTGGGGCACCACAACCTGCTTGTCTGCTCTGTCACTGGTTTCTATCCTGGGGACATTGAGGTCAGGTGGTTCCTGAATGGGCAGGAGGAGACAGCTGGGGTTGTGTCCACAGGCCTGGTCAGCAATGGAGACTGGACTTACCAGATCTTGGTGATGCTGGAAATGACCCCCAAGCATGGTGATGTCTACACCTGCCAAGTGGAGCACTCCAGCCTTCAGAAACCTGTCATCTTGGATTGGAAAGCACAGTCTGAATCTGCCCAGAGTAAGATGCTGAGTGGAATTGGGGGGCTCGTGCTGGGGCTGATCTTCTTTGGGGTTGGCCTCATTGTCCACAAGAGGAGTCAGAAAGGTGA

>Mafu_DAB_2_partial_exon_3-4

CAGGAAGAGATAGCTGGGGTTGTGTCCGTAGGTCAGATCAGCAATAGATACTGGACTTACCAGATCCTGGTGATGCTGGAAATGACCCCCAAGCAAGGACATGTCTACAGCTGCCAAGTGCAGCACTCCAGCCTTCTGAGACCTGTCACCTTGGACTAGAAAGCACAGTCTGAATCTGCTCAGAGTAAGATGCTGAGTGGAATTGGGGTCCTTGTGCTGGGGATAATCTTTTTTGGGTTTGGTCTCATTGTCCACGAGAAGAGCCAGAAAGGTGA

>Mafu_DAB_3_partial_exon_2-4

AACACTTCATGGAGCAGAGAAAGGTCGAATGTCACTTTGTGAATGGGACAGAGCACGTGCGGTATGTACTGAGATGCATCCATAATAGGGAGGAGATCCTGCGCTTCGACAGTGATGTGGGGAAGTTTGTGGCACTGACAGAGCTGGGCCAGCCTGAGGTTGAGTATTGGAACAGCCAGAAGAACATCCTGAAGTATAGACAAGATCAAGTGAACACTTACTGCAGGCCCAACTACCAGGCTGTTAAAGTCTTTTCACAGAGGAGGAAAGGTGAGTGTGATTGTGTATCCATCAAAAATGGCTCCCCTGGGACATCACAACCTGCTTGTCTGCTCTGCCACTGGTTTCTATCCTGGAGACATCGAGGTCAAGTGGTTCCTGAATGGGCAGGAGGAGACAACTGGGATTGTGTCCACAGGCCTGATCAGCAATGGAGACTGGACCTACCAGATCTTGGTGATGCTAGAAATGACCCCCAAGCATGGAGATGTCTACACCTGCCAAGTGAAGCACTCTAGCCTTCAGAGACCTGTCACCTTGGATTGGAAAGCACACTCAGAATCTTCCCAGAGTAAGATGCTGAGTGGAGTTGGGAGCCTCTTGCTGGGCCTGATCTTCTTGGGGGTTGGCCTCATTGTCCACAAGAGGAGTCAGAAAGGTGA

>Mafu_DAB_4

ATGGCATGTGTCTTGCTTCGCAGAGGCATCTGGACAGAGGTTCTGACTGTGACTCTGCTGGTGCTGAATTCCCAGGTGACTGCAGACAGACATGCCCCAGCAGCGCACTTTATGGATCAGTTGAAATCTGAGTGTCACTTTGTCAATGGGGCTGAGCACGTGCGGTTTGTGGGGAGACTCATCTACAGCCATCAAGAGAGCCTGTGCTTCGACAGCGACGTGGGGGAGTTCATGGCCTTGACCGAGCTGGGGCGGCCCCTTGCGTTGAGCCTGAAGTGACTGTATATCCATCAAAGATGGCTCCCCTAGGATACCCAAACCAGCTTGTCTGTTTTGTTACTATCCTGGGGACATTGAGGTCAAGTGGCTCCTGAATGGTCAGGAGGAGACAGCTGGGATTGTGTCTACAGGCCTGATTAGCAATGGAGACTGGACCTACCAGATTCTGGTGATGCTGGAAATGATCCCCAAGCATGGACACCTCTACACCTGTCAAGTGGAGCACTCCAGCCTTCTGAAACCTGTCACTAAGCACAGTCCAGATTTGCCCAGGGAAAGATGCTGAGTGGAGTTGGTGGCCTTGTGCTAGGGTTGATCTTCCTTGTGGTTGGCCTCACTGTTCACCTTAGGAGTCAGAAAGGTGA

>Mafu_DAB_5_partial_exon_3-4

ATGGATTGTGTATCCATCAAAGATGGCTCCCCTGGGACACCACAACCTGCTTGTCTGCTCCGTCCCTGGATTCTATCCTGTGAAAAGTGAGGTCAGGTGGTTCCTGAATGGGCAGGAGGAGACAGCTGGGGTCATGTCCACAGCCTTATCAGTAATGGAGACTGGATCTATCAGATCCTAGTGATGCTGGAAATGACCCCCAAGCATGGAGATGTCGATACCTGCCAAGTGGAGCACTCTAGCCTTGAGAAACCTGTCATTGTGGACTGGAAAGTTTGAATCTGCCCAGAGTAAGATGCCCAGTGGAGTCGAGGGCCTCGTGCTGGGGCTGATGTTCTTTGGGGTTGGCCTCATTGTCCACAAGAGGAATCAGAAAGATGA

>Mafu_DAB_6_partial_exon_3-4

CCCTTGCATCATCACAGTCTGCCCGTCTGCTCAGTGACTGGTTTCTATCCCGGCCAGATGAAAGGCAGCTGGTTCCACAATGGGTAAGAGAAACGAGATGGGGTTGTGTCCTCAGACCCCATAAGGAATGACGACTGGACATTGCAGATTCTGGTGATGCTGGAAATAATCCCCCAGCATAGACAAGTCTACATCTGTCAAATGGAACCTACAAGCCTTCTGAGGGTTCAATCTGAATCTACCTAGAATGAGAGGCTTGGTGGATTTGGGGCTTCACCTGGGGCCTGTTCTTCTTTGGGCTGGAGTTCATTGTCCATCTCAGGAGTCAGAAAGGTAA

>Mafu_DAB_7_partial_exon_3-4

GTGTATCCATCAAAGCTGGCTCCCCTGGGGCACCACAACCTGCTTGTCTGCTCTGTCACTGGTTTCTATCCTGGGGACATTGAGGTCAGGTGGTTCCTGAATGGGCAGGAGGAGACAGCTGGGGTTGTGTCCACAGGCCTGGTCAGCAATGGAGACTGGACTTACCAGATCTTGGTGATGCTGGAAATGACCCCCAAGCGTGGTGATGTCTACACCTGCCATGTGGAGCACTCCAGCCTTCAGAAACCTGTCATCTTGGACTGGAAAGCACAGTCTGAATCTGCCCAGAGTAAGATGCTGAGTGGAGTTGGGGGCCTCGTGCTGGGGCTGATCTTCTTTGGGGTTGGCCTCATTGTCCACAAGAGGAGTCAGAAAGGTGA

>Mafu_DAB_8_partial_exon_2-3

AGGATTTCGTGTTTCAGGACAAGGCTGAGTGTTACTTCACCAACGGCACGGAGCGGGTGCGGTATGTGGCTAGAGACTTCTACAATGGCGAGGAGACTGCCCGCTTCGACAGCGACGTGGGAGTGTACGTGGCGGTGACGGAGCTGGGGCGGCGCGATGTTGAGTACTGGAACGGTCAGGAGGAGATCCTGGAGGAAGAACGAGCCGCTGTGGACACGGTGTGCAGACACAACTACGAGGTACACAAGCCCTTCACGTGGGACAGAAGAGGTGACACCTGCTGGTCTGCTCTGTCACTGGCTTCTATCCAAGCAAGATCAAGGTCACCTGGTTCAAGAATGGGCAGGAGGAGACAGCTGGGATCGTGTCCACGGGTGTGATACAACATGGAGACTGGACCTACCAGACCCTGGTCATGTTGGAAATG

>Mafu_DAB_9_partial_exon_3-4

TTGAACCCAAGGTAGTTGTGTATCCATCAAAGAAGGCTCCCCTTGGATACCACAACCTGCTTGCCTGCTCTGCCACTGGTTTCTATCCTAGAGACACTGAGGTCAGGTGGTTCTTGAATGGGCAGGAGGAGACAATTGGATTTGTGTCCACAGGCCTCATCAGTAATGAAGACTGGGCCTACCAGATCCTGGAGATGCTGGAAATGACTCCCAAGCATGGAGATGTCTATACCTGCCAAGTGGAGCACTCCAGCCTTCAGAGACCTACCATCTTGGACTGGAAAGCACAGTCTGAATTTGCTCAGAGTAAGATGCTAAATGGAGTCAGGGGCCTCGTTCTGGGGCTGATCTTCTTTAGGGTTTGTTTCATGGTCCACAAGAGGAGTCAGAAAGGTGA

>Mafu_DNAzoo_DBB_1

ATGGTTGATATTTGGATCTCTGCTGGCTGCTGGAAGATTGGTCTGTTAATGACATCAATGCTGTTGAGTTTATCTGCATCTTGGGCCAGGGACATCCCAGAGGATTTCGTGTATCAGTACAAGTTTGAGTGTTACTTCAGCAACTGCACGGGGCGGGTGCGCCTTGTGGTTAGAGACATCTACAATGGCGAGGAGGATGCCCGCCTCGACAGTGAAGTGGGGCAGTTCGTGGCCCTGAGGGATCCGGGGTGGCCCGATGCTGAGCAATGGAACGGTCAGAAGGAGATCCTGGAGAAATATCGAGCCAACGTGGACACGCTGTGCAGACACAACTACGAGGCTTATAAGCGCTTCACATTGGACAGAAGAGTCCAGCCCAGAGTGACCATCTCCCCTTCCAAGACAGATGCCCTGCAGCACCTGCTGGTCTGCTCTGTCTCTGGCTTCTATCCAAGCAAGATCAAGTTGACCTGGTTCAAGAATGGGCAAGAGAAGACAGCTGGGATTGTGTCCACGGGTGTGATACAACATGGAGACTGGACCTACCAGACCCTGGTCATGTTGGAAATGACTCCCCAGAGCAGAGATGTCTACACCTGCAGTGTGGAGCATGCCAGCCTACAGAGCCCCATCAGTGTGGAATGGAGGGCACAGTCTGAATCTGCCCAGAGCAAACTGCTGAGTGGAATTGGAGGCTTTGTCCTGGGGCTGATCTTCCTCAGTGTAGGGCTGATCATCCACCTGAAGAACAGGAAAGGTGA

>Mafu_DNAzoo_DBB_2

ATGGTTGATGTTTGGATCTCTGCTGGCTGCTGGAAGACACGTCTGTTAATGACATCAATGCTGTTGAGTTTATCTGCATCTTGGGCCAGGGACATCCCAGAGGATTTCGTGTATCAGTACAAGGGCGAGTGTTACTTCACCAACGGCACGGAGCGGGTGCGCCTTGTGTATAGACAGATCTACAATGGCGAGGAATATGTCCGCTTCGACAGCGACGTGGGGCACTTCGTGGCGGTGACGGAGCTGGGGCGGCCAAGTGCTGAGTACTGGAACAGTCAGGAGGAGATCCTGGAGGAAGAACGAGCCTACGTGGACACGGTGTGCAGACACAACTACCAGTTTGACAAGATCTCAGCAGTGGACAGAAGAGTCCAGCCCAGAGTGACCATCTCCCCCTCCAAGACAGACGCCCTGCAGCACCTGCTGGTCTGCTCTGTCACTGGCTTCTATCCAAGCAAGATCAAGGTCACCTGGTTCAAGAATGGGCAGGAGGAGACAGCTGGGATCGTGTCCACGGGGGTGATACAGCATGGAGACTGGACCTACCAGACCCTGGTCATGTTGGAAATGACTCCCCAGAGCAGAGATGTCTACACCTGCAGTGTGGAGCATGCCAGCCTACAGAGCCCCATCAGTGTGGAATGGAGGGCACAGTCTGAATCTGCCCAGAGCAAACTGCTGAGTGGAATTGGAGGCTTTGTCCTGGGGCTGATATTCCTCAGTGTAGGACTGATCATCCACCTGAAGAACAAGAAAGGTGA

>Mafu_DNAzoo_DCB_1

ATGGAACATGTGGAGTTTCTGGGAGGCCCTGTATGACAGTCATACTGATAGTGCTGAGCACACCCACAGACTGAGGTAGGGACATTCCAAAGATTTACCTACATCAGATGGGGTCTGAGGGTCACATGATCAATGGAACCCAACAAGACTCATCTACAACCAGGTGGAGTTTGTGCACTTTGATAGTGATGTGAGGGATTATTTCAGACAAGCTGTGGAAATCCCAAGTCCAGAAATGAAACAGTAAAAAGGAGAAAGTCAAGCATGCAAGGTCCATAATGAATGTGTGCAGAGACAATTTTCTTTTATATGACAAATTCACATCCAGCCTCTAGTGAAAGTTTTACCACCAAAGATACAACCACCTGGGCACCACAACCTGCTCCTCTGTTCTGTGATATTTCTATCCTGGTGAGATCAAGATCAATTGATTCAGAATCGCGAAAGAAGAGAAGACTGGAATCCTGTCCACAGACCGAATTCAGCACAGTGCCTGCACCTTCCAAACCCTTGTGATACTGGAAATGGCCCTCCAAAGAGGAGATGTCTTTACTTGCCATGTGGACCATGCCAGCTTGCAGAATCCTGTCACTGTAAACTGGAGACCACATTCTGAATCTTCCTGGACTAAGAGGCTGACTGGAATTGAGGCCTTGGTATTTGGACTGATCTCACTTGGAGTGAGACTTTACTTGTCATCCACCTCAGAAATTTGAAAGATTCTTATTCTGGTACCAAAGAAGACCCAGATTTTGAAGGGATTGTGAACATTGCCTCATTGCAACAAGATTTTCCCAGAACTGTTGCCCAGTCCTAG

>Mafu_DNAzoo_DMB_1

ATGACATTACTCTACCTGCTACTAGTGGGCTTCAGCCTGGGTTTTTCAGGAACAGGGGGCTTTGTGACTCATGTGGAGAGTGACTGTATACTGGATGAAGAAGGATCCGTAAAGGACTTCGAATATTGCATCTCCTTCAACAAGGATGTGTTGACCTGCTGGAACTCAGATACTAGCAAGATGGTCACTGTTGAATTTGGAATACTGCGTCCATTAGCTGACTGGCTTTCTGACACCCTCAATAATGATACTGCTTTGATAAACCGCCTGAGTGAAGGATTCCAAAACTGTGCCAGTCACACAAAGCCCTTCTGGGGATCACTGACCCACAGGACACGGCCACCATCAGTGCAAGTAGCTCAGGTCACACCATTCAACACAAGGGAGCCAGTGATGCTGGCCTGTTATGTATGGGGCTTCTATCCTGCTGATGTGGCCATTTCGTGGTTGAAGAATGGGCAGCCTGTCCCACACAGTGGCATCCAGAAGGCTGTACAATCCAATGGGGACTGGACTTATCAGACACGATCCTACTTGGCCCTTACCCCCTCTAGTGGGGATATTTACACTTGCTTTGTAGAGCACGGTGGGACTTCCCAAACCATCTTACAGACCTGGACACCTGGCCTCTCCCTGAAGCAAATAGTGAAGATCTCTGTATCTGTACTGACTCTGAGCTTTGGTCTTATCTTCTTCCTCCTTGGCCTGGTTGCACGTAGAAAAGCTGGCTCCTCTGACTACTCTCCTCTCTCGGGGTCCAATTATCCTGAAGGTAA

>Psoc_DNAzoo_DAB_1_partial_exon_2-4

AGCACTTCACAGAGCAGGTGAAGGGTGAATGCTACTTTGAGAACGGGACGGAGCACGTGCGGTTTGTGCTGAGGGCCATCTACAACCGGGAGGAGTATGTGCGCTTCGACAGCCACGTGGGGGAGTTTGTGGCGGTGACGGAGCTGGGGCGGCGCACTGCCGAGTATGGGAACGCCCAGAAGGAGTTTATGGACCACTTACGGACCGCGGTGGACAGCTATTGCATGCATAACTACGAGGGAATCGAGCCCTTCTCAGTACCCAGGAGGATTAAGCCCGAGGTGATTGTGTATCCATCAAAGATGGTGCCCCTGGGACACCACAACCTGCTTGTCTGCTCTGTCACTGGTTTCTATCCTGGGGACATTGAGGTCAGGTGGTTCCTGAATGGGCAGGAGGAGACGGCTGGCGTTGTGTCCACAGGCCTGATCAGCAATGGAGACTGGACCTACCAGATCCTGGTGATGCTGGAAATGACCCCCAGCCGTGGAGATGTCTACACCTGCCAAGTGGAGCACTCCAGTCTTCAGAGACCTGTCCTCTTTGACTGGAAAGCACAGTCTGGATCTGCCCACAGTAAGATGCTGAGTGGAGTCGGGATCCTTGTGCTGGGGCTGATCTTCTTTGGGGTTGGCCTCATTGTCCACAAGAGGAGTCAGAAAGGTGA

>Psoc_DNAzoo_DAB_2

ATGGTGTGTGTTTTGCTCCCCAAGTATGTCTGGATAGAAGTTCTGGTTATGACTCTGCTGGTGCTGAATTCCCAGGTGGCTGAAGGGAGACATGCCCCTGAGCACTTCACAGAGCAGAGAAAGGCCGAGTGTCACTTTGTGAATGGGACAGAGTACGTACGATATGTGCTGAGATGCATCCACAACCGGGAGGAGATTGTGCGCTTCGACAGCGACGTGGGGGAGTTTGTGGCGCTGACTGAGCTGGGCCGGCCGGAGGCTGAGTATTGGAACAGCCAGAAGGAGATCCTGGAATACAGACGCGACATGGTGAACACTTACTGCAGGCACAACTACCAGGAGATTGAGTACTTTTCAAAGAGCAGGACAGGTGATTGAGCCTGAGGTGATTGTGTATCCATCAAAGACGGCGCCCCTGGGACATCACAACCTGCTTGTCTGCTCTGTCACTGGTTTCTATCCTGGGGACATTAAGGTCAGGTGGTTCCTGAATGGGCAGGAAGAGATAGCTGGGGTTGTGTCTACAGGCCTGATCAGCAATGGAGACTGGACCTACCAGATCCTGGTGATGCTGGAAATGACCCCCAGGCGTGGAGATGTCTACACCTGCCAAGTGGAGCACTCCAGCCTTCAGAAACCTGTTGTCTTGGACTGGAAAGCACAGTGTGAATCTGCCCAGAGTAAGATGCTGAGTGGAGTTGCTGTCCTCACATTGGGCCTGATCTTCTTTGGGGTTGGCCTTATTGTCCACAAGAGGAGTCAGAAAGGTGA

>Psoc_DNAzoo_DAB_3

ATGCTGTGTGCGCGCTCAAAAGTTCTGATGATGACCCTGCTGGTGCTGAATTCCGGGGTGGTTGCAGGCAGAGACAGCCCAAAGCACTTTATGGAGCAGATAAAGGCCGAGTGTCACTTTGTCAATGGGACTGAGCATGTGCGATTTGTGGGGAGACTTATCTACAACAGTCAGGAAATTATGCGCTTCGACAGCGACTTGGGAGAATTCATGGCCTTGACCGAGCTGGGACGGCCCATTGCGGAGCTAATGAACAGCCTGCTGGAGGTTCTGGAGCAAGCGCGGGCCCAGGTGGCCTGGTGCAGAGACAACTATGGGTTGTTGGAGTCCTGGATGCAGAGGAGGGGTGATTGAGCCTGAAGTAACTGTGTATCCATCAAAGATGGCTCCCCTAGGATATCCAAACCAGCTTGTCTGTTTTGTTACTGGTTTCTATCCTGGGGACATTGAGGTCAAGTGGTTCCTGAATGAGCAGGAGGAGACAGCTGGCATTGTGTCCACAGGCCTGATCAGCAATGGAGACTGGACCTATCAGATCCTGGTGATGCTAGAAATGACCCCTAAGCGTGGAGATGTCTATACCTGCCAAGTGGAGCATTCCAGCCTTCAGAATCCTGTCATTGTGGTCTGGGAAGCACAGTCCACGTCTGCCCAAGGAAAGATGCTGAGTGGAATTGGAAGCCTCGTGCTAGGGCTGATCTTCCTGGGGATTGGCCTCGCTGTTCACCTTAGGCGTCAGAGAGGTGA

>Psoc_DNAzoo_DAB_4_partial_exon_2-4

AGTACTTTATGGAGCAGATGAAGGCCGAGTGTCACTTTGTCAATGGGACTGAGCATGTGCGATTTGTGGGGAGACTTATCTACAACAGCCAGGAAATTTTGTGCTTCGACAGCGACTTGAGGGAATTCACGGCTTTGAGCCAGCTGGGGCAGCCCATTGTGGAGCTAATGAACAGCCTGCTGGAGGTCCTGGAGCAAGCGTGGGCCCAGGTGGCCTGGTGCAGAGACAACCATAGGTTGTTGGAGTCCTGGATGCAGAGGATTGAGCCTGAAGTAACTGTGTATCCATCAAAGATGGCTCCCCTAGCATACCCAAACTAGCTTGTCTGTTTTGTTACTGATTTCTATCCTGGGGACATTGAGGTCAAGTGGTTCATGAATGATCAGAAGGAGACAGCTAGGGTTGTGTCCACAGGCCTAATTAGCAATGGAGACTGGACCTGCCAGATCCTGGTGATGCTGGAAATGACCCCTAAGCATGGAGATGTCTACACCTGCCAAGTGGAGCATTCCAGCCTTCAGAATCCTGTCATTGTAGTCTAGGAAGCACAGTCTAGGTCTTCCCAAGGACAGATGCTGAGTGCAATTGGCAGCCTCATGCTAGGGCTGATCTTCCTGGGGATTGGCCTCACTGTTCACCTTAGGAGTCAGAGAGATGA

>Psoc_DNAzoo_DMB_1

ATGAGGTTACTCTACCTGTTACTAGTGGGCTTCAGTCTGGGTTTTTCAGGAGCAGGGGGTTTTGTGACCCATGTGGAGAGTGGCTGTATGCTGGATGAAGAAGGATCAGTAAAGGACTTTACATATTGTATCTCCTTCAACAAGGATGTGTTGACCTGCTGGAACTCAGAGACTAACAAGATGACCACTGTTGATTTTGGAGTGCTATACCCATTAGCTGAGCAACTTTCTAGAATCCTCAATAATGATAGTGCTTTTATAGACCACCTGAGCAAAGGACTCCAGGACTGTGCTAGTCACACAAAGCCCTTCTGGGGATCACTGACCCAAAGGACATGGCCACCATCAGTGCAAATAGCCCAGACTACACCATTTAACACAAGAGAGCCAGTAATGCTGGCCTGTTATGTATGGGGCTTCTATCCTGCTGATGTGGCCATTTCATGGTTGAAGAATGGGCAGCCTATCCCACACAGTGGCATCCAGAGGGCTGTACAATCCAATGGAGACTGGACTTATCAGACACGATCCTACTTGGCCCTTACCCCCTCTAGTGGGGATATTTACTCTTGTCATGTAGAGCATAGTGGGACTTCCCAGATCATCTTACAGACTTGGACACCTGGCCTCTCTCTGAAGCAGACAATAAAGATCTCTGTATCTGTGTTGACTCTGAGCCTTGGCCTCATCTTCTTCCTCCTTGGCCTGGTTGCCTGCCAAAAAGCTGGCTCCTCTGATTACACTCCTCTCTTGGGGTCCAGTTATCCTGAAGGTAA

>vour_DAB_1

ATGGTGTGTGTCTTACTCCCCACAGGCGTCTGTATACAAGTTCTGGCTATGACCCTGTTGGTTGTAAATTCCCACATGGTTGCAGGCAGACACACCCCAGAGCACTTCACCGAGCATGCTAAGTCCGAGTGTCACTTTGAGAACGGGACGGTGCACGTGTGGTTTGTGGATAGATACATCCACAACCGCGAGGAGTTTATGCGGTTTGACAGCGACGTGGGGGAGTATGTGGCGCTGACGGAGCTGAGGCGTCCCAGTGCTGAGTATTGTAACAGCCGCAAGGAGATTCTGGCAGAGACTGGCGGCGGTGGACTGGTTCTGCAGGGTCTACTGCAAGGTGTCTGAGCTCTTCTCAGTGCACAAGAGCGGTGAGTTTATGCCTGAGGTGATTGCGTATCCATCAAAGATGGCCCCCCTGGGACTCCACAACCTGCTTGTCTGCTCTGTCAGTGGTTTCTATCCTGGAAACATTAAGGTCAGGTGGTTCCTGAATGGGCAGGAGGAGACAGCAGGGATTGTGTCCACAGGCCTCATCAGCAATGGAGACTGGACCTACCAGATCCTTGTGATGCTGGAAATGACCCCCAAGCATGGAGACATGTACACCTGCCAAGTGGAGCACTCCAGCCTTCAGAGACCTGCCATGTTGGGTTGGAAAGCACAGTCTGAATCTGCCCAGAGTAAGATGCTGAGTGGAGTTGGGGGCCTCGTGCTGGGGCTGATCTTCTTTGGGGTTGGCCTCATTGTCCACAAGAGGAGTCAGAAAGTCTCCCTCCCATGTTTGGAGGCCCCTGCTTGTGCCCAGAGCTCCAAGAGATTAGTTCTACTGACTCCTTCCCTGGAATAG

>vour_DCB_1

TGTGTGTGGAGCTTCTGGGAGGCCCCTGTATGACAGTCCTATTGATGGTACTGAGCACACCCACAGCCTGGGGCAGGGACATTCCAGGTAAAATTACCTACGTCAGGTGAGGTCTGAGTGTCACATGACCAATGGAATCCAACAGGTCCACTTTTGAAGACTCATCTATGACCGGGAGGAGTTTGTGCACTTTGACAGTGACGTGGGACTATTTGAGGCAAAAATGGAGTTGTGGAGATCCCAAGTCCAGAAATGGAACAGGCAGAAGGAAATAGTCAAGCGTGCAAGGTCCATAGTGAATGTGTGCAGGCACAATTACCTTTTATATGATAAACTCATATTGCAAAGGAAAGGCCAGGGTTAAGGTTTTCCCCTCAAAGATACGACCTCTTGGGCACCACAACCTGCTCCTCTGTTCTGTGACCAGTTTCTATCCTGGTGAGATCAAGGTCAGCTGGTTCAGGAATGCAAAAGAAGAAAAGGCTGGAGTCTGGTCCACAGGCCAAATCCAGAATGGTGACTGGACCTTCCAGACCCTTGTGATGCTGGAAATGACCCCCCAAAGAGGAGATGTCTTTATTTGCCATGTGGACCATGTCAGCTTGCAGAGCCCTATCACTGTAGACTGGAGGGCACCGACTGAATCTGCCCGGACTAAATGCTTACTGGAATTGGGACTTGGTGCTTGGACCGATTTTACTTGGAGTGGGACTTGTCATCCACCTCAGAAGTTTGACAGATTCCTATTCTGGGACCAAAAAAGACTCAATTTTGGAAGGGATTGTGAACATTGCCCCATTGCAGCAAGATTTTCCCAGAGCTGTTGCCCAGTCCTAG

>vour_DMB_1

ATGCTCAAGCAAAGAATGAGGTTACTCCACCTACTACTACTGGGCTTCAGCCTGGGTTTTTCAGGAGCAGCAGGGGGCTTTGTGACCCATGTGGAGAGTGGCTGTATACTGGATGAAGAAGGATCAGTAAAGGACTTCACATATTGTATCTCCTTCAACAAGGATGTGTTGACCTGCTGGGACTCAGAGGCTAACAAGATGGCCGCCGTTGATTTTGGGGTGCTGCATCCATTAGCCGAAGAGCTTTCTGAAATCCTCAGTAACAGAAGTGATTTTATGGACCATATGAACAAAGGACTCCAGGACTGTGCCAGTCACACAAAGCCCTTCTGGGGATCATTGACCCAAAGGACACGGCCACCATCAGTGCAAGTAGCCCAGACCACACCATTCAACACAAGGGAGTCAGTGATGCTGGCTTGTTATGTATGGGGCTTCTATCCTGCTGATGTGGCCATTTTGTGGTTGAAGAATGGGCAGCCTATCCCAAACAGTGGCATCCAGAGGGCTGTACAATCCAATGGGGACTGGACTTATCAGACACGATCCTACTTACCCCTTACCCCCTCTAGTGGGGATATTTACACTTGCCATGTAGAGCACAGTGGAACTTCCCAGCCTATCTTGCAGAACTGGACACCTGGCCTCTCTCTGAAGCAGATAATGAAGATCTCTGTATCTGTATTGACTCTGAGCCTCGGCCTCACCCTCTTCGTCCTTGGCCTGGTTGCCTGCCGAAAAGCTGGCTTCTCTGACTACACTCCTCTCTTGGGGTCCAATTATCCTGAAGGTAA

>Bepe_DAB_1

ATGCCATGTGTCTCCTTCTCCAGAGTCATCTGGACAGAGGTTCTGACTGTGTCCCTGATAGTGCTGGCTTCCCAGGTAGCTGCAGGCAGACATGCCCCAGAGCACTTCACGGAGCAGCTGAAGTTCGAATGTTACTTTGTGAACTGGACAGAGCACGTACGATTTGTGCTCAGATACATCTACAACCGGGAGGAGTACGTGCGCTTTGATAGCGACGTGGGGGAGTTCTTGGCGGTGACGGAGCTGGGGCGGTGCAGTGCTAAGTATTACAATGGACTGGAGGACGAGCTGGAGGAGAAACGAGCCTACGTGGACACTTTGTGCAGGCACAACTACGGGGTGTTTCACAGATTCTTGGTCCCCAGGCGTGGTGACTGAGCCCGAGGTGACTGTGTATCCATCAAAGCTGGCTCCCCTGGGACACCACAACCTGCTTGTCTGCTCTGTCACTGGTTTCTATCCTGGGGACATTGAGGTCAGGTGGTTCCTGAATGGGCAAGAGGAGACAGCTGGGGTTGTGTCCACAGGCCTGATCAGCAATGGAGACTGGACTTACCAGATCTTGGTGATGCTGGAAATGACCCCCAGGCATGGTGATGTCTACACCTGCCAAGTGGAGCACTCCAGCCTTCTGAGACCTGTCGTCTTGGACTGGAAAGCACAGTCTGAATCTTCCCAGAATAAGATGCTGAGTGGAGTCGGGGGCCTTGTGCTGGGGCTGATCTTCTTTGGGGTTGGCCTCATGGTCCTCAAGAGGAGTCAGAAAGGTGA

>Bepe_DAB_10

ATGTTGTGTGTTTCCCTCTCTAGAGGCATCTGGACCAACGTTCTGGCTGTGACCCTGATGGTGCTGACTTCCCAGGTGGCTGCAGGCAGACATGCCCCAGAGCACTTCATGGAGCAGCTGAAGGCCGAGTGTCACTTCGAGAACGGGACGCAGCGCGTGCGGTATGTGCAGAGATACATCTACAACCGGCAGGAGTTAGTGCGCTTCGACAGCGACGTGGGCGAGTTCGTGGAGGTGTCGGAGCTGGGGCGGGGGATCGCTGAGTATTTCAACAGCCAGAAGGAGTTCCTGGAGCAGAGACGGGCCCAGGTGGACAACTACTGCCGGCACAACTACGGGGTGATTGAGCGCTTCTCAGTGGCCAGGCGCGGTGATTGAGCCCGAGGTGATTGTGTATCCATCAAAGTTGACTCCCCTGGGACACCACAACCTGCTTGTCTGCTCTGCCACTGGTTTCTATCCTGGGGACATTGAGGTCAGGTGGTTCCTGAATGGGCAGGAAGAGACAGCTGGGGTTGTGTCCACAGGCCTGGTTGGTAATGGAGACTGGACTTACCAGATCTTGGTGATGCTGGAAATGACCCCCAAGCGTGGTGATGTCTACACATGCCAAGTGGAGCACTCCAGCCTTCAGAAACCTGTCATCTTGGACTGGAAAGCACAGTCTGTATCTGCCCCGAGTAAGATGCTGAGTGGAGCTGGGGGCCTCGTGCTGGGGCTGATCTTCTTTGGAGTTGGTGTCATTGTCTACAAGAGAAGTCAGAAAGGTGA

>Bepe_DAB_11

ATGCTGTGTGCCTCCCTCTCCAGAGGCATCTGGAGAGAGGTTCTGGCTGTGACCCTGCTGGTGCTGAATTCCCAGGTGACTGCAGGCAGACATGCCCCAAAGCTCTTCAGGGAACTGGTAAAATCCGAATGTCATTTTGTGAACGGGACGCAGCACGTGCGGTTTGTGCACAGATACATCTACAACCGGGAGGAGTACGCGCGCTTCGACAGCGACGTGGGGGAGCATGTGGCGGTGACAGAGGTGGGGAGGCCCGATGTGGAGCGTTGGAACCGCTGGAAAGAGCTCCTGGAGCGCGAACGGGCCTACGTGAACACTTTCTGCAGGCACAACTACGAATTGTCTGAACCCTACTTAGACCCCAAGCGCGGTGATTCAGCCTGAGGTGACTGTGTATCCATCAAAGATGGCTCCCCTGGGACACCACAACCTGCTTGTCTGCTCTGTCTCTGGTTTCTATCCTGGGGACATTGAGGTCAGGTGGTTCCTGAATGGGCAGGAGGAGACAGCTGGGGTTGTGTCCACAGGCCTTATCAGCAATGGAGACTGGACTTACCAGATCTTGGTGATGCTGGAAATGACCCCCAAGCGTGATGATGTCTACACCTGCCAAGTGGAGCACTCCAGCCTTCAGAAACCTGTCATCTTGGATTGGAAACCACAGTCTGAATCTGCCCAGAGCAAGATGTTTAGCGGAGTTGGGGGCCTCCTGCTGGGACTGATCTTCTTTGGGGTTGGCCTCATTGTCCACAAGAGGAGTCAGAAAGGTGA

>Bepe_DAB_12

ATGCTGTGTGTCTTACTCCCCCAGGGTATCTGGACAGAGGTTCTGGCTGGGATCCTGCTCATGCTGAATTCCTGGATGGCTGCAGGCAGACATGCCTCAGACCACTTCATGGAGCAGAGAAAGGCCGAGTGTTACTCTGAGAACGGACGCAGCACGTGCGTTTTGTGGAGAGACACATCTACAACCAGAAGGAGGCTGTGCGCTTCGACAGCGACGTGGGGGAGTTTGTGGCGCTGACGAGCGAGGGTGGCCCAGTGCGGAGAGTTGGAACGGCAGGAAGGAGCTCCAGGAGCAGAGGACACCGCCCAAGAAGACACCTTCTGCAGGCACACCTCCAAGGTGATGGAGCCCTTCTCAGTGCGCAGGTGTGGTGGCCCCAGGTGACTGTGTATCCATCAAAAACAGCTCCCCTGGGACACCACAACCTGCTTATCTGCTCTGTCACTGGTTTCTATCCTGGAGACATTGAGGTCAGGTGGTTCCTGAATGGGCAGGAAGAGACAGCTGGGGTTGTGTCCACAAGCCTGATCAGCAATGGAGACTGGACTTACCAGATCTTGGTGATGCTGGAAATGACCCCCAAGCATGGAGACATCTACACTTGCCAAGTGGAGCTCTCCAGCCTTCAGAGACCTATCATCTTGGACAGGAAAGCACAGTCTGAATCTGGTCAAAGTCATATGCTGCGTGAATTCAGGAGCCTTGTATTGGGACTGATCTTCTTTGGGGTTGGCCTCATGGTCTACATGAGGAGTAAGAAAGGTGA

>Bepe_DAB_2

ATGGTATGTGTCTTGCTCTGCAGAGGCATCTGGACAGAGATTCTGACTGTGGCTCTGCTGGTGCTGAATTCCCAGGTGACTGCAGGCAGATATGCCCCAGAACACTTCATGGAGCAAAGAAAGGTCGAGTGTCACTTTGTGAATGGGACAGAACACGTGCGGTATGTACTGAGATGCATCCATAATAGGGAGGAGATCCTGCGCTTTGACAGTGACGTGGGGAAGTTTGTGGCACTGACAGAGCTGGGGCGGCCTATGGTTGAGTATTGGAACAGCCAGAAGGACATCCTGAAGTACAGACAGGACCAAGTGAACACTTACTGCAAGCCCACCTACCAGGCTATTAAGGTCTTTTCACAGAGCAGGAATGGTGACTGAACCTGAGGTGATTGTGTATCCATCAAAAATGGCTCCCCTGGGACATCACAACCTGCTTGTCTGCTCTGCCACTGGTTTCTATCCTGGAGACATTAAGGTCAGGTGGTTCCTGAATGGGCAGGAGGAGACAACTGGGATTGTGTCCACAGGCCTGATCAGCAATGGAGACTGGACCTACCAGATCTTGGTGATGTTAGAAATGACCCCCAAGCATGGAGATGTCTACACCTGCCAAGTGAAGCACTCCAGCCTTCAGAGACCTGTCACCTTGGACTGGAAAGCTCACTCAGAATCTGCCCAGAGTAAGATGCTGAGTGGAGTTGGGAGCCTCTTTCTGGGCCTGATCTTCTTGGGGGTTGGTCTCATTATCCACAAGAGGAGTCAGAAAGGTGA

>Bepe_DAB_3

ATGGTGTGTGTCTTGTTCCCCCAGGGTATCTGTATAGAGGTTCTAGTTGTGACACTGCTGGTGCTGAATGCTCAGGTGACTGTAAGCAGCCATGCCCCAGAGCACTTCACGGAGCAGGTGAAGGGCGAATGTTACTTTGTGGACGGGATGGAGCACGTGCGGTTTGTGCTGAGGGCCATCTACAACCGCGAGGAGTACGTGCGCTTCGACAGCGACGTGGGGGAGTTTGTGGCTGTGACGGAGCTGGGGCGCCGCACTGCGGAGTTTGGCAACAGCCAGAAGGAGTTCATGGACCACTTACGGGCGTCGGTGGACACTTACTGCAGGCACAACTACGAGGGGATCGAGCCCTTTATAGCGCCCAGAAGCGGTGATTCAGCCTGAGGTGACTGTGTATCCATCAAAGCTGGCTCCCCTGGGACACCACAACTTGCTTGTCTGATCTGTCACTGGTTTCTATCCTGGGGACATTGAGGTCAGGTGGTTCCTGAATGGGCAGGAGGAGACAGCTGGGGTTGTGTCCACAGGCCTGATCAGCAATGGAGACTGGACTTACCAGATCCTGGTGATGCTGGAAATGATCCCCAAGCATGGACATGTCTACACCTGCCAAGTGGAGCACTCCAGTCTTCGGAGACCTGTTATTTTTGATTGGAAAGCACAGTCTGAATCTGCCCAGAGTAAGATGCTGAGTGGAGTCGGGGTTCTTGTGCTGGGGTTGATCTTCTTTGGGGTTGGCCTCATCGTTCACAAGAGGAGTCAGAAAGGTGA

>Bepe_DAB_4

ATGCTGTGTGTCTTGCTCCCCAGGGGCATCTGGACAGAGGTTCTGGCTGTGACCCTGCTGGTGCTGACTCTCCAGGTGGCTGCAGGCAGACATGCCCCAAAGCACTTCACGGAGCAGCTGAAGGCCGAGTGTTACTTCGAGAACGGGACGCAGCGGGTGCGGTTTGTGCTGAGAGATATCACGAACCGGGAGGAGAATGTGCGCTTCGACAGCGACGTGGGCGAGTTCGTGGCGCTGACGGAGCTGGGCCGTCCTGATGCTGAGCTGTGGAACAGCCAGGAGGACTACCTGGAGCGCGCACGGGCCGCGGTGGACACTTTGTGCAGGCACAACTACGGGGTGATTGAGCACTTCTTACTCCCCAGGCGCGGTGATTGAGCCCGAGGTGATTGTGTATCCATCAAAGCTGGCTCCCCTGGGACACCACAACCTGCTTGTCTGCTCTGTCACTGGTTTCTATCCTGGGGACATTGAGGTCAGGTGGTTCCTGAATGGGCAGGAAGAGACAGCTGGGGTTGTGTCCACAGGCCTGGTCAGTAATGGAGACTGGACTTACCAGATCTTGGTGATGCTGGAAATGACCCCCAAGCGTGGTGATATCTACACATGCCAAGTGGAGCACTCCAGCCTTCAGAAACCTGTCATCTTGGATTGGAAAGCACAGTCTGAATCTGCCCAGAGTAAGATGCTGAGTGGAGTTGGGGGGCTCGTGCTGGGGCTGATCTTCTTTGGGGTTGGCCTCATTGTCTACAAGAGGAGTCAGAAAGGTGA

>Bepe_DAB_5

ATGCTGTGTGTCTTGCTCCCCAGGGGCATCTGGATAGATGTTCTTGCTGTGACCCTGCTGGTGCTGACTTCCCAGGTGGCTGCAGGCAGACATGCCCCAAAGCACTTCACGGAGCAGTTGAAGGGCGAATGTTACTTTGAGAACGGGACGGAGCACGTGCACTTTGTGCTCAGATACATCTACAACCGGCAGGAATACGCGCGCTTCGACAGCGACGTGGGGGAGTATGTGGCGGTGTCGGAGCTGGGGCGGCGCAGTGCTGAGTATTGGAACAGCCAGGAGGAGCTCCTGGAGCAGAGACGGGCCCAGGTGGACACTTACTGCCGGCACAACTACGGGGTGATTGAGCGCTTCTTAGTCCCCAGGCGCGGTGATTGAGCCCGAGGTGATTGTGTATCCATCAAAGCTGGCTCCCCTGGGACACCACAACCTGCTTGTCTGCTCTGTCACTGGTTTCTATCCTGGGGACATTGAGGTCAGGTGGTTCCTGAATGGGCAGGAAGAGACAGCTGGGGTTGTGTCCACAGGCCTGGTCAGTAATGGAGACTGGACTTACCAGATCTTGGTGATGCTGGAAATGACCCCCAAGCGTGGTGATATCTACACATGCCAAGTGGAGCACTCCAGCCTTCAGAAACCTGTCATCTTGGATTGGAAAGCACAGTCTGAATCTGCCCAGAGTAAGATGCTGAGTGGAGTTGGGGGGGCTCGTGCTGGGGCTGATCTTCTTTGGGGTTGGCCTCATTGTCTACAAGAGGAGTCAGAAAGGTGA

>Bepe_DAB_6

ATGTTGTGTGTCTCCCTCTCCAGAGGCATGTGGATAGAGGTTCTGGCTGTGACCCTGCTGGCATTGAATTCCCAGGTGGCTGCAGGCAGACATGCTCCAGAGCACTTCACGGAGCAGGTGAAGGCTGAGTGTCACTTTGTGAACCGGACGCAGCACGTGCGGTTTGTGGTGAGAGATATCACGAACCGGGAGGAGAATGTGCGCTTCGACAGCGACGTGGGCGAGTTTGTGGCGCTGACGGAGCTGGGCCGTCCTGATGCTAAGCTTTGGAACAGCCAGAAGGACTACCTGGAGCGCGCACGGGCCGCGGTGGACACTTTGTGCAGGCACAACTACGGGGTGATTGAGCCCTTCTCAGTGGCCAGGCGCGGTGACTGAGCCGGAGGTGAATGTGTATCCATCAAAGTTGACTCCCCTGGGACACCACAACCTGCTTGTCTGCTCTGTCACTGGTTTCTATCCTGGGGACATTGAGGTCAAGTGGTTCCTGAATGGGCAGGAGGAGACAGCTGGGGTTGTGTCCACAGGCCTGATCCGCAATGGAGACTGGACTTACCAGATCTTGGTGATGCTGGAAATGACCCCCAAGCGTGGTGATGTCTACACCTGCCAAGTGGAGCACTCTAGCCTTCAGAAACCTGTCATCTTGGATTGGAAAGCACAGTCTGAATCTGCCCAGAGTAAGATGCTGAGTGGAGTTGTGGGGCTCGTACTGGGGCTGATATTCTTTGGGGTTGGCCTCATTGTCCACAAGAGGAGTCAGAAAGGTGA

>Bepe_DAB_7

ATGCTGTGTGTCTTGCTCCCCAGGGGCATCTGGACAGAGGTTCTGGCTGTGACCCTGCTGGTGCTGACTTTCCAGGTGGCTGCAGGCAGACATGCCCCAAAGCACTTCGCGGTGCAGGCGAAGTCCGAGTGTTACTTTGTGAACGGGACGGAGCACGTGCGGTTCATGGACAGATACTTCTACAACCGCGAGGAGACTGTGCGCTTCGACAGCGACGTGGGGGAGTATGTGGCGGTGTCGGAGCTGGGCCGTCCTGATGCTGAGTACTGGAACAGCCAGGAGGAGCTCCTGGAGCGCAAACGGACCGCGGTGGACTGGTTCTGCAGGGTCTGCTACGAGATTTCTGAGCGCTTCTTAGTCCCCAGGCGCGGTAATTGAGCCTGAGGTGATTGTGTATCCATCAAAGCTGGCTCCCCTGGGACACCACAACCTACTTGTCTGCTCTGTCACTGGTTTCTATCCTGGGGACATTGAGGTCAGGTGGTTCCTGAATGGGCAGGAGGAGACAACTGGGGTTGTGTCCACAGGCCTGGTCGGTAATGGAGACTGGACTTACCAGATCTTGGTGATGCTGGAAATAACTCCCAAGCGTGGTGATGTCTACACCTGCCAAGTGGAGCACTCCAGCCTTCAGAAACCTGTCATCTTGGATTGGAAAGCACAGTCTGAATCCGCCCAGAGTAAGATGCTGAGTGGAGTCGGGGGCCTCGTGCTGGGGCTGATATTCTTTGGGGTTGGCCTCATTGTCCACAAGAGGAGTCAGAAAGGTAA

>Bepe_DAB_8

ATGCTGTGTGTCTTGCTCCCCAGGGGCATCTGGACAGAGGTTCTGGCTGTGACCCTGCTGGTGCTGACTTCCCAGGTGGCTGCAGGCAGACATGCCCCAAAGCACTTCACGGAGCAAGGAAAGTGCGAGTGTCACTTCGAGAACGGGACGCAGCACGTGCGCTTTGTGCAGAGACACATCACCAACCGGCAGGAGAACGTGCGCTTCGACAGCGACGTGGGCGAGTTCGTGGCGCTGACGGAGCGGGGGCGGCGGGATGCAGAGCTGTGGAACAGCCAGGAGGACTACCTGGAGCAAACCCGGGCCTCGGTGGACACTTTGTGCAGGCACAACTACCAGTCGTCTGAGCCCTTCTTAGTGCGCAGGCGCGGTGACTGGGCCTGAGGTGATTGTGTATCCATCAAAGCTGGCTCCCCTGGGACACCACAACCTGCTTGTCTGCTCTGTCACTGGTTTCTATCCTGGGGACATTGAGGTCAGGTGGTTCCTGAATGGGCAGGAGGAGACAGCTGGGGTTGTGTCCACAGGCCTGGTCAGCAATGGAGACTGGACTTACCAGATCTTGGTGATGCTGGAAATGACCCCCAAGCATGGTGATGTCTACACATGCCAAGTGGAGCACTCCAGCCTTCAGAAACCTGTCATCTTGGACTGGAAAGCACAGTCTGAATCTGCCCAGAGTAAGATGCTGAGTGGAGTCGGGGGGCTCGTGCTAGGGCTGATCTTCTTTGGGGTTGGCCTTATTGTCCACAAGAGGAGTCAGAAAGGTGA

>Bepe_DAB_9

ATGCTGTGTGTCTCCCTCTCCACAGGCATCTGGACAGAGGTTCTGGCTGTGACCCTATTGGCACTAAATTCCAAGGTGGCTACAGGCAGACATGCTTCAGAGCACTTCGCGGTGCAGGCGAAGTCCGAGTGTTACTTTGTGAACGGGACGGAGCACGTGCGGTTCATGGACAGATACTTCTACAACCGCGAGGAGACTGTGCGCTTCGACAGCGACGTGGGGGAGTATGTGGCGGTGTCGGAGCTGGGGCGGCGGGATGCTGAATACTGGAACAGCCAGGAGGAGCTCCTGGAGCAGAAACGGGCCTACGTGGACACTTTGTGCAGGAACAACTACGAGTTGTCTGAGCGCTTCTTAGTCCCCAGGCGCGGTGACCAGAGGTGATTGTGTATCCATCAAAGTTGACTCCCCTGGGACACCACAACCTGCTTGTCTGCTCTGTCACTGGTTTCTATCCTGGGGACATTGAGGTCAGGTGGTTCCTGAATGGGCAGGAGGAGACAGCTGGGATTGTGTCCACAGGCCTGATCCGCAATGGAGACTGGACTTACCAGATCTTGGTGATGCTGGAAATGACCCCCAAGCGTGGTGATGTCTACACCTGCCAAGTGGACCACTCCAGCCTTCAGAAACCTGTCATCTTGGATTGGAGTGAGATAAAGCACAGTCTGAATCTGCCCAGAGTAAGATGCTGAGTGGAGTTGGGGGCCTCGTGCTGGGGCTTATATTCTTTGGAGTTGGCCTCATTGTCCTCAAGAGGAGTCAGAAAGGTGA

>Bepe_DBB_1

ATGCTTGATGTTTGGATCTCTGCTGGTTACTGGAAGATTGGTCTGTTAATGACTTCGATGCTGTTCAGTCTGTCTGCATCTTGGGCCAGGGACATCCCAGAAAATTTCGTGTATCAGAACAAGGCTGAGTGTTATTTCACCAACGGCACGGAGCAGGTGCGCTTTGTGGTTAGATACATCTACGATGACCAGGAATTTCTCCGCTTTGACAGCGACGTGGGGAAGTTCCTGGCGGTGACGGAGCTGGGGCAGAGTCAGGCTGAGGACTGGAACAGTCTGGAGGAGATGCTGGAGCAAAATCAAGCTGCCGTGGACACGCTGTGTAGATACAACTACGACATGGGCAAGCCCTTCATGGTGGACAGAAGAGGTGATGCATCCCAGAGTGACCATCTCCCCCTCCAAGACAGACGCCCTGCAGCACCTGCTGGTCTGCTCTGTCACTGGCTTCTATCCAAGCAAGATCAAAGTCACCTGGTTCAAGAATGGGCAGGAGGAGACAGCTGGGATCGTGTCCACGGGTGTGATACAACATGGAGACTGGACCTACCAGACCCTGGTCATGTTGGAAATGGCTCCCCAGAGCAGAGATGTCTACACCTGCAGTGTGGAGCATGCCGGCCTACAGAGCCCCATCAGTGTGGAATGGAGGGCACAGTCTGAATCTGCCCAGAGGAAACTGCTGAGTGGAATTGGAGGCTTTGTCCTGGGGCTGATCTTCCTCAGTGTAGGACTGATCATCCACCTGAAGAACAAGAAAGGTGA

>Bepe_DBB_2

ATGTTGGGATCTCTGTTGGCTGCTGGAAGATTGTTCTTCTGTTAATGACATCAATGCTGTTGGGTTTATCTGCATCTTGGACCAGGGACATCCCAGGTAAAGGACTTCGTGTATCAGTACAAGGGCGAGTGTTACTTCACCAATGGCACGGAGCGGGTGCGGCTTGTGTATAGAGTCATGTACAACGGCGAGGAGAATGCCCGCTTCGACAGCGACGTGGGGCACTTTGTGGCCCTGACGGAGCTGGGGCGGCCCGATGTTGAGTACTGGAACAGTCAGGAGGAGATCCTGGAGGAAGAACGAGGCTACGTGGACACGGTGTGCAGACACAACTACGAGGTAGACAAGCCCTTCACTCTGGACAGAAGAGTCCAGCCCAGAGTGACCATCTCCCCCTCCAAGACAGACGCCCTGCAGCACCTGCTGGTCTGCTCTGTCACTGGCTTCTATCCAAGCAAGATCAAGGTCACCTGGTTCAAGAATGGGCAGGAGGAGACAGCTGGGATCGTGTCCACGGGTGTGATACAACATGGAGACTGGACCTACCAGACCCTGGTCATGTTGGAAATGACTCCCCAGAGCAGAGATGTCTACACCTGCAGGGTGGAGCATGCCAGCCTACAGAGCCCCATCAGTGTGGAATGGAGGGCACAGTCTGAATCTGCCCAGAGCAAACTGCTGAGTGGAATTGGAGGCTTTGTCCTGGGGCTGATCTTCCTCAGTGTAGGGCTGATCATCCACCTGAAGAACAAGAAAGGTGA

>Bepe_DBB_3

ATGGTTGATGTTTGGATCTCTGCTGGCTGCTGGAAGATTGGTCTGTTAACGACATCAATGCTGTTGAGTTTATCTGCATCTTGGGCCAGGGACATCCCAGAGGATTTCGTGTATCAGACCACGTTTCTGTGTTACTTCACCAACGGCACGAAGCGGGTGCGGCTTGTGCATAGAGTCATGTACAACGGCGAGGAGATTGTCCGCTTCGACAGCGACGTGGGGGTGTACGTGGCGGTAACGGAGCTGGGGCGGCCCGATGCTGAGTACTGGAACGGTCAGGCGGAGATCCTGGAGGAATATCGAGGCTACGTGGACACGCTGTGCAGACACAACTACGAGGTAGACAAGCCCTTCACGCTGGACAGAAGAGTGCAGCCCAGAGTGACCATCTCCCCCTCCAAGACAGACGCCCTGCAGCACCTGCTGGTCTGCTCTGTCACTGGCTTCTATCCAAGGAAGATCAAGGTCACCTGGTTCAAGAATGGGCAGGAGGAGACAGCTGGGATCGTGTCCACGGGTGTGATACAACATGGAGACTGGACCTACCAGACCCTGGTCATGTTGGAAATGACTCCCCAGAGCAGAGATGTCTACACCTGCAGTGTGGAGCATGCCAGCCTTCAGAGCCCCATCAGTGTGGAATGGAGGGCACAGTCTGAATCTGCCCAGAGCAAACTGCTGAGTGGAATTGGAGGCTTTGTCCTGGGGCTGATCTTCCTCAGTGTAGGGCTGATCATCCACCTGAAGAACAAGAAAGGTGA

>Bepe_DBB_4

ATGGTTGATGTTTGGATCTCTGCTGGCTGCTGGAAGATTGGTCTGTTAACGACATCAATGCTGTTGAGTTTAACTGCATCTTGGGCCAGGGACATCCCAGAGGATTTCGTGTTTCAGTACAAGTTTGAGTGTTACTTCACCAATGGCACGGAGCACGTGCGCCTTGTGGTTAGAGGGATGTACAATGGCCAGGAATTTGCCCGCTTTGACAGCGACTTGGGGCACTTCGTGGCGGTGACGGAGCTGGGGCGGCCCGATGCTGAGTATTGTAACAGTCAGGAGGAGATCCTGGAGGAATATCGAGCCTACGTGGACACGCTTTGCAGACACAACTACAACACTGACAAGCCCTTCACGGTGGACAGAAGAGTCCAGCCCAGAGTGACCATCTCCCCCTCCAAGACAGACGCCCTGCAGCATCTGCTGGTCTGCTCTGTCACTGGCTTCTATCCAAGCAAGAGCAAAGTCATCTGGTTCAAGAATGGGCAGGAGGAGACAGCTGGGATCGTGTCCACGGGTGTGATACAACATGGAGACTGGACCTACCAGACCCTGGTCATGTTGGAAATGACTCCCCAGAGCAGAGATGTCTACACCTTCAGTGTGGAGCATGCCAGCCTACAGAGTCCCATCAGTGTGGAATGGAGGGCACAGTCTGAATCTACCCAGAGCAAACTGCTGAGTGGAACTGGAGGCTTTGTCCTGGGGCTGATCTTCCTCAGTGTAGGGCTGATCATCCACTTGAAGAACAAGAAAGGTGA

>Bepe_DBB_5

ATGGTTGATGTTTGGATCTCTGCTGGCTGCTGGAAGATTGGTCTGCTAATGACATCGATGCTGTTGAGTTTATCTGCGTCTTGGGCCAGAGACATCCCAGAGGACTTCGTGTATCAGTACAAGGGCGAGTGTTACTTCACCAACGGCACGGAGCGGGTGCGTTTTGTGTATAGAAGCATCTACAACGGCGAGGAGGATGTCCGCTTCGACAGCGACGTGGGGGTGTACGTGGCCCTGACGGAGCTGGGGCGGCCCGATGCTGAGTACTGGAACGGTCAGGAGGATAGCCTGGAGGAATATCGAGGCTACGTGGACACGCTGTGCAGACACAACTACGAGGCAGGCAAGCCCTTCACTCTGGACAGAAGAGTCCAGCCCAGAGTGACCATCTCCCCCTCCAAGACAGACGCCCTGCAGCACCTGCTGGTCTGCTCTGTCACTGGCTTCTATCCAAGCAAGATCAAGGTCACCTGGTTCAAGAATGGGCAGGAGGAGACAGCTGGGATCGTGTCCACGGGTGTGATACAACATGGAGACTGGACCTACCAGACCCTGGTCATGTTGGAAATGACTCCCCAGAGCAGAGATGTCTACACCTGCAGTGTGGAGCATGCCAGCCTTCAGAGCCCCATCAGTGTGGAATGGAGGGCACAGTCTGAATCTGCCCGGAGCAAACTGCTGAGTGGAATTGGAGGCTTTGTCCTGGGGCTGATCTTCCTCAGTGTAGGGCTGATCATCCACCTGAAGAACAAGAAAGGTGAACCATGTAATCAGCTCTGTGACAATTGTTCAGACCTACAAACCATCTGAACAGTACCTGCATGAATCTGATGAAGATGAACCATTCCACATGGACTGTGAAAAGAAGGAAACTGTCTGGCAGCTTCCTGAGTTTGGTCATATCTTCAGTTCCAATTCAGTTCAGATTGGGCTAAGTGACATTGCTGTGGACATGGCTTACTTGAACCAACTTATCAGGCAAACCAACCACACCCAAGCCACCATTGTGACTCCAGAGGTGGCAGTGTTTCCCAAGGAGGACGTGGAACTAGAAGAACCCAGTGTCCTCATCTGCCACATTGATAAGTTCTCCATCCCACTGATCAATGTCACATGGCTGTGCAATGGTGAGTCAGTCACCACAGGAGTATCTGAGACTGCCTTCCTGCCTCAGGATGACTGTTCTTTCCACAAGTTTCATTACCTCACTTTCTTCCTTTCAACTGATGATATTTATGACTGTGCACTTGAACACTGGGGCCTCAAAAACCACTTTTCAAGTAGCCTGAGATGCTAACACCACCATCTGAGACAATGGAGATGCTTATCCCTCTTCTAAGAATGGCTGTGGGCCTGGTGGGCATCATGGTGGCTGCCAGCTTCATTATCAGAGGCTTGTGCTCAGGCAAATGATTCCTGATCCCATAGATCAAGTATAATTTTCTGCAAGAAATTTCTGCAAGAAATCTTTCCAGTCCTCCTTAATCTTGGTGCCTTCCCCTGA

>Bepe_DCB_1

ATGGAACATGTGGAGTTTCTGGGAGGCCCCGTATGACAGTCATACTGATAGTGCTGAGCACACCCATAGACTGAGGTAGGGACATTCCAAAGAATTACCTACATCAGATGCAGTCATCAGATTGGGATCAATGAAACCCAACAAGACTCATCTACAACCAGGTGGAGTTTGTGCACTTTGACAGTGATGTGGGGCTATTTCAGATAAGCTGTGGAAATCTCAAGCCCAGAAATGAAACAGTAAGAAGGAGATAGTTAAGCATGCAAGGTCCATAGTGAATGTGTGCAGACACAATTTTCTTTTATATGACAAATTCACAGGGCAAAGGAAAGTCCAGCCCCTAGTGAAAGTTTTACCCCCAAAGATACAACCACCTGGGCACCACAACCTGCTCCTCTGTTCTGTGATGTTTCTATCCTGATGAGATCAAGATCAATTGATTCAGGATCGCAAAAGAAGAGAAGGTTGGAATCCTGTCCACAGACAGAATTCAGCACAAGGACTGGACCTTCCAAACCCTTGTGGTGCTGGAAATGCCCCCCCAAAGAGGAGATGACTTTACTTGCCATGTGGACCATGTCAGCTTGCAGAGCCCTATCACTGTAAACTGGACACATTCTGAATCTTCCTGGACTAAGAGTCTGACTGAAATTGAGGGCTTGGTATTTGGACTGATCTTACTTGGAGTGAGACTTTACTTGTTATCCACCTCAGAAATTTGAAAGATTCCTATTCTGGTACCAAAGAAGACCCAGATTTTGAAGGGACTGTGAACATTGCCTCATTGCAACAAGATTTTCCCAGAACTGTTGCCCAGTCCTAG

>Bepe_DMB_1

ATGACATTACTCTACCTGCTACTAGTGGGCTTCAGCCTGGGTTTTTCAGGAACAGGGGGCTTTGTGACTCATGTGGAGAGTGGCTGTATACTGGATGAAGAAGGATCCGTAAAGGACTTCGAATATTGTATCTCCTTCAACAAGGATGTGTTAACCTGCTGGGACTCAGATAGTAACAAGATGGTCACTGTTGATTTTGGAATACTGCGTCCATTAGCTGACGAACTTTCTGATGCTCTCAATAATGATACTGCTTTGATAAACCGCCTAAGCCAAGGATTCCAGGACTGTGCCAGTCACACAAAGCCCTTCTGGGGATCACTGACCCACAGGACACGGCCACCATCAGTACAAGTAGCTCAGGTCACACCATTCAACACAAGGGAGTCAGTGATGCTGGCCTGTTATGTATGGGGCTTCTATCCTGCTGATGTGGCCATTTCATGGTTGAAGAATGGGCAGCCTGTCCCACACAGCGGCATCCAGAAGGCTGTACAATCCAATGGGGACTGGACTTATCAGACACGATCCTACTTGGCCCTTACCCCCTCTAGTGGGGATATTTACACTTGCTCTGTAGAGCACAGTGGGACTTCCCAGATCATCTTACAGACCTGGACACCTGGCCTCTCCCTGAAGCAGATAGTGAAGATCTCTGTGTCTGTATTGACTCTGAGCTTTGGTGTTATCATCTTCTTCCTTGGCCTGGTTGCACGCAGAAAGGCTGGCTTCTCTGACTATTCTCCTCTCTCGGGGTCCAATTATCCTGAAGGTAA
